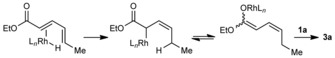# Chain Walking of Allylrhodium Species Towards Esters During Rhodium‐Catalyzed Nucleophilic Allylations of Imines

**DOI:** 10.1002/anie.201508964

**Published:** 2015-12-03

**Authors:** Jose I. Martínez, Joshua J. Smith, Hamish B. Hepburn, Hon Wai Lam

**Affiliations:** ^1^ School of ChemistryUniversity of NottinghamUniversity ParkNottinghamNG7 2RDUK

**Keywords:** allyltrifluoroborates, asymmetric catalysis, imines, isomerization, rhodium

## Abstract

Allylrhodium species derived from δ‐trifluoroboryl β,γ‐unsaturated esters undergo chain walking towards the ester moiety. The resulting allylrhodium species react with imines to give products containing two new stereocenters and a *Z*‐alkene. By using a chiral diene ligand, products can be obtained with high enantioselectivities, where a pronounced matched/mismatched effect with the chirality of the allyltrifluoroborate is evident.

The migration of metal centers along carbon chains occurs in several important reactions.^1^, ^2^, ^3^, ^4^, ^5^, ^6^, ^7^ Many of these migrations take place by β‐hydride elimination and hydrometalation sequences, in which the direction of travel is controlled by thermodynamics, a ligand, or a nearby functional group. With few exceptions,4b–4f these migrations involve simple alkylmetal species. The ability to chain walk a metal together with a second functional group has significant synthetic opportunities, but this mode of reactivity remains largely underdeveloped. Herein, we describe, to our knowledge, the first examples of allylrhodium chain walking, and its application in the preparation of enantioenriched products.

During our studies of enantioselective Rh‐catalyzed nucleophilic allylations of imines,^8^ the reaction of imine **1 a** with racemic allyltrifluoroborate **2 a** 9 in the presence of [{Rh(cod)Cl}_2_] (1.5 mol %) and *i*PrOH (5.0 equiv) was conducted (Scheme 1). Surprisingly, allylation at the α‐ or γ‐carbon atoms relative to the boron atom of **2 a** was not observed. Instead, this reaction gave homoallylic sulfamates **3 a** (68 % yield) and **4 a** (6 % yield), each in >95:5 d.r. (Scheme 1).^10^, ^11^, ^12^ This result suggests the reactive intermediates are allylrhodium species **5** and **6**, formed from migration of the allylrhodium species generated initially from transmetalation of **2 a** with rhodium.

**Scheme 1:**
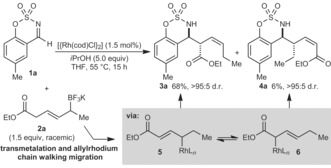
Discovery of allylrhodium chain walking.

The scope of this unexpected reaction was extended to include aldimines bearing methyl, methoxy, bromo, or dioxozole groups, which gave products with high diastereoselectivities in 65–72 % yield (Table 1, entries 1–5). Ketimines containing linear alkyl groups at the imine carbon were also effective (entries 6–9). However, an isopropyl‐substituted imine was recovered unchanged (entry 10). With one exception (entry 3), no products of allylation at the α‐ or γ‐carbons relative to the boron atom of **2 a** were obtained. Furthermore, except for the reactions producing **3 a** and **3 d** (entries 1 and 4), the alternative regioisomers were difficult to detect by ^1^H NMR spectroscopy.

**Table 1:** Investigation of imine scope.^[a]^ 
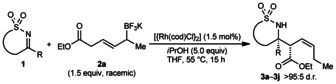

Entry	Product		R	Yield [%]^[b]^
1 2 3 4	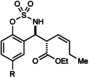	**3 a 3 b 3 c 3 d**	Me H OMe Br	68^[c]^ 72 65^[d]^ 65^[e]^
5	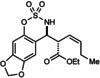	**3 e**		65
6 7 8 9 10	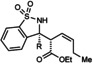	**3 f 3 g 3 h 3 i 3 j**	Me Et *n*Bu (CH_2_)_3_Ph *i*Pr	65 53 54 55 <5

[a] Reactions were conducted using 0.30 mmol of **1**. The diastereomeric ratios were confirmed by ^1^H NMR analysis of the unpurified reactions. [b] Yield of isolated products. [c] The regioisomer **4 a** was isolated in 6 % yield (Scheme 1). [d] In the unpurified reaction, traces of a product derived from allylation without chain walking were detected. [e] Isolated as an 87:13 mixture of **3 d** with the regioisomeric product **4 d**. See Ref. 13.

Next, the potassium allyltrifluoroborate was varied (Table 2). As well as ethyl esters (Table 1) and benzyl esters (Table 2, entries 1–5, 7, and 8), a 2‐naphthyl ester was accommodated (Table 2, entry 6). Regarding the substituent α to the boron atom, alkyl (entries 1, 2, 7, and 8) and chloroalkyl groups (entry 3) were tolerated. Product **3 l** was isolated along with a product of allylation without chain walking, in a 95:5 ratio (entry 2).^13^ Alkyl substituents containing phenyl or benzyloxy groups resulted in lower conversions and yields (entries 4 and 5).

**Table 2:** Investigation of allyltrifluoroborate scope.^[a]^ 
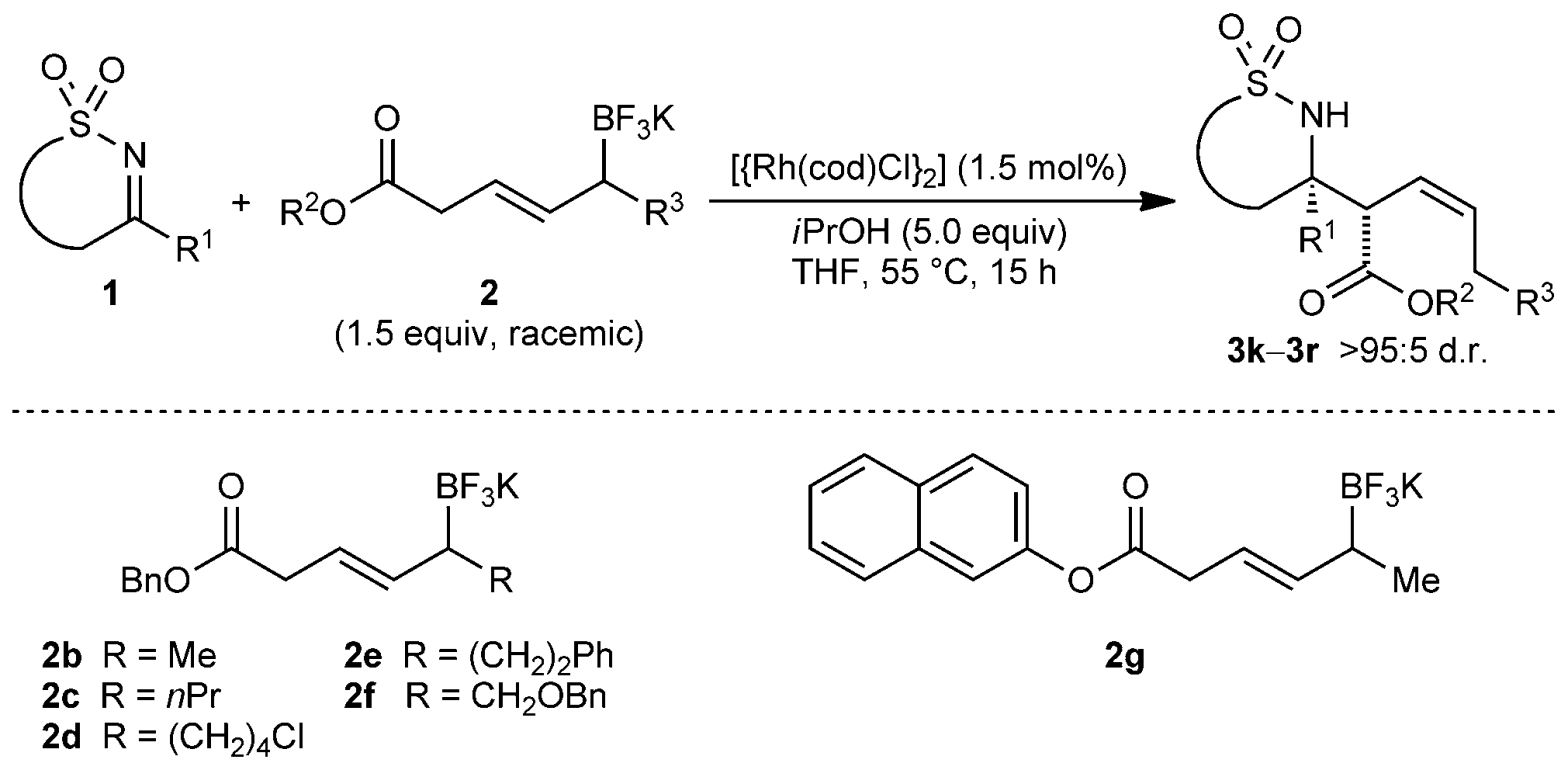

Entry	Product		R	Yield [%]^[b]^
1 2 3 4^[d]^ 5^[d]^	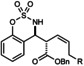	**3 k 3 l 3 m 3 n 3 o**	Me *n*Pr (CH_2_)_4_Cl (CH_2_)_2_Ph CH_2_OBn	69 70^[c]^ 63 36 (59)^[e]^ (53)^[e,f]^
6^[d]^	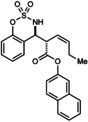	**3 p**		62
7 8	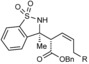	**3 q 3 r**	Me *n*Pr	67 58

[a] Reactions were conducted using 0.30 mmol of **1**. The diastereomeric ratios were confirmed by ^1^H NMR analysis of the unpurified reactions. [b] Yield of isolated products. [c] Isolated as a 95:5 mixture of **3 l** and the product of allylation without allylrhodium chain walking. See Ref. 13. [d] Using 2.5 mol % of [{Rh(cod)Cl}_2_]. [e] Yields in parentheses were determined by ^1^H NMR spectroscopy using 1,3,5‐trimethoxybenzene as an internal standard. [f] Attempts to purify **3 o** by column chromatography were unsuccessful. A pure sample was obtained by preparative TLC.

The reaction of **1 b** with allyltrifluoroborate **2 h**, in which boron is bonded to a primary rather than a secondary carbon, gave not only **3 s**, but also a significant quantity of product **7** in 80:20 d.r., derived from allylation without chain walking [Eq. [Link]].^14^ Products **3 s** and **7** could not be completely separated by column chromatography, and their yields were determined by ^1^H NMR analysis using an internal standard.
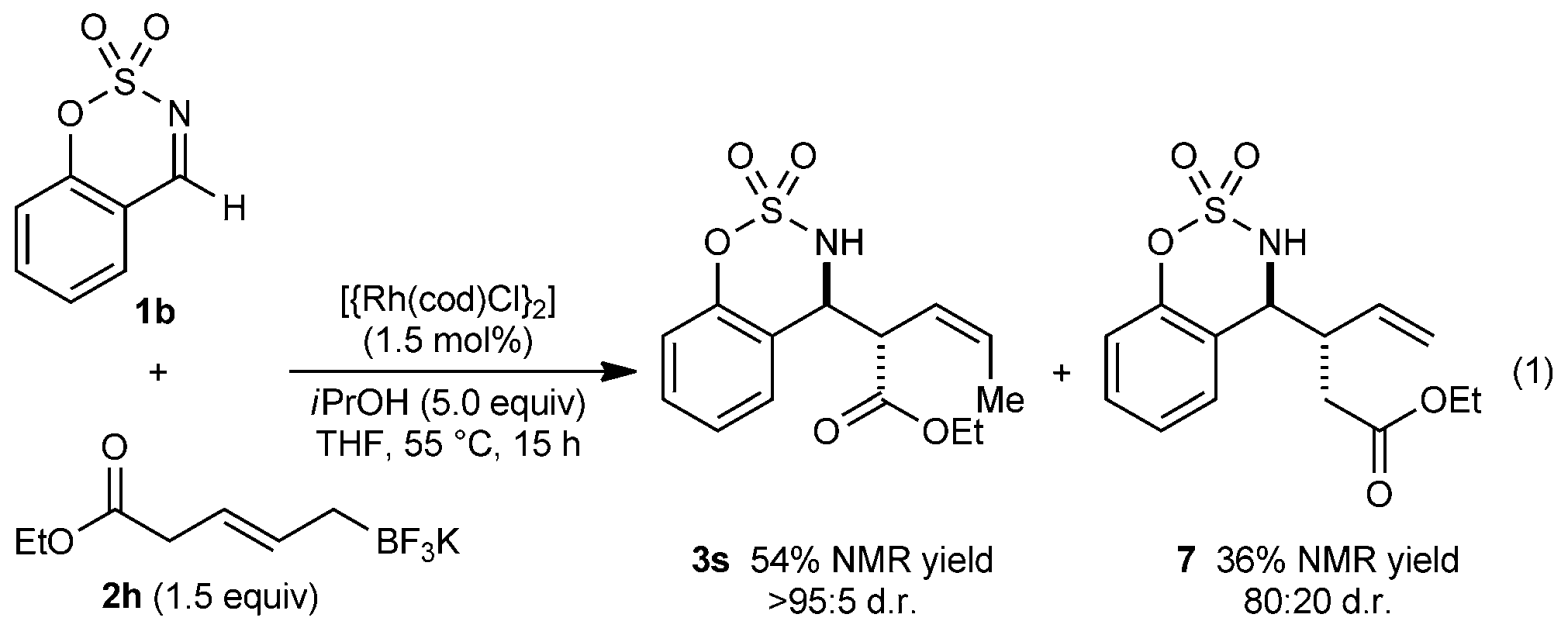



Interestingly, the reaction of *Z*‐allyltrifluoroborate **8** 9 with aldimine **1 b** gave **3 b** in 70 % yield (Scheme 2, top), which is the same product obtained from the corresponding *E*‐isomer **2 a** (Table 1, entry 2). Furthermore, despite possessing a substitution pattern different to all allyltrifluoroborates employed until this point, allyltrifluoroborate **9** reacted in the same manner to give **3 t** (Scheme 2, bottom).^10^ These results suggest that regardless of the geometrical or positional isomerism of the allyltrifluoroborate within the β to δ carbons, the reactions proceed through common types of allylrhodium intermediates. However, homoallylic boron reagents were unreactive.^13^, ^15^

**Scheme 2:**
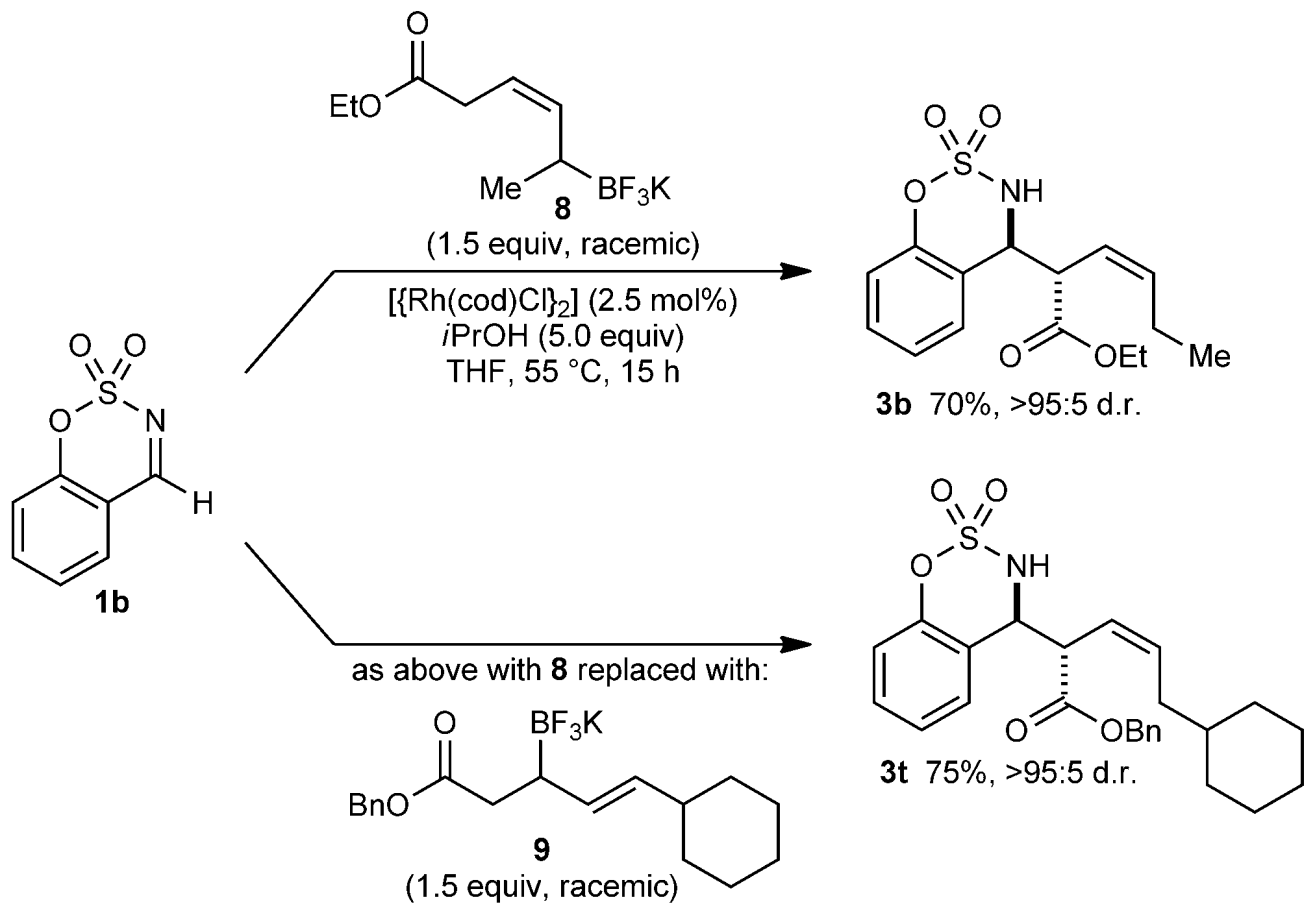
Effect of geometrical and positional isomerism of the allyltrifluoroborate.

Because these reactions provide chiral products from chiral substrates, we investigated whether enantioenriched allyltrifluoroborates would give enantioenriched products. However, the reactions of (*R*)‐**2 a** (94 % *ee*)^9^ with aldimine **1 b** and ketimine **1 f** gave (*S*,*S*)‐**3 b** and (*S*,*S*)‐**3 f**, respectively, with low‐to‐moderate enantiomeric excesses (Scheme 3). Although chain walking of alkylmetal species can proceed with high stereospecificity,7c poor absolute stereochemical transfer is observed in the reactions described herein.

**Scheme 3:**
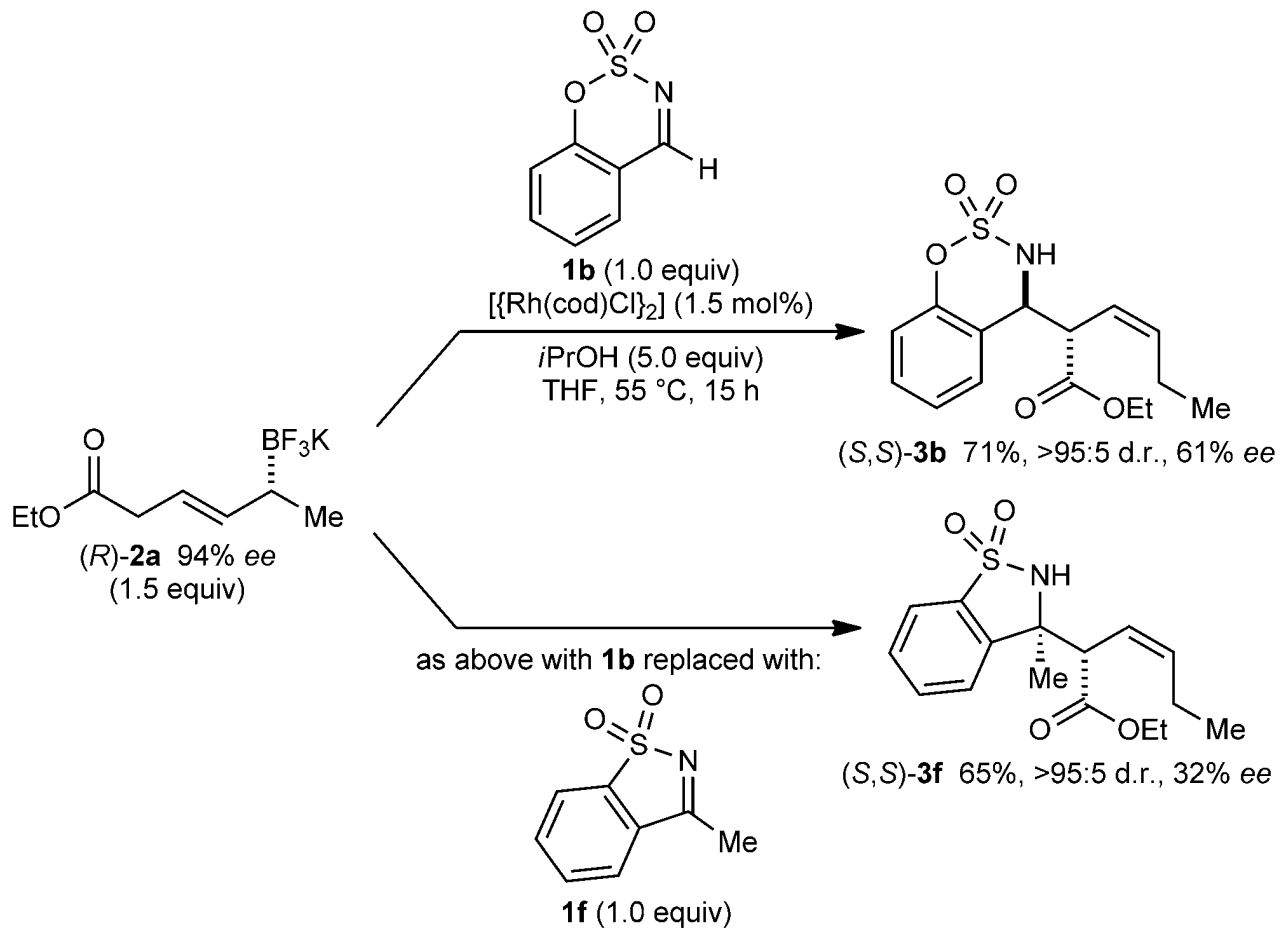
Investigation of absolute stereochemical transfer with (*R*)‐**2 a**.

Next, chiral rhodium complexes were investigated for their ability to provide enantioenriched products from racemic allyltrifluoroborates (Scheme 4).8b Although several chiral dienes^16^ gave poor conversions^13^ in the reaction of aldimine **1 b** with **2 a**, diene **L1** 17 gave (*S*,*S*)‐**3 b** in 72 % yield and 98 % *ee*. Several other products (*S*,*S*)‐**3 k**, (*S*,*S*)‐**3 m**, and (*S*,*S*)‐**3 p** were also prepared in the same manner. However, the yields of some of these reactions were low, and the scope is more limited than when using [{Rh(cod)Cl}_2_]. For example, enantioselective additions to ketimines were unsuccessful.

**Scheme 4:**
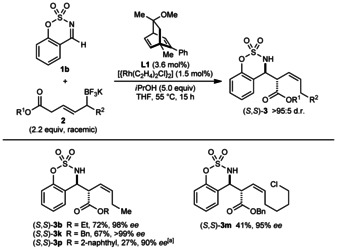
Enantioselective allylations. Reactions were conducted using 0.30 mmol of **1 b**. The diastereomeric ratios were confirmed by ^1^H NMR analysis of the unpurified reactions. Yields are of isolated products. Enantiomeric excesses were determined by HPLC analysis on a chiral stationary phase. [a] Using 2.5 equivalents of allyltrifluoroborate **2 g**.

Interestingly, a pronounced matched/mismatched effect was observed with enantioenriched allyltrifluoroborates. The reaction of **1 b** with (*R*)‐**2 a** (94 % *ee*) using chiral diene **L1** gave (*S*,*S*)‐**3 b** with results identical to the reaction using racemic **2 a** (Scheme 5, top; compare with Scheme 4). However, the corresponding reaction with (*S*)‐**2 a** (94 % *ee*) gave a complex mixture; although **3 b** was detected in small but unquantifiable amounts by ^1^H NMR analysis, it could not be isolated. Currently, it is unclear which steps of the proposed mechanism (see below) are rendered inefficient by the stereochemical mismatch of the ligand and the allyltrifluoroborate.

**Scheme 5:**
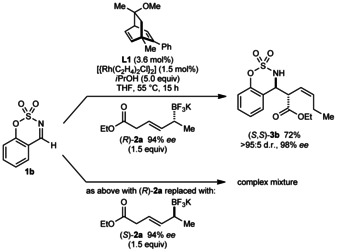
Investigation of matched/mismatched effects.

A proposed mechanism, using imine **1 a** and allyltrifluoroborate **2 a** as representative substrates, is shown in Scheme 6. The reaction of **2 a** with *i*PrOH can reversibly generate a mixed alkoxide/fluoride boron ate complex **11**, which transmetalates with rhodium complex **10** 18, 19 to give interconverting allylrhodium species **12** and **13**. β‐Hydride elimination of **13** then gives a rhodium hydride species bound to ethyl sorbate (as in **14**).^20^, ^21^ Hydrorhodation of the alkene distal to the ester then provides interconverting allylrhodium species **5** and **6**. A possible driving force for this chain walking migration is the formation of a more stable, more conjugated allylrhodium species **5**. Nucleophilic allylation of **1 a** by **5** through a chairlike conformation **15**, in which the ethyl group is pseudoaxial to avoid unfavorable interactions with the cyclooctadiene ligand,8c, 22 gives **16**.^23^ Finally, protonolysis of **16** with HX (X=Cl, F, or O*i*Pr) releases the product **3 a** and regenerates **10**. The minor regioisomer **4 a** is the result of allylation of **1 a** with allylrhodium species **6**.

**Scheme 6:**
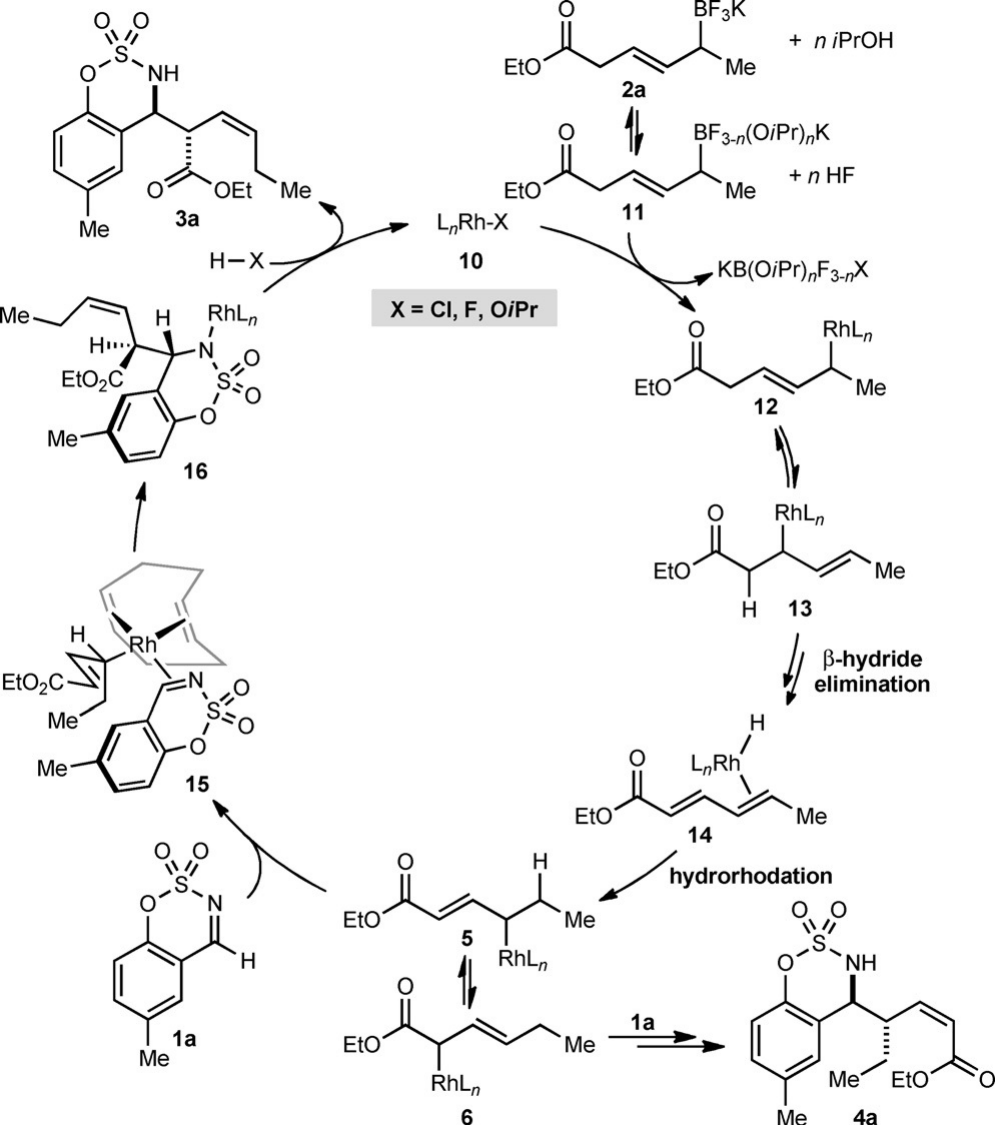
Proposed catalytic cycle.

Support for this mechanism was provided by the reaction of aldimine **1 b**, allyltrifluoroborate **2 c** (1.5 equiv), and ethyl sorbate (**17**, 1.5 equiv), using [{Rh(C_2_H_4_)_2_Cl}_2_] as a precatalyst [Eq. [Link]]. This reaction gave mostly unreacted **1 b** and **17**, along with unidentified products resulting from decomposition of **2 c**. However, by HPLC‐MS, small quantities of the expected product **3 l** derived from allyltrifluoroborate **2 c** (0.4 % yield), the crossover product **3 a** derived from ethyl sorbate (**17**, 3.4 % yield), and α,β,γ,δ‐unsaturated benzyl ester **18** (2.7 % yield) were also detected.^13^

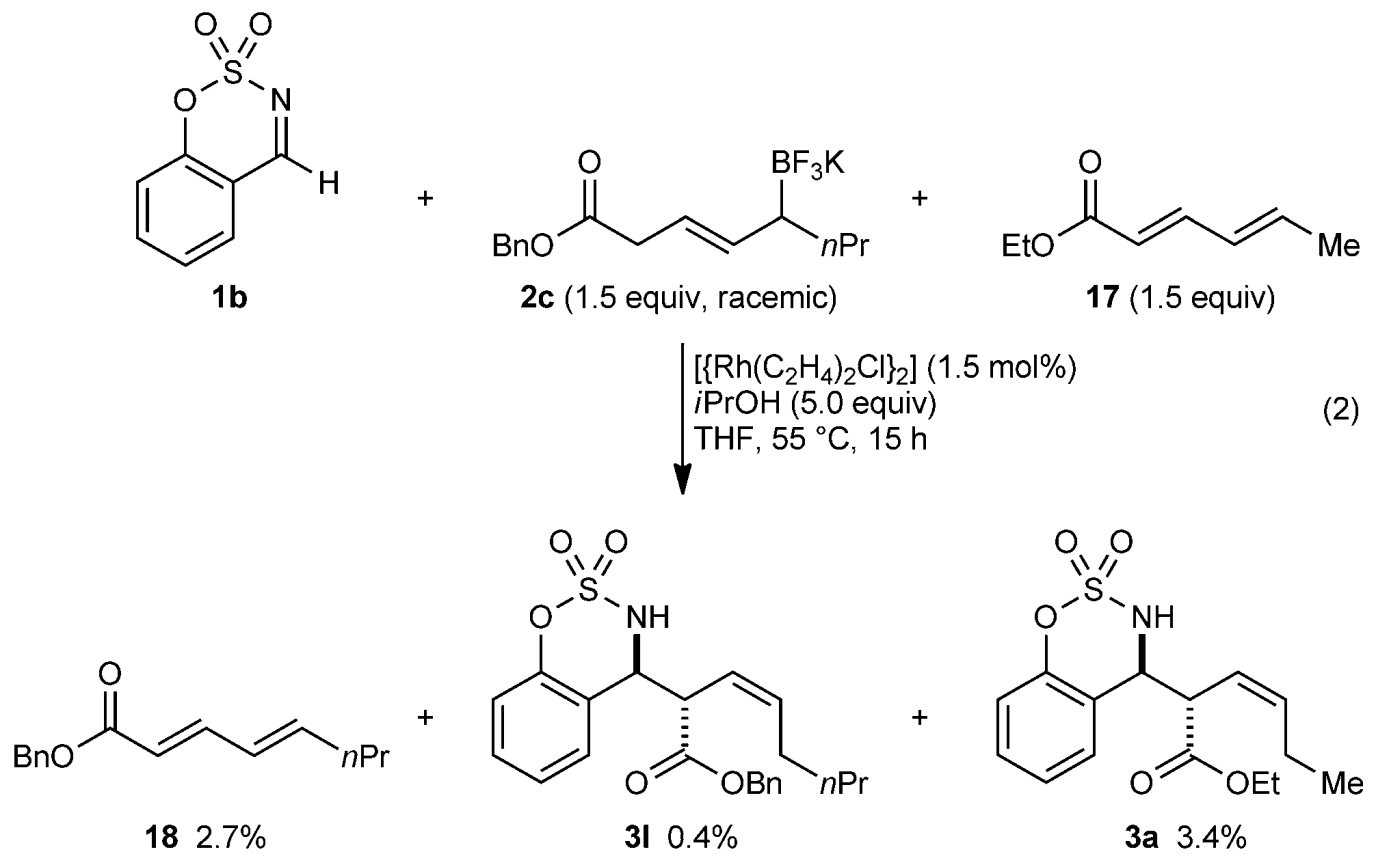



Presumably, the initial catalytic species in this reaction is a complex of rhodium and ethyl sorbate (**17**), possibly the s‐*cis*‐η^4^ complex **19**, which reacts with **2 c** according to the mechanism shown in Scheme 6 to give the rhodium hydride **20** (Scheme 7). Hydrorhodation of the α,β,γ,δ‐unsaturated benzyl ester would give allylrhodium species **21**, which reacts with **1 b** to give the expected product **3 l**. Alternatively, a structural reorganization of **20** could give **22**, which can then undergo hydrorhodation of ethyl sorbate to give allylrhodium species **5** and the crossover product **3 a**.

**Scheme 7:**
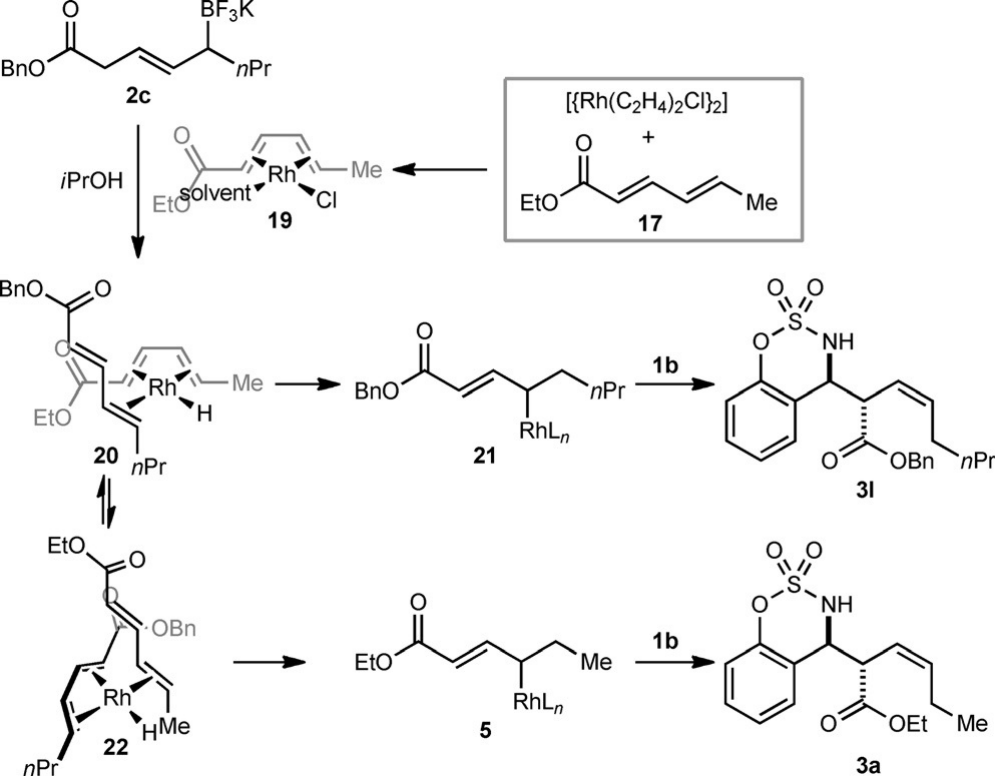
Rationale for the formation of crossover product **3 a**.

In summary, we have reported the chain walking of allylrhodium species derived from δ‐trifluoroboryl β,γ‐unsaturated esters during the rhodium‐catalyzed nucleophilic allylation of imines, which gives products with two new stereocenters and a *Z*‐alkene. Enantioselective catalysis is possible using a chiral diene ligand, where a strong matched/mismatched effect was observed. Further exploration of this new mode of reactivity is underway in our laboratories.

## Supporting information

As a service to our authors and readers, this journal provides supporting information supplied by the authors. Such materials are peer reviewed and may be re‐organized for online delivery, but are not copy‐edited or typeset. Technical support issues arising from supporting information (other than missing files) should be addressed to the authors.

Supplementary
