# Supplementary material for: Chain Walking of Allylrhodium Species Towards Esters During Rhodium‐Catalyzed Nucleophilic Allylations of Imines
Source: Angew Chem Int Ed Engl. 2015 Dec 3;55(3):1108–12. doi: 10.1002/anie.201508964 (PMC4736453; doi:10.1002/anie.201508964)
Supplement: Supplementary file 1 — Supplementary [file ANIE-55-1108-s001.pdf]

Angewandte Chemie
Eine Zeitschrift der Gesellschaft Deutscher Chemiker

Supporting Information

Chain Walking of Allylrhodium Species Towards Esters During Rhodium-Catalyzed Nucleophilic Allylations of Imines

*Jose I. Martínez, Joshua J. Smith, Hamish B. Hepburn, and Hon Wai Lam**

anie_201508964_sm_miscellaneous_information.pdf

Supporting Information

Contents **Page**

1. General Information 2
2. Preparation of Cyclic Imines 3
3. Preparation of Potassium Allyltrifluoroborates 4
4. Preparation of Homoallylic Boron Reagents 16
5. Racemic Rhodium-Catalyzed Allylations of Cyclic Imines 16
6. Investigation of Absolute Stereochemical Transfer with (*R*)-**2a** 30
7. Enantioselective Rhodium-Catalyzed Allylations of Cyclic Imines 32
8. Crossover Experiment 38
9. NMR Spectra 43

1. General Information

All commercially available reagents were used as received. THF was dried and purified by passage through activated alumina columns using a solvent purification system. "Petroleum ether" refers to Sigma-Aldrich product 24587 (petroleum ether boiling point 40–60 °C). Thin layer chromatography (TLC) was performed on Merck DF-Alufoilien $60F_{254}$ 0.2 mm precoated plates. Product spots were visualized by UV light at 254 nm, and subsequently developed using potassium permanganate or vanillin solution as appropriate. Flash column chromatography was carried out using silica gel (Fisher Scientific 60Å particle size 35–70 micron) or using an Interchim Puriflash 430 Series automated purification system with IR-50SI 50µm pre-packed columns. Melting points were recorded on a Gallenkamp melting point apparatus and are uncorrected. Infra-red spectra were recorded on a Nicolet Avatar 360 FT instrument on the neat compound using an attenuated total reflection (ATR) accessory with a diamond crystal and a germanium sample plate, or on a Bruker Tensor 27 FT instrument as a $CHCl_3$ solution. NMR spectra were acquired on Bruker AVA500, Bruker AVA400, Bruker AV(III)400, Bruker DPX400, or Bruker DPX300 spectrometers. ^{1}H and ^{13}C NMR spectra were referenced to external tetramethylsilane via the residual protonated solvent (^{1}H) or the solvent itself (^{13}C). All chemical shifts are reported in parts per million (ppm). For $CDCl_3$, the shifts are referenced to 7.27 ppm for ^{1}H NMR spectroscopy and 77.0 ppm for ^{13}C NMR spectroscopy. For $(CD_3)_2CO$ the shifts are referenced to 2.05 ppm for ^{1}H NMR spectroscopy and 29.84 ppm for ^{13}C NMR spectroscopy. For CD_3OD the shifts are referenced to 3.31 ppm and ^{1}H NMR spectroscopy and 49.00 ppm for ^{13}C NMR spectroscopy. For CD_3CN the shifts are referenced to 1.94 ppm for ^{1}H NMR spectroscopy and 118.26 ppm for ^{13}C NMR spectroscopy. Abbreviations used in the description of resonances are: s (singlet), d (doublet), t (triplet), q (quartet), app (apparent), br (broad), m (multiplet). Coupling constants (J) are quoted to the nearest 0.1 Hz. Assignments were made using the DEPT sequence with secondary pulses at 90° and 135°. Proton-decoupled ^{19}F NMR spectra were recorded on a Bruker DPX300 (282 MHz), Bruker AV(III)400 (376 MHz), Bruker AVA400 (376 MHz) or Bruker DPX400 (376 MHz) spectrometer. Chemical shifts (δ) are quoted in parts per million (ppm) downfield of $CFCl_3$ (δ = 0 ppm), using trifluoroacetic acid as internal standard (CF_3COOH at –76.55 ppm). High resolution mass spectra were recorded using electrospray ionization (ESI) or electron impact ionization (EI) techniques. Optical rotations were performed on a Bellingham and Stanley ADP 400 polarimeter. Chiral HPLC analysis was performed on an Agilent 1200 series instrument using 4.6 x 250 mm columns.

2. Preparation of Cyclic Imines

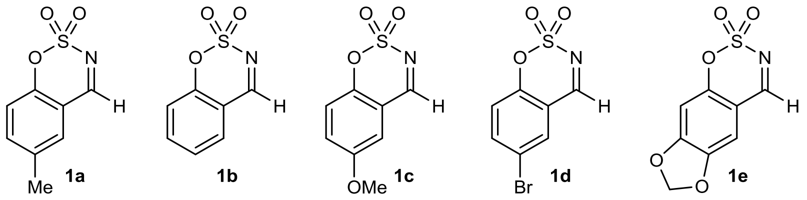

The benzoxathiazine-2-2-dioxides **1a**, **1b**, **1c**, **1d**, and **1f** were prepared according to a previously reported literature procedures.[1]

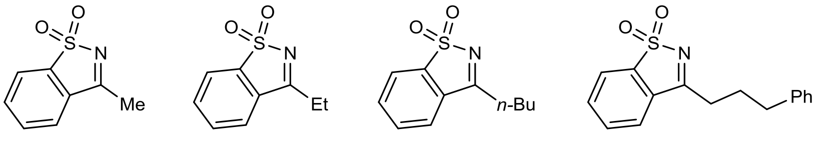

1f **1g** **1h** **1i**

The benzoisothiazole-1-1-dioxides **1f**, **1g**, **1h**, and **1i** were prepared according to previously reported procedures.[2]

3-Isopropylbenzo[*d*]isothiazole 1,1-dioxide (1j)

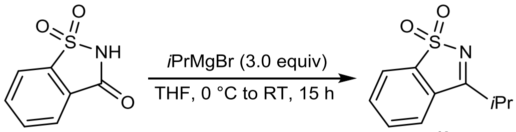

1j

Saccharin (1.83 g, 10.0 mmol) was dissolved in THF (25 mL) under N_2 at 0 °C. Isopropylmagnesium chloride (2.0 M in THF, 15 mL, 30 mmol) was slowly added and the solution was stirred for 15 h while slowly warming to room temperature. The reaction was poured carefully onto ice and the solution was acidified (2 M HCl). The aqueous layer was extracted with Et_2O (4 x 50 mL) and the organic layers were combined, washed with brine (50 mL), dried over $MgSO_4$, and concentrated *in vacuo* to leave a white solid. Recrystallization of the solid from Et_2O gave the ketimine **1j** (890 mg, 43%) as a pale yellow solid that displayed spectroscopic data consistent with those reported previously.[3] ^{13}C NMR data were not provided in previously reported data and are reported here as follows: ^{13}C NMR (100.6 MHz, $CDCl_3$) δ 180.4 (C=O), 140.2 (C), 133.8 (CH), 133.4 (CH), 130.6 (C), 124.0 (CH), 122.5 (CH), 30.5 (CH), 17.8 (2 x CH_3).

1. Y. Luo, A. J. Carnell, H. W. Lam, *Angew. Chem., Int. Ed.* **2012**, *51*, 6762–6766.
2. H. B. Hepburn, H. W. Lam, *Angew. Chem., Int. Ed.* **2014**, *53*, 11605–11610.
3. (a) C. K. F. Hermann, J. A. Campbell, T. D. Greenwood, J. A. Lewis, J. F. Wolfe, *J. Org. Chem.* **1992**, *57*, 5328–5334. (b) H. Teeninga, J. B. F. N. Engberts, *J. Org. Chem.* **1983**, *48*, 537–542.

3. Preparation of Potassium Allyltrifluoroborates

The majority of the allylboron reagents used in this study were prepared by the copper-catalyzed 1,6-boration of α,β,γ,δ-unsaturated esters[4] followed by conversion of the resulting pinacol boronic esters into potassium allyltrifluoroborates.

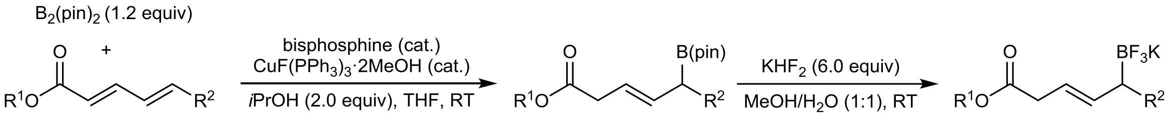

3.1 Alkenylboron Reagents Required for Preparation of α,β,γ,δ-Unsaturated Esters

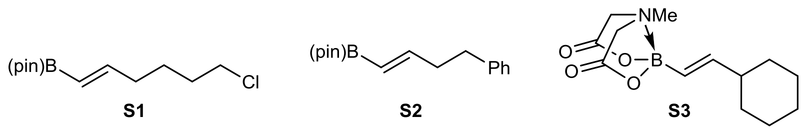

Alkenylboron reagents **S1** and **S3** are commercially available. Alkenylboron reagent **S2** was prepared according to a previously reported procedure.[5]

2-[(*E*)-3-(Benzyloxy)prop-1-en-1-yl]-4,4,5,5-tetramethyl-1,3,2-dioxaborolane (S4)

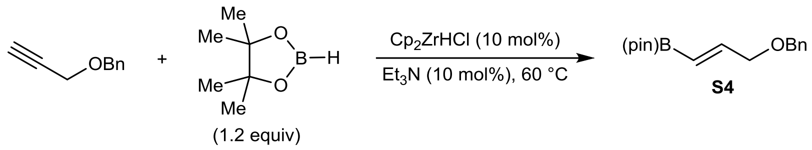

Following a slightly modified literature procedure,[6] zirconocene hydrochloride (516 mg, 2.00 mmol) was added to an oven-dried flask fitted with a reflux condenser containing [(prop-2-yn-1-yloxy)methyl]benzene[7] (2.92 g, 20.0 mmol), which was then purged with N_2. Et_3N (278 μL, 2.00 mmol) was added followed by dropwise addition of pinacolborane (3.48 mL, 24.0 mmol). The reaction mixture was heated to 60 °C for 16 h. The mixture was filtered through a short silica plug eluting with Et_2O/petroleum ether (1:4) and the filtrate was concentrated *in vacuo* to leave a 4:1 mixture of the *alkenylboronic ester* **S4** and pinacol as a colorless oil which was used without further purification (5.27 g, 80% purity, 19.2 mmol, 87%); R_f 0.78 (20% EtOAc/petroleum ether); IR 2978, 2931, 2854, 1644, 1454, 1371, 1359, 1343, 1324, 1144 cm^{-1}; ^{1}H NMR (400 MHz, $CDCl_3$) δ 7.40–7.24 (5H, m, Ar**H**), 6.69 (1H, dt, *J* = 18.2, 4.7 Hz, =C**H**CH_2), 5.77 (1H, dt, *J* = 18.2, 1.8 Hz, C**H**=CHCH$_2$), 4.54 (2H, s, C**H**$_2$Ph), 4.12 (2H, dd, *J* = 4.7, 1.8 Hz, =CHC**H**$_2$), 1.28 (12H, s, 2 x C(C**H**$_3$)$_2$); ^{13}C NMR (100.6 MHz, $CDCl_3$) δ 149.1 (CH), 138.2 (C), 128.3 (2 x CH), 127.6 (2 x CH), 127.5 (CH), 83.3 (2 x C), 72.3 (CH_2), 71.7 (CH_2), 24.8 (4 x CH_3), the carbon (CH) adjacent to

4. Y. Luo, I. D. Roy, A. G. E. Madec, H. W. Lam, *Angew. Chem., Int. Ed.* **2014**, *53*, 4186–4190.
5. G. Pattison, G. Piraux, H. W. Lam, *J. Am. Chem. Soc.* **2010**, *132*, 14373–14375.
6. Y. D. Wang, G. Kimball, A. S.; Prashad, Y. Wang, *Tetrahedron Lett.* **2005**, *46*, 8777–8780.
7. D. Farran, A. M. Z. Slawin, P. Kirsch, D. O'Hagan, *J. Org. Chem.* **2009**, *74*, 7168–7171.

boron was not observed due to quadrupolar coupling effects of ^{11}B; HRMS (ESI) Exact mass calculated for $C_{16}H_{23}BO_3Na$ $[M+Na]^+$: 297.1632, found: 297.1631.

3.2 Preparation of α,β,γ,δ-Unsaturated Esters

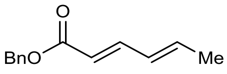

S5

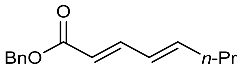

18

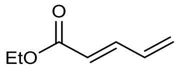

S6

α,β,γ,δ-Unsaturated esters **S5**,[4,8] **18**,[4] and **S6**[9] were prepared according to previously reported procedures.

Suzuki Coupling: General Procedure A

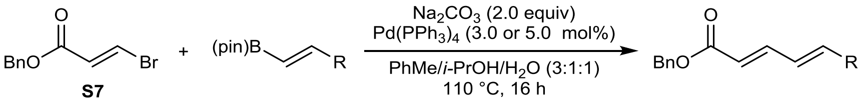

S7

Following a slightly modified literature procedure,[4] a solution of alkenyl bromide **S7**[4] (1.0 equiv), the appropriate alkenylboronic ester (1.2 equiv), $Pd(PPh_3)_4$ (3.0 or 5.0 mol%), and Na_2CO_3 (2.0 equiv) in toluene (12 mL/mmol), *i*-PrOH (4 mL/mmol) and H_2O (4 mL/mmol) was degassed by bubbling N_2 (10 min) before heating to 110 °C for 16 h. The reaction mixture was cooled to room temperature, washed with saturated aqueous $NaHCO_3$ solution (10 mL/mmol) followed by saturated aqueous NH_4Cl solution (10 mL/mmol). The organic layer was dried (Na_2SO_4) and concentrated *in vacuo*. Purification of the residue by flash column chromatography gave the α,β,γ,δ-unsaturated ester.

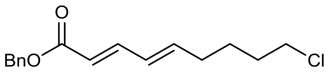

Benzyl (2*E*,4*E*)-9-chlorononа-2,4-dienoate (S8). The title compound was prepared according to General Procedure A from alkenylboronic ester **S1** (4.84 g, 19.8 mmol), alkenyl bromide **S7** (3.98 g, 16.5 mmol), $Pd(PPh_3)_4$ (0.57 g, 0.50 mmol), and Na_2CO_3 (3.50 g, 33.0 mmol), and purified by flash column chromatography (5% Et_2O/cyclohexane) to give a colorless oil (3.91 g, 85%) that displayed spectroscopic data consistent with those reported previously.[4]

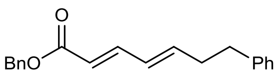

Benzyl (2*E*,4*E*)-7-phenylhepta-2,4-dienoate (S9). The title compound was prepared according to General Procedure A from alkenylboronic ester **S2** (2.48 g, 9.60 mmol), alkenyl bromide **S7** (1.93 g, 8.0 mmol), $Pd(PPh_3)_4$ (0.46 g, 0.40 mmol), and Na_2CO_3 (1.70 g, 16.0 mmol), and purified by flash column chromatography (2.5%

8. A. Mori, Y. Miyakawa, E. Ohashi, T. Haga, T. Maegawa, H. Sajiki, *Org. Lett.* **2006**, *8*, 3279–3281.
9. J. W. Wrigglesworth, B. Cox, G. C. Lloyd-Jones, K. I. Booker-Milburn, *Org. Lett.* **2011**, *13*, 5326–5329.

Et_2O/cyclohexane) to give a colorless oil (2.06 g, 88%) that displayed spectroscopic data consistent with those reported previously.[4]

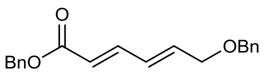

Benzyl (2*E*,4*E*)-6-(benzyloxy)hexa-2,4-dienoate (S10). The title compound was prepared according to General Procedure A from alkenylboronic ester **S4** (2.63 g, 9.60 mmol), alkenyl bromide **S7** (1.93 g, 8.0 mmol), $Pd(PPh_3)_4$ (0.46 g, 0.40 mmol), and Na_2CO_3 (1.70 g, 16.0 mmol), and purified by flash column chromatography (0% to 10% EtOAc/petroleum ether) to give a colorless oil (1.89 g, 77%). R_f 0.69 (20% EtOAc/petroleum ether); IR 3032, 2850, 2361, 1712 (C=O), 1646, 1619, 1454, 1258, 1225, 1134 cm^{-1}; ^1H NMR (400 MHz, $CDCl_3$) δ 7.46–7.28 (11H, m, Ar**H** and O=CCH=C**H**), 6.45 (1H, ddtd, *J* = 15.3, 11.1, 1.6, 0.7 Hz, C**H**=CHCH$_2$), 6.21 (1H, dtdd, *J* = 15.3, 5.2, 0.6, 0.6 Hz, CH=C**H**CH$_2$), 5.96 (1H, ddd, *J* = 15.4, 0.7, 0.6 Hz, O=CC**H**=CH), 5.21 (2H, s, PhC**H**$_2$O$_2$C), 4.56 (2H, s, CH$_2$OC**H**$_2$Ph), 4.17–4.14 (2H, m, C**H**$_2$CH=CH); ^{13}C NMR (100.6 MHz, $CDCl_3$) δ 166.7 (C), 144.3 (CH), 138.9 (CH), 137.8 (C), 136.0 (C), 129.1 (CH), 128.5 (2 x CH), 128.4 (2 x CH), 128.2 (3 x CH), 127.8 (CH), 127.7 (2 x CH), 121.1 (CH), 72.6 (CH_2), 69.6 (CH_2), 66.2 (CH_2); HRMS (ESI) Exact mass calculated for $C_{20}H_{20}O_3Na$ $[M+Na]^+$: 331.1305, found: 331.1298.

Naphthalen-2-yl (2*E*,4*E*)-hexa-2,4-dienoate (S11)

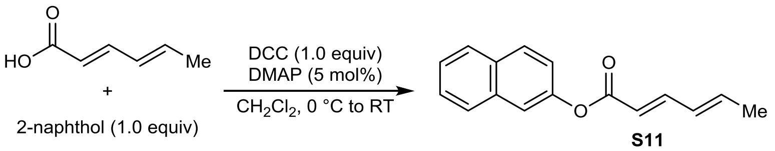

To a solution of sorbic acid (5.60 g, 50.0 mmol), 2-naphthol (7.21 g, 50.0 mmol), and DMAP (305 mg, 2.50 mmol) in CH_2Cl_2 (125 mL) at 0 °C was added DCC (10.3 g, 50.0 mmol) portionwise over 5 min. The mixture was stirred at room temperature for 12 h, filtered through a plug of celite® using CH_2Cl_2 (3 x 35 mL) as the eluent, and concentrated *in vacuo*. Purification of the residue by flash column chromatography (30% EtOAc/petroleum ether) gave *α,β,γ,δ-unsaturated ester* **S12** as a white solid (6.00 g, 50%). R_f 0.60 (30% EtOAc/petroleum ether); m.p. 72–74 °C (Et_2O); IR 3057, 3022, 1725 (C=O), 1647, 1615, 1328, 1126, 999 cm^{-1}; ^1H NMR (400 MHz, $CDCl_3$) δ 7.89–7.79 (3H, m, Ar**H**), 7.61 (1H, d, *J* = 2.2 Hz, Ar**H**), 7.54–7.44 (3H, m, Ar**H** and O=CCH=C**H**), 7.30–7.27 (2H, m, Ar**H**), 6.39–6.20 (2H, m, C**H**=C**H**CH$_3$), 6.03 (1H, d, *J* = 15.5 Hz, O=CC**H**=CH), 1.92 (3H, d, *J* = 5.8 Hz, C**H**$_3$); ^{13}C NMR (100.6 MHz, $CDCl_3$) δ 165.8 (C), 148.5 (C), 147.0 (CH), 140.8 (CH), 133.8 (C), 131.4 (C), 129.8 (CH), 129.3 (CH), 127.7 (CH), 127.6 (CH), 126.5 (CH), 125.6 (CH), 121.3 (CH), 118.5 (CH), 118.0 (CH), 18.8 (CH_3); HRMS (ESI) Exact mass calculated for $C_{16}H_{14}O_2Na$ $[M+Na]^+$: 261.0891, found: 261.0890.

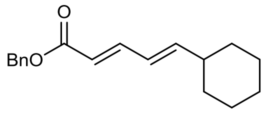

Benzyl (2*E*,4*E*)-5-cyclohexylpenta-2,4-dienoate (S12). The title compound was prepared according to a slight modification of General Procedure A (in that MIDA boronate was used in place of a boronic ester) from alkenyl MIDA boronate **S3** (6.36 g, 24.0 mmol), alkenyl bromide **S7** (4.82 g, 20.0 mmol), $Pd(PPh_3)_4$ (0.69 g, 0.60 mmol), and Na_2CO_3 (4.24 g, 40.0 mmol), and purified by flash column chromatography (0% to 5% Et_2O/cyclohexane) to give a colorless oil (4.93 g, 91%) that displayed spectroscopic data consistent with those reported previously.[4]

3.3 Copper-Catalyzed 1,6-Boration and Conversion of Pinacol Boronic Esters into Potassium Allyltrifluoroborates

General Procedure B

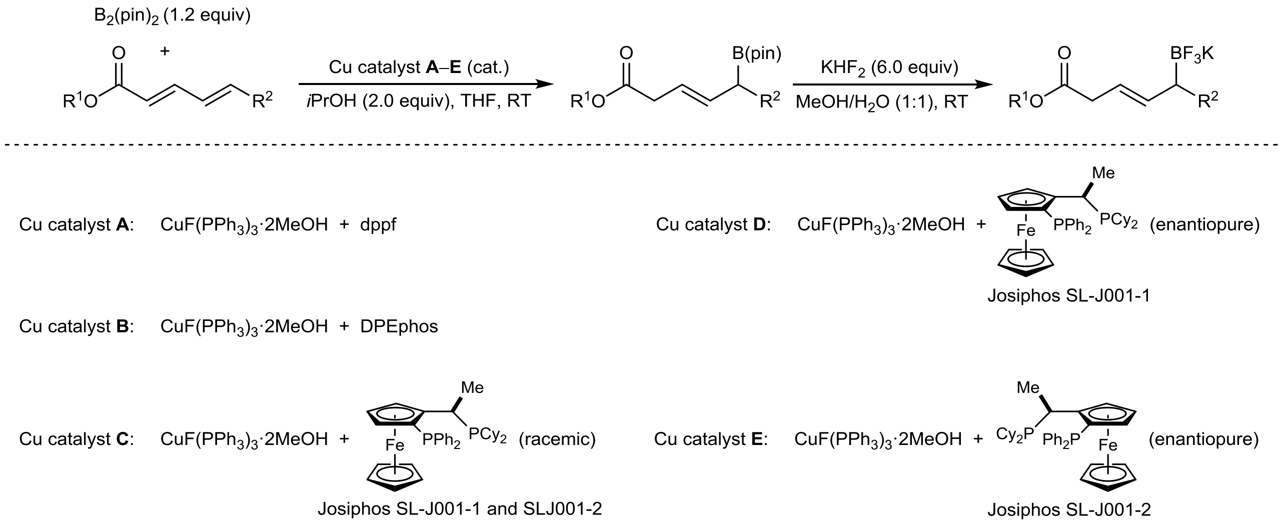

The appropriate α,β,γ,δ-unsaturated ester (1.0 equiv) and $B_2(pin)_2$ (1.2 equiv) were placed in a round bottomed flask and dissolved in THF (8 mL/mmol of α,β,γ,δ-unsaturated ester). *i*-PrOH (2.0 equiv) was added and the mixture was degassed by bubbling N_2 through it for 30 min. One of previously prepared **Catalyst Solutions A**–**E** (see below for their preparation, 1.0 mL/mmol of α,β,γ,δ-unsaturated ester) was added and the reaction was stirred overnight at room temperature. The mixture was diluted with petroleum ether (12 mL/mmol of α,β,γ,δ-unsaturated ester), filtered through a silica plug, eluting with EtOAc, and concentrated *in vacuo*. The residue was dissolved in 1:1 MeOH/H_2O (5 mL/mmol of α,β,γ,δ-unsaturated ester), KHF_2 (6.0 equiv) was added, and the reaction was stirred vigorously overnight at room temperature before being concentrated *in vacuo*. The residue was suspended in boiling acetone, filtered through a cotton wool plug, and the filtrate was concentrated *in vacuo*. The solid obtained was heated to 60–70 °C under reduced pressure for 30 min before triturating with Et_2O to leave the potassium allyltrifluoroborate.

Preparation of Catalyst Solution A: A round bottomed flask containing $CuF(PPh_3)_3{\cdot}2MeOH$ (74.5 mg, 0.075 mmol) and dppf (49.9 mg, 0.090 mmol) was flushed with N_2. Anhydrous and degassed THF (5 mL) was added and the mixture was stirred at 50 °C for 30 min under positive N_2 pressure. Anhydrous and degassed THF (10 mL) was added to give a 5.0 mM solution of catalyst solution.

Preparation of Catalyst Solution B: A round bottomed flask containing $CuF(PPh_3)_3{\cdot}2MeOH$ (74.5 mg, 0.075 mmol), DPEphos (48.7 mg, 0.090 mmol) was flushed with N_2. Anhydrous and degassed THF (5 mL) was added and the mixture was stirred at 50 °C for 30 min under positive N_2 pressure. Anhydrous and degassed THF (10 mL) was added to give a 5.0 mM solution of catalyst solution.

Preparation of Catalyst Solution C: A round bottomed flask containing $CuF(PPh_3)_3{\cdot}2MeOH$ (74.5 mg, 0.075 mmol), Josiphos (SL-J001-1) (28.8 mg, 0.045 mmol) and Josiphos (SL-J001-2) (28.8 mg, 0.045 mmol) was flushed with N_2. Anhydrous and degassed THF (2.5 mL) was added and the mixture was stirred at 50 °C for 30 min under positive N_2 pressure. Anhydrous and degassed THF (5 mL) was added to give a 10.0 mM solution of catalyst solution.

Preparation of Catalyst Solution D: A round bottomed flask containing $CuF(PPh_3)_3{\cdot}2MeOH$ (74.5 mg, 0.075 mmol), Josiphos (SL-J001-1) (57.6 mg, 0.090 mmol) was flushed with N_2. Anhydrous and degassed THF (5 mL) was added and the mixture was stirred at 50 °C for 30 min under positive N_2 pressure. Anhydrous and degassed THF (10 mL) was added to give a 5.0 mM solution of catalyst solution.

Preparation of Catalyst Solution E: A round bottomed flask containing $[CuF(PPh_3)_3{\cdot}2MeOH]$ (24.8 mg, 0.025 mmol), Josiphos (SL-J001-2) (19.2 mg, 0.030 mmol) was flushed with N_2. Anhydrous and degassed THF (5 mL) was added and the mixture was stirred at 50 °C for 30 min under positive N_2 pressure. Anhydrous and degassed THF (3.3 mL) was added to give a 5.0 mM solution of catalyst solution.

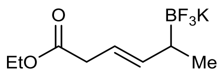

Ethyl (*E*)-5-(trifluoro-λ^4-boranyl)hex-3-enoate potassium (2a). The title compound was prepared according to General Procedure B using ethyl sorbate (**17**, 2.10 g, 15.0 mmol), $B_2(pin)_2$ (4.57 g, 18.0 mmol), and catalyst solution A (15 mL, [Cu] = 0.5 mol%) to give a white solid (1.67 g, 46%). m.p. decomposed at 140 °C (acetone); IR 2967, 1722 (C=O), 1180, 1033, 1007, 970, 914, 806 cm^{-1}; ^1H NMR (400 MHz, CD_3OD) δ 5.80 (1H, ddt, J =

15.3, 7.3, 1.3 Hz, CH=C**H**CH), 5.22 (1H, dtd, *J* = 15.3, 7.0, 1.4 Hz, C**H**=CHCH), 4.14 (2H, q, *J* = 7.1 Hz, $CH_3C\mathbf{H}_2$), 3.00 (2H, ddd, 7.0, 1.1, 1.1 Hz, $C\mathbf{H}_2$CH=CH), 1.32–1.22 (4H, m, C**H**B and $C\mathbf{H}_3CH_2$), 0.93 (3H, d, *J* = 7.1 Hz, $CHC\mathbf{H}_3$); ^{13}C NMR (100.6 MHz, CD_3OD) δ 175.5 (C), 144.9 (CH), 115.6 (CH), 61.6 (CH_2), 39.5 (CH_2), 14.9 (CH_3), 14.5 (CH_3), the carbon (CH) adjacent to boron was not observed due to quadrupolar coupling effects of ^{11}B; ^{19}F NMR (376 MHz, CD_3OD) δ –147.9 to –148.9 (m); HRMS (ESI) Exact mass calculated for $C_8H_{13}BF_3O_2$ $[M–K]^-$: 209.0966, found: 209.0974.

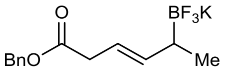

Benzyl (*E*)-5-(trifluoro-λ^4-boranyl)hex-3-enoate potassium (2b). The title compound was prepared according to General Procedure B using benzyl sorbate (**S5**, 1.42 g, 7.00 mmol), $B_2(pin)_2$ (2.13 g, 8.40 mmol), and catalyst solution A (7 mL, [Cu] = 0.5 mol%) to give a white solid (1.31 g, 60%). m.p. decomposed at 150 °C (acetone); IR 2964, 1722 (C=O), 1178, 1009, 986, 747 cm^{-1}; ^{1}H NMR (400 MHz, CD_3OD) δ 7.57–7.15 (5H, m, Ar**H**), 5.87–5.73 (1H, ddt, *J* = 15.3, 7.3, 1.3 Hz, =C**H**CH), 5.22 (1H, dtd, *J* = 15.3, 6.9, 1.3 Hz, C**H**=CHCH), 5.11 (2H, s, $PhC\mathbf{H}_2$), 3.11–2.94 (2H, m, $O{=}CC\mathbf{H}_2$), 1.24 (1H, br s, C**H**B), 0.90 (3H, d, *J* = 7.1 Hz, $C\mathbf{H}_3$); ^{13}C NMR (100.6 MHz, $CDCl_3$) δ 175.1 (C), 145.1 (CH), 137.7 (C), 129.5 (CH), 129.1 (CH), 129.0 (CH), 115.4 (CH), 67.2 (CH_2), 39.6 (CH_2), 14.9 (CH_3), the carbon (CH) adjacent to boron was not observed due to quadrupolar coupling effects of ^{11}B; ^{19}F NMR (376 MHz, CD_3OD) δ –147.9 to –149.0 (m); HRMS (ESI) Exact mass calculated for $C_{13}H_{15}BF_3O_2$ $[M–K]^-$: 271.1123, found: 271.1115.

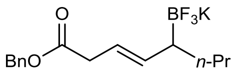

Benzyl (*E*)-5-(trifluoro-λ^4-boranyl)oct-3-enoate potassium (2c). The title compound was prepared according to General Procedure B using α,β,γ,δ-unsaturated ester **18** (230 mg, 1.00 mmol), $B_2(pin)_2$ (605 mg, 1.20 mmol), and catalyst solution B (1 mL, [Cu] = 0.5 mol%) to give a white solid (106 mg, 31%). m.p. decomposed at 195 °C (acetone); IR 2954, 2922, 2857, 1723 (C=O), 1189, 1174, 1060, 974, 928 cm^{-1}; ^{1}H NMR (500 MHz, $(CD_3)_2CO$) δ 7.43–7.26 (5H, m, Ar**H**), 5.61 (1H, ddt, *J* = 15.3, 9.0, 1.3 Hz, =C**H**CH), 5.15 (1H, dtd, *J* = 15.3, 7.0, 0.7 Hz, C**H**=CHCH), 5.11 (2H, s, $PhC\mathbf{H}_2$), 3.01 (2H, dd, *J* = 7.0, 1.3 Hz, $O{=}CC\mathbf{H}_2{=}$), 1.50–1.41 (1H, m, $C\mathbf{H}_aH_bCH_2CH_3$), 1.40–1.29 (1H, m, $C\mathbf{H}_aH_bCH_3$), 1.27–1.09 (3H, m, $C\mathbf{H}CH_a\mathbf{H}_bCH_a\mathbf{H}_bCH_3$), 0.80 (3H, t, *J* = 7.2 Hz, $C\mathbf{H}_3$); ^{13}C NMR (125.8 MHz, $(CD_3)_2CO$) δ 173.9 (C), 145.0 (CH), 137.6 (C), 129.3 (2 x CH), 128.8 (2 x CH), 128.7 (CH), 115.5 (CH), 66.4 (CH_2), 39.1 (CH_2), 33.6 (CH_2), 23.3 (CH_2), 14.9 (CH_3), the carbon (CH) next to boron was not observed due to quadrupolar coupling effects of ^{11}B; ^{19}F NMR (376 MHz, $(CD_3)_2CO$) δ –144.5 to –145.9 (m); HRMS (ESI) Exact mass calculated for $C_{15}H_{19}BF_3O_2$ $[M–K]^-$: 299.1436, found: 299.1435.

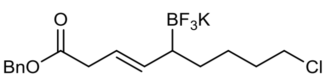

Benzyl (*E*)-9-chloro-5-(trifluoro-λ^4-boranyl)non-3-enoate potassium (2d). The title compound was prepared according to General Procedure B using α,β,γ,δ-unsaturated ester **S8** (1.95 g, 7.00 mmol), $B_2(pin)_2$ (2.13 g, 8.40 mmol), and catalyst solution B (7 mL, [Cu] = 0.5 mol%) to give a white solid (1.38 g, 51%). m.p. 129–130 °C (acetone); IR 3030, 2970, 2941, 2852, 1726 (C=O), 1357, 1228, 1217, 1186, 977 cm^{-1}; ^{1}H NMR (400 MHz, $(CD_3)_2CO$) δ 7.50–7.20 (5H, m, Ar**H**), 5.62 (1H, ddt, *J* = 15.3, 9.0, 1.4 Hz, =C**H**CH), 5.18 (1H, dtd, *J* = 15.1, 6.9, 1.0 Hz, C**H**=CHCH), 5.11 (2H, s, PhC**H**$_2$), 3.52 (2H, t, *J* = 7.0 Hz, C**H**$_2$Cl), 3.02 (2H, dd, *J* = 7.0, 1.3 Hz, O=CC**H**$_2$), 1.82–1.60 (2H, m, C**H**$_2$CH$_2$Cl), 1.60–1.39 (2H, m, C**H**$_2$CH$_2$CH$_2$Cl), 1.38–1.22 (2H, m, CHBC**H**$_2$), 1.11 (1H, m, C**H**B); ^{13}C NMR (100.6 MHz, $(CD_3)_2CO$) δ 173.9 (C), 144.5 (CH), 137.6 (CH), 129.3 (2 x CH), 128.82 (2 x CH), 128.75 (CH), 116.0 (CH), 66.5 (CH$_2$), 46.1 (CH$_2$), 39.0 (CH$_2$), 34.1 (CH$_2$), 30.2 (CH$_2$), 27.5 (CH$_2$), the carbon (CH) adjacent to boron was not observed due to quadrupolar coupling effects of ^{11}B, and the signal at 30.2 ppm which overlaps with the solvents signals, was observed in the DEPT 135 spectrum; ^{19}F NMR (376 MHz, $(CD_3)_2CO$) δ –144.80 to –145.77 (m); HRMS (ESI) Exact mass calculated for $C_{16}H_{20}BClF_3O_2$ [M–K]$^-$: 347.1202, found: 347.1200.

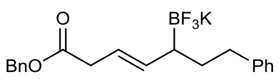

Benzyl (*E*)-7-phenyl-5-(trifluoro-λ^4-boranyl)hept-3-enoate potassium (2e). The title compound was prepared according to General Procedure B using α,β,γ,δ-unsaturated ester **S9** (2.16 g, 8.00 mmol), $B_2(pin)_2$ (2.44 g, 9.60 mmol), and catalyst solution C (8 mL, [Cu] = 1.0 mol%) to give a white solid (793 mg, 25%). m.p. decomposed at 180 °C (acetone); IR 3026, 2970, 2930, 1737 (C=O), 1724 (C=O), 1366, 1228, 1217, 1295, 1178 cm^{-1}; ^{1}H NMR (400 MHz, $(CD_3)_2CO$) δ 7.44–7.23 (5H, m, Ar**H**), 7.23–7.10 (4H, m, Ar**H**), 7.10–7.01 (1H, m, Ar**H**), 5.71 (1H, ddt, *J* = 15.3, 9.0, 1.2 Hz, =C**H**CH), 5.23 (1H, dtd, *J* = 15.3, 6.9, 0.6 Hz, C**H**=CHCH), 5.12 (2H, s, PhC**H**$_2$O), 3.06 (2H, d, *J* = 6.9 Hz, O=CC**H**$_2$), 2.70–2.60 (1H, m, C**H**$_a$H$_b$Ph), 2.51–2.39 (1H, m, CH$_a$**H**$_b$Ph), 1.87–1.77 (1H, m, C**H**$_a$H$_b$CH$_2$Ph), 1.61–1.49 (1H, m, CH$_a$**H**$_b$CH$_2$Ph), 1.19 (1H, br s, C**H**B); ^{13}C NMR (100.6 MHz, $(CD_3)_2CO$) δ 173.8 (C), 145.7 (C), 144.6 (CH), 137.6 (C), 129.30 (2 x CH), 129.28 (2 x CH), 128.8 (2 x CH), 128.74 (2 x CH), 128.72 (CH), 125.7 (CH), 116.1 (CH), 66.4 (CH$_2$), 39.2 (CH$_2$), 36.7 (CH$_2$), 33.6 (CH$_2$), the carbon (CH) adjacent to boron was not observed due to quadrupolar coupling effects of ^{11}B; ^{19}F NMR (376 MHz $(CD_3)_2CO$) δ –144.5 to –145.7 (m); HRMS (ESI) Exact mass calculated for $C_{20}H_{21}BF_3O_2$ [M–K]$^-$: 361.1595, found: 361.1592.

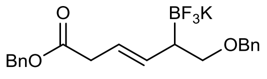

Benzyl (*E*)-6-(benzyloxy)-5-(trifluoro-λ^4-boranyl)hex-3-enoate potassium (2f). The title compound was prepared according to General Procedure B using α,β,γ,δ-unsaturated ester **S10** (1.54 g, 5.00 mmol), $B_2(pin)_2$ (1.52 g, 6.00 mmol), and catalyst

solution C (5 mL, [Cu] = 1.0 mol%) to give a white solid (1.36 g, 66%). m.p. decomposed at 120 °C (acetone); IR 3027, 2970, 2946, 1738 (C=O), 1721 (C=O), 1365, 1217, 1193, 995, 974 cm^{-1}; ^{1}H NMR (400 MHz, $(CD_3)_2CO$) δ 7.41–7.24 (9H, m, Ar**H**), 7.24–7.17 (1H, m, Ar**H**), 5.71 (1H, ddt, *J* = 15.4, 8.3, 1.2 Hz, =C**H**CH), 5.30 (1H, dtd, *J* = 15.4, 7.0, 0.9 Hz, C**H**=CHCH), 5.11 (2H, s, PhC$\mathbf{H}_2$OC=O), 4.45 (1H, d, *J* = 12.3 Hz, $CH_2OC\mathbf{H}_aH_bPh$), 4.35 (1H, d, *J* = 12.3 Hz, $CH_2OCH_a\mathbf{H}_bPh$), 3.66 (1H, dd, *J* = 9.9, 3.7 Hz, $C\mathbf{H}_aH_bOBn$), 3.54 (1H, dd, *J* = 11.1, 9.9 Hz, $CH_a\mathbf{H}_bOBn$), 3.04 (2H, dd, *J* = 7.0, 0.7 Hz, O=CC$\mathbf{H}_2$), 1.72 (1H, br s, C**H**B); ^{13}C NMR (100.6 MHz, $(CD_3)_2CO$) δ 173.9 (C), 142.7 (CH), 141.2 (C), 137.6 (C), 129.3 (2 x CH), 128.8 (4 x CH), 128.7 (CH), 128.2 (2 x CH), 127.6 (CH), 116.4 (CH), 74.2 (CH_2), 72.3 (CH_2), 66.5 (CH_2), 39.2 (CH_2), the carbon (CH) adjacent to boron was not observed due to quadrupolar coupling effects of ^{11}B; ^{19}F NMR (376 MHz $(CD_3)_2CO$) δ –143.4 to –144.9 (m); HRMS (ESI) Exact mass calculated for $C_{20}H_{21}BF_3O_3$ $[M–K]^-$: 377.1541, found: 377.1558.

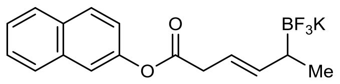

Naphthalen-2-yl (*E*)-5-(trifluoro-λ^4-boranyl)hex-3-enoate potassium (2g). The title compound was prepared according to General Procedure B using α,β,γ,δ-unsaturated ester **S11** (1.19 g, 5.00 mmol), $B_2(pin)_2$ (1.52 g, 6.00 mmol), and catalyst solution C (5 mL, [Cu] = 1.0 mol%) to give a white solid (985 mg, 57%). m.p. decomposed at 140 °C (acetone); IR 3057, 2962, 2868, 1746 (C=O), 1242, 1208, 1145, 1117, 966, 919 cm^{-1}; ^{1}H NMR (400 MHz, $(CD_3)_2CO$) δ 7.97–7.86 (3H, m, Ar**H**), 7.64 (1H, d, *J* = 2.2 Hz, Ar**H**), 7.56–7.45 (2H, m, Ar**H**), 7.30 (1H, dd, *J* = 8.9, 2.3 Hz, Ar**H**), 5.98 (1H, ddt, *J* = 15.4, 7.0, 1.3 Hz, =C**H**CH), 5.25 (1H, dtd, *J* = 15.4, 6.9, 1.5 Hz, C**H**=CHCH), 3.27 (2H, dt, *J* = 6.9, 1.2 Hz, O=CC$\mathbf{H}_2$), 1.24 (1H, br s, C**H**B), 0.93 (3H, d, *J* = 7.0 Hz, C$\mathbf{H}_3$); ^{13}C NMR (100.6 MHz, $(CD_3)_2CO$) δ 172.7 (C), 149.8 (C), 146.7 (CH), 134.8 (C), 132.3 (C), 130.0 (CH), 128.6 (CH), 128.4 (CH), 127.4 (CH), 126.5 (CH), 122.6 (CH), 119.5 (CH), 113.4 (CH), 39.1 (CH_2), 15.2 (CH_3), the carbon (CH) adjacent to boron was not observed due to quadrupolar coupling effects of ^{11}B; ^{19}F NMR (376 MHz $(CD_3)_2CO$) δ –146.1 to –147.5 (m); HRMS (ESI) Exact mass calculated for $C_{16}H_{15}BF_3O_2$ $[M–K]^-$: 307.1133, found: 307.1123.

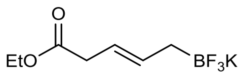

Ethyl (*E*)-5-(trifluoro-λ^4-boranyl)pent-3-enoate potassium (2h).[10] The title compound was prepared according to General Procedure B using α,β,γ,δ-unsaturated ester **S6** (320 mg, 2.50 mmol), $B_2(pin)_2$ (773 mg, 3.00 mmol), and catalyst solution C (1.25 mL, [Cu] = 0.5 mol%) to give a white solid (341 mg, 58%). m.p. decomposed at 175 °C (acetone); IR 2986, 1727 (C=O), 1224, 1184, 955 cm^{-1}; ^{1}H NMR (400 MHz, CD_3CN) δ 5.72–5.59

10. V. J. Olsson, S. Sebelius, N. Selander, K. J. Szabó, *J. Am. Chem. Soc.* **2006**, *128*, 4588–4589.

(1H, m, =C**H**CH_2B), 5.26–5.09 (1H, m, C**H**=CHCH_2B), 4.11 (2H, q, *J* = 7.1 Hz, CH_3C**H**$_2$), 2.98 (2H, dd, *J* = 7.0, 1.1 Hz, O=CC**H**$_2$), 1.25 (3H, t, *J* = 7.1 Hz, C**H**$_3$$CH_2$), 1.05 (2H, br s, C**H**$_2$B); ^{13}C NMR (100.6 MHz, CD_3CN) δ 174.3 (C), 139.03-139.01 (m, CH), 117.7 (CH), 61.1 (CH_2), 38.9 (CH_2), 14.5 (CH_3), the carbon (CH_2) adjacent to boron was not observed due to quadrupolar coupling effects of ^{11}B; ^{19}F NMR (376 MHz, CD_3CN) δ –139.1 to –142.4 (m); HRMS (ESI) Exact mass calculated for $C_7H_{11}BF_3O_2$ [M–K]$^-$: 195.0810, found: 195.0817.

Ethyl (*Z*)-5-(trifluoro-λ^4-boranyl)hex-3-enoate potassium (8)

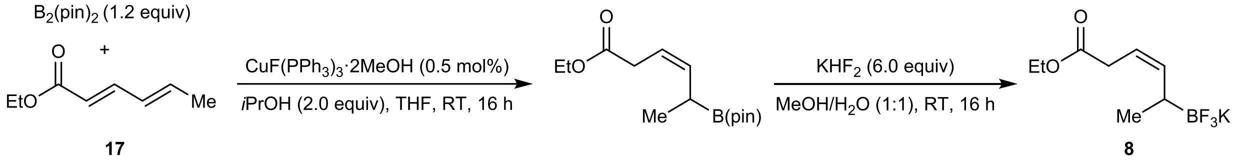

Ethyl sorbate (**17**, 701 mg, 5.00 mmol), B_2(pin)$_2$ (1.52 g, 6.00 mmol), and CuF(PPh_3)$_3$·2MeOH (25 mg, 0.025 mmol, 0.5 mol%) were placed in a 150 mL round bottomed flask, which was flushed with N_2. THF (40 mL) and *i*-PrOH (765 µL, 10.0 mmol) were added and the reaction was stirred for 16 h at room temperature. The mixture was diluted with petroleum ether (60 mL), filtered through a silica plug, eluting with EtOAc, and concentrated *in vacuo*. The residue was dissolved in 1:1 MeOH/H_2O (25 mL), KHF_2 (2.34 g, 30.0 mmol) was added, and the reaction was stirred vigorously for 16 h at room temperature before being concentrated *in vacuo*. The residue was suspended in boiling acetone, filtered through a cotton wool plug, and the filtrate was concentrated *in vacuo*. The solid obtained was heated to 60–70 °C under reduced pressure for 30 min before triturating with Et_2O to give the *allyltrifluoroborate* **8** as a white solid (240 mg, 20%). m.p. decomposed at 130 °C (acetone); IR 2979, 1726 (C=O), 1181, 1003, 964, 915, 760 cm^{-1}; ^{1}H NMR (400 MHz, $(CD_3)_2CO$) δ 5.53 (1H, dddd, *J* = 10.7, 10.7, 1.6, 1.6 Hz, =C**H**CH), 5.14 (1H, dddd, *J* = 10.7, 7.1, 7.1, 0.9 Hz, C**H**=CHCH), 4.07 (2H, q, *J* = 7.1 Hz, CH_3C**H**$_2$), 3.11 (1H, ddd, 17.3, 7.1, 1.6 Hz, O=CC**H**$_a$H$_b$), 3.02 (1H, ddd, 17.3, 7.1, 1.6 Hz, O=CCH$_a$**H**$_b$), 1.43 (1H, br s, C**H**B), 1.20 (3H, t, *J* = 7.1 Hz, C**H**$_3$$CH_2$), 0.86 (3H, d, *J* = 7.0 Hz, CHC**H**$_3$); ^{13}C NMR (100.6 MHz, $(CD_3)_2CO$) δ 174.0 (C), 144.4 (CH), 114.9 (CH), 60.6 (CH_2), 33.6 (CH_2), 16.8 (CH_3), 14.5 (CH_3), the carbon (CH) adjacent to boron was not observed due to quadrupolar coupling effects of ^{11}B; ^{19}F NMR (376 MHz, $(CD_3)_2CO$) δ –145.8 to –147.5 (m); HRMS (ESI) Exact mass calculated for $C_8H_{13}BF_3O_2$ [M–K]$^-$: 209.0966, found: 209.0983.

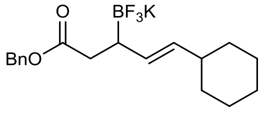

Benzyl (*E*)-5-cyclohexyl-5-(trifluoro-λ^4-boranyl)pent-3-enoate potassium (9). The title compound was prepared according to General Procedure B using α,β,γ,δ-unsaturated ester **S12** (270 mg, 1.00 mmol), B_2(pin)$_2$ (305 mg,

1.20 mmol), and catalyst solution B (1 mL, [Cu] = 1 mol%) to give a white solid (271 mg, 72%); m.p. 130–132 °C (acetone); IR 3005, 2970, 2922, 1737 (C=O), 1722 (C=O), 1452, 1366, 1353, 1217, 968 cm^{-1}; ^{1}H NMR (400 MHz, $(CD_3)_2CO$) δ 7.45–7.23 (5H, m, Ar**H**), 5.49 (1H, ddd, *J* = 15.4, 8.4, 1.1 Hz, CHCH=C**H**), 5.07–4.99 (1H, dd, *J* = 15.4, 6.7 Hz, CHC**H**=CH), 5.01 (2H, s, PhC**H**$_2$O), 2.43 (1H, dd, *J* = 14.0, 4.5 Hz, O=CC**H**$_a$H$_b$), 2.20 (1H, dd, *J* = 14.0, 11.1 Hz, O=CCH$_a$**H**$_b$), 1.86–1.74 (1H, m, CH_2C**H**CH_2), 1.70–1.53 (6H, m, cyclohexyl protons), 1.29–1.06 (3H, m, C**H**B and cyclohexyl protons), 1.06–0.92 (2H, m, cyclohexyl protons); ^{13}C NMR (100.6 MHz, $(CD_3)_2CO$) δ 176.1 (C), 138.4 (C), 134.6 (CH), 131.4 (CH), 129.1 (2 x CH), 128.7 (2 x CH), 128.4 (CH), 65.4 (CH_2), 42.0 (CH), 37.3 (CH_2), 34.6 (2 x CH_2), 27.1 (CH_2), 27.0 (2 x CH_2), the carbon (CH) adjacent to boron was not observed due to quadrupolar coupling effects of ^{11}B; ^{19}F NMR (376 MHz $(CD_3)_2CO$) δ –146.2 to –147.5 (m); HRMS (ESI) Exact mass calculated for $C_{18}H_{23}BF_3O_2$ [M–K]$^-$: 339.1749, found: 339.1748.

Ethyl (*R*,*E*)-5-(trifluoro-λ^4-boranyl)hex-3-enoate potassium (*R*-2a) and ethyl (*R*,*E*)-5-hydroxyhex-3-enoate (S13)

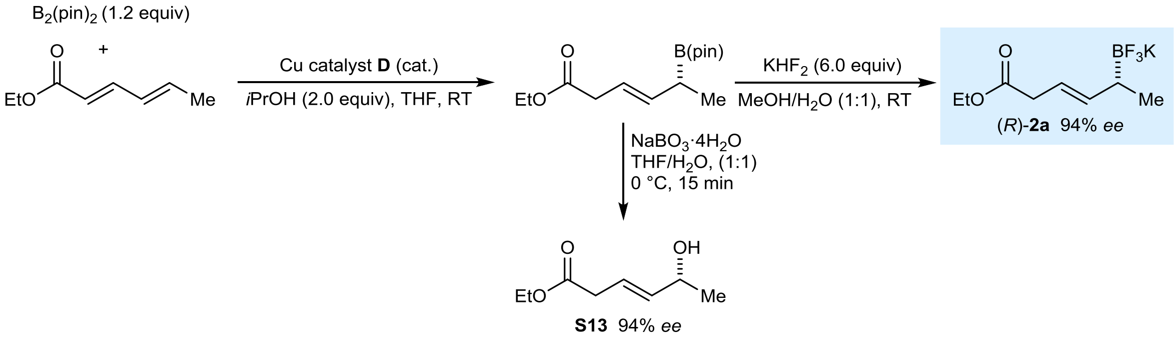

The title compound was prepared according to General Procedure B using ethyl sorbate (2.10 g, 15.0 mmol), $B_2(pin)_2$ (4.57 g, 18.0 mmol), and catalyst solution D (15 mL, [Cu] = 0.5 mol%) to give a white solid (1.90 g, 51%). Spectroscopic data for racemic **2a** are reported on pages 8 and 9. $[\alpha]_D^{20}$ +88.2 (*c* 1.0, MeOH); m.p. decomposed at 140 °C (Et_2O). The enantiomeric excess of (*R*)-**2a** was determined to be 94% ee, as measured on the corresponding alcohol, which was prepared by oxidation of an aliquot (0.5 mL, ~0.06 mmol) of a solution of the preceding pinacol boronic ester *en route* to (*R*)-**2a**, according to the following procedure:

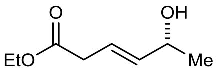

Ethyl (*R*,*E*)-5-hydroxyhex-3-enoate (S13). The aliquot (see above) was poured onto ice (*ca.* 1 g) and $NaBO_3·4H_2O$ (42.7 mg, 0.28 mmol) was added. The mixture was stirred for 15 min at 0 °C, diluted with H_2O (10 mL) and extracted with EtOAc (2 x 10 mL). The combined organic layers were dried (Na_2SO_4) and concentrated *in vacuo*. The *alcohol* **S13** was isolated after preparative TLC purification (20% EtOAc/petroleum ether) as a colorless oil.

R_f = 0.30 (20% EtOAc/petroleum ether); $[\alpha]_D^{20}$ +10.5 (*c* 1.00, $CHCl_3$); IR 3607, 2982, 2929, 1729 (C=O), 1371, 1251, 1177, 972, 826 cm^{-1}; ^{1}H NMR (500 MHz, $CDCl_3$) δ 5.79 (1H, dtd, *J* = 15.3, 6.8, 1.0 Hz, C**H**=CHCH), 5.22 (1H, ddt, *J* = 15.3, 6.3, 1.3 Hz, CH=C**H**CH), 4.38–4.30 (1H, m, C**H**OH), 4.17 (2H, q, *J* = 7.1 Hz, C**H**$_2$CH$_3$), 3.09 (2H, d, 6.9 Hz, C**H**$_2$CH=CH), 1.70, (br s, O**H**), 1.30 (3H, d, *J* = 6.4 Hz, CHC**H**$_3$), 1.29 (3H, d, *J* = 7.1 Hz, C**H**$_3$CH$_2$); ^{13}C NMR (125.8 MHz, $CDCl_3$) δ 171.7 (C), 138.1 (CH), 122.1 (CH), 68.4 (CH), 60.7 (CH$_2$), 37.6 (CH$_2$), 23.1 (CH$_3$), 14.2 (CH$_3$); HRMS (ESI) Exact mass calculated for $C_8H_{14}O_3Na$ $[M+Na]^+$: 181.0841, found: 181.0846. Enantiomeric excess was determined by HPLC with a Chiralpak AS–H column (95:5 *i*-hexane:*i*-PrOH, 1.0 mL/min, 210.4 nm, 25 °C); t_r (minor) = 9.7 min; t_r (major) = 10.8 min, 94% ee.

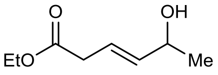

(racemic)

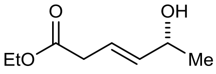

Data file: C:\CHEM32\1\DATA\JIM\DEF_LC 2014-10-24 09-12-01\JIM308 1 ASH 1 95 05.D
Sample name: JIM308 1 ASH 1 95 05
Instrument: AGILENT 1260
Injection date: 10/24/2014 9:24:16 AM
Acq. method: ASH95B05A.35MIN.1.0 ML.10UL.M

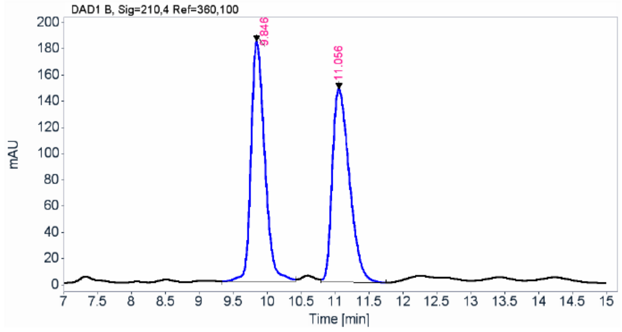

Signal: DAD1 B, Sig=210,4 Ref=360,100

RT [min]	Type	Width [min]	Area	Height	Area%
9.846	BV	0.2188	2589.274	183.2700	50.01
11.056	VB	0.2771	2588.204	147.2011	49.99

Data file: C:\CHEM32\1\DATA\JIM\DEF_LC 2014-10-24 12-47-30\JIM301 2 ASH 1 95 05.D
Sample name: JIM301 2 ASH 1 95 05
Instrument: AGILENT 1260
Injection date: 10/24/2014 12:48:27 PM
Acq. method: ASH95B05A.35MIN.1.0 ML.10UL.M

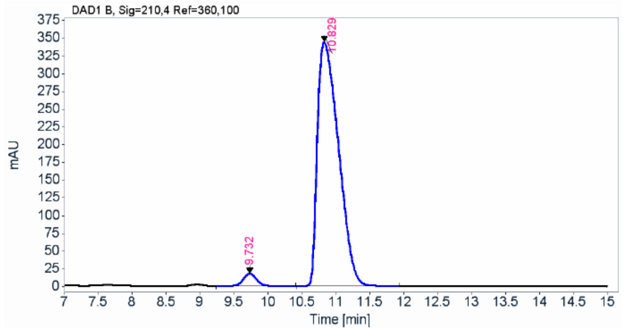

Signal: DAD1 B, Sig=210,4 Ref=360,100

RT [min]	Type	Width [min]	Area	Height	Area%
9.732	BB	0.2110	236.338	17.3396	3.09
10.829	BB	0.3517	7416.668	343.7717	96.91

Ethyl (*S*,*E*)-5-(trifluoro-λ^4-boranyl)hex-3-enoate potassium (*S*-2a) and ethyl (*R*,*E*)-5-hydroxyhex-3-enoate (S14)

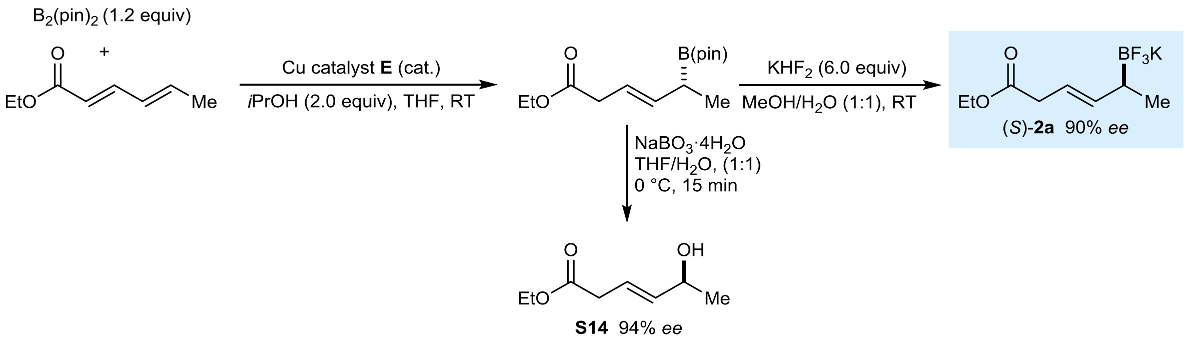

The title compound was prepared according to General Procedure B using ethyl sorbate (701 mg, 5.00 mmol), $B_2(pin)_2$ (1.52 g, 6.00 mmol), and catalyst solution E (3 mL, [Cu] = 0.5 mol%) to give

a white solid (484 mg, 43%). Spectroscopic data for racemic **2a** are reported on pages 8 and 9. $[\alpha]_D^{20}$ –80.4 (*c* 1.0, MeOH); m.p. decomposed at 140 °C (Et_2O). The enantiomeric excess of (*S*)-**2a** was determined to be 94% ee, as measured on the corresponding alcohol, which was prepared by oxidation of an aliquot (0.5 mL, ~0.06 mmol) of a solution of the preceding pinacol boronic ester *en route* to (*S*)-**2a**, according according to the following procedure:

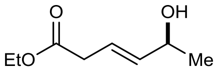

Ethyl (*S*,*E*)-5-hydroxyhex-3-enoate (S14). The aliquot (see above) was poured onto ice (*ca.* 1 g) and $NaBO_3·4H_2O$ (42.7 mg, 0.28 mmol) was added. The mixture was stirred for 15 min at 0 °C, diluted with H_2O (10 mL) and extracted with EtOAc (2 x 10 mL). The combined organic layers were dried (Na_2SO_4) and concentrated *in vacuo*. The *alcohol* **S14** was isolated after preparative TLC purification (20% EtOAc/petroleum ether) as a colorless oil, which displayed spectroscopic data consistent with those reported for (*R*)-**2a** (previous page). (However, there was insufficient material to measure the optical rotation value.) Enantiomeric excess was determined by HPLC with a Chiralpak AS–H column (95:5 *i*-hexane:*i*-PrOH, 1.0 mL/min, 210.4 nm, 25 °C); t_r (major) = 9.7 min; t_r (minor) = 11.0 min, 94% ee.

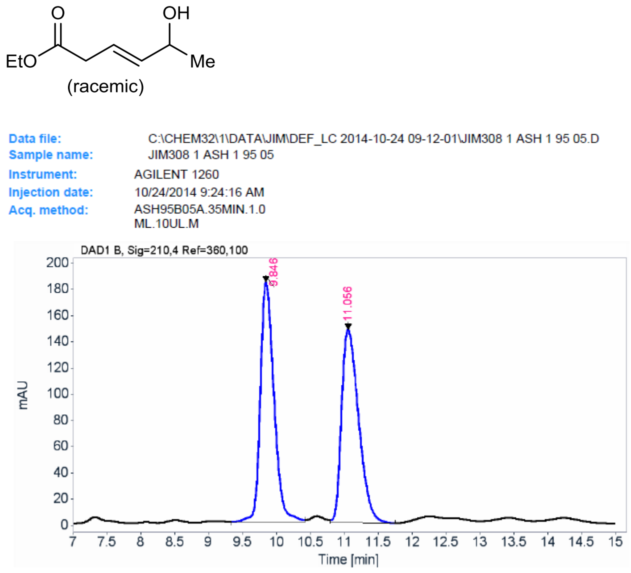

Signal: DAD1 B, Sig=210,4 Ref=360,100

RT [min]	Type	Width [min]	Area	Height	Area%
9.846	BV	0.2188	2589.274	183.2700	50.01
11.056	VB	0.2771	2588.204	147.2011	49.99

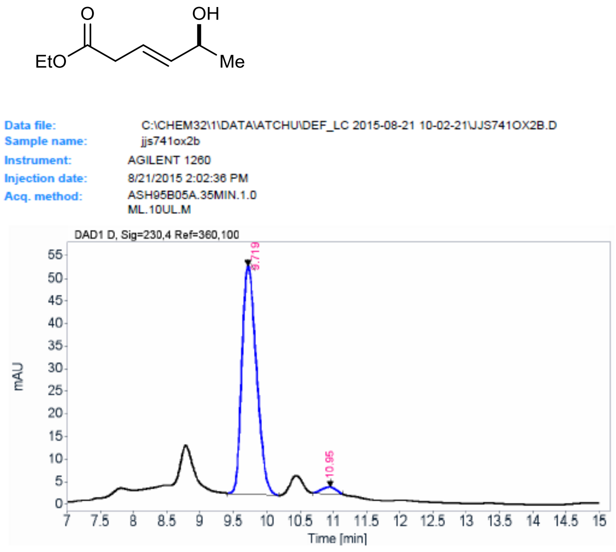

Signal: DAD1 D, Sig=230,4 Ref=360,100

RT [min]	Type	Width [min]	Area	Height	Area%
9.719	MM	0.2543	771.739	50.5789	97.04
10.950	MM	0.2573	23.518	1.5232	2.96

4. Preparation of Homoallylic Boron Reagents

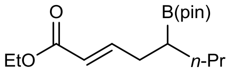

23

Homoallylic boronic ester **23** was prepared according to a previously reported procedure.[11]

Ethyl (*E*)-5-(trifluoro-l4-boranyl)oct-3-enoate potassium (24)

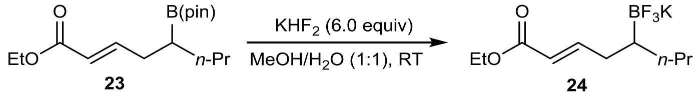

Homoallylic boronic ester **23** (296 mg, 1.00 mmol) was dissolved in 1:1 MeOH/H_2O (4 mL). KHF_2 (469 mg, 6.00 mmol) was added and the reaction was stirred vigorously at room temperature for 15 h before being concentrated *in vacuo*. The residue was suspended in boiling acetone, filtered through a cotton wool plug, and the filtrate was concentrated *in vacuo*. The solid obtained was heated to 60–70 °C under reduced pressure for 30 min before triturating with Et_2O to leave *potassium homoallylic trifluoroborate* **24** as a white solid (219 mg, 79%). m.p. 192–194 °C (acetone); IR 2957, 2925, 2867, 1705 (C=O), 1646, 1212, 1164, 1035, 966, 927 cm^{-1}; ^{1}H NMR (400 MHz, CDO_3D) δ 7.17 (1H, dt, *J* = 15.5, 7.6 Hz, O=CC=C**H**), 5.66 (1H, dt, *J* = 15.5, 1.6 Hz, O=CC**H**=), 4.09 (2H, q, *J* = 7.1 Hz, C$\mathbf{H}_2$O), 2.31–2.19 (1H, m, =CHC$\mathbf{H}_a$$H_b$), 2.11–2.01 (1H, m, =CHC$H_a$$\mathbf{H}_b$), 1.41–1.25 (3H, m, C$\mathbf{H}_a$$H_bC\mathbf{H}_2$$CH_3$), 1.22 (3H, t, *J* = 7.1 Hz, C$\mathbf{H}_3$$CH_2$O), 1.18–1.04 (1H, m, C$H_a$$\mathbf{H}_b$$CH_2CH_3$), 0.80 (3H, t, *J* = 7.0 Hz, CH_2CH_2C$\mathbf{H}_3$), 0.38 (1H, br s, C**H**B); ^{13}C NMR (100.6 MHz, CD_3OD) δ 167.2 (C), 155.8 (CH), 119.8 (CH), 59.9 (CH_2), 35.8 (CH_2), 34.6 (CH_2), 22.8 (CH_2), 15.3 (CH_3), 14.7 (CH_3), the carbon (CH) adjacent to boron was not observed due to quadrupolar coupling effects of ^{11}B; ^{19}F NMR (376.3 MHz, $(CD_3)_2CO$) δ –144.48 to –145.69 (m); HRMS (ESI) Exact mass calculated for $C_{10}H_{17}BF_3O_2$ $[M–K]^-$: 237.1279, found: 237.1292.

5. Racemic Rhodium-Catalyzed Allylations of Cyclic Imines

General Procedure C

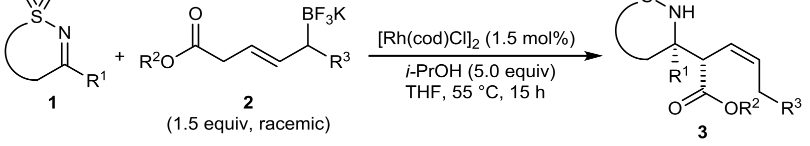

A microwave vial containing the appropriate cyclic imine (0.30 mmol), $[Rh(cod)Cl]_2$ (2.2 mg, 0.0045 mmol, unless otherwise stated), and the appropriate potassium allyltrifluoroborate (0.45

11. I. Ibrahem, P. Breistein, A. Córdova, *Chem. Eur. J.* **2012**, *18*, 5175–5179.

mmol) was flushed with N_2 before anhydrous THF (3 mL) and *i*-PrOH (115 µL, 1.50 mmol) were added. The mixture was heated at 55 °C for 15 h. The reaction was cooled to room temperature, diluted with EtOAc (10 mL), and filtered through a silica plug eluting with EtOAc. The filtrate was concentrated *in vacuo* and the residue was purified by flash column chromatography to give the products.

(±)-Ethyl (2*S*,*Z*)-2-[(4*S*)-6-methyl-2,2-dioxo-3,4-dihydro-1,2λ^6,3-benzoxathiazin-4-yl]hex-3-enoate (3a) and (±)-ethyl (2*S*,*Z*)-4-[(4*S*)-6-methyl-2,2-dioxo-3,4-dihydro-1,2λ^6,3-benzoxathiazin-4-yl]hex-2-enoate (4a)

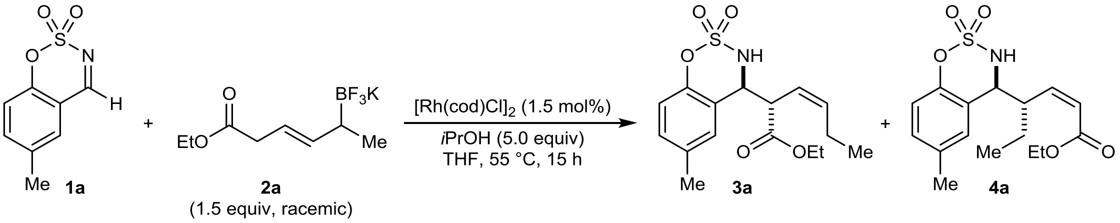

General Procedure C was followed using aldimine **1a** (59 mg, 0.30 mmol) and potassium allyltrifluoroborate **2a** (112 mg, 0.45 mmol), and purified by flash column chromatography (5% EtOAc/petroleum ether) to give *sulfamate* **4a** as a colorless solid (6 mg, 6%) followed by *sulfamate* **3a** as a colorless solid (69 mg, 68%).

Data for **3a**: R_f 0.39 (20% EtOAc/petroleum ether); m.p. 89–91 °C (Et_2O); IR 3222 (NH), 2977, 1702 (C=O), 1433, 1372, 1305, 1178, 1116, 853, 826 cm^{-1}; ^{1}H NMR (400 MHz, $CDCl_3$) δ 7.11 (1H, ddd, *J* = 8.4, 1.4, 0.6 Hz, Ar**H**), 7.00–6.97 (1H, m, Ar**H**), 6.93 (1H, d, *J* = 8.4 Hz, Ar**H**), 5.69 (1H, dtd, *J* = 10.8, 7.5, 0.7 Hz, =C**H**CH_2), 5.56–5.47 (2H, m, CHC**H**= and N**H**), 5.12 (1H, t, *J* = 5.9 Hz, NHC**H**), 4.27–4.13 (2H, m, OC**H**$_2$), 4.06 (1H, ddd, *J* = 9.7, 5.5, 0.8 Hz, O=CC**H**), 2.31 (3H, s, ArC**H**$_3$), 2.16–1.95 (2H, m, =CHC**H**$_2$), 1.24 (3H, t, *J* = 7.1 Hz, OCH_2C**H**$_3$), 0.91 (3H, t, *J* = 7.5 Hz, =CHCH_2C**H**$_3$); ^{13}C NMR (100.6 MHz, $CDCl_3$) δ 171.9 (C), 149.1 (C), 138.7 (CH), 135.2 (C), 130.3 (CH), 126.7 (CH), 120.4 (C), 120.3 (CH), 118.9 (CH), 61.7 (CH_2), 58.2 (CH), 47.6 (CH), 21.3 (CH_2), 20.8 (CH_3), 14.0 (CH_3), 13.5 (CH_3); HRMS (ESI) Exact mass calculated for $C_{16}H_{21}NO_5SNa$ $[M+Na]^+$: 362.1033, found: 362.1030.

Recrystallization of **3a** from Et_2O gave crystals that were suitable for X-ray diffraction:

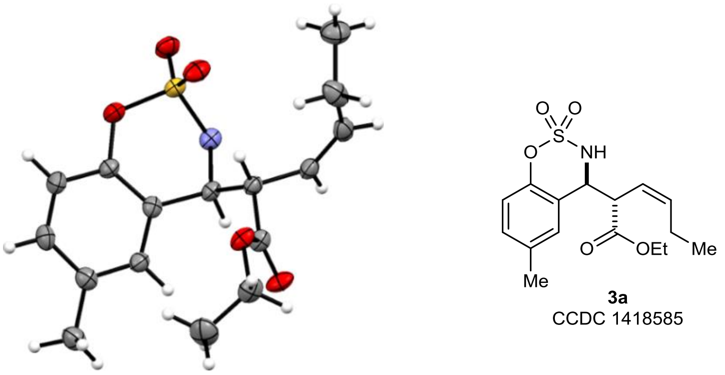

3a
CCDC 1418585

Data for **4a**: R_f 0.42 (20% EtOAc/petroleum ether); m.p. 126–128 °C (Et_2O); IR 3265 (NH), 3974, 2930, 1719 (C=O), 1414, 1361, 1182, 1116, 855, 835 cm^{-1}; ^{1}H NMR (400 MHz, $CDCl_3$) δ 7.15 (1H, s, Ar**H**), 7.09 (1H, d, *J* = 8.3 Hz, Ar**H**), 6.90 (1H, d, *J* = 8,3 Hz, Ar**H**), 5.99–5.87 (2H, m, C**H**=C**H**), 4.91 (1H, dd, *J* = 8.7, 4.6 Hz, NHC**H**), 4.74 (1H, d, *J* = 8.5 Hz, N**H**), 4.44–4.36 (1H, m, C**H**C=), 4.20 (2H, q, *J* = 7.1 Hz, OC**H**$_2$), 2.29 (3H, s, ArC**H**$_3$), 1.82–1.69 (1H, m, =CHC**H**$_a$H$_b$), 1.66–1.57 (1H, m, =CHCH$_a$**H**$_b$), 1.30 (3H, t, *J* = 7.1 Hz, OCH$_2$C**H**$_3$), 1.03 (3H, t, *J* = 7.4 Hz, CHCH$_2$C**H**$_3$); ^{13}C NMR (100.6 MHz, $CDCl_3$) δ 166.2 (C), 145.3 (CH), 142.8 (C), 135.0 (C), 130.1 (CH), 127.1 (CH), 123.7 (CH), 121.3 (C), 118.5 (CH), 60.4 (CH_2), 59.4 (CH), 41.2 (CH), 24.6 (CH_2), 20.8 (CH_3), 14.2 (CH_3), 11.5 (CH_3); HRMS (ESI) Exact mass calculated for $C_{16}H_{21}NO_5SNa$ $[M+Na]^+$: 362.1033, found: 362.1036.

Recrystallization of **4a** from Et_2O gave crystals that were suitable for X-ray diffraction:

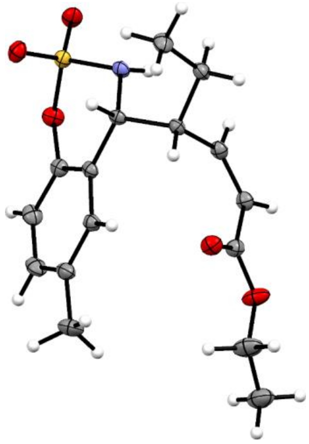

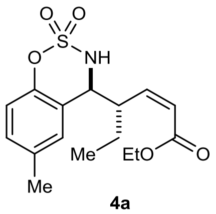

4a
CCDC 1418586

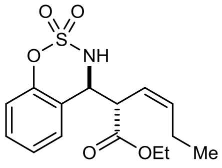

(±)-Ethyl (2*S*,*Z*)-2-[(4*S*)-2,2-dioxo-3,4-dihydro-1,2λ^6,3-benzoxathiazin-4-yl]hex-3-enoate (3b). *Method 1:* The title compound was prepared according to General Procedure C using aldimine **1b** (55 mg, 0.30 mmol) and potassium allyltrifluoroborate **2a** (112 mg, 0.45 mmol) and purified by flash column chromatography (10% EtOAc/petroleum ether) to give a colorless solid (70 mg, 72%). R_f 0.52 (20% EtOAc/petroleum ether); m.p. 89–90 °C (*i*-PrOH/Et_2O, 1:1); IR 3320 (NH), 3011, 2972, 1720 (C=O), 1426, 1373, 1172, 928, 901 cm^{-1}; ^{1}H NMR (400 MHz, $CDCl_3$) δ 7.35–7.29 (1H, m, Ar**H**), 7.22–7.13 (2H, m, Ar**H**), 7.03 (1H, dd, *J* = 8.2, 1.1 Hz, Ar**H**), 5.68 (1H, dtd, *J* = 10.8, 7.4, 0.8 Hz, =C**H**CH$_2$), 5.56–5.46 (2H, m, CHC**H**= and N**H**), 5.15 (1H, dd, *J* = 5.8, 5.8 Hz, NHC**H**), 4.16 (2H, qd, *J* = 7.1, 1.5 Hz, OC**H**$_2$), 4.06 (1H, ddd, *J* = 9.6, 5.8, 0.8 Hz, O=CC**H**), 2.15–1.93 (2H, m, =CHC**H**$_2$), 1.22 (3H, t, *J* = 7.1 Hz, OCH$_2$C**H**$_3$), 0.90 (3H, t, *J* = 7.5 Hz, CHCH$_2$C**H**$_3$); ^{13}C NMR (100.6 MHz, $CDCl_3$) δ 171.8 (C), 151.3 (C), 138.8 (CH), 129.8 (CH), 126.4 (CH), 125.4 (CH), 120.9 (C), 120.3 (CH), 119.2 (CH), 61.7 (CH_2), 58.2 (CH), 47.6 (CH), 21.3 (CH_2), 14.0 (CH_3), 13.6

(CH_3); HRMS (ESI) Exact mass calculated for $C_{15}H_{19}NO_5SNa$ $[M+Na]^+$: 348.0882, found: 348.0885.

Method 2:

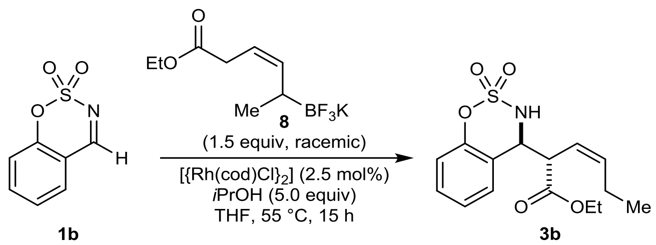

1b **3b**

The title compound was prepared according to General Procedure C using aldimine **1b** (55 mg, 0.30 mmol) and potassium allyltrifluoroborate **8** (112 mg, 0.45 mmol), and purified by flash column chromatography (10% EtOAc/petroleum ether) to give a colorless solid (68 mg, 70%).

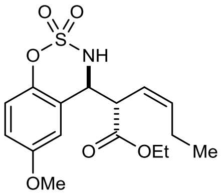

(±)-Ethyl (2*S*,*Z*)-2-[(4*S*)-6-methoxy-2,2-dioxo-3,4-dihydro-1,2λ[6],3-benzoxathiazin-4-yl]hex-3-enoate (3c). The title compound was prepared according to General Procedure C using aldimine **1c** (64 mg, 0.30 mmol) and potassium allyltrifluoroborate **2a** (112 mg, 0.45 mmol) and purified by flash column chromatography (10% EtOAc/petroleum ether) to give a colorless solid (69 mg, 65%). R_f 0.48 (10% EtOAc/petroleum ether); m.p. 71–72 °C (Et_2O); IR 3325 (NH), 3043, 2937, 1720 (C=O), 1495, 1465, 1428, 1172, 1037, 929, 856 cm^{-1}; ^1H NMR (400 MHz, $CDCl_3$) δ 6.96 (1H, d, *J* = 9.0 Hz, Ar**H**), 6.83 (1H, dd, J = 9.0, 2.8 Hz, Ar**H**), 6.69 (1H, d, *J* = 2.8 Hz, Ar**H**), 5.69 (1H, dtd, *J* = 10.8, 7.4, 0.8 Hz, =C**H**CH_2), 5.54–5.46 (2H, m, CHC**H**= and N**H**), 5.09 (1H, dd, *J* = 5.9, 5.9 Hz, NHC**H**), 4.18 (2H, q, *J* = 7.1, OC**H**$_2$), 4.05 (1H, ddd, *J* = 9.6, 5.9, 0.8 Hz, O=CC**H**), 3.75 (3H, s, OC**H**$_3$), 2.16–1.96 (2H, m, =CHC**H**$_2$), 1.23 (3H, t, *J* = 7.1 Hz, OCH_2C**H**$_3$), 0.91 (3H, t, *J* = 7.5 Hz, $CHCH_2C$**H**$_3$); ^{13}C NMR (100.6 MHz, $CDCl_3$) δ 171.8 (C), 156.7 (C), 144.9 (C), 138.7 (CH), 121.7 (C), 120.3 (CH), 119.9 (CH), 115.0 (CH), 111.4 (CH), 61.7 (CH_2), 58.2 (CH), 55.7 (CH_3) 47.7 (CH), 21.3 (CH_2), 14.0 (CH_3), 13.6 (CH_3); HRMS (ESI) Exact mass calculated for $C_{16}H_{21}NO_6SNa$ $[M+Na]^+$: 378.0987, found: 378.0985.

(±)-Ethyl (2*S*,*Z*)-2-[(4*S*)-6-bromo-2,2-dioxo-3,4-dihydro-1,2λ⁶,3-benzoxathiazin-4-yl]hex-3-enoate (3d) and (±)-ethyl (2*S*,*Z*)-4-[(4*S*)-6-bromo-2,2-dioxo-3,4-dihydro-1,2λ⁶,3-benzoxathiazin-4-yl]hex-2-enoate (4d)

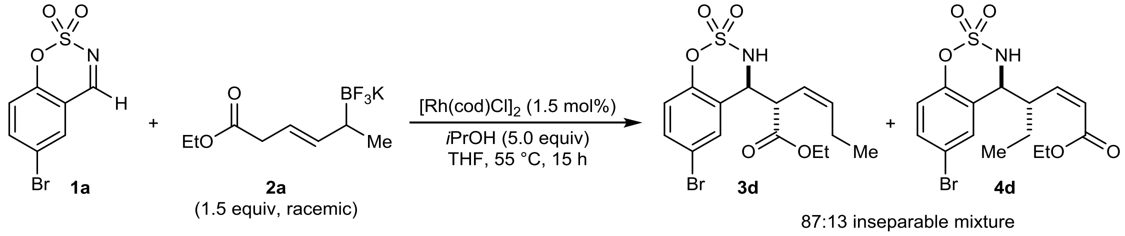

General Procedure C was followed using aldimine **1d** (79 mg, 0.30 mmol) and potassium allyltrifluoroborate **2a** (112 mg, 0.45 mmol), and purified by flash column chromatography (10% EtOAc/petroleum ether) to give an 87:13 inseparable mixture of *sulfamates* **3d** and **3d** as a red oil (69 mg, 65%).

Date for **3d**: R_f 0.71 (20% EtOAc/petroleum ether); IR 3327 (NH), 3011, 2971, 2936, 1719 (C=O), 1475, 1430, 1372, 1191, 1170, 1029, 834, 821 cm^{-1}; ^{1}H NMR (400 MHz, $CDCl_3$), 7.42 (1H, ddd, *J* = 8.7, 2.3, 0.7 Hz, Ar**H**), 7.32 (1H, dd, *J* = 2.3, 0.7 Hz, Ar**H**), 6.91 (1H, d, *J* = 8.7 Hz, Ar**H**), 5.69 (1H, dtd, *J* = 10.8, 7.5, 1.0 Hz, =C**H**CH_2), 5.65 (1H, br s, N**H**), 5.50–5.42 (1H, m,CHC**H**=), 5.12 (1H, d, *J* = 5.8 Hz, NHC**H**), 4.28–4.13 (2H, q, *J* = 7.1 Hz, OC**H**$_2$$CH_3$), 4.06 (1H, ddd, *J* = 9.8, 5.8, 1.0 Hz, O=CC**H**), 2.16–1.97 (2H, m, =CHC**H**$_2$), 1.26 (3H, t, *J* = 7.1 Hz, OCH_2C**H**$_3$), 0.93 (3H, t, *J* = 7.5 Hz, $CHCH_2$C**H**$_3$); ^{13}C NMR (100.6 MHz, $CDCl_3$) δ 171.7 (C), 150.4 (C), 139.1 (CH), 132.7 (CH), 129.4 (CH), 122.8 (C), 120.8 (CH), 119.9 (CH), 118.0 (C), 61.9 (CH_2), 57.8 (CH), 47.4 (CH), 21.3 (CH_2), 14.0 (CH_3), 13.5 (CH_3); HRMS (ESI) Exact mass calculated for $C_{15}H_{18}NO_5SBrNa$ $[M+Na]^+$: 425.9987, found: 425.9982.

Characteristic peaks for **4d**: ^{1}H NMR (400 MHz, $CDCl_3$), δ 7.52 (1H, dd, *J* = 2.3, 0.6 Hz, Ar**H**), 7.37 (1H, ddd, *J* = 8.7, 2.3, 0.6 Hz, Ar**H**), 6.85 (1H, d, *J* = 8.7 Hz, Ar**H**), 5.97–5.86 (2H, m, C**H**=C**H**), 4.91 (1H, d, *J* = 3.6 Hz, NHC**H**), 4.47–4.38 (1H, m, C**H**C=), 1.78–1.53 (2H, m, CHC**H**$_2$), 1.31 (3H, t, *J* = 7.1 Hz, OCH_2C**H**$_3$), 1.02 (3H, t, *J* = 7.4 Hz, $CHCH_2$C**H**$_3$); ^{13}C NMR (100.6 MHz, $CDCl_3$) δ 166.2 (C), 150.4 (C), 144.5 (CH), 132.4 (CH), 129.8 (CH), 124.1 (CH), 123.5 (C), 120.4 (CH), 60.6 (CH_2), 59.1 (CH), 40.7 (CH), 24.5 (CH_2), 14.2 (CH_3), 11.4 (CH_3).

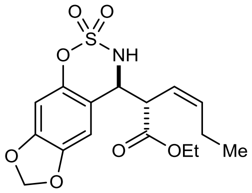

(±)-Ethyl (2*S*,*Z*)-2-[(13*S*)-11,11-dioxo-4,6,10-trioxa-11λ⁶-thia-12-azatricyclo[7.4.0.0³,⁷]trideca-1(9),2,7-trien-13-yl]hex-3-enoate (3e). The title compound was prepared according to General Procedure C using aldimine **1e** (68 mg, 0.30 mmol) and potassium allyltrifluoroborate **2a** (112 mg, 0.45 mmol) and purified by flash column chromatography (5% EtOAc/petroleum ether) to give a yellow oil (71 mg, 65%). R_f 0.26 (20% EtOAc/petroleum ether); IR 3286 (NH), 2980, 2928, 1731

(C=O), 1505, 1433, 1241, 1198, 1133, 1033 cm^{-1}; ^{1}H NMR (400 MHz, $CDCl_3$) δ 6.61 (1H, s, Ar**H**), 6.54 (1H, s, Ar**H**), 5.99 (2H, m, OC**H**$_2$O), 5.71 (1H, dt, *J* = 10.6, 7.4 Hz, =C**H**CH_2), 5.52–5.44 (2H, m, CHC**H**= and N**H**), 5.01 (1H, t, *J* = 6.0 Hz, NHC**H**), 4.25-4.15 (2H, m, OC**H**$_2CH_3$), 3.99 (1H, dd, *J* = 9.6, 5.7 Hz, O=CC**H**), 2.16–1.99 (2H, m, =CHC**H**$_2$), 1.25 (3H, t, *J* = 7.1 Hz, OCH_2C**H**$_3$), 0.95 (3H, t, *J* = 7.5 Hz, CCH_2C**H**$_3$); ^{13}C NMR (100.6 MHz, $CDCl_3$) δ 171.7 (C), 148.2 (C), 145.8 (C), 145.4 (C), 138.8 (CH), 120.3 (CH), 113.3 (C), 105.1 (CH), 102.2 (CH_2), 100.8 (CH), 61.8 (CH_2), 58.1 (CH), 47.8 (CH), 21.3 (CH_2), 14.0 (CH_3), 13.6 (CH_3); HRMS (ESI) Exact mass calculated for $C_{16}H_{19}NO_7SNa$ $[M+Na]^+$: 392.0774, found: 392.0772.

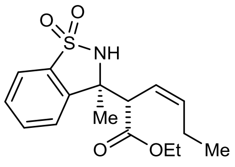

(±)-Ethyl (2*S*,*Z*)-2-[(3*S*)-3-methyl-1,1-dioxo-2,3-dihydro-1λ^6,2-benzothiazol-3-yl]hex-3-enoate (3f). The title compound was prepared according to General Procedure C using ketimine **1f** (54 mg, 0.30 mmol) and potassium allyltrifluoroborate **2a** (112 mg, 0.45 mmol) and purified by flash column chromatography (10% EtOAc/petroleum ether) to give a colorless solid (63 mg, 65%). R_f 0.21 (20% EtOAc/petroleum ether); m.p. 94–96 °C (Et_2O); IR 3368 (NH), 3011, 2969, 2930, 1721 (C=O), 1300, 1173, 971 cm^{-1}; ^{1}H NMR (400 MHz, $CDCl_3$) δ 7.76 (1H, ddd, *J* = 7.6, 1.1, 0.7 Hz, Ar**H**), 7.61 (1H, ddd, *J* = 7.6, 7.6, 1.1 Hz, Ar**H**), 7.52 (1H, ddd, *J* = 7.6, 7.6, 0.7 Hz, Ar**H**), 7.32–7.28 (1H, m, Ar**H**), 5.97 (1H, s, N**H**), 5.45–5.33 (2H, m, C**H**=C**H**), 4.21 (2H, q, *J* = 7.1 Hz, OC**H**$_2CH_3$), 3.86–3.81 (1H, m, C**H**C=), 2.08–1.95 (1H, m, =CHC**H**$_a$H$_b$), 1.93–1.81 (1H, m, =CHCH$_a$**H**$_b$), 1.68 (3H, s, CC**H**$_3$), 1.27 (3H, t, *J* = 7.1 Hz, OCH_2C**H**$_3$), 0.79 (3H, t, *J* = 7.5 Hz, $CHCH_2C$**H**$_3$); ^{13}C NMR (100.6 MHz, $CDCl_3$) δ 172.1 (C), 141.3 (C), 138.0 (CH), 134.8 (C), 133.1 (CH), 129.6 (CH), 122.7 (CH), 121.5 (CH), 120.4 (CH), 64.0 (C), 61.5 (CH_2), 52.2 (CH), 27.6 (CH_3), 21.2 (CH_2), 14.1 (CH_3), 13.6 (CH_3); HRMS (ESI) Exact mass calculated for $C_{16}H_{21}NO_4SNa$ $[M+Na]^+$: 346.1089, found: 346.1089.

Recrystallization of **3f** from CH_2Cl_2 gave crystals that were suitable for X-ray diffraction:

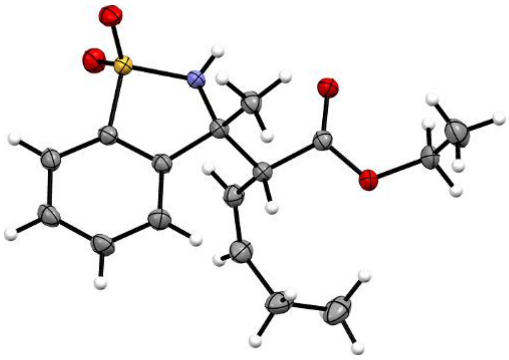

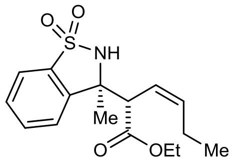

3f
CCDC 1418587

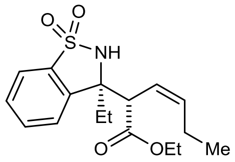

(±)-Ethyl (2*S*,*Z*)-2-[(3*S*)-3-ethyl-1,1-dioxo-2,3-dihydro-1λ^6,2-benzothiazol-3-yl]hex-3-enoate (3g). The title compound was prepared according to General Procedure C using ketimine **1g** (59 mg, 0.30 mmol) and potassium allyltrifluoroborate **2a** (112 mg, 0.45 mmol) and purified by flash column chromatography (10% EtOAc/petroleum ether) to give a colorless solid (54 mg, 53%). R_f 0.28 (20% EtOAc/petroleum ether); m.p. 74–75 °C (Et_2O); IR 3364 (NH), 3011, 2974, 2936, 1719 (C=O), 1730, 1293, 1168, 1161, 1001, 907 cm^{-1}; ^{1}H NMR (400 MHz, $CDCl_3$) δ 7.78–7.74 (1H, m, Ar**H**), 7.61 (1H, ddd, *J* = 7.6, 7.6, 1.2 Hz, Ar**H**), 7.52 (1H, ddd, *J* = 7.6, 7.6, 1.0 Hz, Ar**H**), 7.27–7.23 (1H, m, Ar**H**), 6.10 (1H, s, N**H**), 5.44–5.27 (2H, m, C**H**=C**H**), 4.21 (2H, q, *J* = 7.1 Hz, OC**H**$_2$CH$_3$), 3.89–3.75 (1H, m, C**H**C=), 2.05–1.88 (3H, m, =CHC**H**$_a$H$_b$ and CC**H**$_2$CH$_3$), 1.88–1.72 (1H, m, =CHCH$_a$**H**$_b$), 1.28 (3H, t, *J* = 7.1 Hz, OCH$_2$C**H**$_3$), 0.76 (3H, t, *J* = 7.5 Hz, CCH$_2$C**H**$_3$), 0.71 (3H, t, *J* = 7.3 Hz, CHCH$_2$C**H**$_3$); ^{13}C NMR (100.6 MHz, $CDCl_3$) δ 172.4 (C), 139.1 (C), 137.8 (CH), 136.0 (C), 133.0 (CH), 129.6 (CH), 122.5 (CH), 121.5 (CH), 120.3 (CH), 68.3 (C), 61.6 (CH$_2$), 52.1 (CH), 32.3 (CH$_2$), 21.1 (CH$_2$), 14.1 (CH$_3$), 13.6 (CH$_3$), 7.8 (CH$_3$); HRMS (ESI) Exact mass calculated for $C_{17}H_{23}NO_4SNa$ [M+Na]$^+$: 360.1245, found: 360.1246.

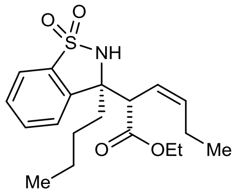

(±)-Ethyl (2*S*,*Z*)-2-[(3*S*)-3-butyl-1,1-dioxo-2,3-dihydro-1λ^6,2-benzothiazol-3-yl]hex-3-enoate (3h). The title compound was prepared according to General Procedure C using ketimine **1h** (67 mg, 0.30 mmol) and potassium allyltrifluoroborate **2a** (112 mg, 0.45 mmol) and purified by flash column chromatography (10% EtOAc/petroleum ether) to give a colorless solid (58 mg, 54%) as a colorless solid. R_f 0.38 (20% EtOAc/petroleum ether); m.p. 135–136 °C (Et_2O); IR 3364 (NH), 3069, 2964, 2932, 1719 (C=O), 1295, 1167, 1161, 1134, 928 cm^{-1}; ^{1}H NMR (400 MHz, $CDCl_3$) δ 7.77–7.73 (1H, m, Ar**H**), 7.61 (1H, ddd, *J* = 7.6, 7.6, 1.2 Hz, Ar**H**), 7.52 (1H, ddd, *J* = 7.6, 7.6, 0.9 Hz, Ar**H**), 7.27–7.23 (1H, m, Ar**H**), 6.11 (1H, s, N**H**), 5.41–5.28 (2H, m, C**H**=C**H**), 4.22 (2H, q, *J* = 7.1 Hz, OC**H**$_2$CH$_3$), 3.87–3.79 (1H, m, O=CC**H**), 2.05–1.73 (4H, m, =CHC**H**$_2$ and CC**H**$_2$), 1.48–1.35 (1H, m, CCH$_2$C**H**$_a$H$_b$), 1.29 (3H, t, *J* = 7.1 Hz, OCH$_2$C**H**$_3$), 1.26–1.09 (3H, m, CCH$_2$CH$_a$**H**$_b$ and CCH$_2$CH$_2$C**H**$_2$), 0.80–0.73 (6H, m, CH$_2$CH$_2$C**H**$_3$ and =CHCH$_2$C**H**$_3$); ^{13}C NMR (100.6 MHz, $CDCl_3$) δ 172.4 (C), 139.5 (C), 137.8 (CH), 135.8 (C), 133.0 (CH), 129.6 (CH), 122.5 (CH), 121.5 (CH), 120.2 (CH), 67.7 (C), 61.6 (CH$_2$), 52.3 (CH), 39.1 (CH$_2$), 25.3 (CH$_2$), 22.4 (CH$_2$), 21.1 (CH$_2$), 14.1 (CH$_3$), 13.7 (CH$_3$), 13.6 (CH$_3$); HRMS (ESI) Exact mass calculated for $C_{19}H_{27}NO_4SNa$ [M+Na]$^+$: 388.1558, found: 388.1564.

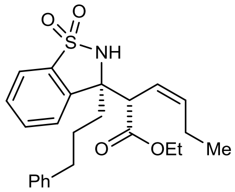

(±)-Ethyl (2*S*,*Z*)-2-[(3*S*)-1,1-dioxo-3-(3-phenylpropyl)-2,3-dihydro-1λ^6,2-benzothiazol-3-yl]hex-3-enoate (3i). The title compound was prepared according to General Procedure C using ketimine **1i** (86 mg, 0.30 mmol) and potassium allyltrifluoroborate **2a** (112 mg, 0.45 mmol) and purified by flash column chromatography (10% EtOAc/petroleum ether) to give a colorless oil (70 mg, 55%). R_f 0.31 (20% EtOAc/petroleum ether); IR 3362 (NH), 3063, 3011, 2936, 1718 (C=O), 1454, 1297, 1172, 1161, 1133, 1132, 1026, 924 cm^{-1}; ^{1}H NMR (400 MHz, $CDCl_3$) δ 7.80–7.70 (1H, m, Ar**H**), 7.61 (1H, ddd, *J* = 7.6, 7.6, 1.3 Hz, Ar**H**), 7.57 (1H, ddd, *J* = 7.6, 7.6, 1.0 Hz, Ar**H**), 7.25–7.18 (2H, m, Ar**H**), 7.18–7.11 (2H, m, Ar**H**), 7.06–7.00 (2H, m, Ar**H**), 6.11 (1H, s, N**H**), 5.39–5.36 (2H, m, C**H**=C**H**), 4.18 (2H, qd, *J* = 7.1, 1.6 Hz, OC**H**$_2$CH$_3$), 3.82–3.74 (1H, m, O=CC**H**), 2.63–2.54 (1H, m, C**H**$_a$H$_b$Ph), 2.52–2.38 (1H, m, CH$_a$**H**$_b$Ph), 2.05–1.88 (5H, m, C=CHC**H**$_2$ and C**H**$_a$H$_b$CH$_2$Ph and C**H**$_2$CH$_2$CH$_2$Ph), 1.25 (3H, t, *J* = 7.1 Hz, OCH$_2$C**H**$_3$), 1.15–1.01 (1H, m, CH$_a$**H**$_b$CH$_2$Ph), 0.74 (3H, t, *J* = 7.5 Hz, CHCH$_2$C**H**$_3$); ^{13}C NMR (100.6 MHz, $CDCl_3$) δ 172.3 (C), 141.3 (C), 139.3 (C), 137.9 (CH), 135.8 (C), 133.1 (CH), 129.7 (CH), 128.33 (2 x CH), 128.27 (2 x CH), 125.9 (CH), 122.5 (CH), 120.0 (CH), 67.6 (C), 61.6 (CH_2), 52.2 (CH), 38.5 (CH_2), 35.2 (CH_2), 24.5 (CH_2), 21.1 (CH_2) 14.0 (CH_3), 13.6 (CH_3); HRMS (ESI) Exact mass calculated for $C_{24}H_{29}NO_4SNa$ [M+Na]$^+$: 450.1715, found: 450.1726.

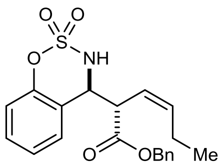

(±)-Benzyl (2*S*,*Z*)-2-[(4*S*)-2,2-dioxo-3,4-dihydro-1,2λ^6,3-benzoxathiazin-4-yl]hex-3-enoate (3k). The title compound was prepared according to General Procedure C using aldimine **1b** (55 mg, 0.30 mmol) and potassium allyltrifluoroborate **2b** (140 mg, 0.45 mmol) and purified by flash column chromatography (10% EtOAc/petroleum ether) to give a colorless solid (80 mg, 69%). R_f 0.52 (20% EtOAc/petroleum ether); m.p. 106–108 °C (Et_2O); IR 3322 (NH), 3068, 3043, 2969, 1724 (C=O), 1487, 1377, 1192, 929, 824 cm^{-1}; ^{1}H NMR (400 MHz, $CDCl_3$) δ 7.44–7.36 (3H, m, Ar**H**), 7.36–7.27 (3H, m, Ar**H**), 7.17–7.07 (2H, m, Ar**H**), 7.05 (1H, dd, *J* = 8.2, 0.9 Hz, Ar**H**), 5.73 (1H, dtd, *J* = 10.7, 7.4, 0.7 Hz, =C**H**CH$_2$), 5.59–5.49 (2H, m, C**H**=CHCH$_2$ and N**H**), 5.24–5.13 (3H, m, C**H**$_2$Ph and NHC**H**), 4.17 (1H, ddd, *J* = 9.6, 5.9, 0.8 Hz, O=CC**H**), 2.15–1.94 (2H, m, =CHC**H**$_2$), 0.93 (3H, t, *J* = 7.5 Hz, CHCH$_2$C**H**$_3$); ^{13}C NMR (100.6 MHz, $CDCl_3$) δ 171.6 (C), 151.2 (C), 138.9 (CH), 134.9 (C), 129.8 (CH), 128.62 (2 x CH), 128.55 (CH), 128.3 (2 x CH), 126.4 (CH), 125.4 (CH), 120.8 (C), 120.3 (CH), 119.1 (CH), 67.4 (CH_2), 58.1 (CH), 47.8 (CH), 21.3 (CH_2), 13.5 (CH_3); HRMS (ESI) Exact mass calculated for $C_{20}H_{21}NO_5SNa$ [M+Na]$^+$: 410.1038, found: 410.1033.

(±)-Benzyl (2*S*,*Z*)-2-[(4*S*)-2,2-dioxo-3,4-dihydro-1,2λ⁶,3-benzoxathiazin-4-yl]oct-3-enoate (3l)

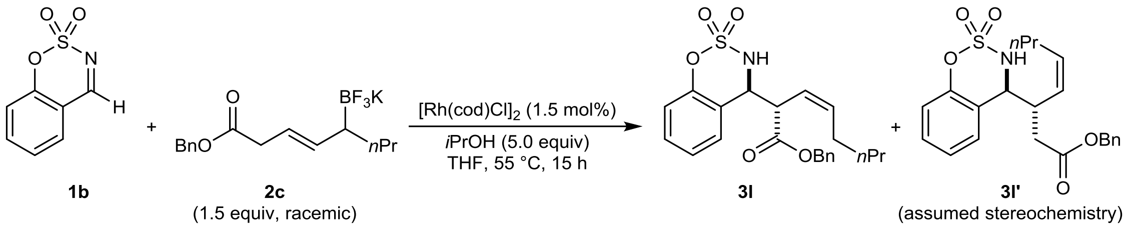

The title compound was prepared according to General Procedure C using aldimine **1b** (55 mg, 0.30 mmol) and potassium allyltrifluoroborate **2c** (152 mg, 0.45 mmol) and purified by flash column chromatography (5% EtOAc/petroleum ether) to give a 95:5 inseparable mixture of **3l** and the product **3l'** resulting from allylation without allylrhodium chain walking as a colorless solid (88 mg, 70%).

Data for **3l**: R_f 0.45 (20% EtOAc/petroleum ether); m.p. 70–72 °C (Et_2O); IR 3280 (NH), 2952, 2932, 1700 (C=O), 1492, 1455, 1366, 1253, 1200, 1167 cm^{-1}; ^1H NMR (400 MHz, $CDCl_3$) δ 7.41–7.33 (3H, m, Ar**H**), 7.32–7.26 (3H, m, Ar**H**), 7.14–7.05 (2H, m, Ar**H**), 7.03 (1H, d, *J* = 8.2 Hz, Ar**H**), 5.71 (1H, dt, *J* = 10.8, 7.4 Hz, =C**H**CH_2), 5.55 (1H, t, *J* = 10.2 Hz, CHC**H**=), 5.50 (1H, d, *J* = 6.5 Hz, N**H**), 5.22–5.10 (3H, m, C**H**$_2$Ph and NHC**H**), 4.14 (1H, dd, *J* = 9.6, 5.8 Hz, O=CC**H**), 2.12–1.94 (2H, m, =CHC**H**$_2$), 1.30–1.19 (4H, m, C**H**$_2$C**H**$_2$$CH_3$), 0.85 (3H, t, *J* = 7.0 Hz, C**H**$_3$); ^{13}C NMR (100.6 MHz, $CDCl_3$) δ 171.6 (C), 151.2 (C), 137.6 (CH), 134.9 (C), 129.8 (CH), 128.64 (2 x CH), 128.58 (CH), 128.4 (2 x CH), 126.4 (CH), 125.4 (CH), 120.8 (C), 120.7 (CH), 119.2 (CH), 67.4 (CH_2), 58.1 (CH), 47.8 (CH), 31.1 (CH_2), 27.7 (CH_2), 22.3 (CH_2), 13.9 (CH_3); HRMS (ESI) Exact mass calculated for $C_{22}H_{25}NO_5SNa$ $[M+Na]^+$: 438.1346, found: 438.1333.

Characteristic peaks for **3l'**: ^1H NMR (400 MHz, $CDCl_3$) δ 5.04–4.92 (2H, m, N**HCH**), 3.86–3.76 (1H, m, C**H**C=), 2.78 (1H, dd, *J* = 16.3, 7.8 Hz, O=CC**H**$_a$H$_b$), 2.57 (1H, dd, *J* = 16.3, 6.2 Hz, O=CCH$_a$**H**$_b$).

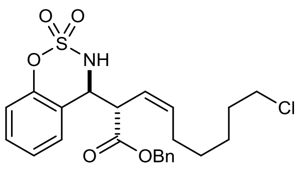

(±)-Benzyl (2*S*,*Z*)-9-chloro-2-[(4*S*)-2,2-dioxo-3,4-dihydro-1,2λ⁶,3-benzoxathiazin-4-yl]non-3-enoate (3m). The title compound was prepared according to General Procedure C using aldimine **1b** (55 mg, 0.30 mmol) and potassium allyltrifluoroborate **2d** (174 mg, 0.45 mmol) and purified by flash column chromatography (10% to 30% EtOAc/petroleum ether then a second purification using 75% to 100% CH_2Cl_2/petroleum ether) to give a colorless solid (87 mg, 63%). R_f 0.35 (20% EtOAc/petroleum ether); m.p. 83–85 °C (Et_2O); IR 3184 (NH), 2934, 2868, 1709 (C=O), 1436, 1380, 1304, 1203, 1186, 1167 cm^{-1}; ^1H NMR (400 MHz, $CDCl_3$) δ 7.41–7.34 (3H, m, Ar**H**), 7.34–7.28 (3H, m, Ar**H**), 7.14–7.00 (3H, m, Ar**H**), 5.71 (1H, dtd, *J* = 10.8, 7.3, 1.0 Hz, =C**H**CH_2), 5.57 (1H, ddt, *J* = 10.8, 9.6, 1.6 Hz, CHC**H**=), 5.44 (1H, br s, N**H**), 5.22–5.11 (3H, m, C**H**$_2$Ph and

NHC**H**), 4.13 (1H, ddd, *J* = 9.6, 6.0, 1.0 Hz, O=CC**H**), 3.48 (2H, t, *J* = 6.7 Hz, C**H**$_2$Cl), 2.15–1.93 (2H, m, =CHC**H**$_2$), 1.70 (2H, m, C**H**$_2$CH$_2$Cl), 1.42–1.20 (4H, m, =CCH$_2$C**H**$_2$C**H**$_2$); ^{13}C NMR (100.6 MHz, CDCl$_3$) δ 171.6 (C), 151.2 (C), 137.0 (CH), 134.9 (C), 129.8 (CH), 128.7 (2 x CH), 128.6 (CH), 128.4 (2 x CH), 126.4 (CH), 125.4 (CH), 121.3 (CH), 120.7 (C), 119.2 (CH), 67.5 (CH$_2$), 58.1 (CH), 48.0 (CH), 44.9 (CH$_2$), 32.3 (CH$_2$), 28.3 (CH$_2$), 27.8 (CH$_2$), 26.5 (CH$_2$); HRMS (ESI) Exact mass calculated for $C_{23}H_{26}ClNO_5SNa$ [M+Na]$^+$: 486.1112, found: 486.1118.

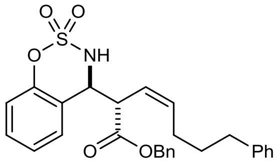

(±)-Benzyl (2*S*,*Z*)-2-[(4*S*)-2,2-dioxo-3,4-dihydro-1,2λ^6,3-benzoxathiazin-4-yl]-7-phenylhept-3-enoate (3n). The title compound was prepared following a slight modification of General Procedure C (in the catalyst loading used) using aldimine **1b** (55 mg, 0.30 mmol), potassium allyltrifluoroborate **2e** (180 mg, 0.45 mmol), and 2.5 mol% of [Rh(cod)Cl]$_2$ (3.7 mg, 0.0075 mmol), and purified by flash column chromatography (0% to 30% EtOAc/petroleum ether, then a second purification using 50% to 100% CH_2Cl_2/petroleum ether) to give a colorless solid (52 mg, 36%). R$_f$ 0.35 (20% EtOAc/petroleum ether); m.p. 93–96 °C (Et$_2$O); IR 3188 (NH), 3022, 2962, 2932, 2864, 1699 (C=O), 1384, 1305, 1186, 1168 cm^{-1}; ^{1}H NMR (400 MHz, CDCl$_3$) δ 7.37–7.33 (3H, m, Ar**H**), 7.32–7.24 (5H, m, Ar**H**), 7.21–7.15 (1H, m, Ar**H**), 7.14–7.09 (2H, m, Ar**H**), 7.09–7.00 (3H, m, Ar**H**), 5.74 (1H, dtd, *J* = 10.8, 7.3, 0.7 Hz, =C**H**CH$_2$), 5.58 (1H, ddt, *J* = 10.8, 9.6, 1.5 Hz, C**H**=CHCH$_2$), 5.42 (1H, d, *J* = 6.5 Hz, N**H**), 5.20–5.10 (3H, m, OC**H**$_2$Ph and NHC**H**), 4.07 (1H, ddd, *J* = 9.6, 5.6, 0.8 Hz, O=CC**H**), 2.54 (2H, app t, *J* = 7.5 Hz, CH$_2$C**H**$_2$Ph), 2.14–1.96 (2H, m, =CHC**H**$_2$), 1.67–1.53 (2H, m, C**H**$_2$CH$_2$Ph); ^{13}C NMR (100.6 MHz, CDCl$_3$) δ 171.5 (C), 151.2 (C), 141.9 (C), 137.0 (CH), 134.9 (C), 129.8 (CH), 128.7 (2 x CH), 128.6 (CH), 128.44 (2 x CH), 128.35 (2 x CH), 128.3 (2 x CH), 126.4 (CH), 125.8 (CH), 125.4 (CH), 121.2 (CH), 120.7 (C), 119.2 (CH), 67.5 (CH$_2$), 58.1 (CH), 47.8 (CH), 35.4 (CH$_2$), 30.6 (CH$_2$), 27.5 (CH$_2$); HRMS (ESI) Exact mass calculated for $C_{27}H_{27}NO_5SNa$ [M+Na]$^+$: 500.1502, found: 500.1504.

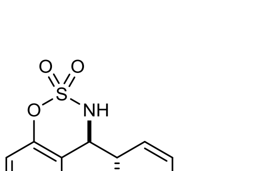

(±)-Benzyl (2*S*,*Z*)-6-(benzyloxy)-2-[(4*S*)-2,2-dioxo-3,4-dihydro-1,2λ^6,3-benzoxathiazin-4-yl]hex-3-enoate (3o). The title compound was prepared following a slight modification of General Procedure C (in the catalyst loading used) using aldimine **1b** (55 mg, 0.30 mmol), potassium allyltrifluoroborate **2g** (187 mg, 0.45 mmol), and 2.5 mol% of [Rh(cod)Cl]$_2$ (3.7 mg, 0.0075 mmol), giving an NMR yield of 53% (using 1,3,5-trimethoxybenzene as in internal standard). A small amount of **3o** for characterization purposes was isolated after iterative purification by preparative TLC (40% Et$_2$O/petroleum ether) as a colorless oil. R$_f$ 0.34 (20% EtOAc/petroleum ether); IR 3263 (NH), 3033, 2923, 2857, 1731 (C=O), 1454, 1377, 1209, 1172, 1105 cm^{-1}; ^{1}H NMR (500 MHz,

$CDCl_3$) δ 7.39–7.33 (5H, m, Ar**H**), 7.30–7.25 (4H, m, Ar**H**), 7.23–7.19 (2H, m, Ar**H**), 7.00–6.95 (3H, m, Ar**H**), 6.16 (1H, d, *J* = 8.0 Hz, N**H**), 5.83–5.77 (1H, m, =C**H**CH_2), 5.67 (1H, ddt, *J* = 11.9, 10.7, 1.3 Hz, C**H**=CHCH_2), 5.28 (1H, d, *J* = 12.1 Hz, O=COC$\mathbf{H}_a$$H_b$Ph), 5.28 (1H, app t, *J* = 7.8 Hz, NHC**H**), 5.15 (1H, d, *J* = 12.1 Hz, O=COC$H_a$$\mathbf{H}_b$Ph), 4.56 (1H, d, *J* = 12.5 Hz, CH_2OC$\mathbf{H}_a$$H_b$Ph), 4.47 (1H, d, *J* = 12.5 Hz, CH_2OC$H_a$$\mathbf{H}_b$Ph), 4.06 (1H, ddd, *J* = 10.3, 8.2, 0.7 Hz, O=CC**H**), 3.45–3.35 (2H, m, C$\mathbf{H}_2$OBn), 2.57–2.47 (1H, m, =CHC$\mathbf{H}_a$$H_b$), 2.23–2.16 (1H, m, =CHC$H_a$$\mathbf{H}_b$); ^{13}C NMR (125.8 MHz, $CDCl_3$) δ 171.4 (C), 151.2 (C), 137.5 (C), 135.0 (C), 132.9 (CH), 129.6 (CH), 128.7 (2 x CH), 128.64 (CH), 128.59 (2 x CH), 128.4 (2 x CH), 128.0 (2 x CH), 127.8 (CH), 126.0 (CH), 125.3 (CH), 124.8 (CH), 121.8 (C), 119.1 (CH), 77.3 (CH_2), 68.1 (CH_2), 67.5 (CH_2), 56.9 (CH), 48.8 (CH), 28.3 (CH_2); HRMS (ESI) Exact mass calculated for $C_{27}H_{27}NO_6SNa$ $[M+Na]^+$: 516.1451, found: 516.1456.

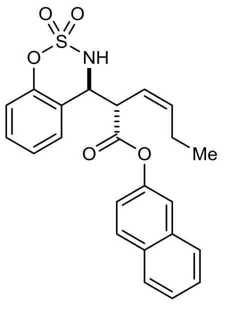

(±)-Naphthalen-2-yl (2*S*,*Z*)-2-[(4*S*)-2,2-dioxo-3,4-dihydro-1,2λ^6,3-benzoxathiazin-4-yl]hex-3-enoate (3p). The title compound was prepared following a slight modification of General Procedure C (in the catalyst loading used) using aldimine **1b** (55 mg, 0.30 mmol), potassium allyltrifluoroborate **2h** (156 mg, 0.45 mmol), and 2.5 mol% of $[Rh(cod)Cl]_2$ (3.7 mg, 0.0075 mmol), and purified by flash column chromatography (CH_2Cl_2) to give a colorless viscous oil (79 mg, 62%). R_f 0.35 (20% EtOAc/petroleum ether); IR 3255 (NH), 2961, 2826, 2855, 1747 (C=O), 1425, 1377, 1172, 1158, 1142 cm^{-1}; ^{1}H NMR (400 MHz, $CDCl_3$) δ 7.86 (2H, d, *J* = 9.0 Hz, Ar**H**), 7.83–7.78 (1H, m, Ar**H**), 7.55–7.47 (2H, m, Ar**H**), 7.45 (1H, d, *J* = 2.2 Hz, Ar**H**), 7.43–7.38 (1H, m, Ar**H**), 7.38–7.33 (1H, m, Ar**H**), 7.28–7.22 (1H, m, Ar**H**), 7.12 (1H, dd, *J* = 8.2, 1.2 Hz, Ar**H**), 7.10 (1H, dd, *J* = 8.9, 2.4 Hz, Ar**H**), 5.86 (1H, dtd, *J* = 10.8, 7.4, 0.8 Hz, =C**H**CH_2), 5.66 (1H, ddt, *J* = 10.8, 9.7, 1.6 Hz, C**H**=CHCH_2), 5.44 (1H, d, *J* = 6.7 Hz, N**H**), 5.32 (1H, app t, *J* = 6.5 Hz, NHC**H**), 4.44 (1H, ddd, *J* = 9.6, 6.2, 1.0 Hz, O=CC**H**), 2.32–2.10 (2H, m, =CHC$\mathbf{H}_2$), 1.01 (3H, t, *J* = 7.5 Hz, C$\mathbf{H}_3$); ^{13}C NMR (100.6 MHz, $CDCl_3$) δ 170.7 (C), 151.3 (C), 147.8 (C), 139.7 (CH), 133.6 (C), 131.6 (C), 130.1 (CH), 129.7 (CH), 127.8 (CH), 127.7 (CH), 126.8 (CH), 126.6 (CH), 126.1 (CH), 125.6 (CH), 120.8 (C), 120.4 (CH), 120.1 (CH), 119.4 (CH), 118.3 (CH), 58.3 (CH), 48.1 (CH), 21.5 (CH_2), 13.7 (CH_3); HRMS (ESI) Exact mass calculated for $C_{23}H_{21}NO_5SNa$ $[M+Na]^+$: 446.1033, found: 446.1046.

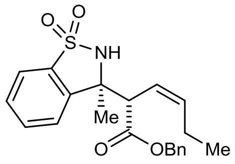

(±)-Benzyl (2*SZ*)-2-[(3*S*)-3-methyl-1,1-dioxo-2,3- dihydro-1λ^6,2-benzothiazol-3-yl]hex-3-enoate (3q). The title compound was prepared according to General Procedure C using ketimine **1f** (54 mg, 0.30 mmol) and

potassium allyltrifluoroborate **2b** (140 mg, 0.45 mmol), and purified by flash column chromatography (10% EtOAc/petroleum ether) to give a colorless solid (78 mg, 67%). R_f 0.21 (20% EtOAc/petroleum ether); m.p. 99–100 °C (Et_2O); IR 3369 (NH), 3069, 3043, 2970, 1725 (C=O), 1454, 1300, 1161, 1131, 1094, 997, 914, 844 cm^{-1}; ^{1}H NMR (400 MHz, $CDCl_3$) δ 7.78–7.69 (1H, m, Ar**H**), 7.57 (1H, ddd, *J* = 7.6, 7.6, 1.2 Hz, Ar**H**), 7.50 (1H, ddd, *J* = 7.6, 7.6, 0.9 Hz, Ar**H**), 7.40–7.30 (5H, m, Ar**H**), 7.29–7.25 (1H, m, Ar**H**), 5.96 (1H, s, N**H**), 5.47–5.35 (2H, m, C**H**=C**H**), 5.23–5.14 (2H, q, *J* = 7.1 Hz, OC**H**$_2$Ph), 3.96–3.85 (1H, m, O=CC**H**), 2.07–1.93 (1H, m, =CHC**H**$_a$H$_b$), 1.93–1.78 (1H, m, =CHCH$_a$**H**$_b$), 1.65 (3H, s, CC**H**$_3$), 0.76 (3H, t, *J* = 7.5 Hz, CH$_2$C**H**$_3$); ^{13}C NMR (100.6 MHz, $CDCl_3$) δ 171.9 (C), 141.2 (C), 138.2 (CH), 135.1 (C), 134.7 (C), 133.1 (CH), 129.6 (CH), 128.6 (2 x CH), 128.5 (CH), 128.3 (2 x CH), 122.7 (CH), 121.4 (CH), 120.3 (CH), 67.2 (CH_2) 64.0 (C), 52.2 (CH), 27.4 (CH_3), 21.1 (CH_2), 13.5 (CH_3); HRMS (ESI) Exact mass calculated for $C_{21}H_{24}NO_4S$ $[M+H]^+$: 386.1429, found: 386.1422

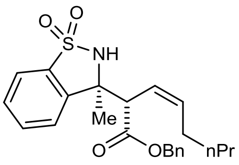

(±)-Benzyl (2*S*,*Z*)-2-[(3*S*)-3-methyl-1,1-dioxo-2,3-dihydro-1λ^6,2-benzothiazol-3-yl]oct-3-enoate (3r). The title compound was prepared according to General Procedure C using ketimine **1f** (54 mg, 0.30 mmol) and potassium allyltrifluoroborate **2c** (152 mg, 0.45 mmol), and purified by flash column chromatography (10% EtOAc/petroleum ether) to give a white solid (72 mg, 58%). R_f 0.28 (20% EtOAc/petroleum ether); m.p. 94–96 °C (Et_2O); IR 3371 (NH), 3068, 3042, 2961, 1725 (C=O), 1522, 1299, 1162, 926, 821 cm^{-1}; ^{1}H NMR (400 MHz, $CDCl_3$) δ 7.75 (1H, ddd, *J* = 7.5, 1.3, 0.8 Hz, Ar**H**), 7.56 (1H, ddd, *J* = 7.5, 7.5, 1.3 Hz, Ar**H**), 7.51 (1H, ddd, *J* = 7.5, 7.5, 0.8 Hz, Ar**H**), 7.41–7.31 (5H, m, Ar**H**), 7.28–7.24 (1H, m, Ar**H**), 5.90 (1H, s, N**H**), 5.48–5.38 (2H, m, C**H**=C**H**), 5.18 (2H, s, OC**H**$_2$Ph), 3.93–3.85 (1H, m, O=CC**H**), 2.05–1.93 (1H, m, =CHC**H**$_a$H$_b$), 1.87–1.77 (1H, m, =CHCH$_a$**H**$_b$), 1.65 (3H, s, CC**H**$_3$), 1.22–1.02 (4H, m, C**H**$_2$C**H**$_2$CH$_3$), 0.81 (3H, t, *J* = 7.1 Hz, CH$_2$C**H**$_3$); ^{13}C NMR (100.6 MHz, $CDCl_3$) δ 171.9 (C), 141.2 (C), 136.9 (CH), 135.1 (C), 134.8 (C), 133.1 (CH), 128.7 (CH), 128.6 (2 x CH), 128.4 (2 x CH), 122.7 (CH), 121.5 (CH), 120.8 (CH), 67.3 (CH_2), 64.0 (C), 52.3 (CH), 31.1 (CH_2), 27.6 (CH_3), 27.5 (CH_2), 22.2 (CH_2), 13.8 (CH_3); HRMS (ESI) Exact mass calculated for $C_{23}H_{27}NO_4SNa$ $[M+Na]^+$: 436.1568, found: 436.1558.

Allylation of Cyclic Imine 1b with Terminal Allyltrifluoroborate 2h: Ethyl (2*S*,*Z*)-2-[(4*S*)-2,2-dioxido-3,4-dihydrobenzo[*e*][1,2,3]oxathiazin-4-yl)pent-3-enoate (3s) and ethyl (3*S*)-3-[(4*S*)-2,2-dioxido-3,4-dihydrobenzo[*e*][1,2,3]oxathiazin-4-yl]pent-4-enoate (7)

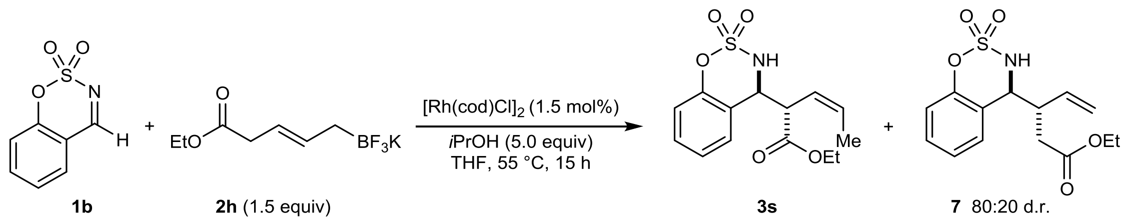

General Procedure C was followed using aldimine **1b** (59 mg, 0.30 mmol) and potassium allyltrifluoroborate **2h** (105 mg, 0.45 mmol). ^{1}H NMR analysis of the unpurified reaction mixture using 1,3,5-trimethoxybenzene as an internal standard showed the presence of *sulfamate* **3s** (54% NMR yield) and *sulfamate* **7** (80:20 d.r., 36% NMR yield). These products could not be separated cleanly by flash column chromatography, but pure samples for characterization purposes were obtained by preparative TLC (40% Et_2O/petroleum ether).

Data for **3s**: Colorless oil. R_f 0.43 (20% EtOAc/petroleum ether); IR 3259 (NH), 2958, 1722 (C=O), 1671, 1371, 1172, 754 cm^{-1}; ^{1}H NMR (400 MHz, $CDCl_3$) δ 7.37-7.31 (1H, m, Ar**H**), 7.25–6.15 (2H, m, Ar**H**), 7.06 (1H, dd, *J* = 8.2, 1.1 Hz, Ar**H**), 5.82 (1H, dqd, *J* = 10.7, 6.9, 1.0 Hz, =C**H**CH_3), 5.61 (1H, ddq, *J* = 10.7, 9.5, 1.7 Hz, CHC**H**=), 5.50 (1H, d, *J* = 6.4 Hz, N**H**), 5.18 (1H, dd, *J* = 6.4, 5.4 Hz, NHC**H**), 4.20 (2H, dq, *J* = 7.1, 2.6 Hz, OC$\mathbf{H_2}$), 4.10 (1H, ddd, *J* = 9.5, 5.4, 0.7 Hz, O=CC**H**), 1.66 (3H, dd, *J* = 6.9, 1.7 Hz, =CHC$\mathbf{H_3}$), 1.24 (3H, t, *J* = 7.1 Hz, $OCH_2C\mathbf{H_3}$); ^{13}C NMR (100.6 MHz, $CDCl_3$) δ 171.7 (C), 151.3 (C), 131.4 (CH), 129.8 (CH), 126.4 (CH), 125.4 (CH), 122.2 (CH_2), 120.9 (C), 119.2 (CH), 61.8 (CH_2), 58.2 (CH), 47.3 (CH), 14.0 (CH_3), 13.5 (CH_3); HRMS (ESI) Exact mass calculated for $C_{14}H_{17}NO_5SNa$ [M+Na]$^+$: 334.0720, found: 334.0729.

Data for **7** (Isolated as a 10:1 mixture of diastereomers): Colorless oil. R_f 0.42 (20% EtOAc/petroleum ether); IR 3286 (NH), 1980, 1731 (C=O), 1639, 1198, 1033, cm^{-1}; HRMS (ESI) Exact mass calculated for $C_{14}H_{17}NO_5SNa$ [M+Na]$^+$: 334.0720, found: 334.0732.

Major diastereomer: ^{1}H NMR (400 MHz, $CDCl_3$) δ 7.44 (1H, dt, *J* = 7.8, 1.3 Hz, Ar**H**), 7.39-7.32 (1H, m, Ar**H**), 7.26 (1H, td, *J* = 7.6, 1.4 Hz, Ar**H**), 7.04 (1H, dd, = 8.2, 1.3 Hz, Ar**H**), 5.68 (1H, ddd, *J* = 17.6, 10.5, 5.4 Hz, =C**H**), 5.35 (1H, d, *J* = 10.5 Hz, =C$\mathbf{H_a}H_b$), 5.26 (1H, ddd, *J* = 17.6, 1.1 Hz, =$CH_a\mathbf{H_b}$), 4.97 (1H, dd, *J* = 10.1, 3.0 Hz, NHC**H**), 4.53 (1H, d, *J* = 10.1 Hz, N**H**), 4.21 (2H, q, *J* = 7.1 Hz, OC$\mathbf{H_2}$), 3.81-3.71 (1H, m, CHC**H**CH=), 2.86–2.69 (2H, m, CHC$\mathbf{H_2}$), 1.30 (3H, t, *J* = 7.1 Hz, $OCH_2C\mathbf{H_3}$); ^{13}C NMR (100.6 MHz, $CDCl_3$) δ 171.7 (C), 151.9 (C), 133.3 (CH), 129.8 (CH), 126.1 (CH), 125.6 (CH), 120.6 (C), 120.2 (CH_2), 119.2 (CH), 61.1 (CH_2), 58.7 (CH), 40.0 (CH), 33.7 (CH_2), 14.2 (CH_3).

Characteristic peaks for minor diastereomer: ^{1}H NMR (400 MHz, $CDCl_3$), δ 7.26–7.21 (1H, m, Ar**H**), 7.22–7.17 (1H, m, Ar**H**), 6.03–5.95 (2H, m, =C**H** and =C**H**$_a$H$_b$), 5.30–5.29 (1H, m, =CH$_a$**H**$_b$), 4.09 (2H, qd, J = 7.1, 1.4 Hz, OC**H**$_2$), 3.46-3.40 (1H, m, CHC**H**CH=), 2.62–2.52 (2H, m, CHC**H**$_2$), 1.23 (3H, t, J = 7.1 Hz, OCH_2C**H**$_3$); ^{13}C NMR (100.6 MHz, $CDCl_3$) δ 172.5 (C), 135.5 (CH), 129.7 (C), 126.5 (CH), 125.3 (CH), 119.3 (CH), 118.9 (CH_2), 61.3 (CH_2), 59.6 (CH), 42.2 (CH), 34.0 (CH_2), 14.1 (CH_3).

(±)-Benzyl (2*S*,*Z*)-5-cyclohexyl-2-[(4*S*)-2,2-dioxo-3,4-dihydro-1,2λ^{6},3-benzoxathiazin-4-yl]pent-3-enoate (3t)

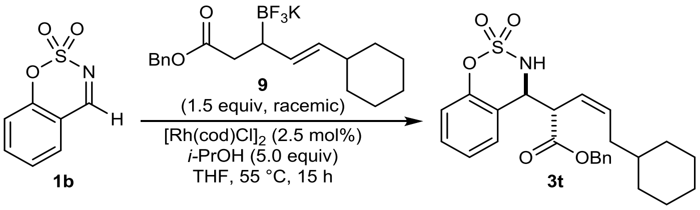

The title compound was prepared following a slight modification of General Procedure C (in the catalyst loading used) using aldimine **1b** (55 mg, 0.30 mmol), potassium allyltrifluoroborate **9** (170 mg, 0.45 mmol), and [Rh(cod)Cl]$_2$ (3.7 mg, 0.0075 mmol), and purified by flash column chromatography (10% EtOAc/petroleum ether) to give a colorless solid (89 mg, 75%). R_f 0.65 (20% EtOAc/petroleum ether); m.p. 132–134 °C (Et_2O); IR 3320 (NH), 3068, 3042, 2853, 1723 (C=O), 1487, 1453, 1426, 1377, 1192, 1173, 1107, 871, 826 cm^{-1}; ^{1}H NMR (400 MHz, $CDCl_3$) δ 7.40–7.32 (3H, m, Ar**H**), 7.32–7.24 (3H, m, Ar**H**), 7.14–7.04 (2H, m, Ar**H**), 7.03 (1H, dd, J = 8.2, 1.0 Hz, Ar**H**), 5.77–5.68 (1H, m, =C**H**CH_2), 5.62–5.54 (1H, m, C**H**=$CHCH_2$), 5.49 (1H, d, J = 6.5 Hz, N**H**), 5.20–5.10 (3H, m, NHC**H** and OC**H**$_2$Ph), 4.14 (1H, ddd, J = 9.6, 5.6, 0.8 Hz, O=CC**H**), 2.02–1.84 (2H, m, =CHC**H**$_2$), 1.74–1.57 (5H, m, cyclohexyl protons), 1.32–1.05 (4H, m, cyclohexyl protons), 0.92–0.75 (2H, m, cyclohexyl protons); ^{13}C NMR (100.6 MHz, $CDCl_3$) δ 171.6 (C), 151.2 (C), 136.3 (CH), 134.9 (C), 129.8 (CH), 128.62 (2 x CH), 128.57 (2 x CH), 128.4 (CH), 126.4 (CH), 125.4 (CH), 121.3 (CH), 120.7 (C), 119.1 (CH), 67.4 (CH_2), 58.2 (CH), 47.8 (CH), 37.8 (CH), 35.6 (CH_2), 33.2 (CH_2), 32.9 (CH_2), 26.3 (CH_2), 26.2 (2 x CH_2); HRMS (ESI) Exact mass calculated for $C_{25}H_{29}NO_5SNa$ $[M+Na]^+$: 478.1664, found: 478.1661.

Recrystallization of **3t** from Et_2O gave crystals that were suitable for X-ray diffraction:

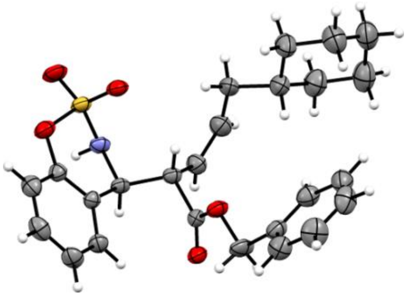

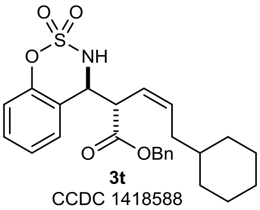

3t

CCDC 1418588

Note: A resolution took place during recrystallization, and the crystal analysed was composed of molecules of a single enantiomer only. Furthermore, the cyclohexyl group and the adjacent methylene group were disordered over two positions (disorder not shown, for clarity). The occupancies of the two components were refined competitively, converging at a ratio of 0.74:0.26. Enhanced rigid bond and similarity restraints were applied to the thermal parameters of the disordered atoms.

6. Investigation of Absolute Stereochemical Transfer with (*R*)-2a

Ethyl (2*S*,*Z*)-2-[(4*S*)-2,2-dioxo-3,4-dihydro-1,2λ^6,3-benzoxathiazin-4-yl]hex-3-enoate [(*S*,*S*)-3b]

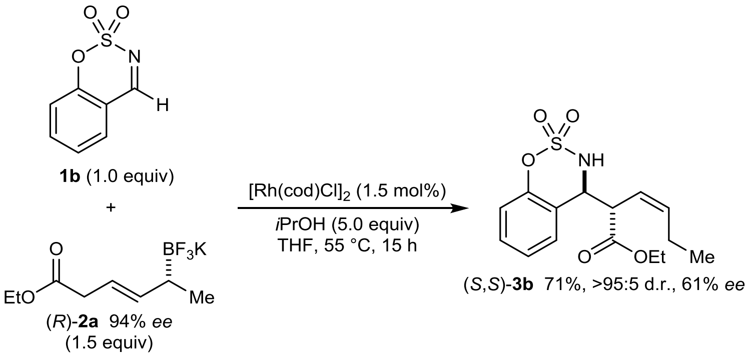

The title compound was prepared according to General Procedure C (see page 16) using aldimine **1b** (55 mg, 0.30 mmol) and potassium allyltrifluoroborate (*R*)-**2a** (112 mg, 0.45 mmol) and purified by flash column chromatography (10% EtOAc/petroleum ether) to give a colorless solid (69 mg, 71%). Spectroscopic data for racemic **3b** are reported on page 18. Enantiomeric excess was determined by HPLC with a Chiralpak AD–H column (90:10 *i*-hexane:*i*-PrOH, 1.0 mL/min, 210.4 nm, 25 °C); t_r (minor) = 9.5 min; t_r (major) = 11.0 min, 61% ee.

Data file: C:\CHEM32\1\DATA\JIM\DEF_LC 2014-07-25 12-37-38\JIM223 1 ADH 1 90 10.D
Sample name: JIM223 1 ADH 1 90 10
Instrument: AGILENT 1260
Injection date: 7/25/2014 12:49:22 PM
Acq. method: ADH90B10A.60MIN.1.0 ML.M

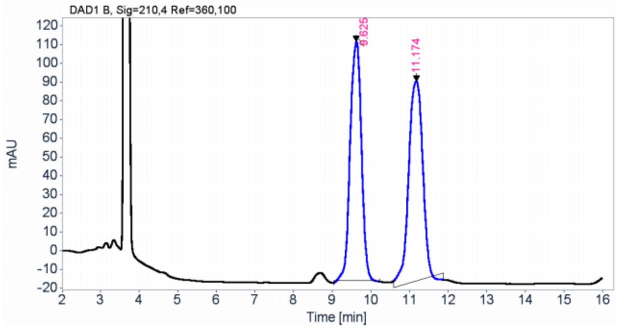

Signal: DAD1 B, Sig=210,4 Ref=360,100

RT [min]	Type	Width [min]	Area	Height	Area%
9.625	MM	0.3362	2566.653	127.2439	48.74
11.174	MM	0.4224	2699.594	106.5271	51.26

Data file: C:\CHEM32\1\DATA\JIM\DEF_LC 2014-10-22 17-40-18\JIM305 1 ADH 1 90 10.D
Sample name: JIM305 1 ADH 1 90 10
Instrument: AGILENT 1260
Injection date: 10/22/2014 8:16:46 PM
Acq. method: ADH90B10A.25MIN.1.0 ML.M

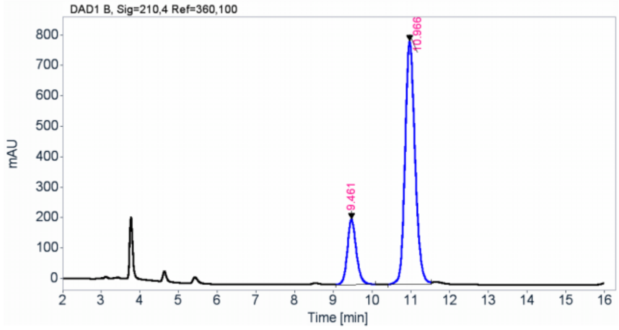

Signal: DAD1 B, Sig=210,4 Ref=360,100

RT [min]	Type	Width [min]	Area	Height	Area%
9.461	MM	0.2694	3474.096	214.8947	19.55
10.966	MF	0.2979	14299.034	799.9875	80.45

Ethyl (2*S*,*Z*)-2-[(3*S*)-3-methyl-1,1-dioxo-2,3-dihydro-1λ^6,2-benzothiazol-3-yl]hex-3-enoate [(*S*,*S*)-3f]

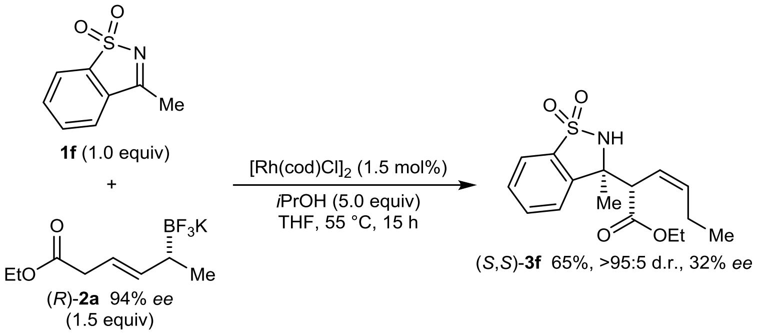

The title compound was prepared according to General Procedure C using ketimine **1f** (54 mg, 0.30 mmol) and potassium allyltrifluoroborate (*R*)-**2a** (112 mg, 0.45 mmol) and purified by flash column chromatography (10% EtOAc/petroleum ether) to give a colorless solid (63 mg, 65%). Spectroscopic data for racemic **3f** are reported on page 20. Enantiomeric excess was determined by HPLC with a Chiralpak OD–H column (90:10 *i*-hexane:*i*-PrOH, 1.0 mL/min, 210.4 nm, 25 °C); t_r (major) = 13.3 min; t_r (minor) = 14.9 min, 32% ee.

Data file: C:\CHEM32\1\DATA\AMAEL\DEF_LC 2014-10-22 08-29-58\JIM243 2 ODH 1 90 10.D
Sample name: JIM243 2 ODH 1 90 10
Instrument: AGILENT 1260
Injection date: 10/22/2014 1:55:51 PM
Acq. method: ODH90B10A.1.0ML.35 MIN.M

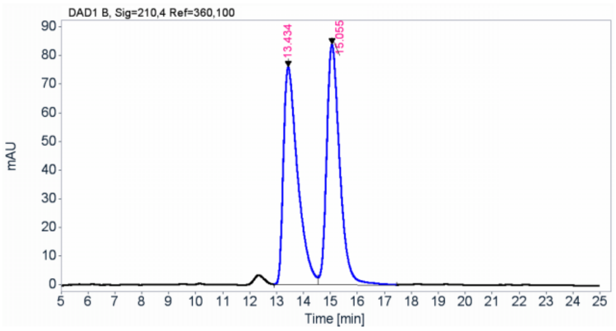

Signal: DAD1 B, Sig=210,4 Ref=360,100

RT [min]	Type	Width [min]	Area	Height	Area%
13.434	BV	0.5470	2772.636	75.7483	49.61
15.055	VB	0.5139	2815.992	83.8243	50.39

Data file: C:\CHEM32\1\DATA\AMAEL\DEF_LC 2014-12-02 11-07-35\JIM347A 1 ODH 1 90 10.D
Sample name: JIM347A 1 ODH 1 90 10
Instrument: AGILENT 1260
Injection date: 12/2/2014 1:42:15 PM
Acq. method: ODH90B10A.1.0ML.35 MIN.M

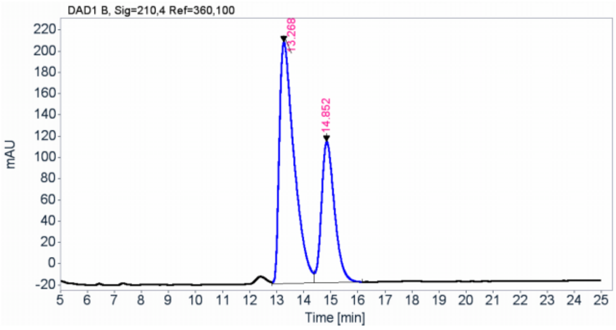

Signal: DAD1 B, Sig=210,4 Ref=360,100

RT [min]	Type	Width [min]	Area	Height	Area%
13.268	VV	0.5600	8612.076	227.3275	65.85
14.852	VB	0.5141	4465.489	132.8732	34.15

7. Enantioselective Allylation of Cyclic Imines

Evaluation of Chiral Diene Ligands (with 1.5 Equiv of the Potassium Allyltrifluoroborate)

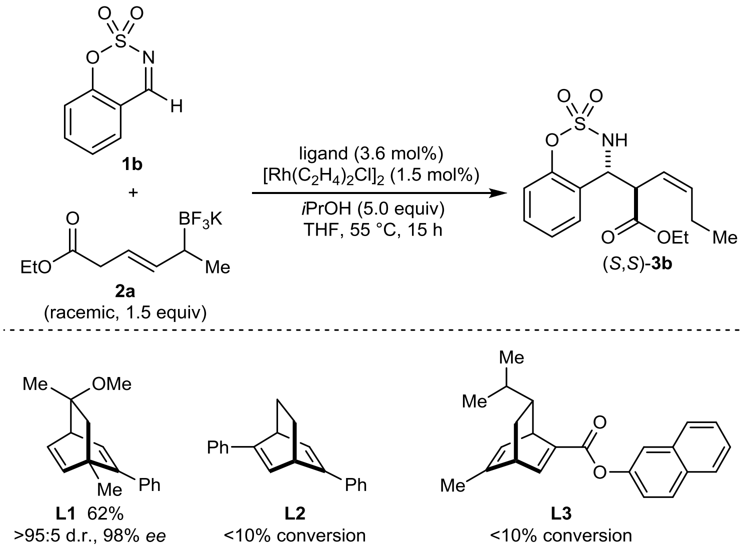

Reactions were conducted using 0.10 mmol of **1b**.

Using Racemic Potassium Allyltrifluoroborates: General Procedure D

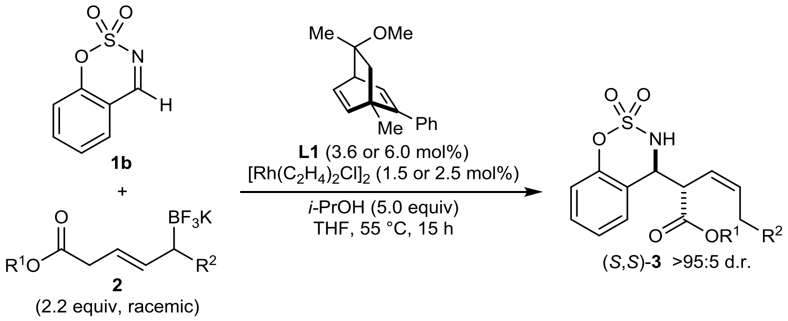

A microwave vial containing the appropriate cyclic imine (0.30 mmol) and the appropriate potassium allyltrifluoroborate (0.66 mmol) was flushed with N_2 before catalyst solution **A** or **B** (see below for their preparation, 3.0 mL) and *i*-PrOH (115 µL, 1.50 mmol) were added. The mixture was heated at 55 °C for 15 h. The reaction was cooled to room temperature, diluted with EtOAc (10 mL), and filtered through a silica plug eluting with EtOAc. The filtrate was concentrated *in vacuo* and the residue was purified by flash column chromatography to give the allylation product.

Preparation of Catalyst Solution A: A microwave vial containing $[Rh(C_2H_4)_2Cl]_2$ (8.8 mg, 0.023 mmol) and **L1**[12] (13.0 mg, 0.054 mmol) was flushed with N_2. Anhydrous THF (15 mL) was added and the mixture was stirred at 50 °C for 30 min to give a 3.0 mM solution of Rh(I)/**L1**.

12. (a) C. Fischer, C. Defieber, T. Suzuki, E. M. Carreira, *J. Am. Chem. Soc.* **2004**, *126*, 1628–1629. (b) T. Gendrineau, O. Chuzel, H. Eijsberg, J.-P. Genet, S. Darses, *Angew. Chem. Int. Ed.* **2008**, *47,* 7669–7672.

Preparation of Catalyst Solution B: A microwave vial containing $[Rh(C_2H_4)_2Cl]_2$ (14.6 mg, 0.0375 mmol) and **L1**[12] (21.6 mg, 0.09 mmol) was flushed with N_2. Anhydrous THF (15 mL) was added and the mixture was stirred at 50 °C for 30 min to give a 5.0 mM solution of Rh(I)/**L1**.

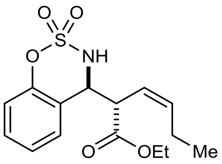

Ethyl (2*S*,*Z*)-2-[(4*S*)-2,2-dioxo-3,4-dihydro-1,2λ^6,3-benzoxathiazin-4-yl]hex-3-enoate [(*S*,*S*)-3b]. The title compound was prepared according to General Procedure D using aldimine **1b** (55 mg, 0.30 mmol), potassium allyltrifluoroborate **2a** (164 mg, 0.66 mmol), and catalyst solution A (3.0 mL, 3.0 mol% Rh(I)/**L1**), and purified by flash column chromatography (10% EtOAc/petroleum ether) to give a colorless solid (70 mg, 72%). Spectroscopic data for racemic **3b** are reported on page 18. $[\alpha]_D^{20}$ –62.5 (*c* 1.0, $CHCl_3$); m.p. 92–94 °C (*i*-PrOH/Et_2O, 1:1). Enantiomeric excess was determined by HPLC with a Chiralpak AD–H column (95:5 *i*-hexane:*i*-PrOH, 1.0 mL/min, 210.4 nm, 25 °C); t_r (minor) = 18.0 min; t_r (major) = 21.8 min, 98% ee.

Data file: C:\CHEM32\1\DATA\JIM\DEF_LC 2015-08-09 17-54-45\JIM373 1 ADH 1 95 05.D
Sample name: JIM373 1 ADH 1 95 05
Instrument: AGILENT 1260
Injection date: 8/9/2015 8:40:55 PM
Acq. method: ADH95B05A.45MIN.1.0 ML.M

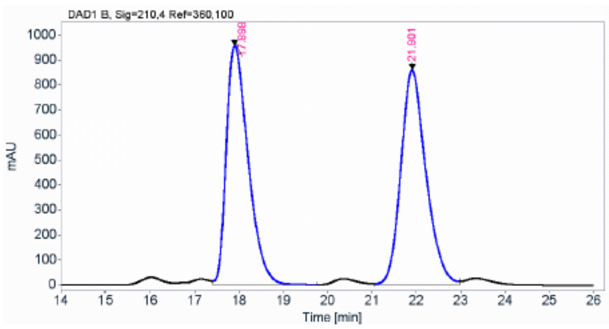

Signal: DAD1 B, Sig=210,4 Ref=360,100

RT [min]	Type	Width [min]	Area	Height	Area%
17.898	VB	0.5248	32752.844	958.0850	49.15
21.901	VV	0.5887	33883.301	861.8146	50.85

Data file: C:\CHEM32\1\DATA\JIM\DEF_LC 2015-08-09 17-54-45\JIM325 1 ADH 1 95 05.D
Sample name: JIM325 1 ADH 1 95 05
Instrument: AGILENT 1260
Injection date: 8/9/2015 7:08:51 PM
Acq. method: ADH95B05A.45MIN.1.0 ML.M

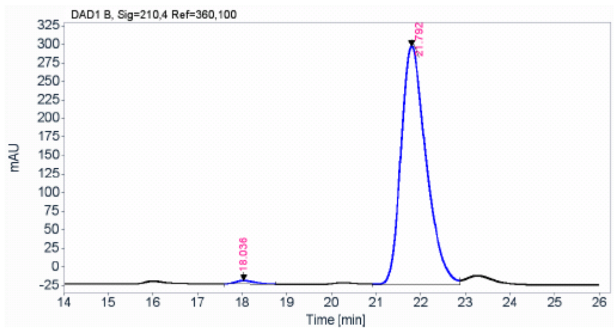

Signal: DAD1 B, Sig=210,4 Ref=360,100

RT [min]	Type	Width [min]	Area	Height	Area%
18.036	MM	0.5204	157.120	5.0319	1.23
21.792	MF	0.6533	12605.138	321.5646	98.77

Recrystallization of (*S*,*S*)-**3b** from Et_2O gave crystals that were suitable for X-ray diffraction:

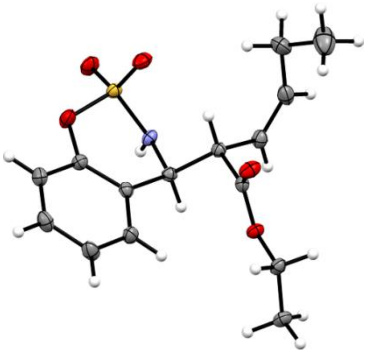

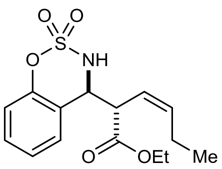

(*S*,*S*)-**3b**
CCDC 1418589
Flack parameter 0.003(14)

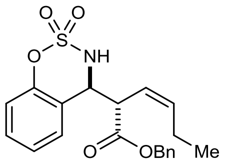

Benzyl (2*S*,*Z*)-2-[(4*S*)-2,2-dioxo-3,4-dihydro-1,2λ[6],3-benzoxathiazin-4-yl]hex-3-enoate [(*S*,*S*)-3k]. The title compound was prepared according to General Procedure D using aldimine **1b** (55 mg, 0.30 mmol), potassium allyltrifluoroborate **2b** (205 mg, 0.66 mmol), and catalyst solution A (3.0 mL, 3.0 mol% Rh(I)/**L1**), and purified by flash column chromatography (10% EtOAc/petroleum ether) to give a colorless solid (77 mg, 67%). Spectroscopic data for racemic **3k** are reported on page 22. $[\alpha]_D^{20}$ +36.7 (*c* 1.0, $CHCl_3$); m.p. 100–102 °C (Et_2O). Enantiomeric excess was determined by HPLC with a Chiralpak AD–H column (90:10 *i*-hexane:*i*-PrOH, 1.0 mL/min, 210.4 nm, 25 °C); t_r (minor) = 13.9 min; t_r (major) = 19.7 min, >99% ee.

Data file: C:\CHEM32\1\DATA\JIM\DEF_LC 2014-10-02 15-14-51\JIM292 2 ADH 1 90 10.D
Sample name: JIM292 2 ADH 1 90 10
Instrument: AGILENT 1260
Injection date: 10/2/2014 3:26:57 PM
Acq. method: ADH90B10A.60MIN.1.0 ML.M

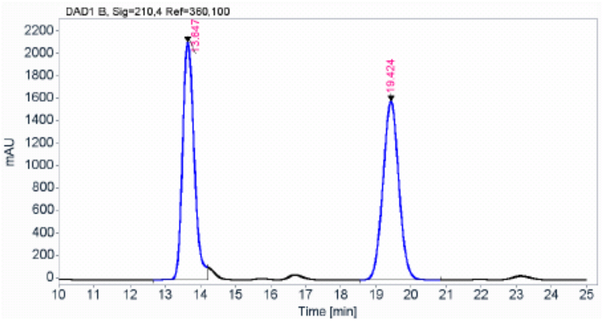

Signal: DAD1 B, Sig=210,4 Ref=360,100

RT [min]	Type	Width [min]	Area	Height	Area%
13.647	MF	0.3788	48108.973	2116.9316	48.98
19.424	MF	0.5247	50119.875	1591.9442	51.02

Data file: C:\CHEM32\1\DATA\JIM\DEF_LC 2014-12-15 11-16-43\JIM361A 1 ADH 1 90 10.D
Sample name: JIM361A 1 ADH 1 90 10
Instrument: AGILENT 1260
Injection date: 12/15/2014 11:29:20 AM
Acq. method: ADH90B10A.25MIN.1.0 ML.M

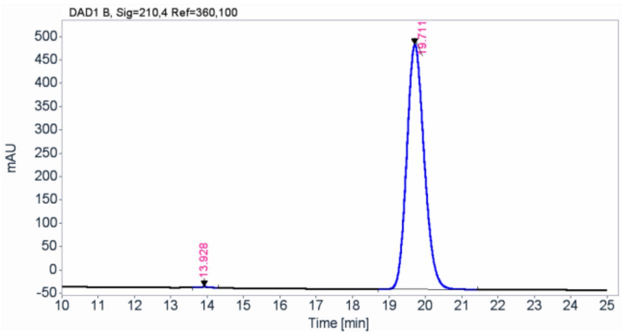

Signal: DAD1 B, Sig=210,4 Ref=360,100

RT [min]	Type	Width [min]	Area	Height	Area%
13.928	BB	0.3054	32.107	1.6049	0.18
19.711	BB	0.5171	17501.063	524.6886	99.82

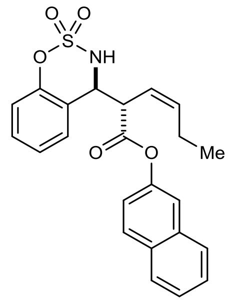

Naphthalen-2-yl (2*S*,*Z*)-2-[(4*S*)-2,2-dioxo-3,4-dihydro-1,2λ^6,3-benzoxathiazin-4-yl]hex-3-enoate [(*S*,*S*)-3p]. The title compound was prepared according to a slight modification of General Procedure D (in the equivalents of potassium allyltrifluoroborate used) using aldimine **1b** (55 mg, 0.30 mmol), potassium allyltrifluoroborate **2g** (260 mg, 0.75 mmol), and catalyst solution B (3.0 mL, 5.0 mol% Rh(I)/**L1**), and purified by flash column chromatography (CH_2Cl_2 then a second purification using 10% EtOAc/petroleum ether) to give a colorless viscous oil (34 mg, 27%). Spectroscopic data for racemic **3p** are reported on page 25. $[\alpha]^{20}_{D}$ –64.3 (*c* 1.0, $CHCl_3$). Enantiomeric excess was determined by HPLC with a Chiralpak AD–H column (90:10 *i*-hexane:EtOH, 1.0 mL/min, 210.4 nm, 25 °C); t_r (minor) = 33.2 min; t_r (major) = 36.5 min, 90% ee.

Data file: C:\CHEM32\1\DATA\JIM\DEF_LC 2015-04-01 09-35-02\JJD451 1 ADH 1 90 10 ETOH.D
Sample name: JJD451 1 ADH 1 90 10 EtOH
Instrument: AGILENT 1260
Injection date: 4/1/2015 1:22:49 PM
Acq. method: ADH90B10D.50MIN.1.0 ML.M

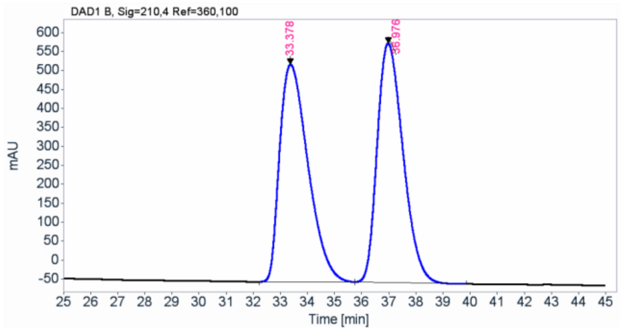

Signal: DAD1 B, Sig=210,4 Ref=360,100

RT [min]	Type	Width [min]	Area	Height	Area%
33.378	BB	1.1492	41580.855	572.6005	49.87
36.976	BB	1.0410	41789.367	630.7023	50.13

Data file: C:\CHEM32\1\DATA\JIM\DEF_LC 2015-04-01 09-35-02\JIM653 1 ADH 1 90 10 ETOH.D
Sample name: JIM653 1 ADH 1 90 10 EtOH
Instrument: AGILENT 1260
Injection date: 4/1/2015 4:53:58 PM
Acq. method: ADH90B10D.50MIN.1.0 ML.M

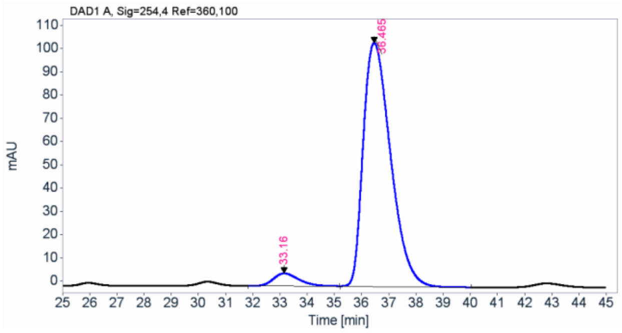

Signal: DAD1 A, Sig=254,4 Ref=360,100

RT [min]	Type	Width [min]	Area	Height	Area%
33.160	BB	0.9408	351.425	5.3891	4.59
36.465	BB	1.1019	7309.569	104.5243	95.41

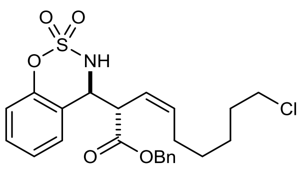

Benzyl (2*S*,*Z*)-9-chloro-2-[(4*S*)-2,2-dioxo-3,4-dihydro-1,2λ[6],3-benzoxathiazin-4-yl]non-3-enoate [(*S*,*S*)-3m]. The title compound was prepared according to General Procedure D using aldimine **1b** (55 mg, 0.30 mmol), potassium allyltrifluoroborate **2d** (255 mg, 0.66 mmol), and catalyst solution A (3.0 mL, 3.0 mol% Rh(I)/**L1**), and purified by flash column chromatography (75% to 100% CH_2Cl_2/petroleum ether) to give a colorless solid (58 mg, 41%). Spectroscopic data for racemic **3m** are reported on page 23. $[\alpha]^{20}_{D}$ –43.6 (*c* 1.0, $CHCl_3$); m.p. 86–88 °C (Et_2O). Enantiomeric excess was determined by HPLC with a Chiralpak AD–H column (90:10 *i*-hexane:*i*-PrOH, 1.0 mL/min, 210.4 nm, 25 °C); t_r (minor) = 17.2 min; t_r (major) = 25.4 min, 95% ee.

Data file: C:\CHEM32\1\DATA\JIM\DEF_LC 2015-02-13 10-27-16\JJS562B.D
Sample name: JJS562
Instrument: AGILENT 1260
Injection date: 2/13/2015 11:25:56 AM
Acq. method: ADH90B10A.60MIN.1.0 ML.M

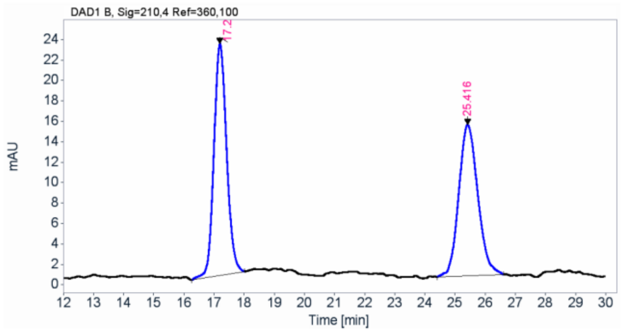

Signal: DAD1 B, Sig=210,4 Ref=360,100

RT [min]	Type	Width [min]	Area	Height	Area%
17.200	BB	0.4574	663.530	22.6720	50.60
25.416	BB	0.6553	647.754	14.7794	49.40

Data file: C:\CHEM32\1\DATA\DAVE\DEF_LC 2015-02-18 13-39-25\JJS622A.D
Sample name: jjs622
Instrument: AGILENT 1260
Injection date: 2/18/2015 1:51:36 PM
Acq. method: ADH90B10A.40MIN.1.0 ML.M

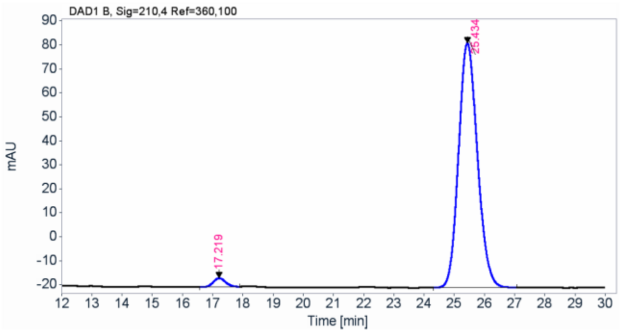

Signal: DAD1 B, Sig=210,4 Ref=360,100

RT [min]	Type	Width [min]	Area	Height	Area%
17.219	BB	0.4315	102.192	3.6183	2.26
25.434	BB	0.6699	4421.962	101.9661	97.74

Using Enantioenriched Potassium Allyltrifluoroborate

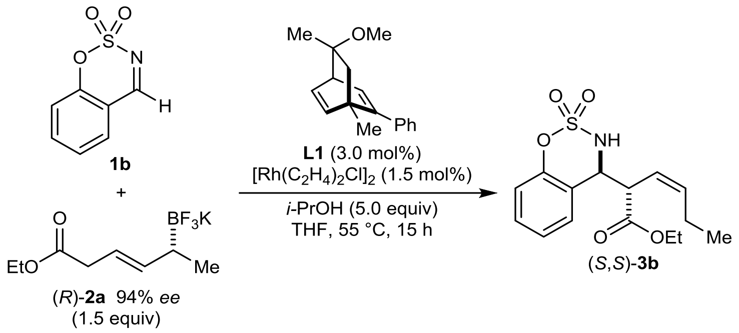

A microwave vial containing the cyclic imine **1b** (0.30 mmol) and the appropriate potassium allyltrifluoroborate (0.66 mmol) was flushed with N_2 before catalyst solution **A** (3 mL) and *i*-PrOH (115 µL, 1.50 mmol) were added. The mixture was heated at 55 °C for 15 h. The reaction was cooled to room temperature, diluted with EtOAc (10 mL), and filtered through a silica plug eluting with EtOAc. The filtrate was concentrated *in vacuo* and the residue was purified by flash column chromatography (10% EtOAc/petroleum ether) to give (*S*,*S*)-**3b** as a colorless solid (70 mg, 72%, 98% ee). Spectroscopic data for racemic **3b** and (*S*,*S*)-**3b** are reported on page 18 and 33, respectively.

Data file: C:\CHEM32\1\DATA\JIM\DEF_LC 2015-08-09 17-54-45\JIM373 1 ADH 1 95 05.D
Sample name: JIM373 1 ADH 1 95 05
Instrument: AGILENT 1260
Injection date: 8/9/2015 8:40:55 PM
Acq. method: ADH95B05A.45MIN.1.0 ML.M

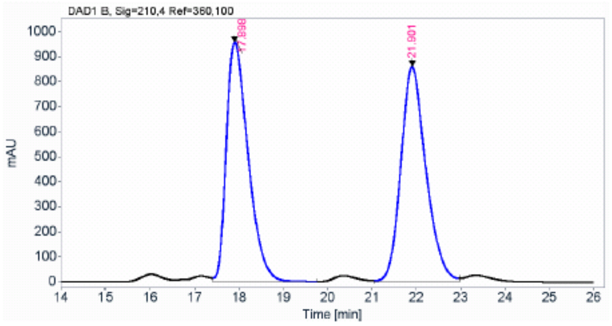

Signal: DAD1 B, Sig=210,4 Ref=360,100

RT [min]	Type	Width [min]	Area	Height	Area%
17.898	VB	0.5248	32752.844	958.0850	49.15
21.901	VV	0.5887	33883.301	861.8146	50.85

Data file: C:\CHEM32\1\DATA\JIM\DEF_LC 2015-08-09 17-54-45\JIM321 1 ADH 1 95 05.D
Sample name: JIM321 1 ADH 1 95 05
Instrument: AGILENT 1260
Injection date: 8/9/2015 7:54:55 PM
Acq. method: ADH95B05A.45MIN.1.0 ML.M

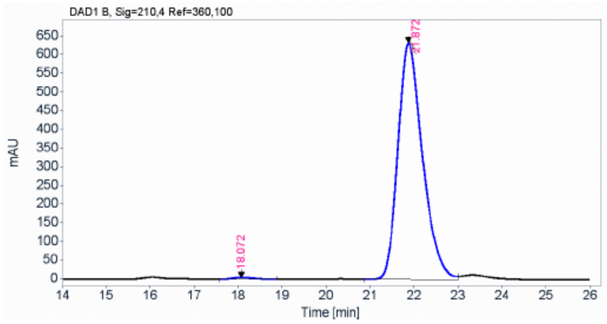

Signal: DAD1 B, Sig=210,4 Ref=360,100

RT [min]	Type	Width [min]	Area	Height	Area%
18.072	BB	0.4810	176.234	5.6020	0.71
21.872	MF	0.6467	24582.393	633.5806	99.29

8. Crossover Experiment

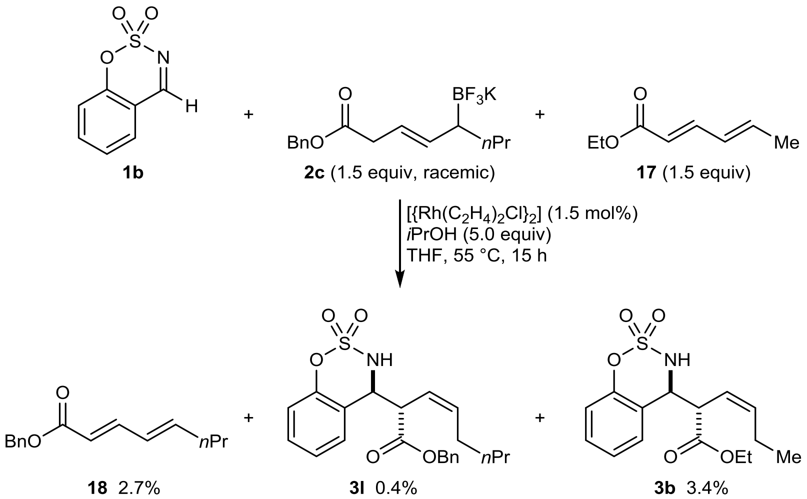

A microwave vial containing the aldimine **1b** (18.3 mg, 0.10 mmol), $[Rh(C_2H_4)_2Cl]_2$ (1.9 mg, 0.005 mmol), potassium allyltrifluoroborate **2c** (33.8 mg, 0.10 mmol), and ethyl sorbate (**17**) (14.0 mg, 0.10 mmol) was flushed with N_2 before anhydrous THF (3 mL) and *i*-PrOH (115 µL, 1.50 mmol) were added. The mixture was heated at 55 °C for 15 h. The reaction was cooled to room temperature, diluted with EtOAc (10 mL), and filtered through a silica plug eluting with EtOAc. The filtrate was concentrated *in vacuo*. Quantification of compounds **18**, **3l**, and **3b** was carried out by HPLC-MS as follows:

First, calibration was carried out using solutions containing varying concentrations of authentic samples of all three of **18**, **3b**, and **3l** in 30% H_2O/MeCN (Table 1), using an Agilent Poroshell 120 EC-C18 column (3.0 x 50 mm; particle size, 2.7 µm, gradient,[13] 0.5 mL/min, 40 °C); rt (**18**) = 6.0 min; t_r (**3b**) = 10.3 min; t_r (**3l**) = 10.6 min. Ions detected were recorded over a range of 100–1000 *m*/*z* and integration was performed on extracted ion chromatograms at 231.1377 +/– 0.005 *m*/*z* (**18**), 326.1059 +/– 0.005 *m*/*z* (**3b**), and 416.1533 +/– 0.005 *m*/*z* (**3l**). Figure 1 shows a representative example of a calibration chromatogram. Each compound solution was run twice and a good correlation between concentration and area was obtained (Figures 2–4).

Next, the crude reaction mixture was dissolved in 30% H_2O/MeCN (1 mL total). Three separate aliquots of this solution were run under equivalent chromatography conditions (Figures 5–7). Peaks with the same retention times as the authentic samples of **18**, **3l**, and **3b** were identified and confirmed as the correct compounds by HRMS analysis (Table 2). An average of the signal areas over three runs for each compound was used to calculate the mass and yields of each

13. The solvents for HPLC analysis were: solvent A: 99.9% H_2O, 0.1% HCO_2H; solvent B, 99.9% MeCN, 0.1% HCO_2H. The injection volume was 1 µL. The solvent gradient was run from 60% A, 40% B, holding for 1 min, then a linear gradient to 30% A, 70% B over 9 min, then an increased linear gradient to 100% B over 2 min before holding for 4 min.

compound in the crude reaction mixture (Table 3). The yields of compounds **18**, **3l**, and **3b** were calculated to be 2.7%, 0.4%, and 3.4%, respectively.

Compound Spectrum Report

Analysis Info

Analysis Name	D:\Data\Service Work\j_mar\cal_5_long_BB1_01_64.d	Acquisition Date	8/27/2015 4:24:46 PM
Method	lcms hystar method.m	Operator	Nottingham SoC
Sample Name	cal_5_long	Instrument	impact II 1825265.10067
Comment			

Acquisition Parameter

Source Type	ESI	Ion Polarity	Positive	Set Nebulizer	1.8 Bar
Focus	Active	Set Capillary	4500 V	Set Dry Heater	220 °C
Scan Begin	100 m/z	Set End Plate Offset	-500 V	Set Dry Gas	8.0 l/min
Scan End	1000 m/z	Set Charging Voltage	2000 V	Set Divert Valve	Source
		Set Corona	0 nA	Set APCI Heater	0 °C

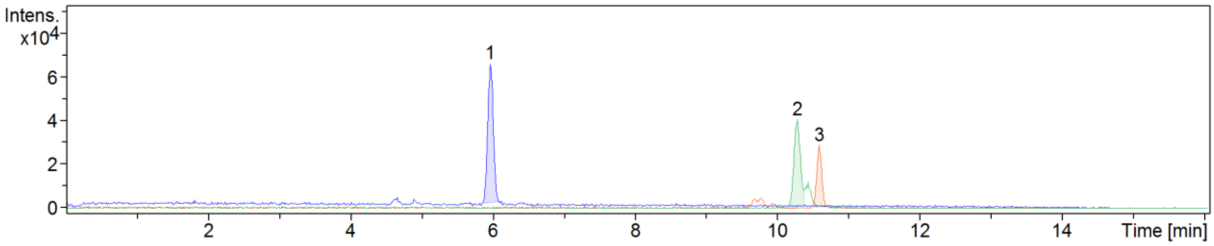

#	RT [min]	Area	Int. Type	I	S/N	Chromatogram	Max. m/z	FWHM [min]
1	6.0	336643	Manual	65978	87.7	EIC 326.1057±0.005 +All MS	211.0293	0.1
2	10.3	265719	Manual	40022	114.1	EIC 231.1376±0.005 +All MS	236.0372	0.1
3	10.6	132758	Manual	28504	135.3	EIC 416.1531±0.005 +All MS	421.0520	0.1

Figure 1: Example of a calibration chromatogram

Table 1: Calibration solutions

**Calibration solution**	**3b** (mg/mL)	Area **3b**	**3l** (mg/mL)	Area **3l**	**18** (mg/mL)	Area **18**
**1**	0.087	336643	0.108	132758	0.066	265719
**1’**	0.087	361852	0.108	134572	0.066	301507
**2**	0.174	714656	0.216	281955	0.132	413970
**2’**	0.174	720981	0.216	286859	0.132	624363
**3**	0.435	2028099	0.54	993402	0.33	1357520
**3’**	0.435	2155381	0.54	882639	0.33	1575276

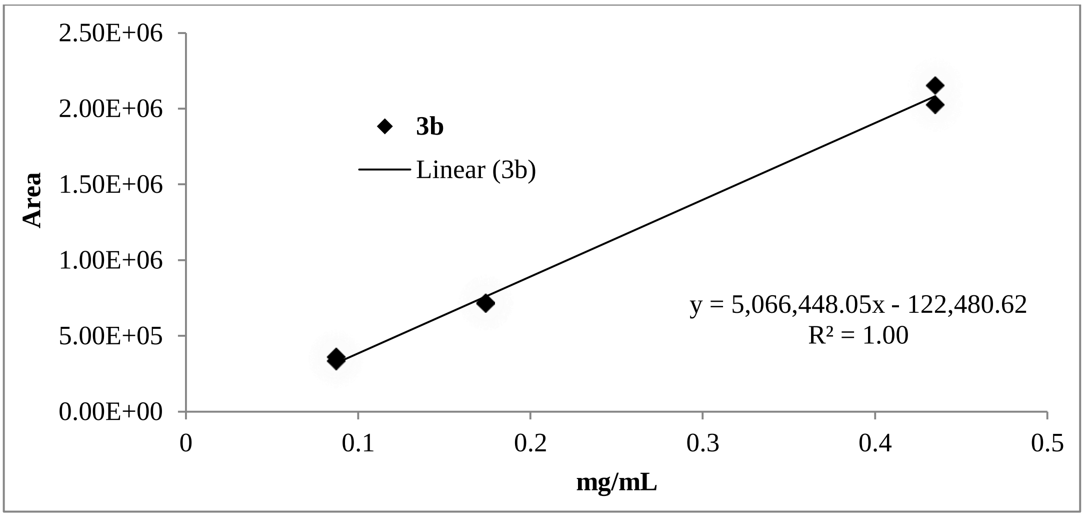

Figure 2: Calibration plot for compound **3b**

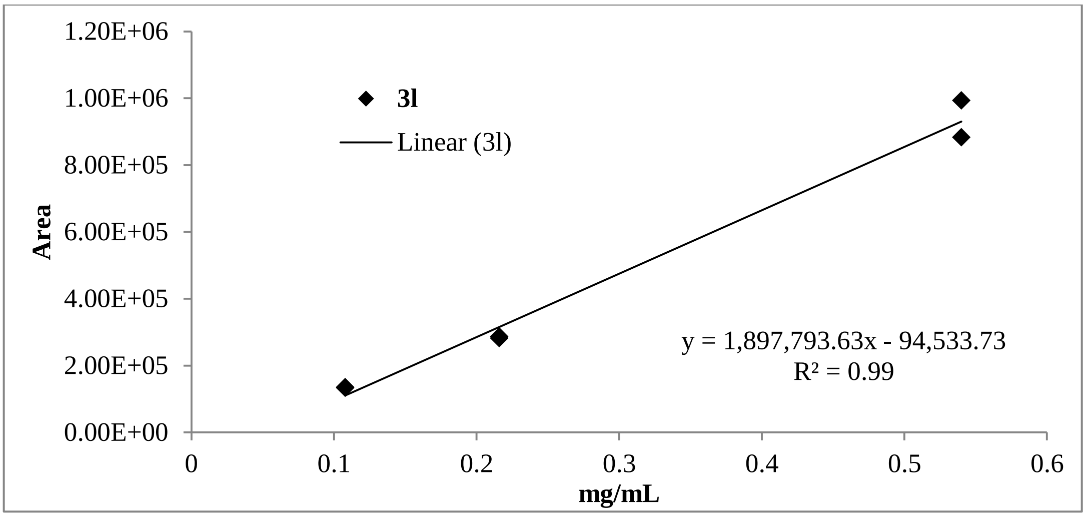

Figure 3: Calibration plot for compound **3l**

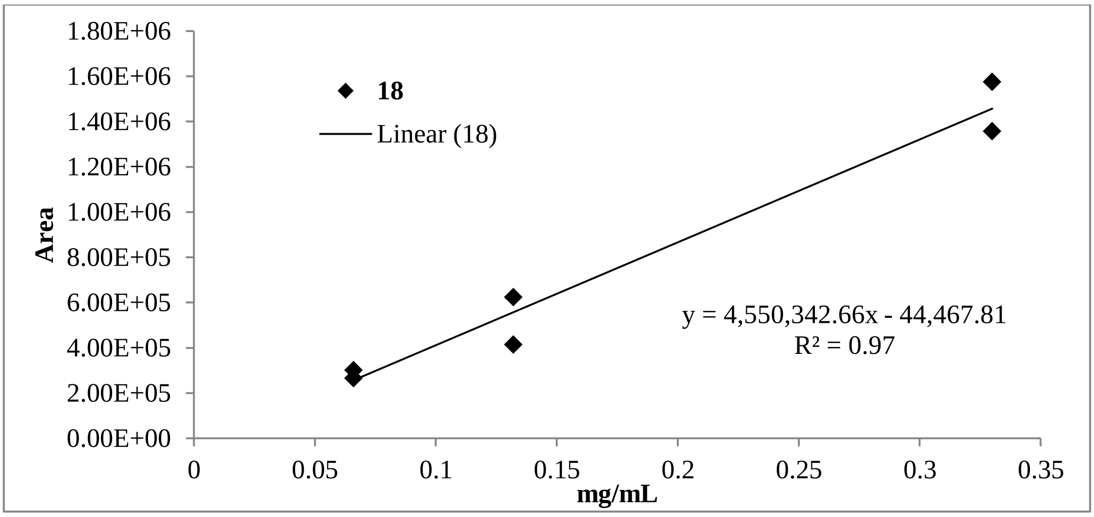

Figure 4: Calibration plot for compound

Compound Spectrum Report

Analysis Info

		Acquisition Date	8/27/2015 6:12:09 PM
Analysis Name	D:\Data\Service Work\j_mar\sample_601cr_long_BD5_02_69.d		
Method	lcms hystar method.m	Operator	Nottingham SoC
Sample Name	sample_601cr_long	Instrument	impact II 1825265.10067
Comment			

Acquisition Parameter

Source Type	ESI	Ion Polarity	Positive	Set Nebulizer	1.8 Bar
Focus	Active	Set Capillary	4500 V	Set Dry Heater	220 °C
Scan Begin	100 m/z	Set End Plate Offset	-500 V	Set Dry Gas	8.0 l/min
Scan End	1000 m/z	Set Charging Voltage	2000 V	Set Divert Valve	Source
		Set Corona	0 nA	Set APCI Heater	0 °C

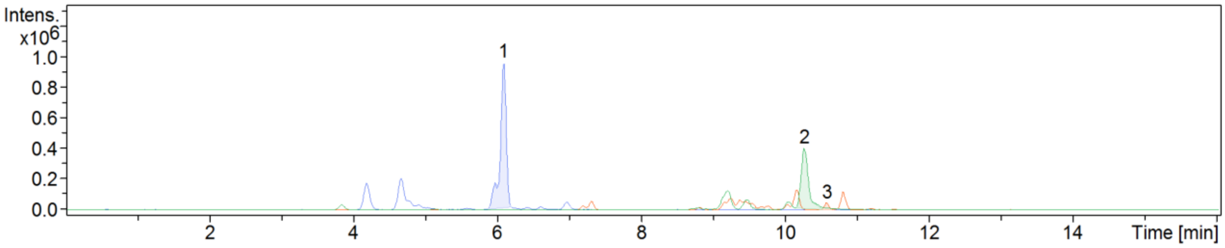

#	RT [min]	Area	Int. Type	I	S/N	Chromatogram	Max. m/z	FWHM [min]
1	6.1	5569493	Chromatogram	962108	1118.1	EIC 326.1062±0.005 +All MS	211.0288	0.1
2	10.3	2731579	Chromatogram	402483	568.7	EIC 231.1376±0.005 +All MS	231.1382	0.1
3	10.6	203540	Manual	44796	113.8	EIC 416.1541±0.005 +All MS	421.0520	0.1

Figure 5: HPLC-MS chromatogram for solution sample 1

Compound Spectrum Report

Analysis Info

		Acquisition Date	8/26/2015 5:24:37 PM
Analysis Name	D:\Data\Service Work\j_mar\sample_601cr_long_BD5_01_51.d		
Method	lcms hystar method.m	Operator	Nottingham SoC
Sample Name	sample_601cr_long	Instrument	impact II 1825265.10067
Comment			

Acquisition Parameter

Source Type	ESI	Ion Polarity	Positive	Set Nebulizer	1.8 Bar
Focus	Active	Set Capillary	4500 V	Set Dry Heater	220 °C
Scan Begin	100 m/z	Set End Plate Offset	-500 V	Set Dry Gas	8.0 l/min
Scan End	1000 m/z	Set Charging Voltage	2000 V	Set Divert Valve	Source
		Set Corona	0 nA	Set APCI Heater	0 °C

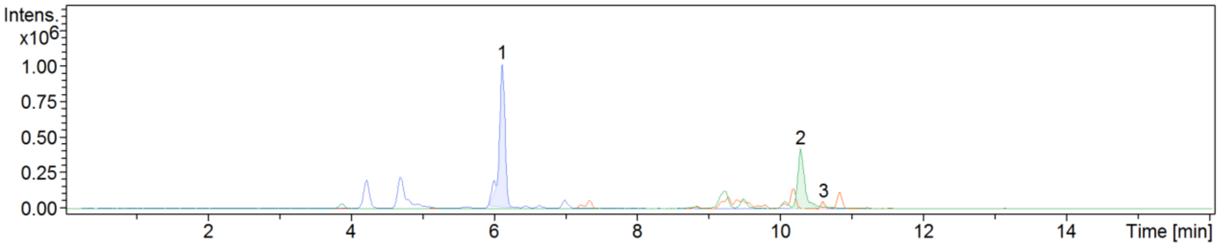

#	RT [min]	Area	Int. Type	I	S/N	Chromatogram	Max. m/z	FWHM [min]
1	6.1	5531557	Chromatogram	1015228	935.6	EIC 326.1057±0.005 +All MS	211.0292	0.1
2	10.3	2739534	Chromatogram	420048	550.0	EIC 231.1376±0.005 +All MS	231.1380	0.1
3	10.6	247817	Manual	47520	98.5	EIC 416.1531±0.005 +All MS	421.0514	0.1

Figure 6: HPLC-MS chromatogram for solution sample 2

Compound Spectrum Report

Analysis Info

Analysis Name	D:\Data\Service Work\j_mar\sample_601cr_long_BD5_02_52.d	Acquisition Date	8/26/2015 5:46:05 PM
Method	lcms hystar method.m	Operator	Nottingham SoC
Sample Name	sample_601cr_long	Instrument	impact II 1825265.10067
Comment			

Acquisition Parameter

Source Type	ESI	Ion Polarity	Positive	Set Nebulizer	1.8 Bar
Focus	Active	Set Capillary	4500 V	Set Dry Heater	220 °C
Scan Begin	100 m/z	Set End Plate Offset	-500 V	Set Dry Gas	8.0 l/min
Scan End	1000 m/z	Set Charging Voltage	2000 V	Set Divert Valve	Source
		Set Corona	0 nA	Set APCI Heater	0 °C

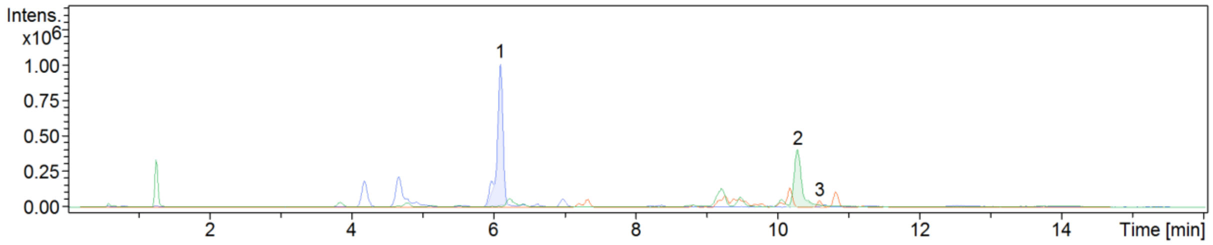

#	RT [min]	Area	Int. Type	I	S/N	Chromatogram	Max. m/z	FWHM [min]
1	6.1	5524386	Chromatogram	1017483	562.8	EIC 326.0005 +All MS	211.0292	0.1
2	10.3	2844934	Chromatogram	408806	216.2	EIC 230.9999 +All MS	231.1379	0.1
3	10.6	230809	Manual	47968	110.2	EIC 416.1536±0.005 +All MS	421.0516	0.1

Figure 7: HPLC-MS chromatogram for solution sample 3

Table 2: Crude reaction area analysis

Solution Sample	Area **3b**	Area **3l**	Area **18**
1	5569493	203540	2731579
2	5531557	247817	2739534
3	5524386	230809	2844934
**Average**	5541812	227389	2772016

Table 3: Calculated yields

	**3b**	**3l**	**18**
**Mass (mg)**	1.12	0.17	0.62
**Yield (%)**	3.44	0.41	2.69

9. NMR Spectra

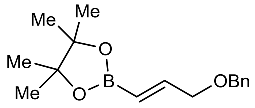

S4

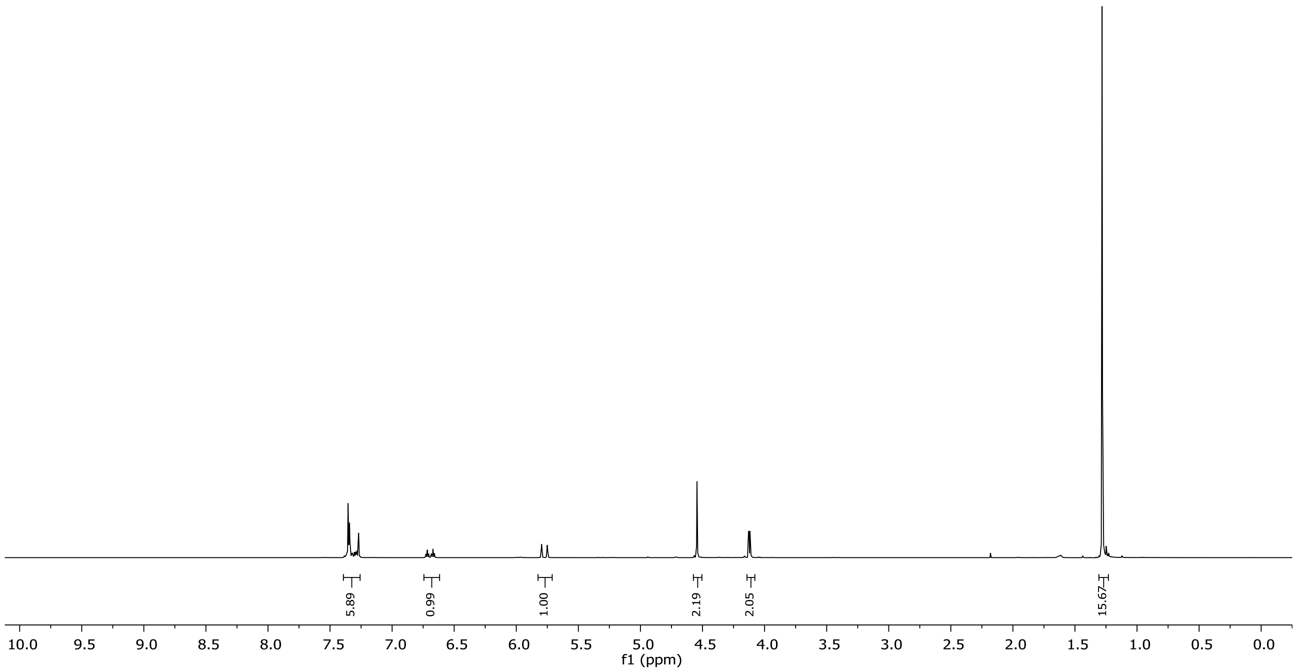

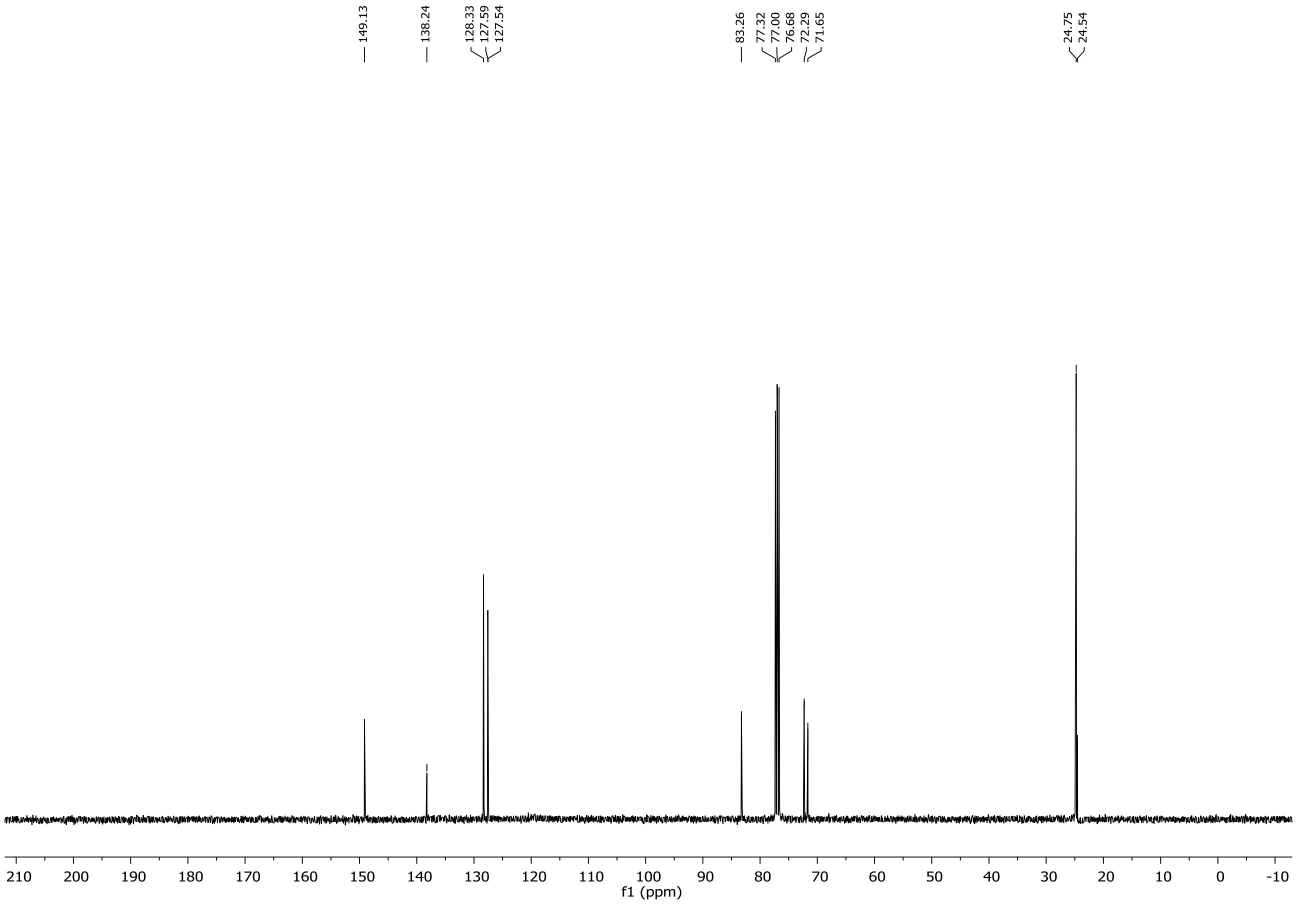

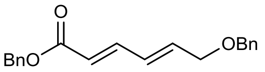
BnO
OBn

S10

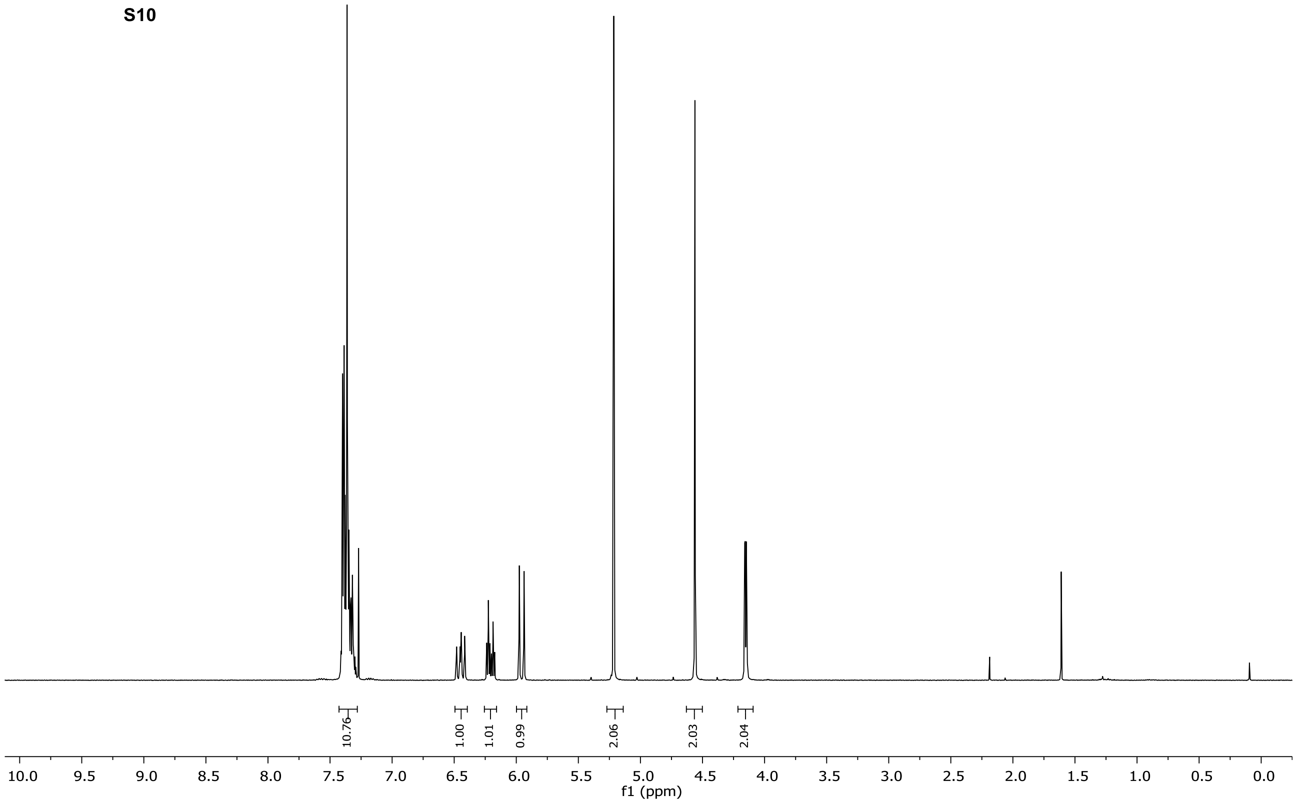
10.76
1.00
1.01
0.99
2.06
2.03
2.04
10.0 9.5 9.0 8.5 8.0 7.5 7.0 6.5 6.0 5.5 5.0 4.5 4.0 3.5 3.0 2.5 2.0 1.5 1.0 0.5 0.0
f1 (ppm)

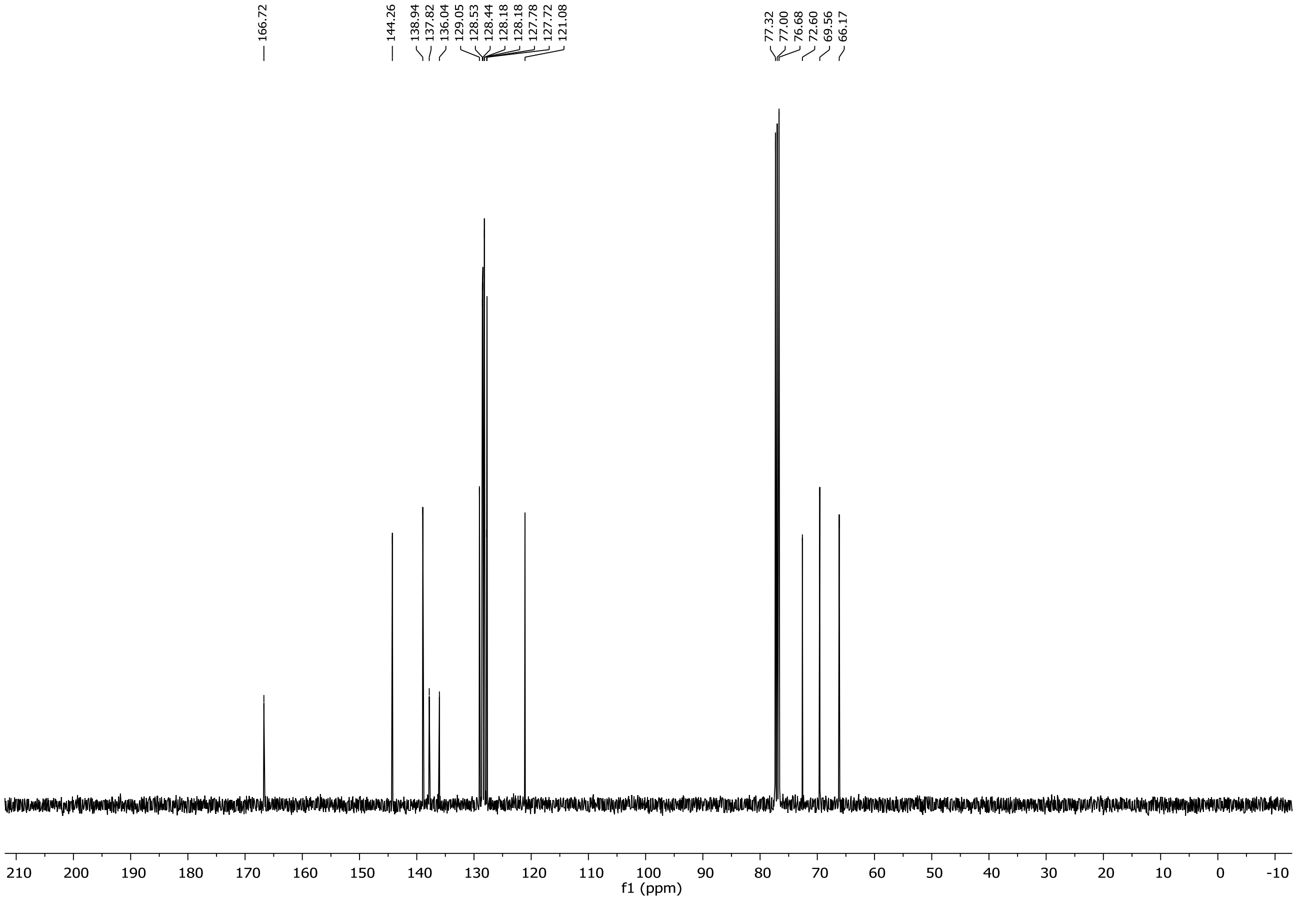
166.72
144.26
138.94
137.82
136.04
129.05
128.53
128.44
128.18
128.18
127.78
127.72
121.08
77.32
77.00
76.68
72.60
69.56
66.17
210 200 190 180 170 160 150 140 130 120 110 100 90 80 70 60 50 40 30 20 10 0 -10
f1 (ppm)

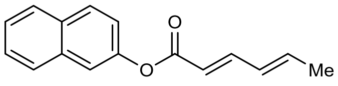

S11

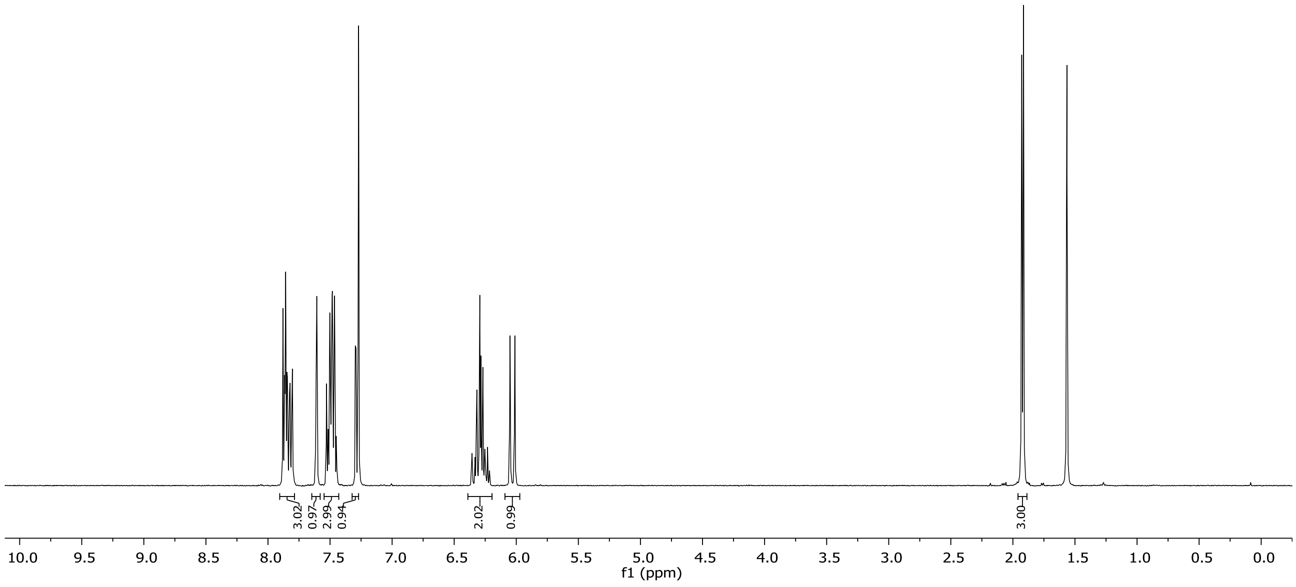

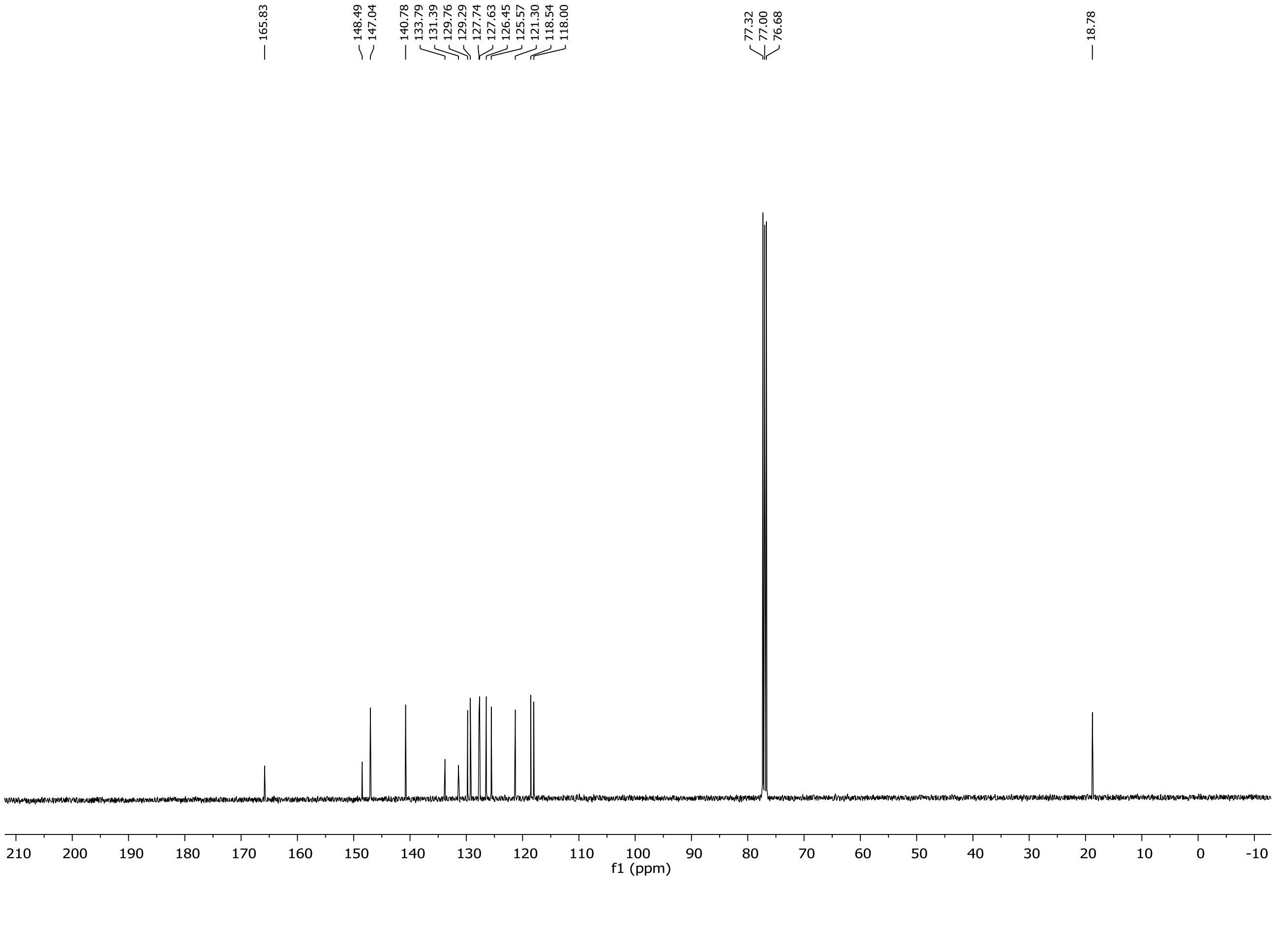

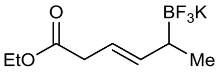
O
BF3K
EtO
Me

2a

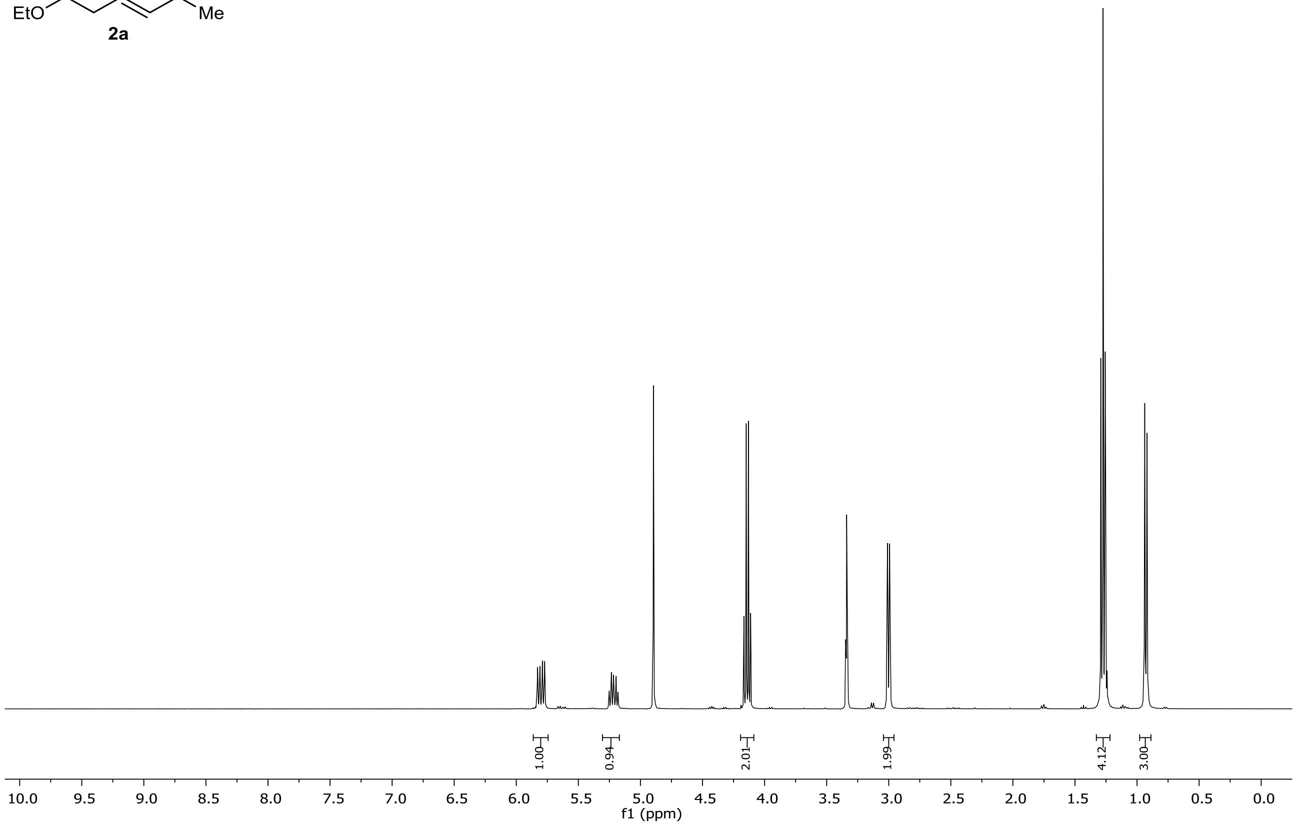
1.00
0.94
2.01
1.99
4.12
3.00
10.0 9.5 9.0 8.5 8.0 7.5 7.0 6.5 6.0 5.5 5.0 4.5 4.0 3.5 3.0 2.5 2.0 1.5 1.0 0.5 0.0
f1 (ppm)

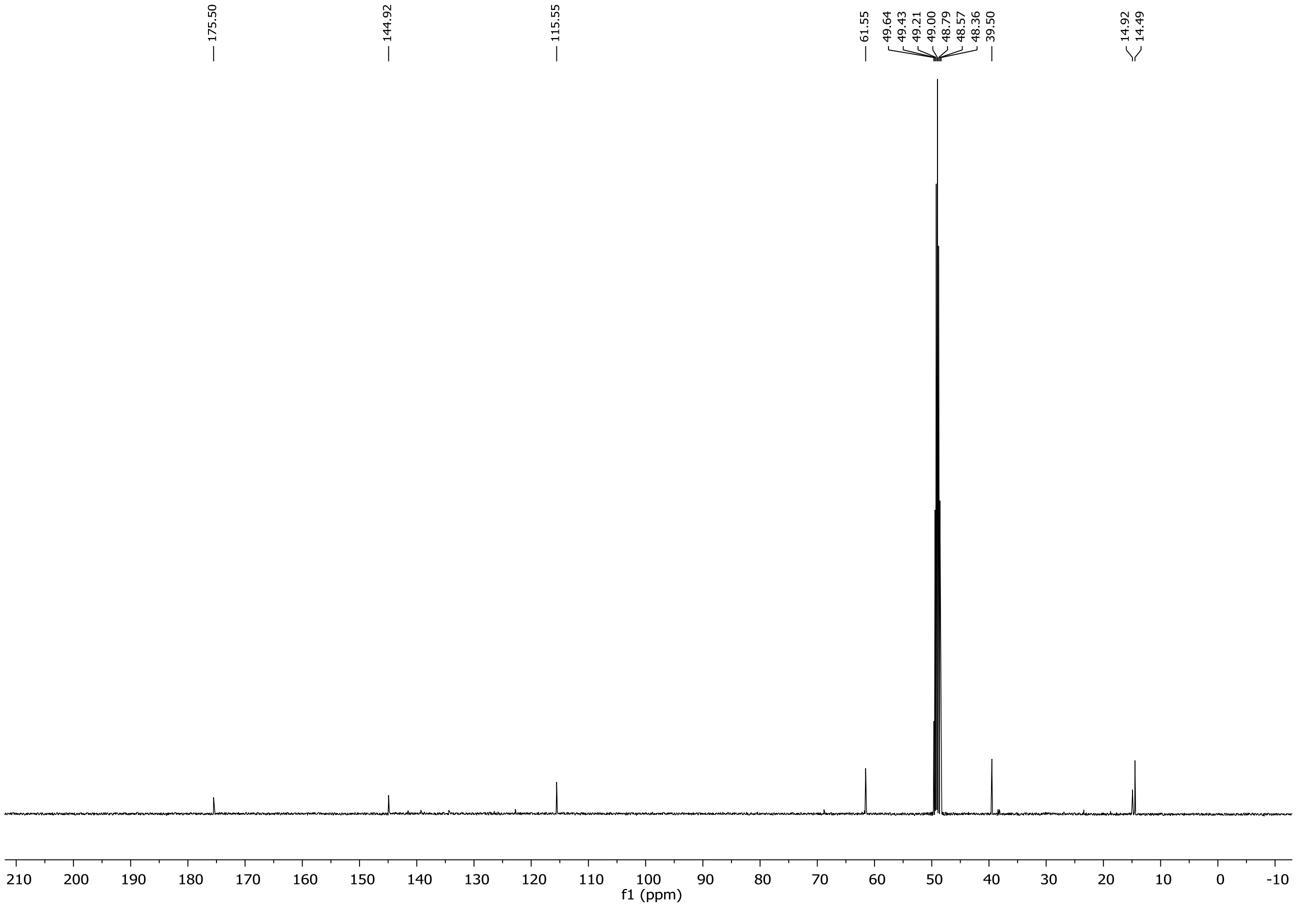
175.50
144.92
115.55
61.55
49.64
49.43
49.21
49.00
48.79
48.57
48.36
39.50
14.92
14.49
210 200 190 180 170 160 150 140 130 120 110 100 90 80 70 60 50 40 30 20 10 0 -10
f1 (ppm)

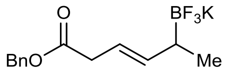
O
BF3K
BnO
Me

2b

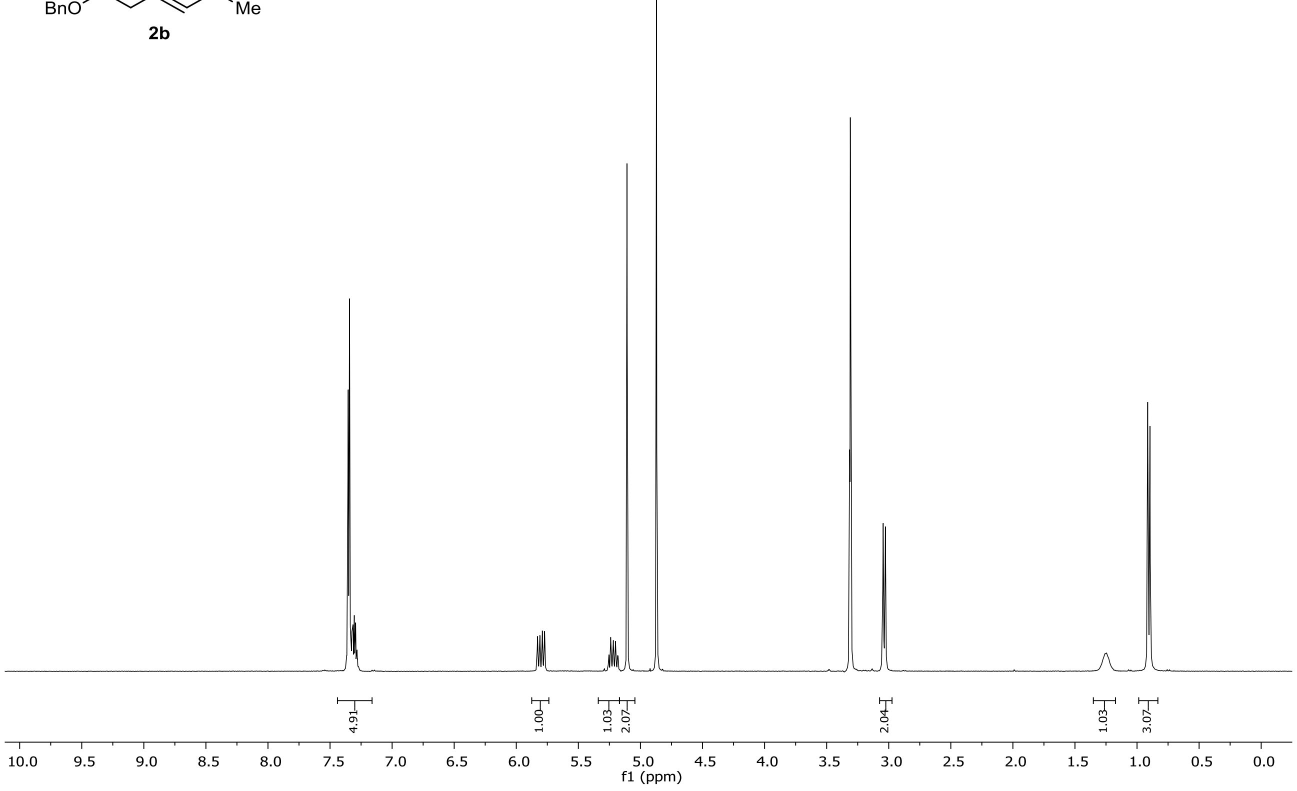
4.91
1.00
1.03
2.07
2.04
1.03
3.07
10.0 9.5 9.0 8.5 8.0 7.5 7.0 6.5 6.0 5.5 5.0 4.5 4.0 3.5 3.0 2.5 2.0 1.5 1.0 0.5 0.0
f1 (ppm)

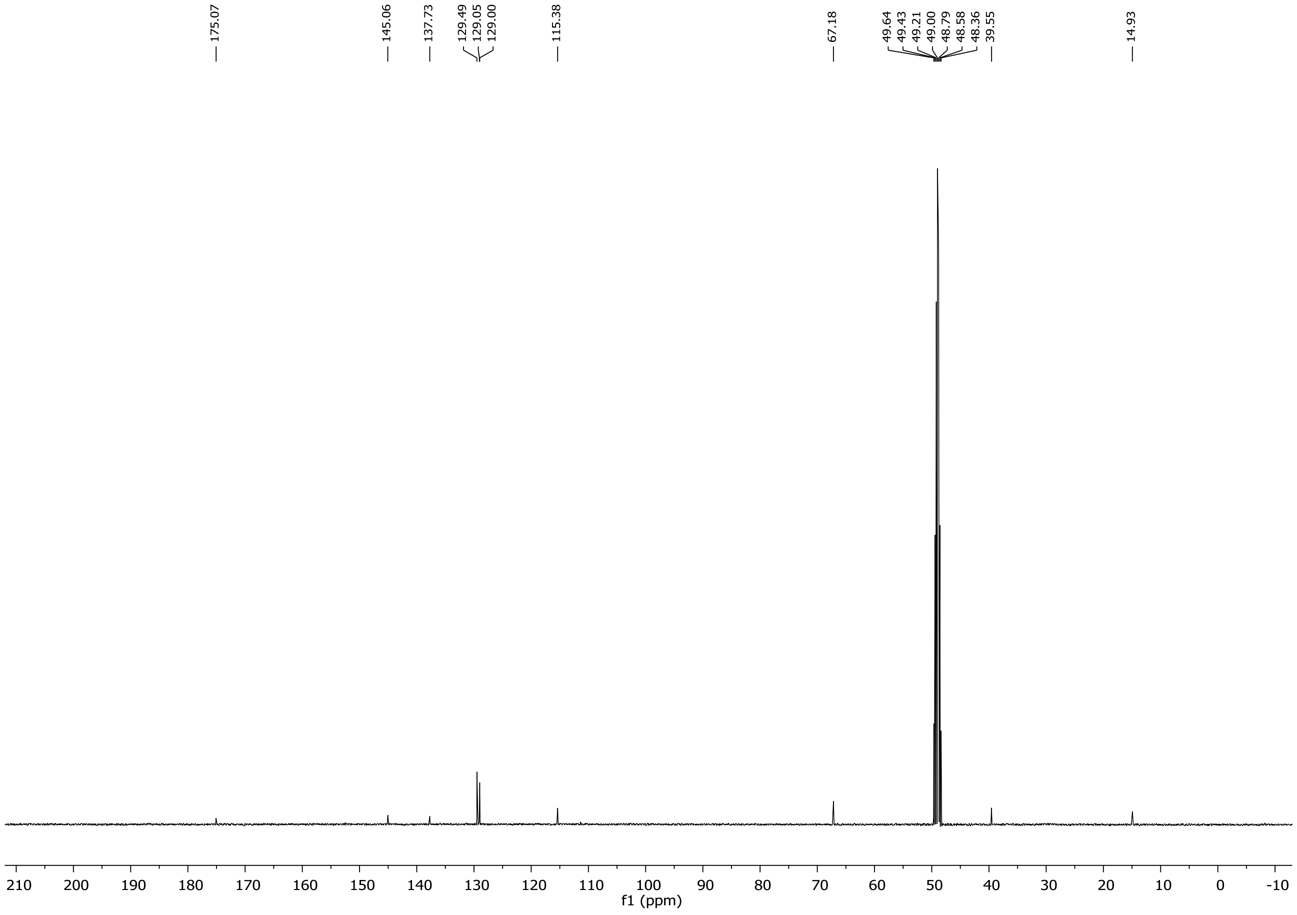
175.07
145.06
137.73
129.49
129.05
129.00
115.38
67.18
49.64
49.43
49.21
49.00
48.79
48.58
48.36
39.55
14.93
210 200 190 180 170 160 150 140 130 120 110 100 90 80 70 60 50 40 30 20 10 0 -10
f1 (ppm)

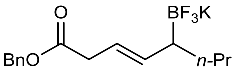
O
BF3K
BnO
n-Pr

2c

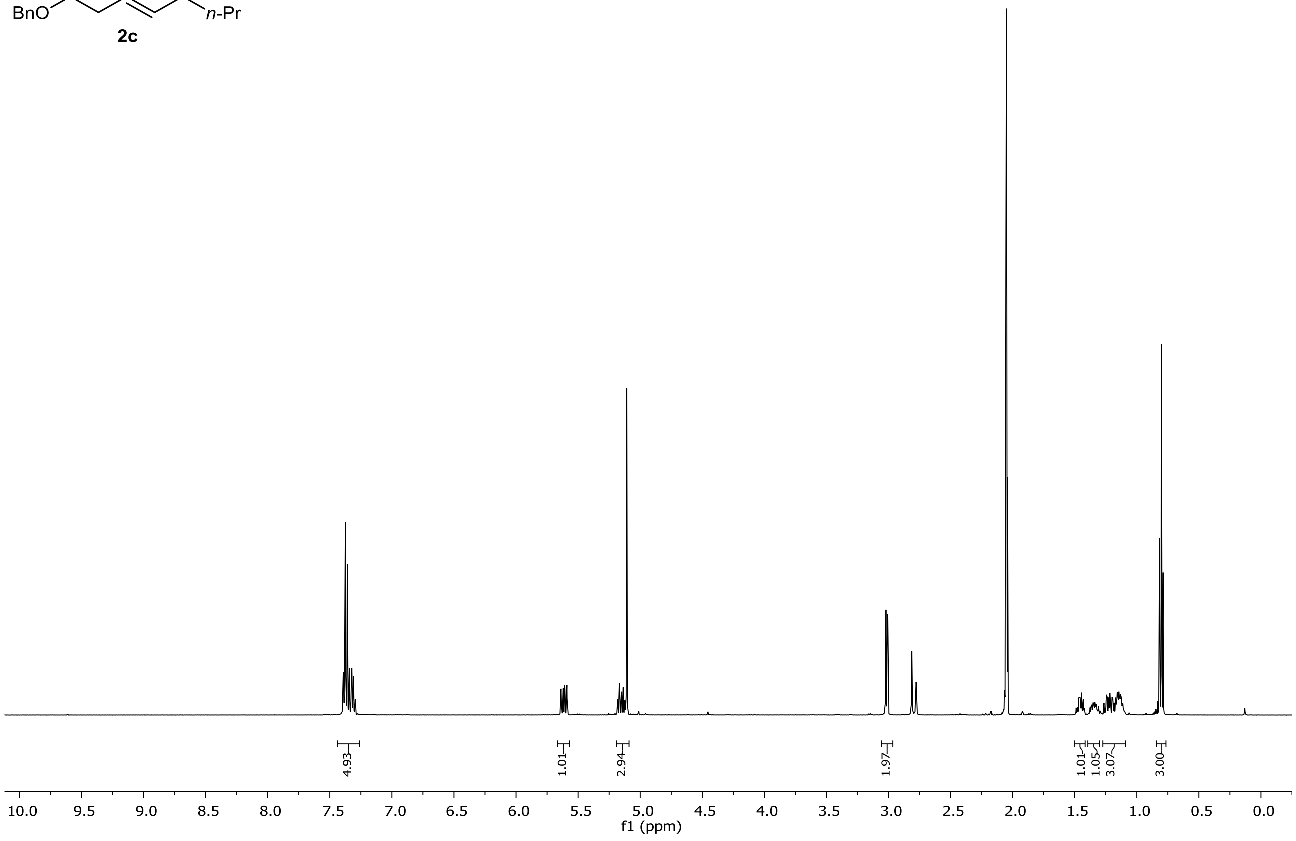
4.93
1.01
2.94
1.97
1.01
1.05
3.07
3.00
10.0 9.5 9.0 8.5 8.0 7.5 7.0 6.5 6.0 5.5 5.0 4.5 4.0 3.5 3.0 2.5 2.0 1.5 1.0 0.5 0.0
f1 (ppm)

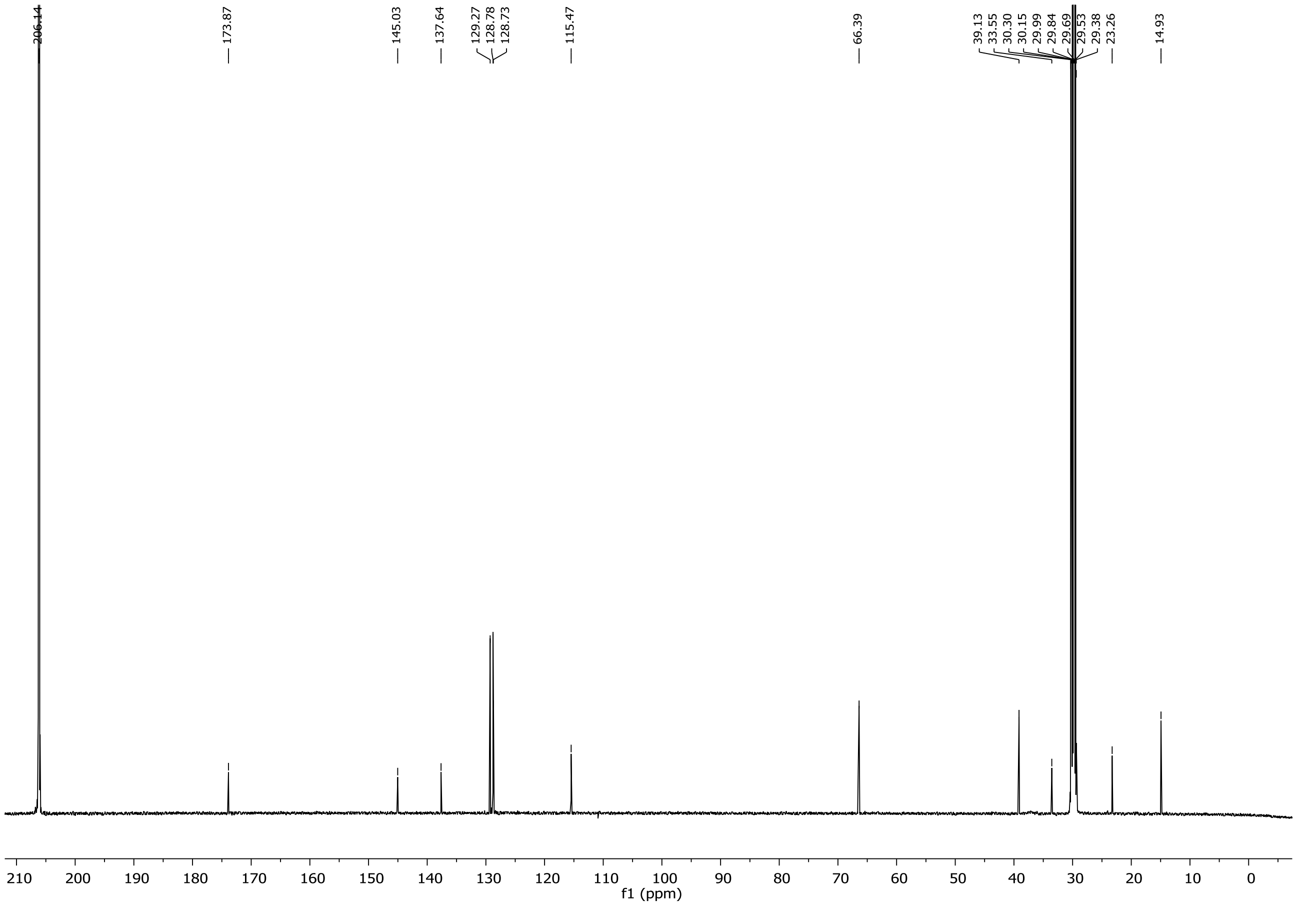
206.14
173.87
145.03
137.64
129.27
128.78
128.73
115.47
66.39
39.13
33.55
30.30
30.15
29.99
29.84
29.69
29.53
29.38
23.26
14.93
210 200 190 180 170 160 150 140 130 120 110 100 90 80 70 60 50 40 30 20 10 0
f1 (ppm)

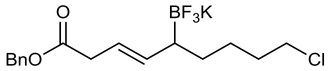
O
BF3K
BnO
Cl

2d

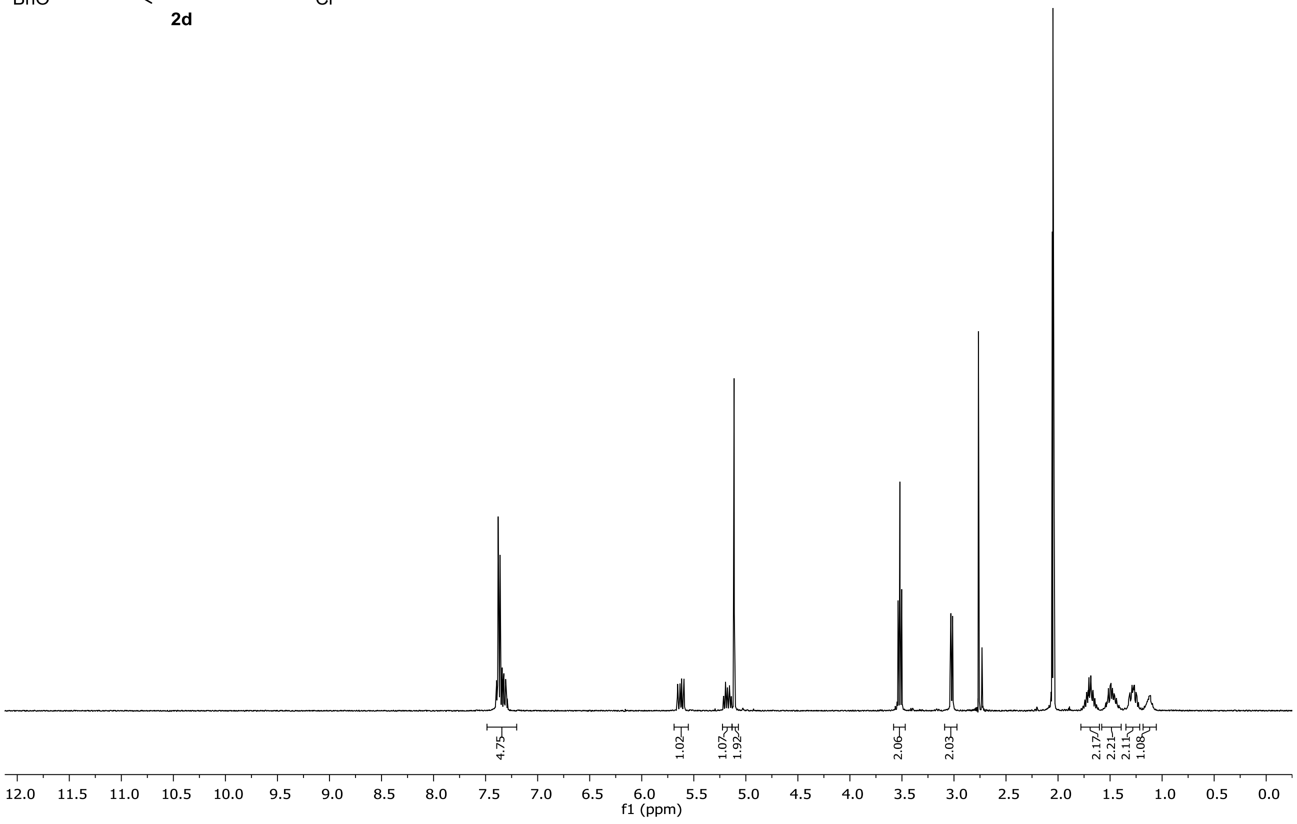
4.75
1.02
1.07
1.92
2.06
2.03
2.17
2.21
2.11
1.08
12.0 11.5 11.0 10.5 10.0 9.5 9.0 8.5 8.0 7.5 7.0 6.5 6.0 5.5 5.0 4.5 4.0 3.5 3.0 2.5 2.0 1.5 1.0 0.5 0.0
f1 (ppm)

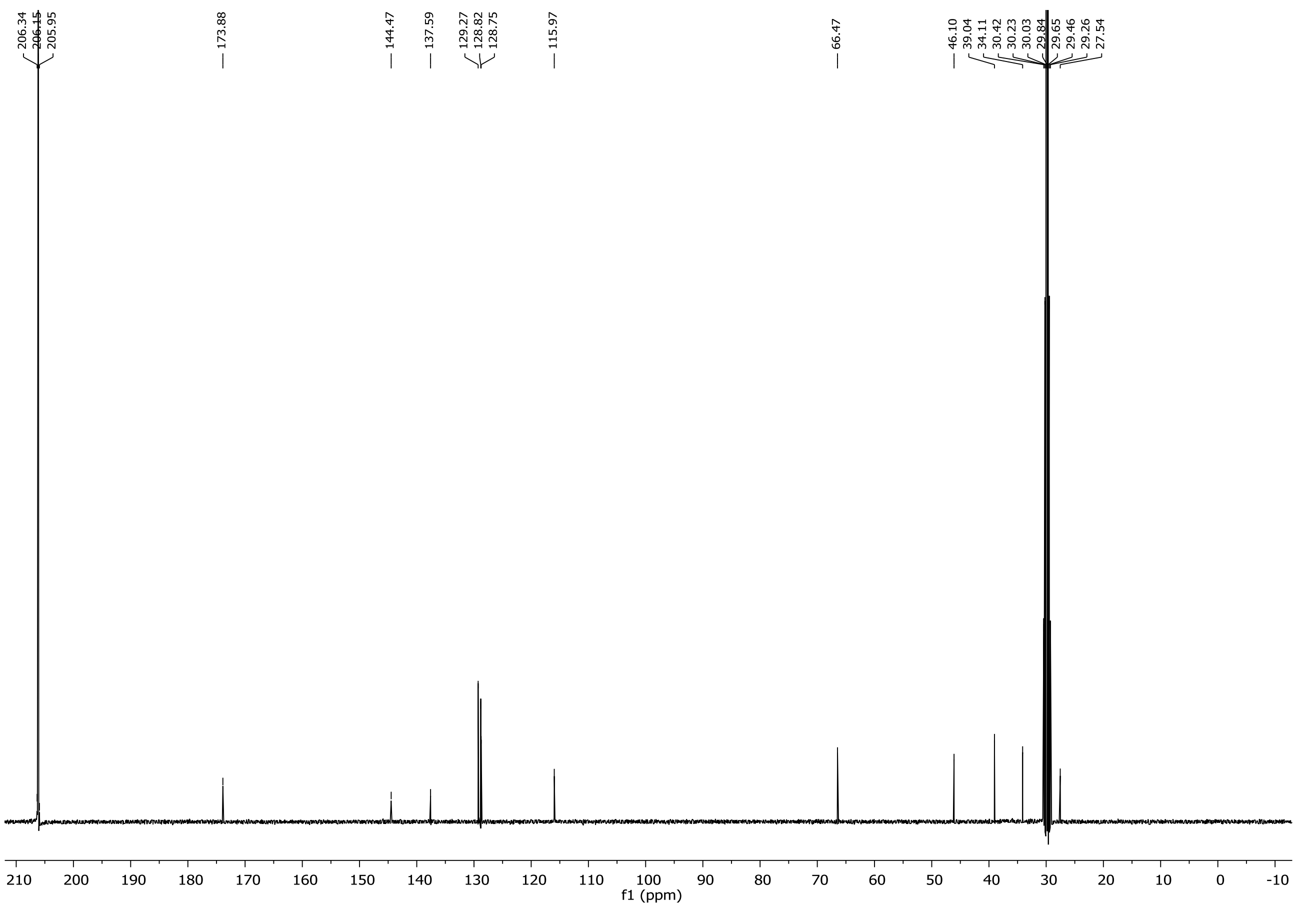
206.34
206.15
205.95
173.88
144.47
137.59
129.27
128.82
128.75
115.97
66.47
46.10
39.04
34.11
30.42
30.23
30.03
29.84
29.65
29.46
29.26
27.54
210 200 190 180 170 160 150 140 130 120 110 100 90 80 70 60 50 40 30 20 10 0 -10
f1 (ppm)

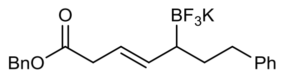
O
BF3K
BnO
Ph

2e

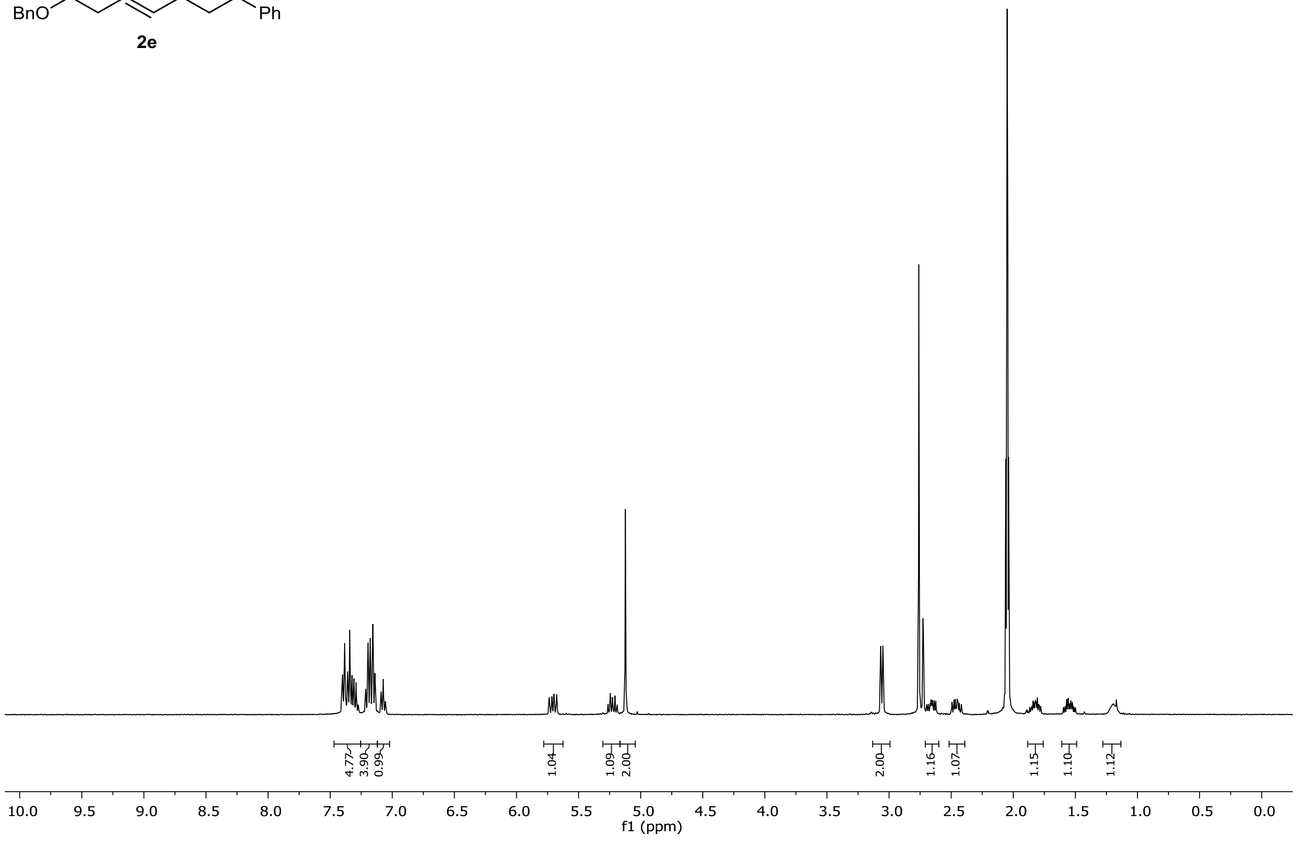
4.77
3.90
0.99
1.04
1.09
2.00
2.00
1.16
1.07
1.15
1.10
1.12
10.0 9.5 9.0 8.5 8.0 7.5 7.0 6.5 6.0 5.5 5.0 4.5 4.0 3.5 3.0 2.5 2.0 1.5 1.0 0.5 0.0
f1 (ppm)

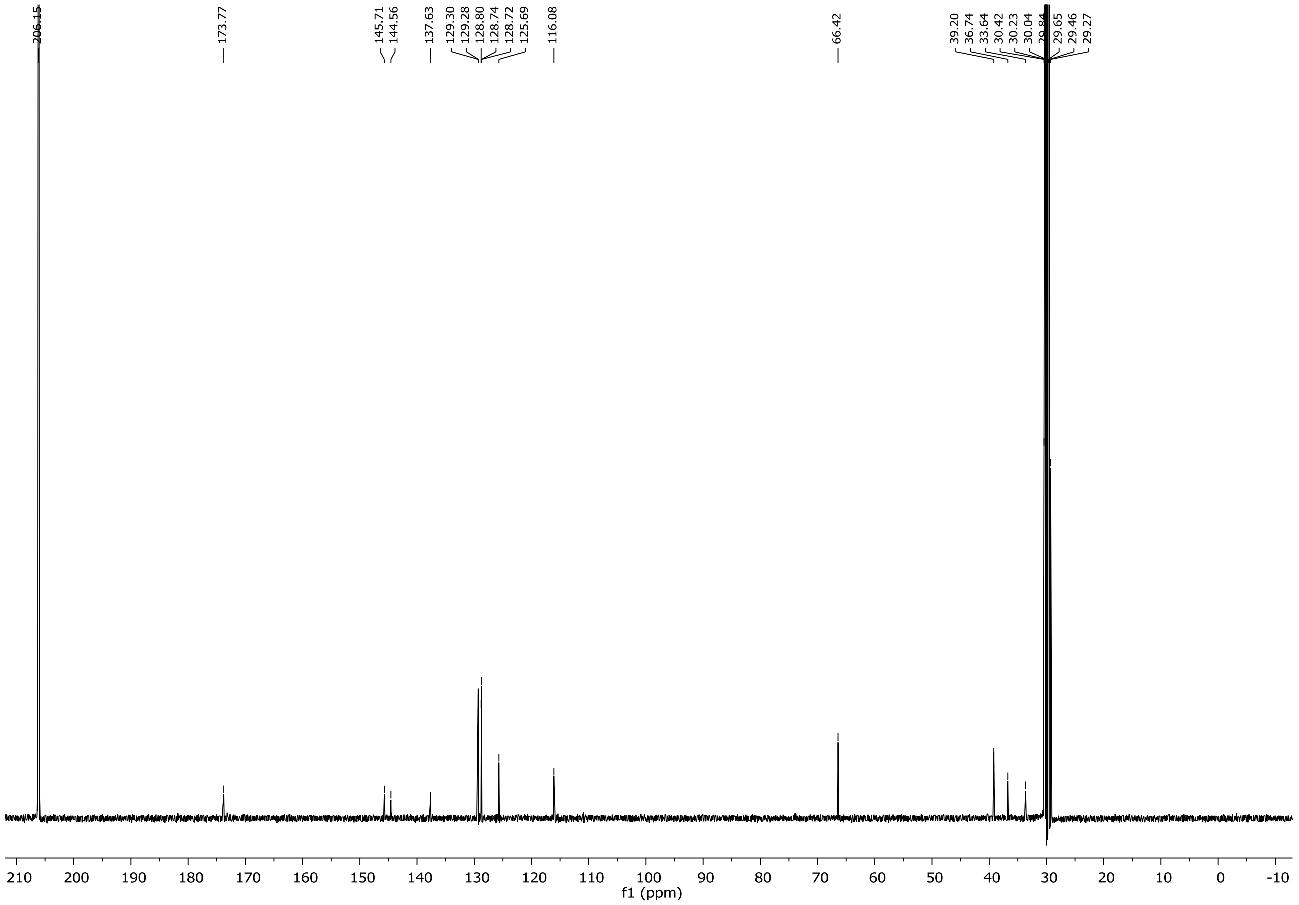
206.15
173.77
145.71
144.56
137.63
129.30
129.28
128.80
128.74
128.72
125.69
116.08
66.42
39.20
36.74
33.64
30.42
30.23
30.04
29.84
29.65
29.46
29.27
210 200 190 180 170 160 150 140 130 120 110 100 90 80 70 60 50 40 30 20 10 0 -10
f1 (ppm)

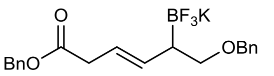
O
BF3K
BnO
OBn

2f

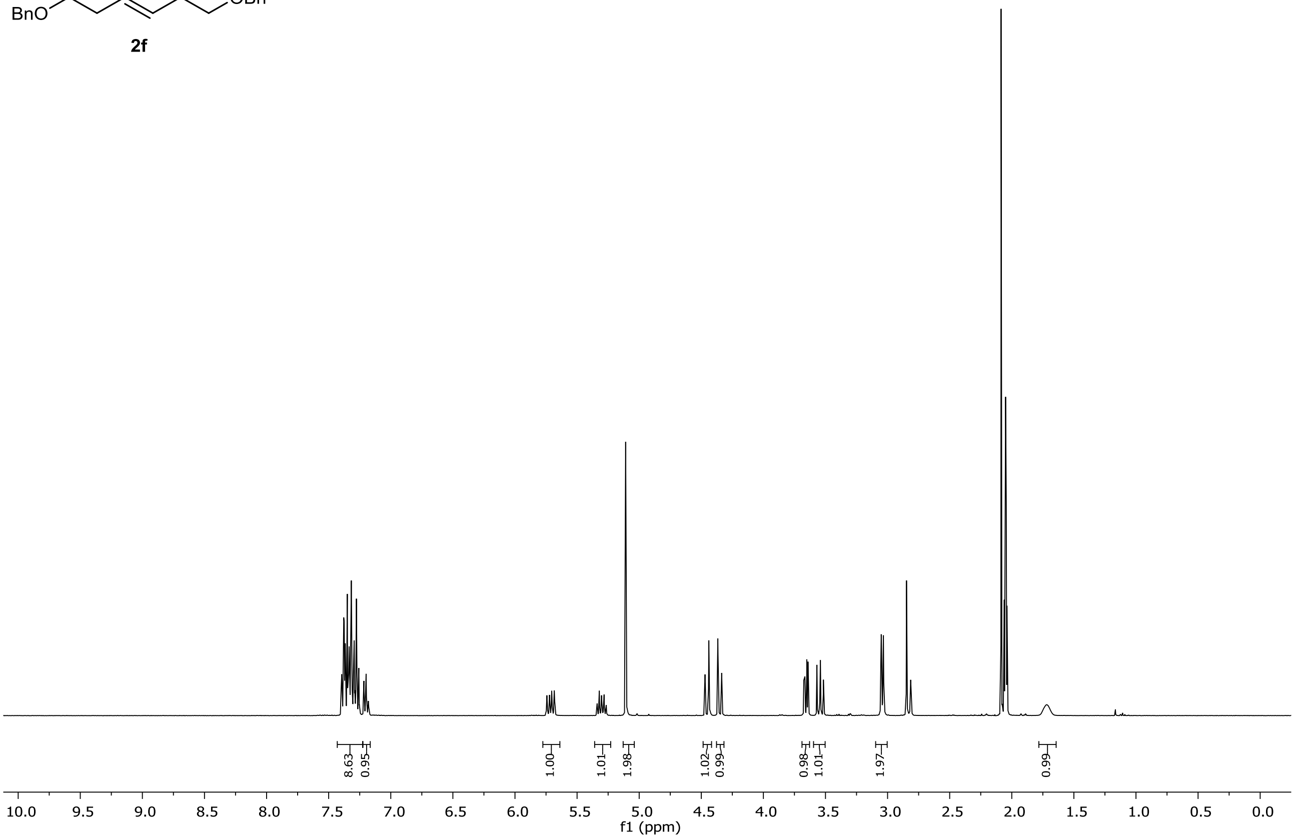
8.63
0.95
1.00
1.01
1.98
1.02
0.99
0.98
1.01
1.97
0.99
10.0 9.5 9.0 8.5 8.0 7.5 7.0 6.5 6.0 5.5 5.0 4.5 4.0 3.5 3.0 2.5 2.0 1.5 1.0 0.5 0.0
f1 (ppm)

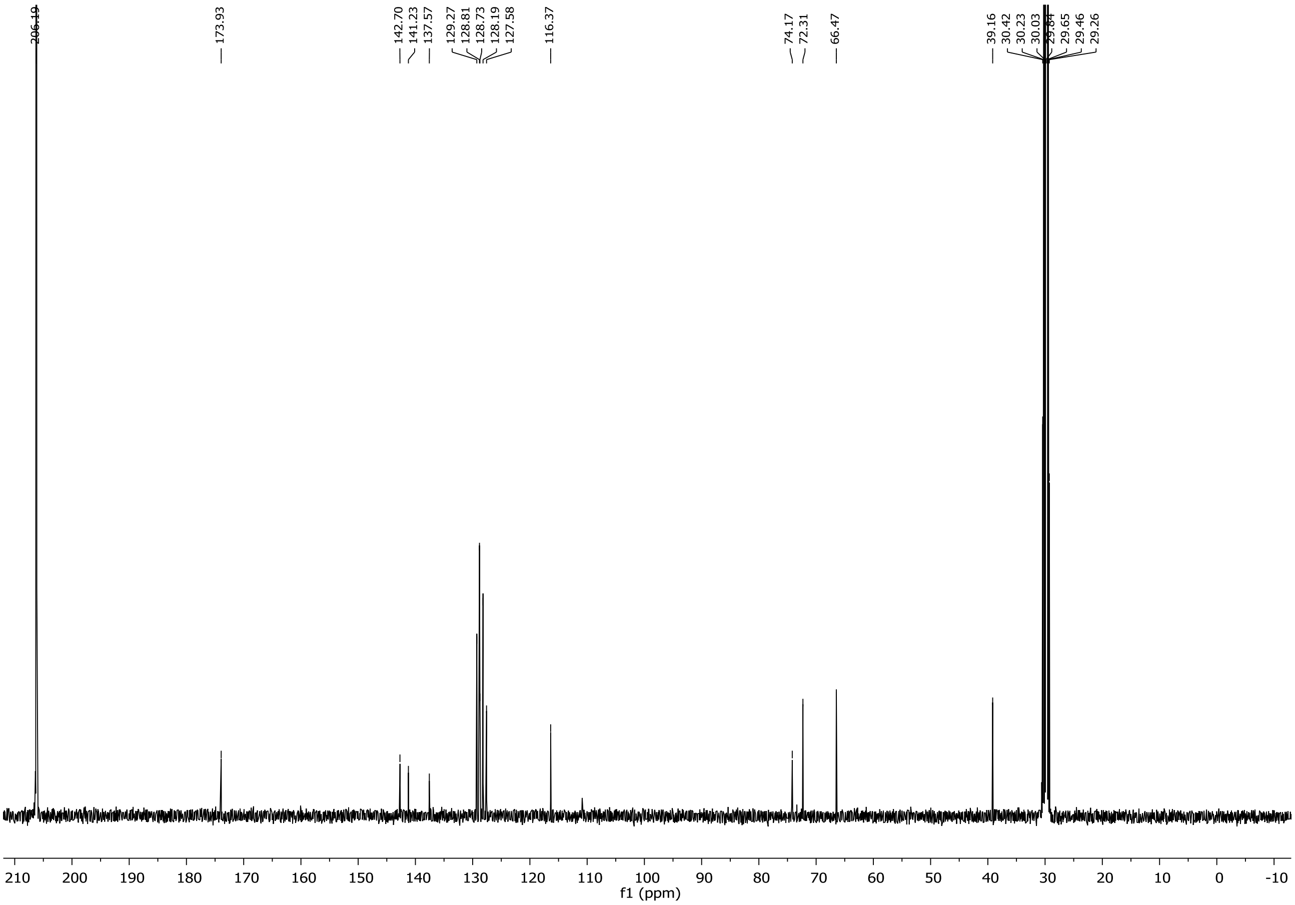
206.19
173.93
142.70
141.23
137.57
129.27
128.81
128.73
128.19
127.58
116.37
74.17
72.31
66.47
39.16
30.42
30.23
30.03
29.84
29.65
29.46
29.26
210 200 190 180 170 160 150 140 130 120 110 100 90 80 70 60 50 40 30 20 10 0 -10
f1 (ppm)

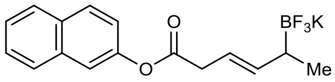
BF3K
O
O
Me

2g

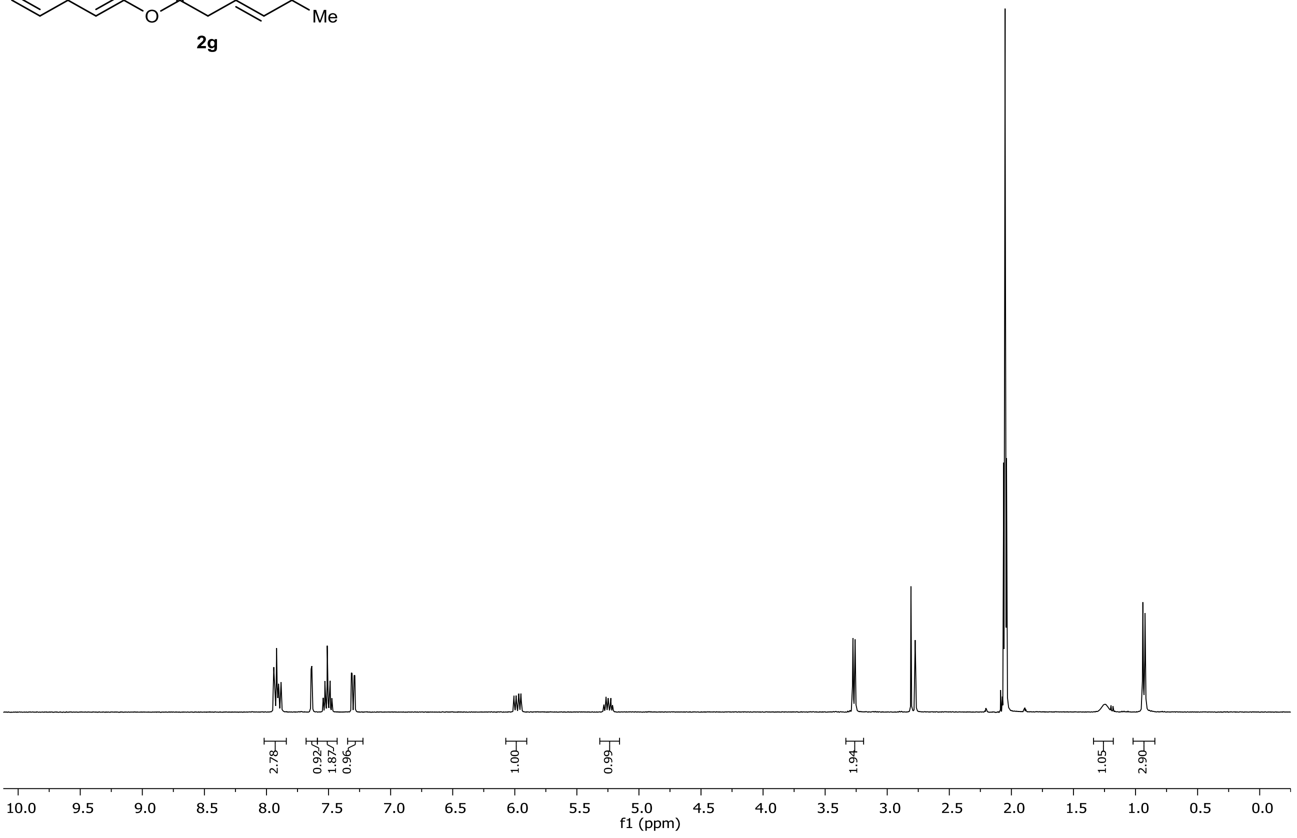
2.78
0.92
1.87
0.96
1.00
0.99
1.94
1.05
2.90
10.0 9.5 9.0 8.5 8.0 7.5 7.0 6.5 6.0 5.5 5.0 4.5 4.0 3.5 3.0 2.5 2.0 1.5 1.0 0.5 0.0
f1 (ppm)

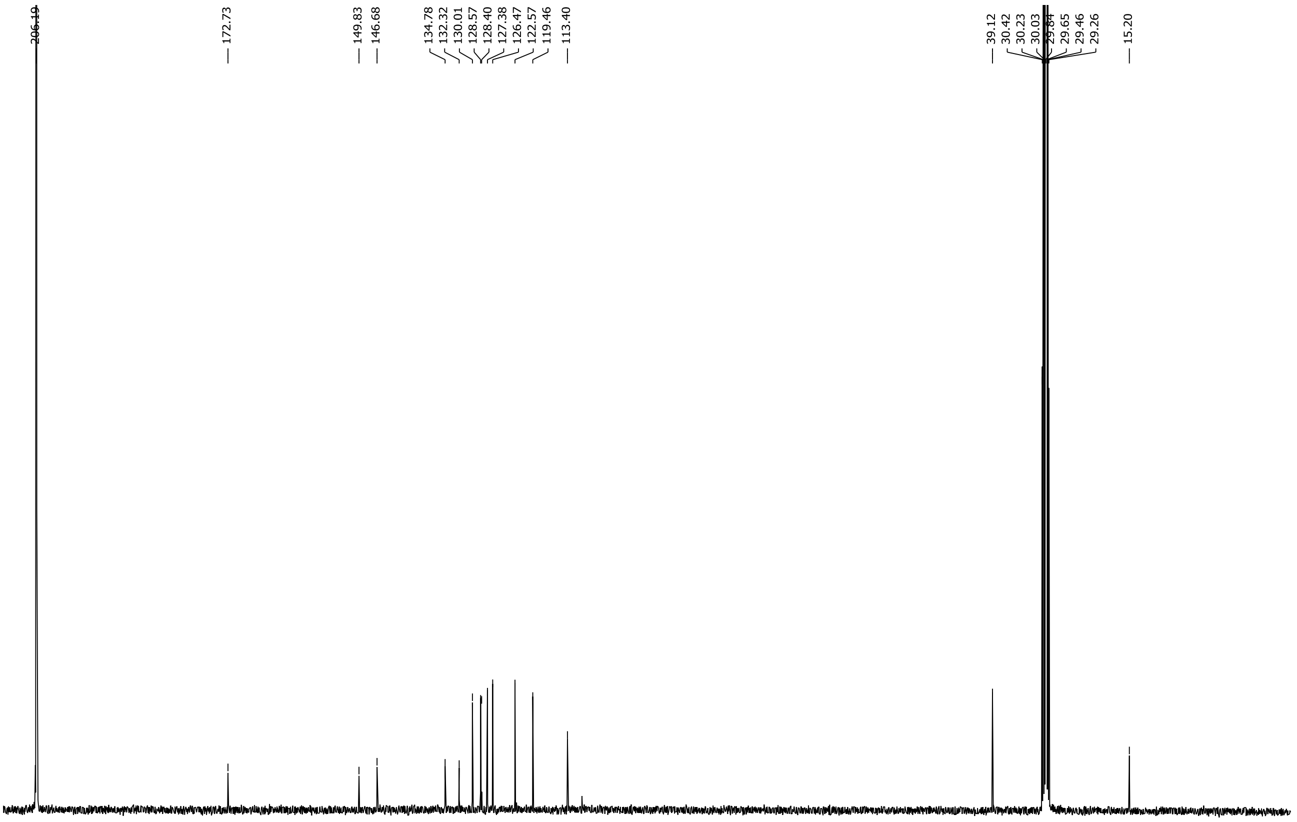
206.19
172.73
149.83
146.68
134.78
132.32
130.01
128.57
128.40
127.38
126.47
122.57
119.46
113.40
39.12
30.42
30.23
30.03
29.84
29.65
29.46
29.26
15.20
210 200 190 180 170 160 150 140 130 120 110 100 90 80 70 60 50 40 30 20 10 0 -10
f1 (ppm)

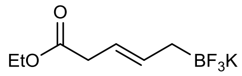
EtO
BF3K

2h

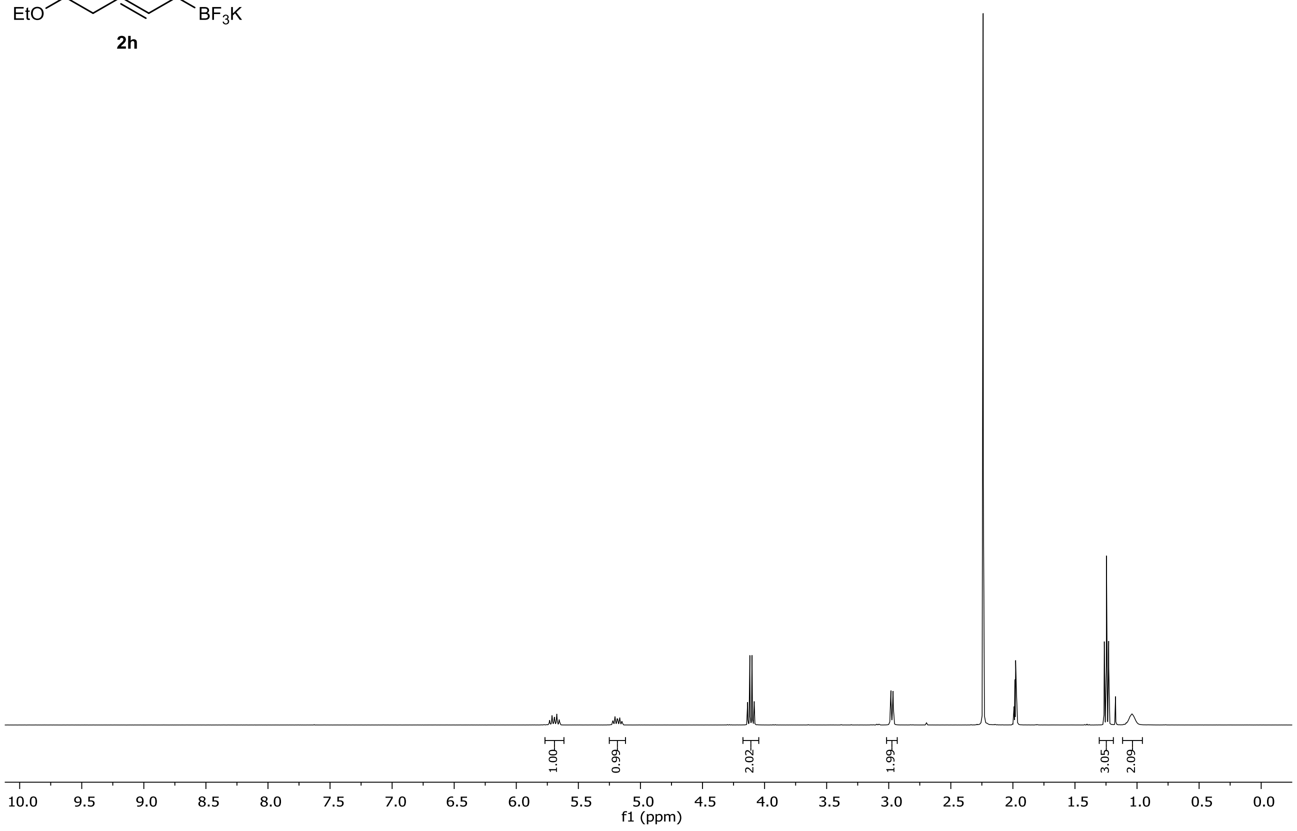
1.00
0.99
2.02
1.99
3.05
2.09
10.0 9.5 9.0 8.5 8.0 7.5 7.0 6.5 6.0 5.5 5.0 4.5 4.0 3.5 3.0 2.5 2.0 1.5 1.0 0.5 0.0
f1 (ppm)

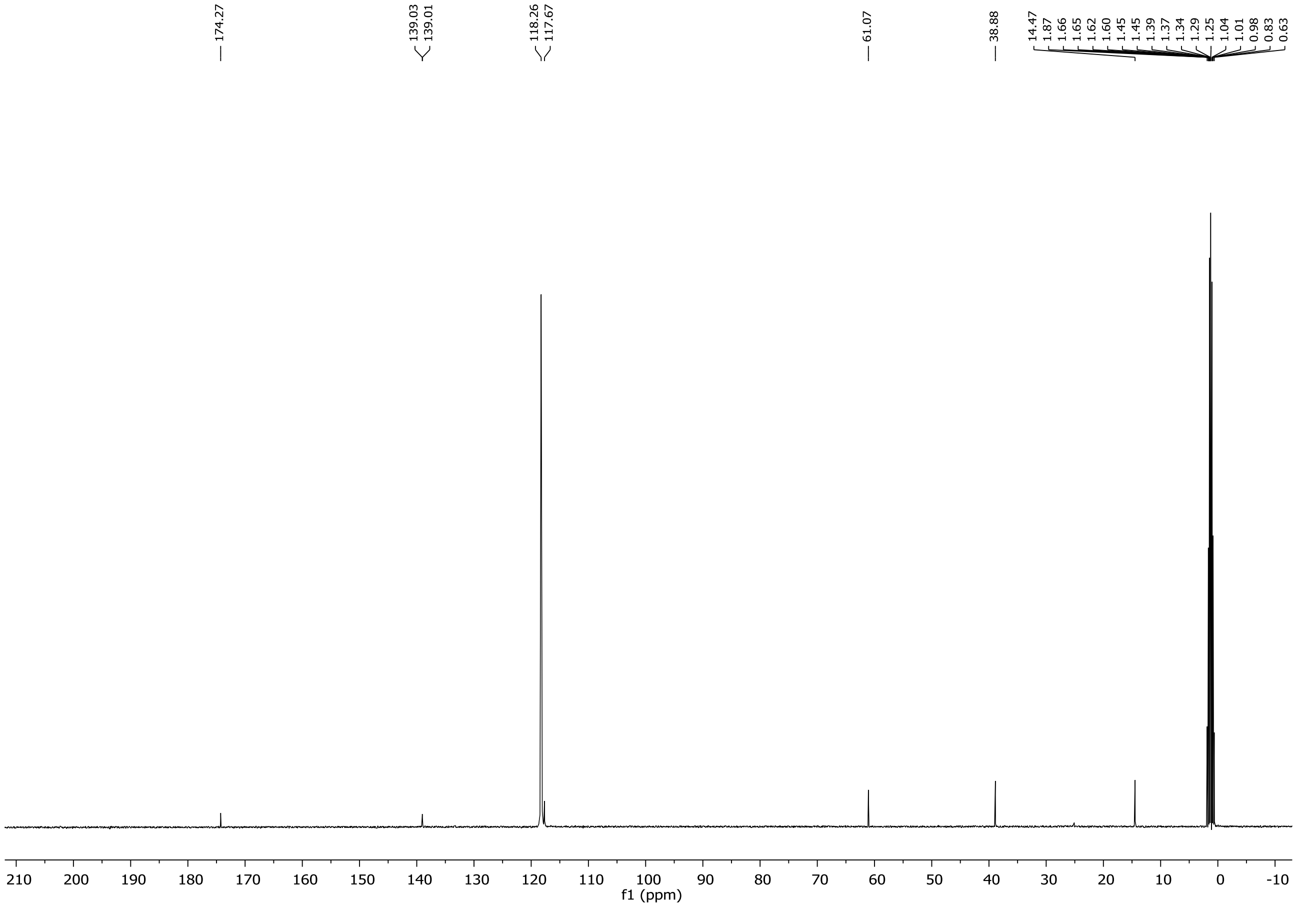
174.27
139.03
139.01
118.26
117.67
61.07
38.88
14.47
1.87
1.66
1.65
1.62
1.60
1.45
1.45
1.39
1.37
1.34
1.29
1.25
1.04
1.01
0.98
0.83
0.63
210 200 190 180 170 160 150 140 130 120 110 100 90 80 70 60 50 40 30 20 10 0 -10
f1 (ppm)

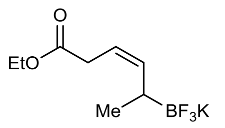
O
EtO
Me
BF3K

8

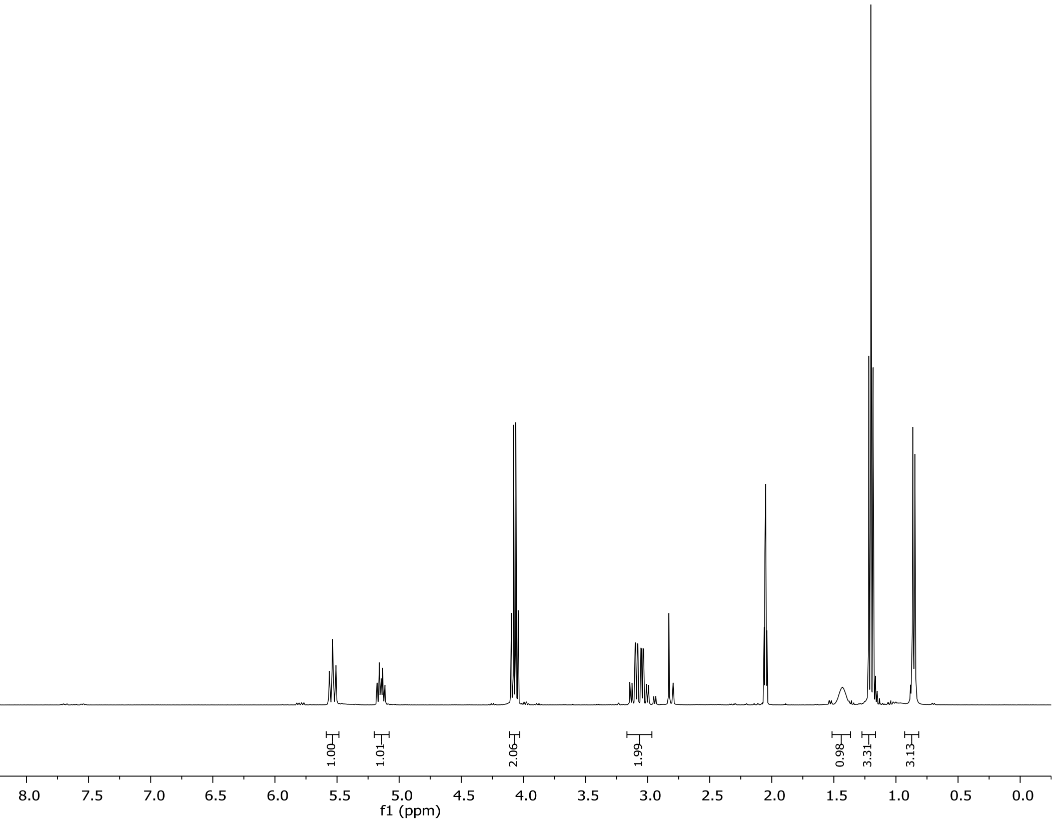
1.00
1.01
2.06
1.99
0.98
3.31
3.13
10.0 9.5 9.0 8.5 8.0 7.5 7.0 6.5 6.0 5.5 5.0 4.5 4.0 3.5 3.0 2.5 2.0 1.5 1.0 0.5 0.0
f1 (ppm)

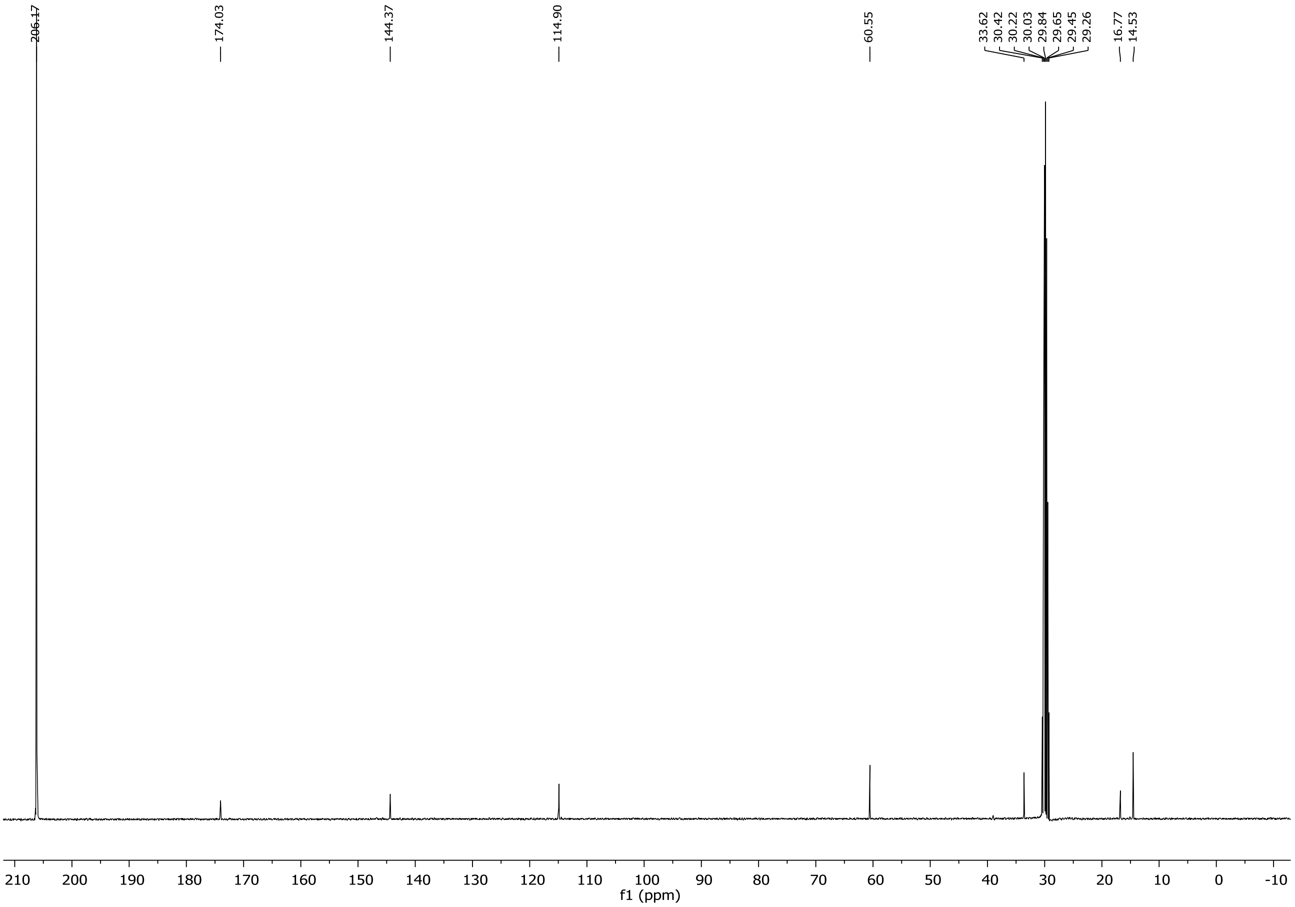
206.17
174.03
144.37
114.90
60.55
33.62
30.42
30.22
30.03
29.84
29.65
29.45
29.26
16.77
14.53
210 200 190 180 170 160 150 140 130 120 110 100 90 80 70 60 50 40 30 20 10 0 -10
f1 (ppm)

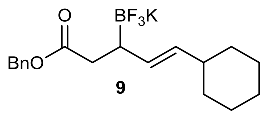
BnO
O
BF3K

9

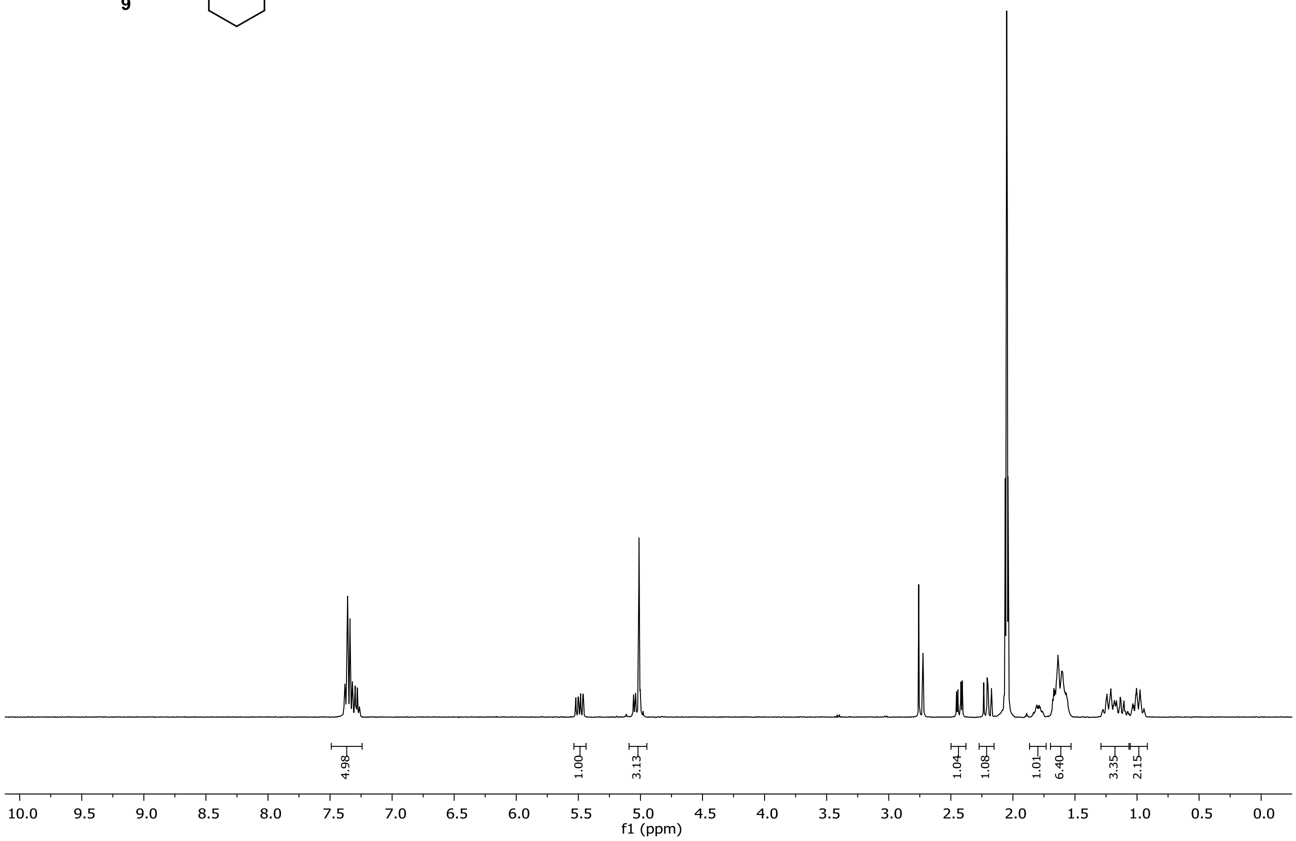
4.98
1.00
3.13
1.04
1.08
1.01
6.40
3.35
2.15
10.0 9.5 9.0 8.5 8.0 7.5 7.0 6.5 6.0 5.5 5.0 4.5 4.0 3.5 3.0 2.5 2.0 1.5 1.0 0.5 0.0
f1 (ppm)

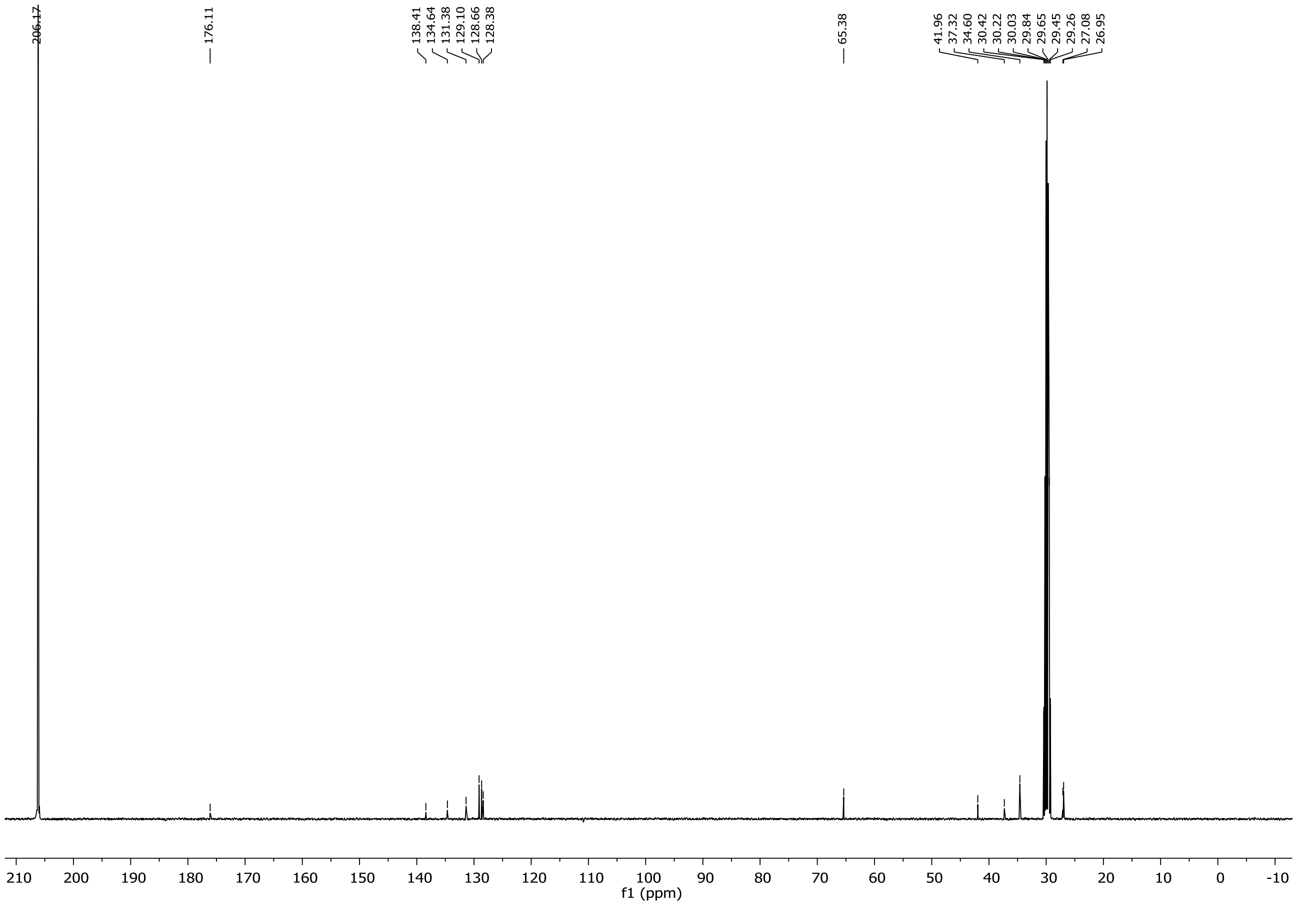
206.17
176.11
138.41
134.64
131.38
129.10
128.66
128.38
65.38
41.96
37.32
34.60
30.42
30.22
30.03
29.84
29.65
29.45
29.26
27.08
26.95
210 200 190 180 170 160 150 140 130 120 110 100 90 80 70 60 50 40 30 20 10 0 -10
f1 (ppm)

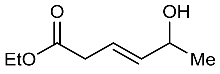

S13 (and **S14**)

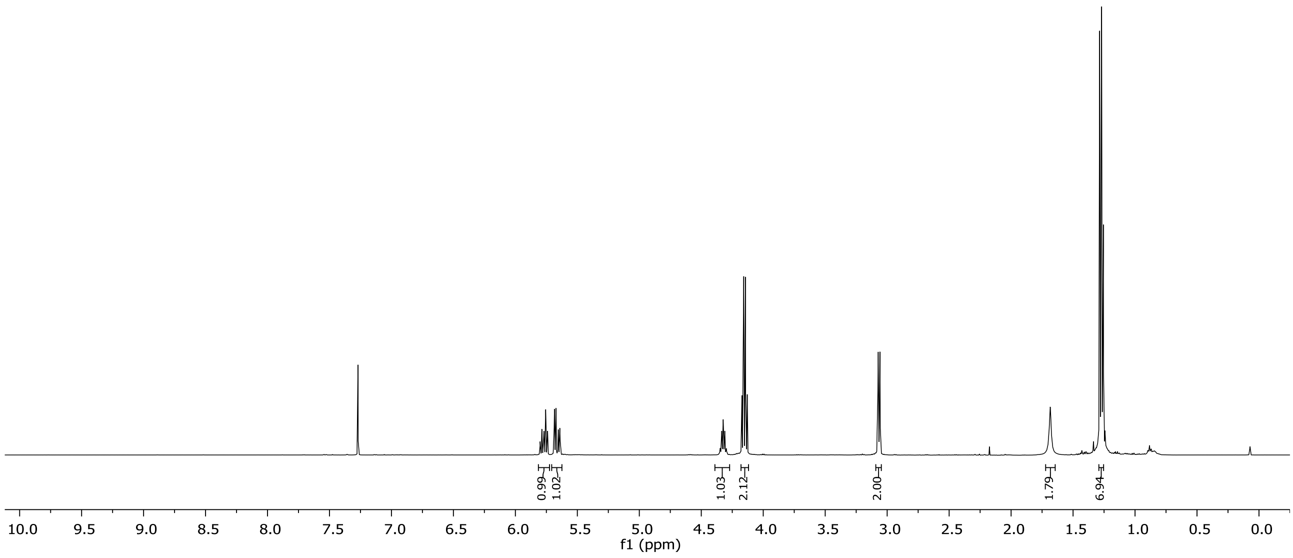

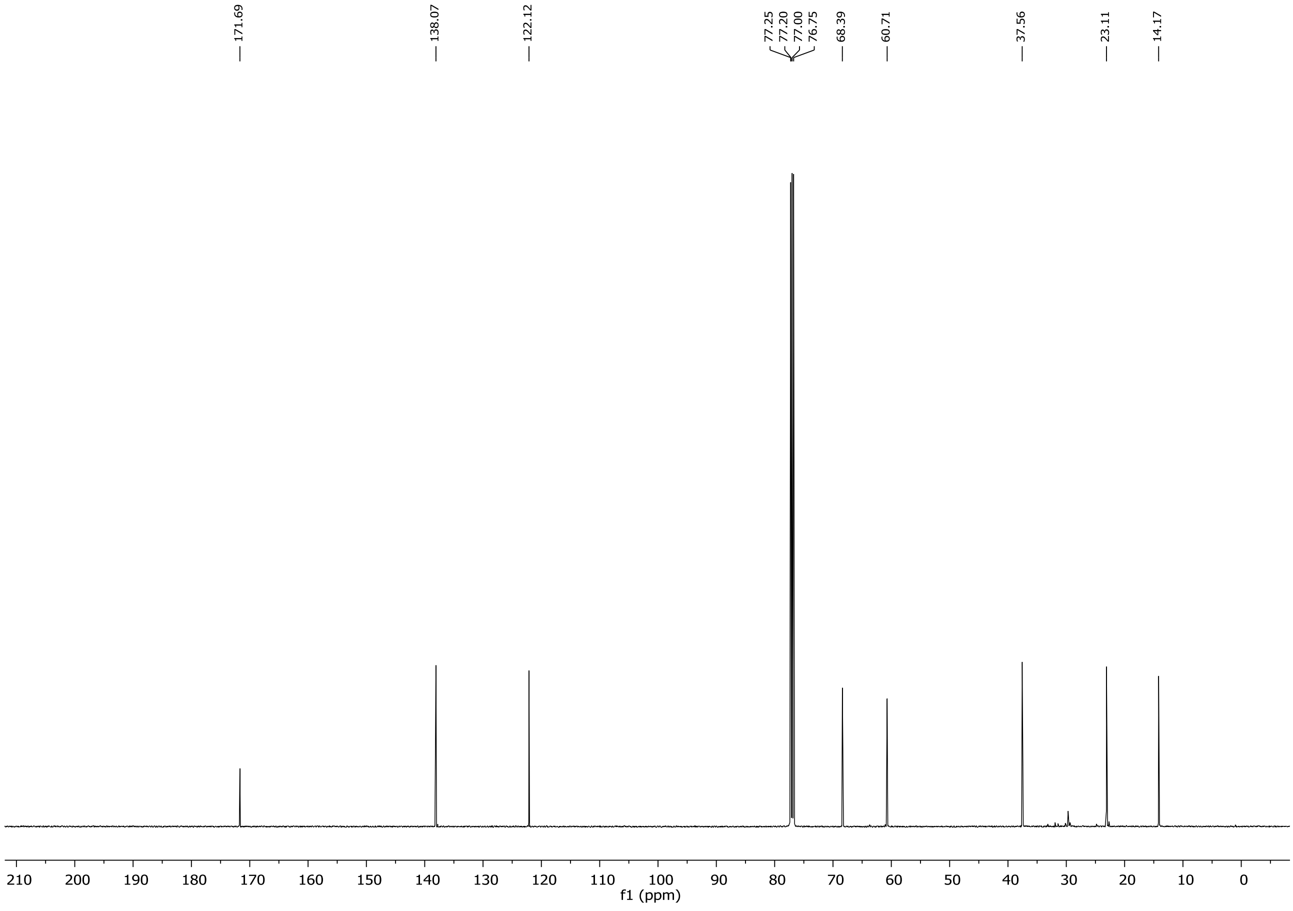

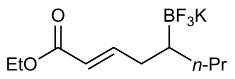

24

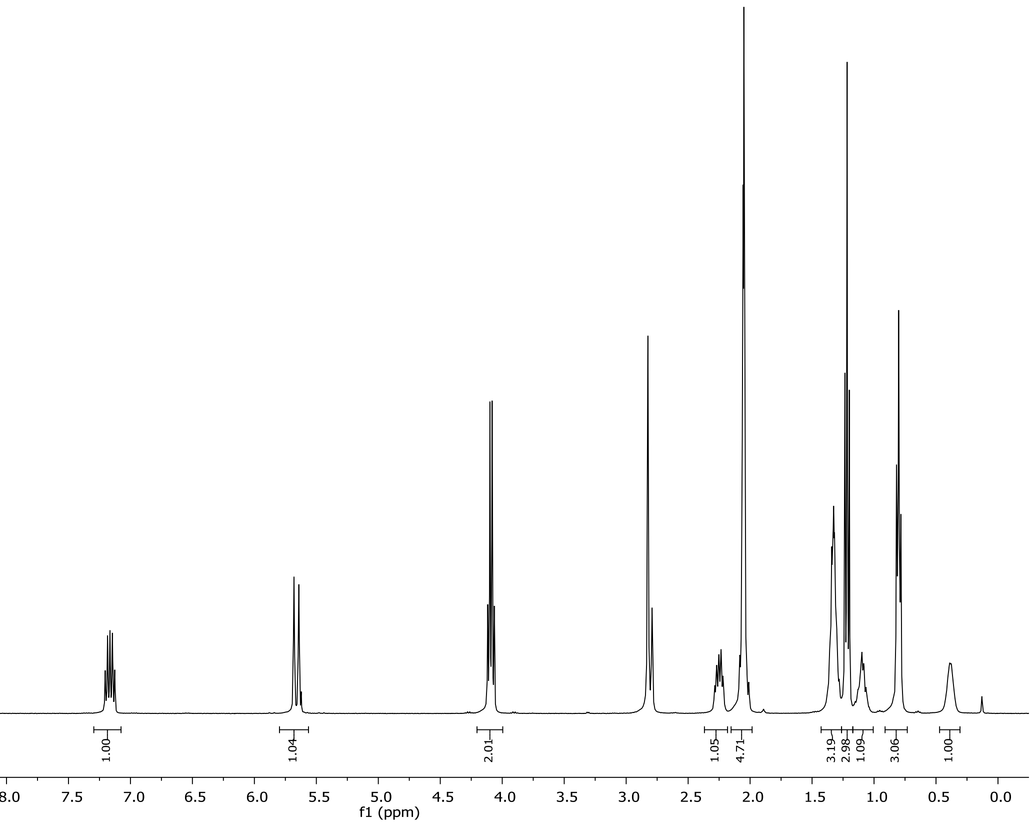
1.00
1.04
2.01
1.05
4.71
3.19
2.98
1.09
3.06
1.00
10.0 9.5 9.0 8.5 8.0 7.5 7.0 6.5 6.0 5.5 5.0 4.5 4.0 3.5 3.0 2.5 2.0 1.5 1.0 0.5 0.0
f1 (ppm)

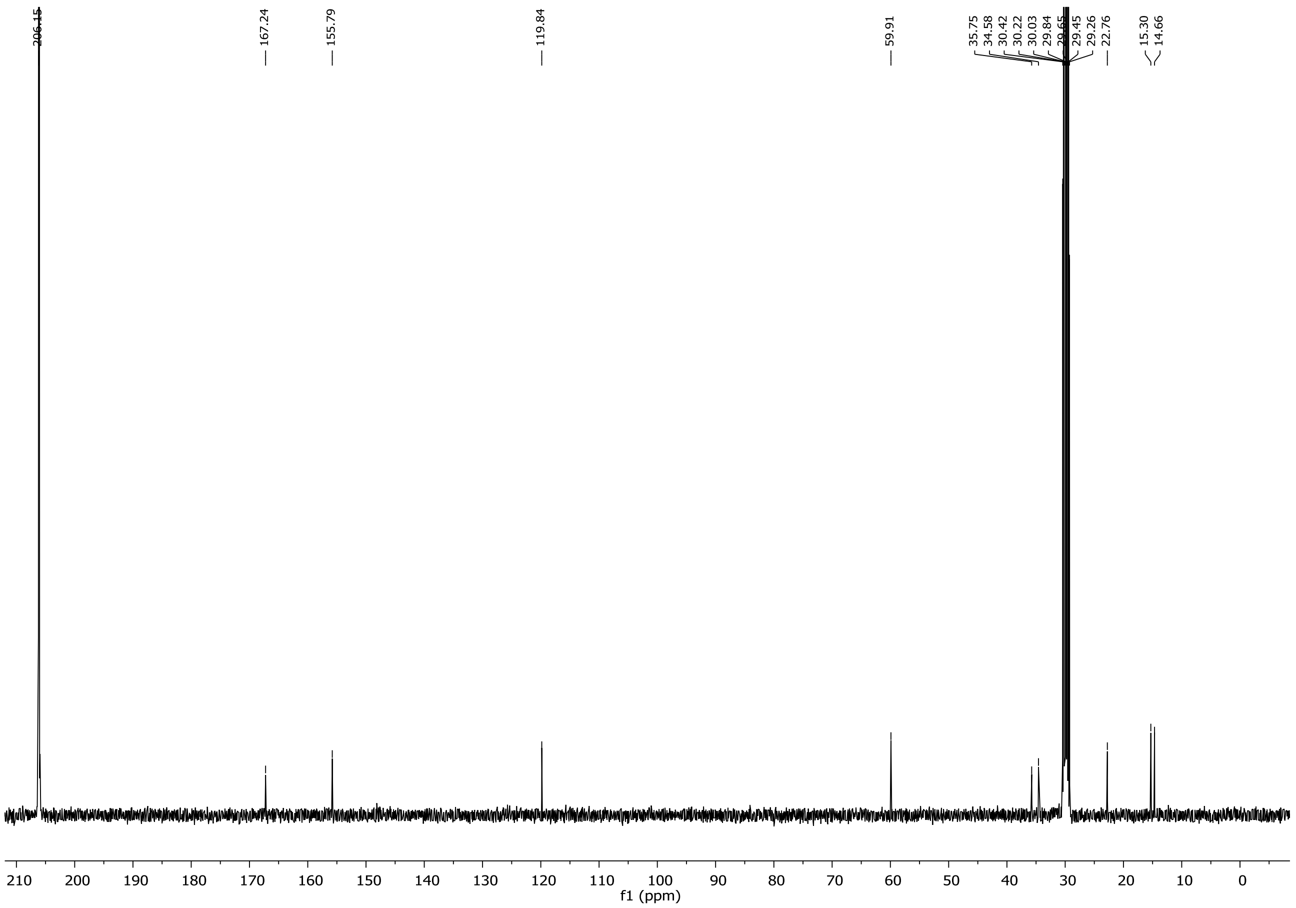
206.15
167.24
155.79
119.84
59.91
35.75
34.58
30.42
30.22
30.03
29.84
29.65
29.45
29.26
22.76
15.30
14.66
210 200 190 180 170 160 150 140 130 120 110 100 90 80 70 60 50 40 30 20 10 0
f1 (ppm)

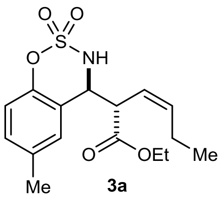
O
O
S
O
NH
O
OEt
Me
Me

3a

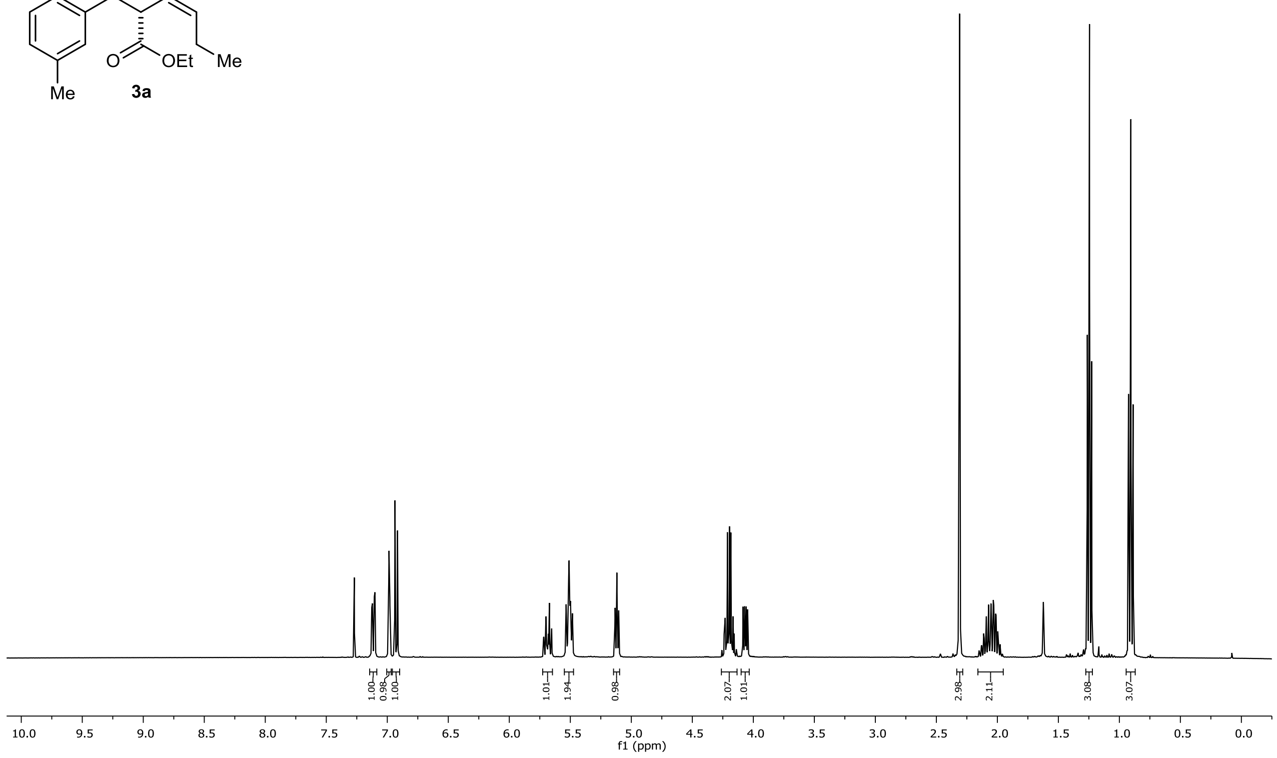
1.00
0.98
1.00
1.01
1.94
0.98
2.07
1.01
2.98
2.11
3.08
3.07
10.0 9.5 9.0 8.5 8.0 7.5 7.0 6.5 6.0 5.5 5.0 4.5 4.0 3.5 3.0 2.5 2.0 1.5 1.0 0.5 0.0
f1 (ppm)

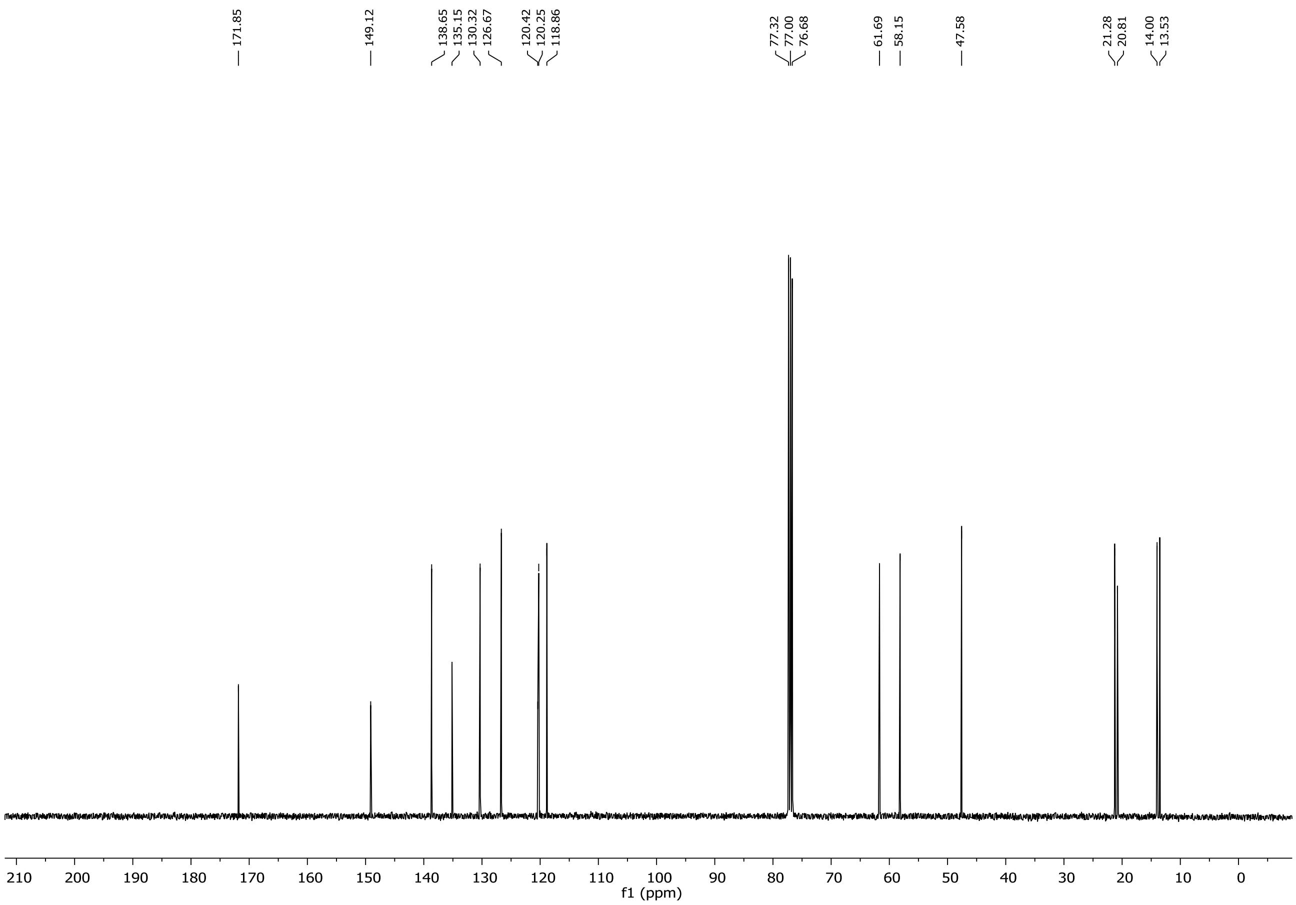
171.85
149.12
138.65
135.15
130.32
126.67
120.42
120.25
118.86
77.32
77.00
76.68
61.69
58.15
47.58
21.28
20.81
14.00
13.53
210 200 190 180 170 160 150 140 130 120 110 100 90 80 70 60 50 40 30 20 10 0
f1 (ppm)

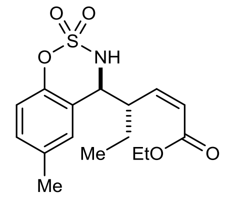
O
O
S
O
NH
Me
EtO
O
Me

4a

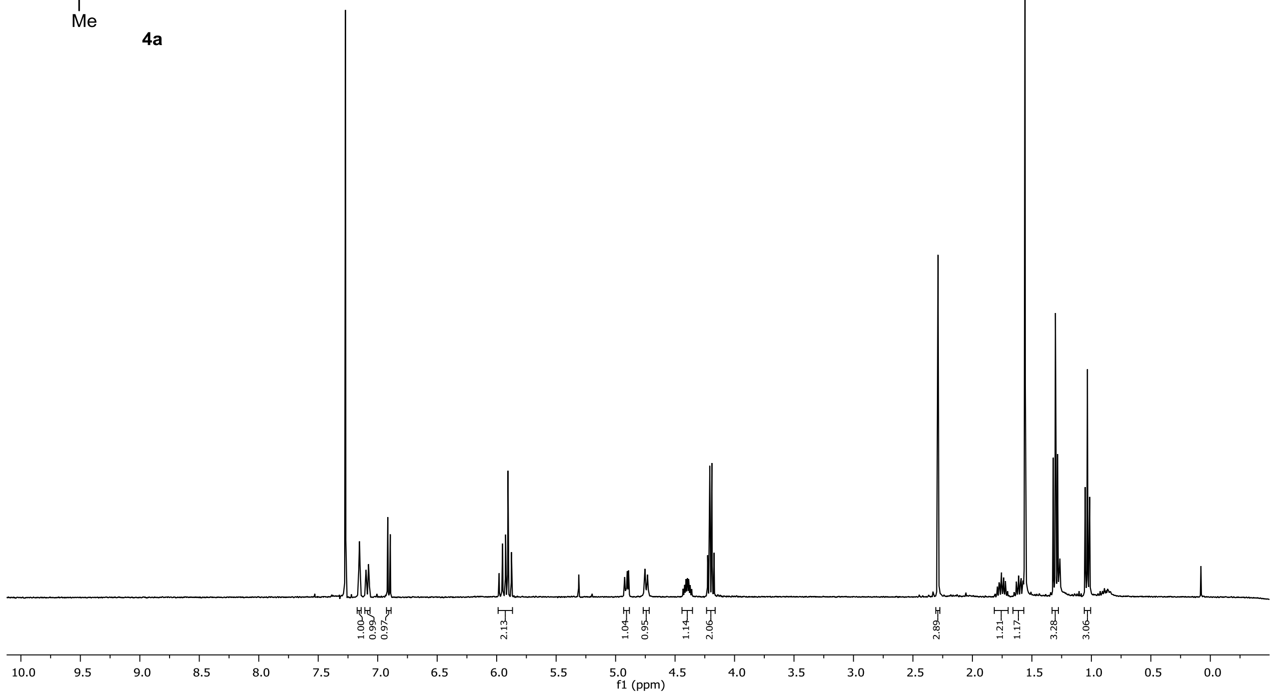
1.00
0.99
0.97
2.13
1.04
0.95
1.14
2.06
2.89
1.21
1.17
3.28
3.06
10.0 9.5 9.0 8.5 8.0 7.5 7.0 6.5 6.0 5.5 5.0 4.5 4.0 3.5 3.0 2.5 2.0 1.5 1.0 0.5 0.0
f1 (ppm)

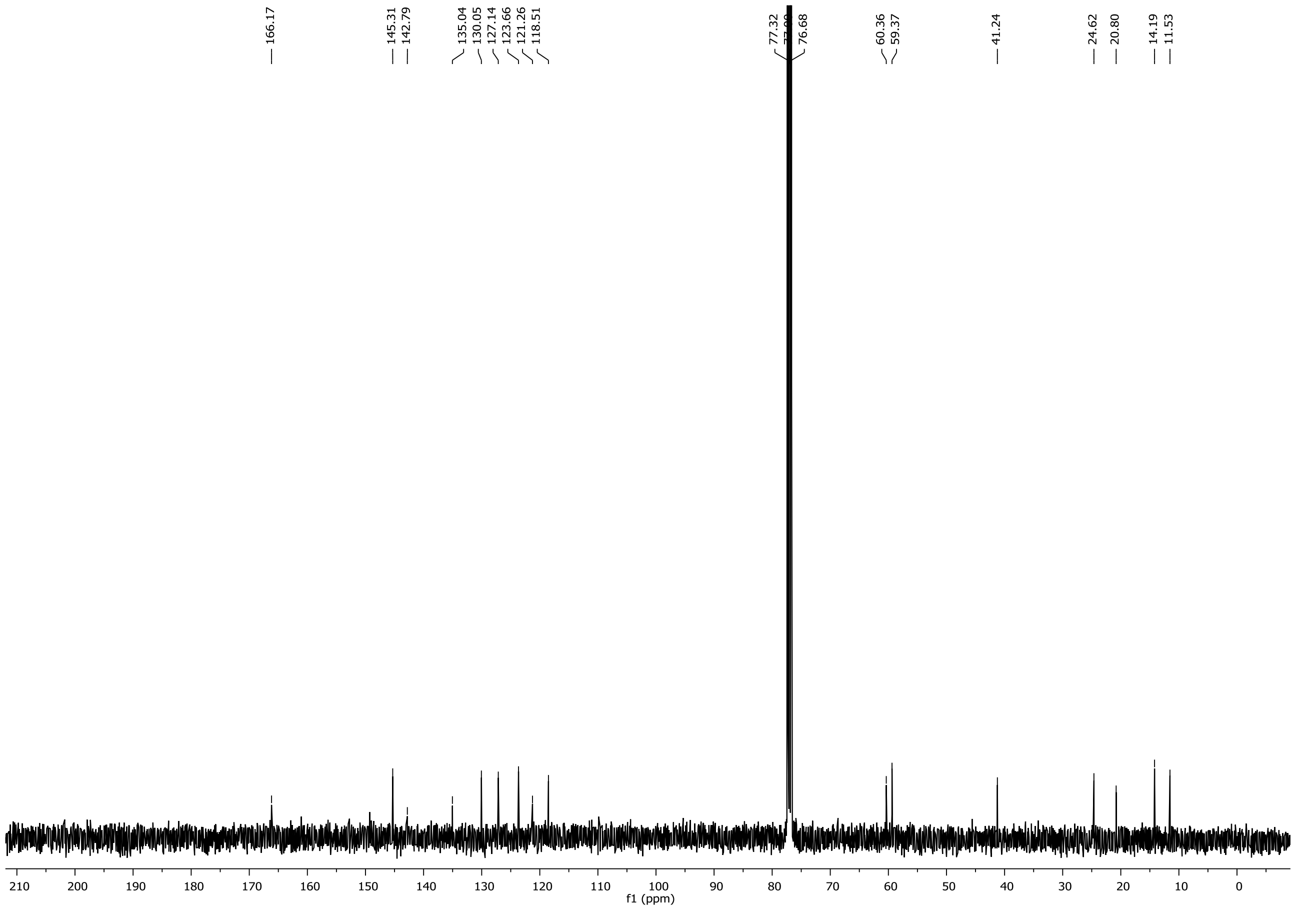
166.17
145.31
142.79
135.04
130.05
127.14
123.66
121.26
118.51
77.32
76.68
60.36
59.37
41.24
24.62
20.80
14.19
11.53
210 200 190 180 170 160 150 140 130 120 110 100 90 80 70 60 50 40 30 20 10 0
f1 (ppm)

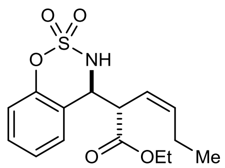
O
O
S
O
NH
O
OEt
Me

3b

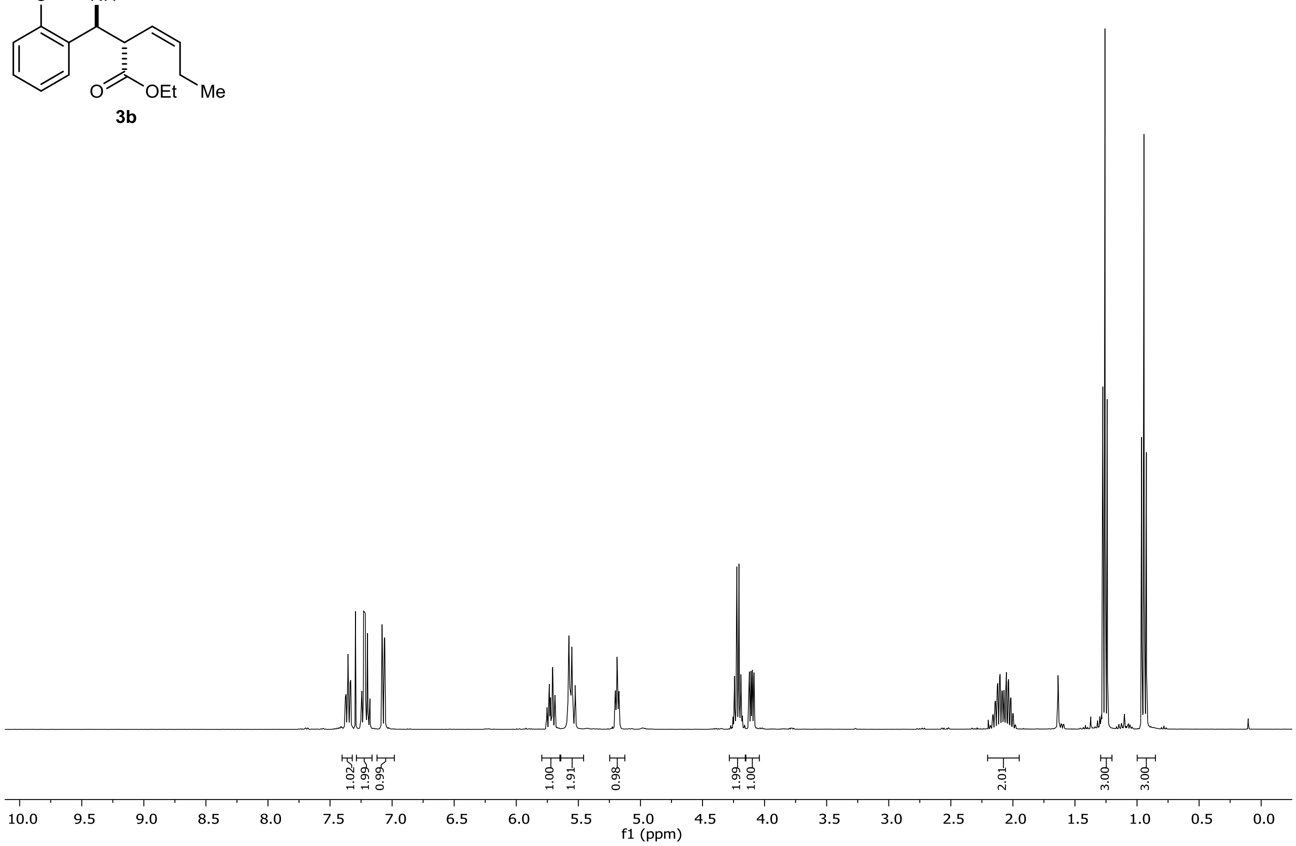
1.02
1.99
0.99
1.00
1.91
0.98
1.99
1.00
2.01
3.00
3.00
10.0 9.5 9.0 8.5 8.0 7.5 7.0 6.5 6.0 5.5 5.0 4.5 4.0 3.5 3.0 2.5 2.0 1.5 1.0 0.5 0.0
f1 (ppm)

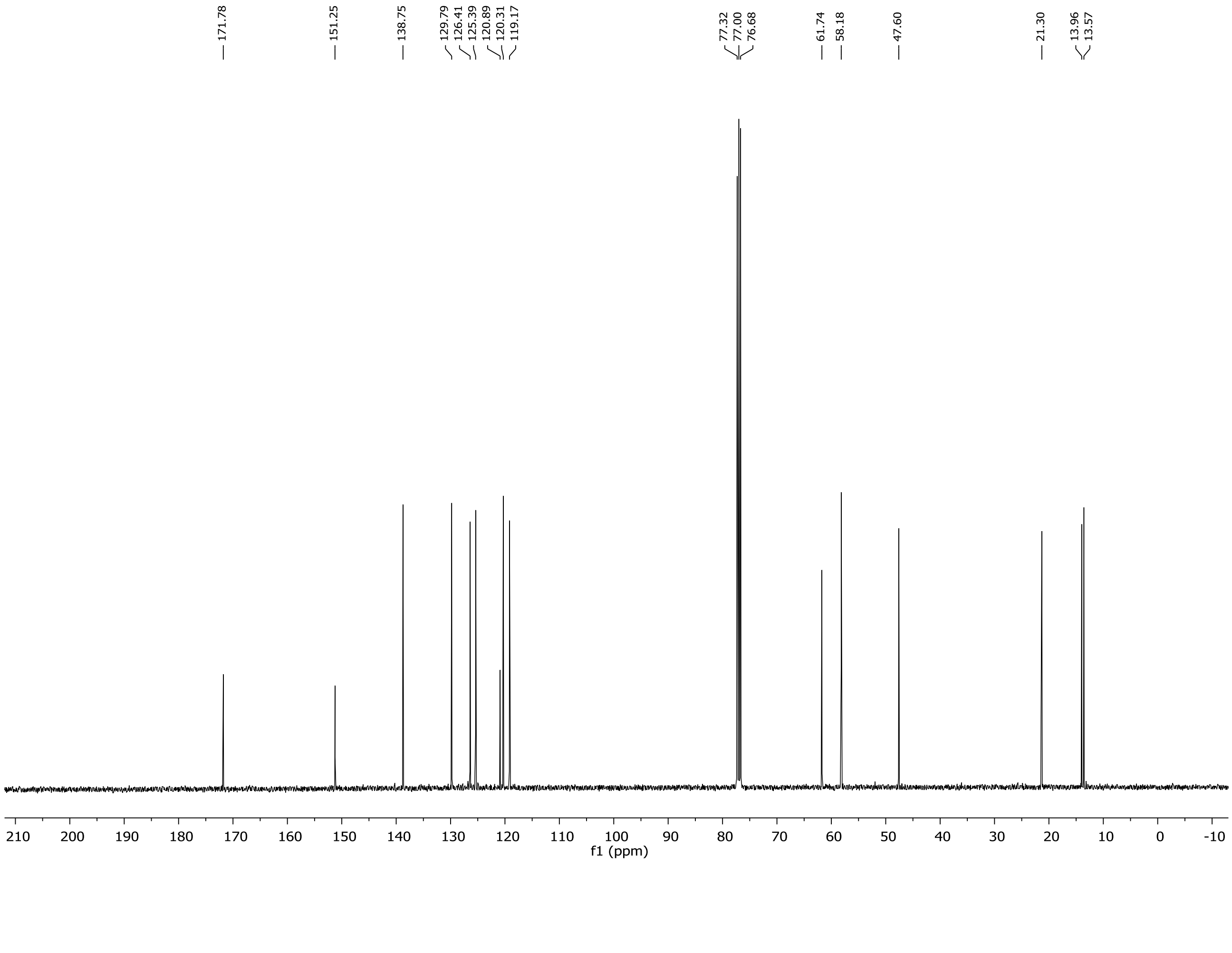
171.78
151.25
138.75
129.79
126.41
125.39
120.89
120.31
119.17
77.32
77.00
76.68
61.74
58.18
47.60
21.30
13.96
13.57
210 200 190 180 170 160 150 140 130 120 110 100 90 80 70 60 50 40 30 20 10 0 -10
f1 (ppm)

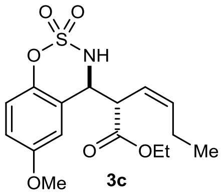
O O
S
O NH
O OEt Me
OMe

3c

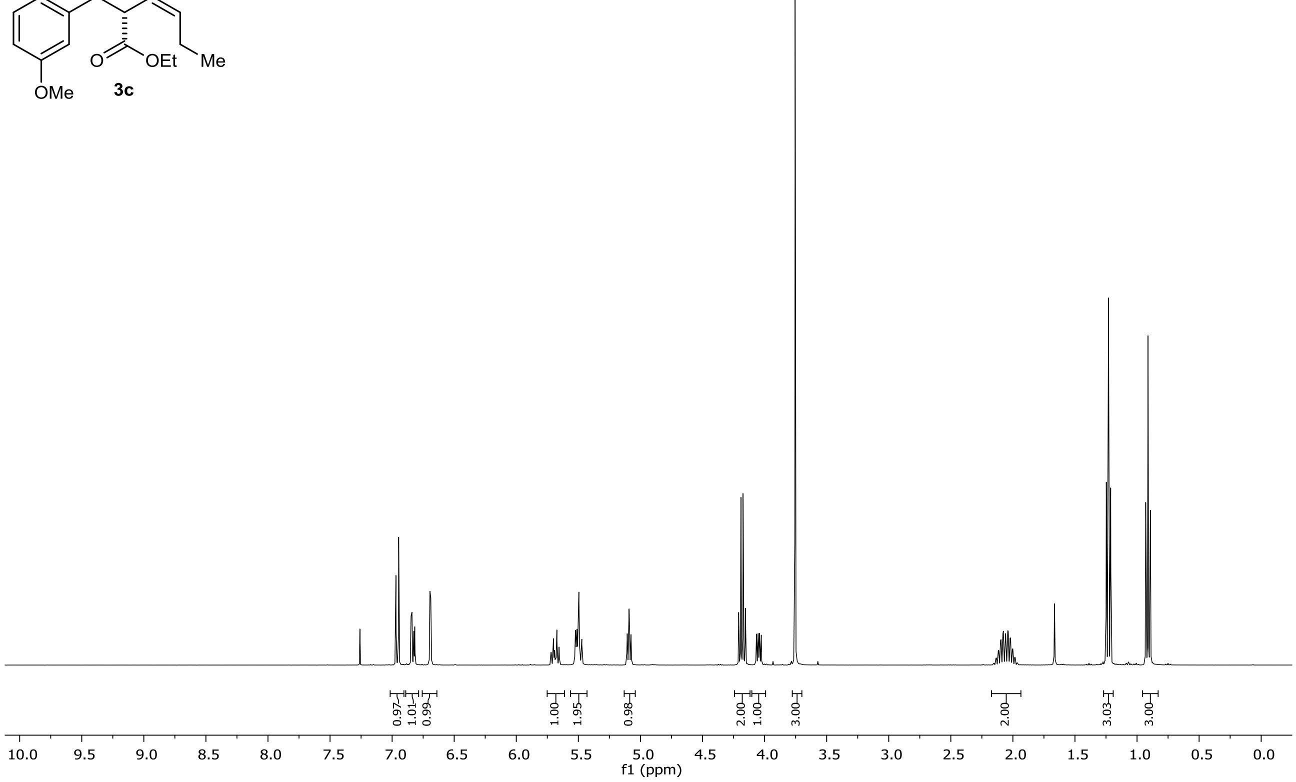
0.97
1.01
0.99
1.00
1.95
0.98
2.00
1.00
3.00
2.00
3.03
3.00
10.0 9.5 9.0 8.5 8.0 7.5 7.0 6.5 6.0 5.5 5.0 4.5 4.0 3.5 3.0 2.5 2.0 1.5 1.0 0.5 0.0
f1 (ppm)

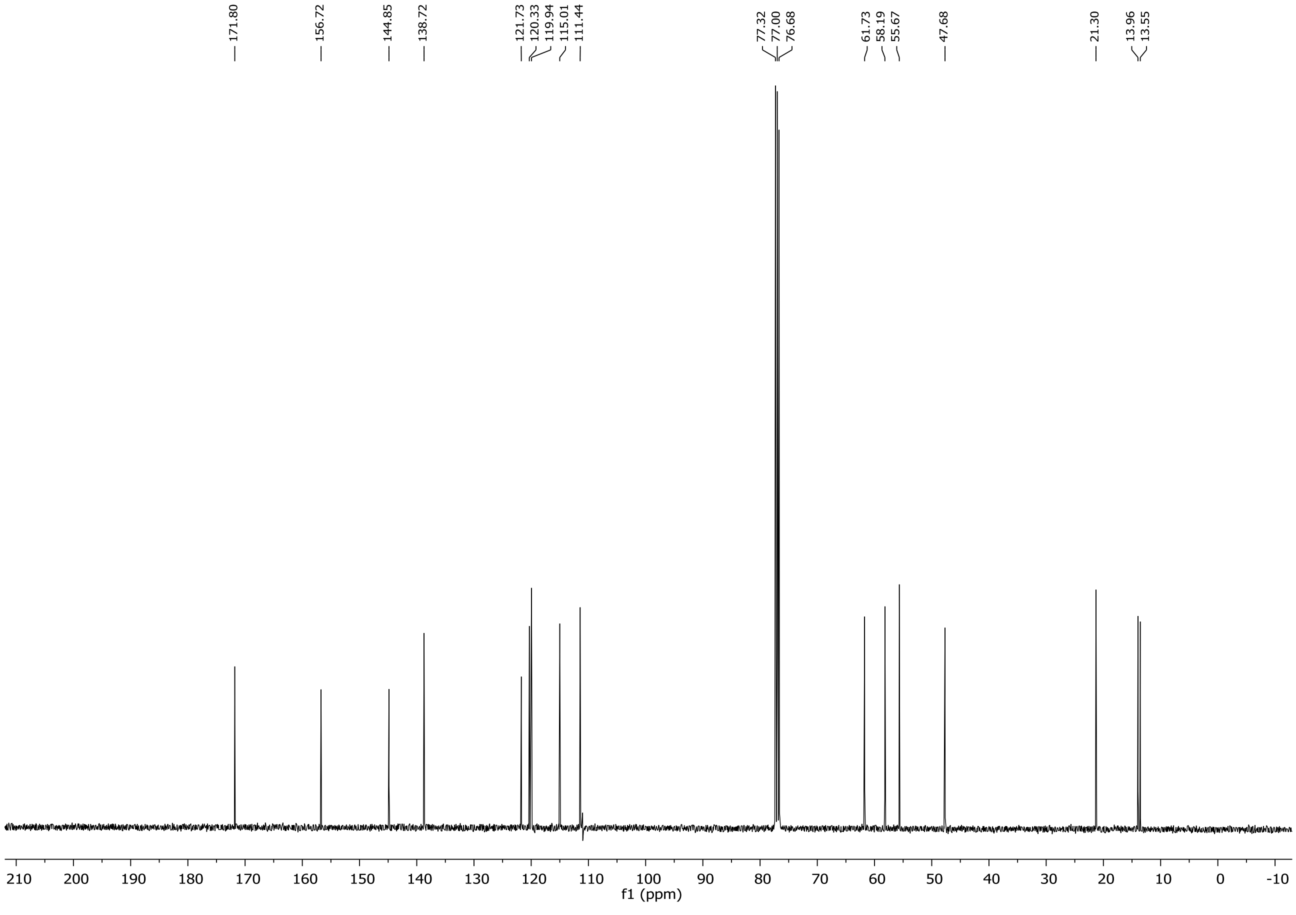
171.80
156.72
144.85
138.72
121.73
120.33
119.94
115.01
111.44
77.32
77.00
76.68
61.73
58.19
55.67
47.68
21.30
13.96
13.55
210 200 190 180 170 160 150 140 130 120 110 100 90 80 70 60 50 40 30 20 10 0 -10
f1 (ppm)

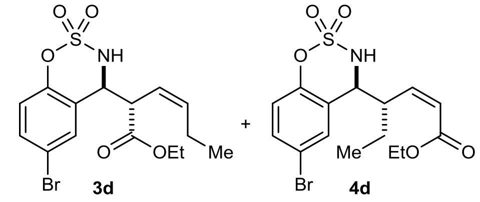

87:13 inseparable mixture

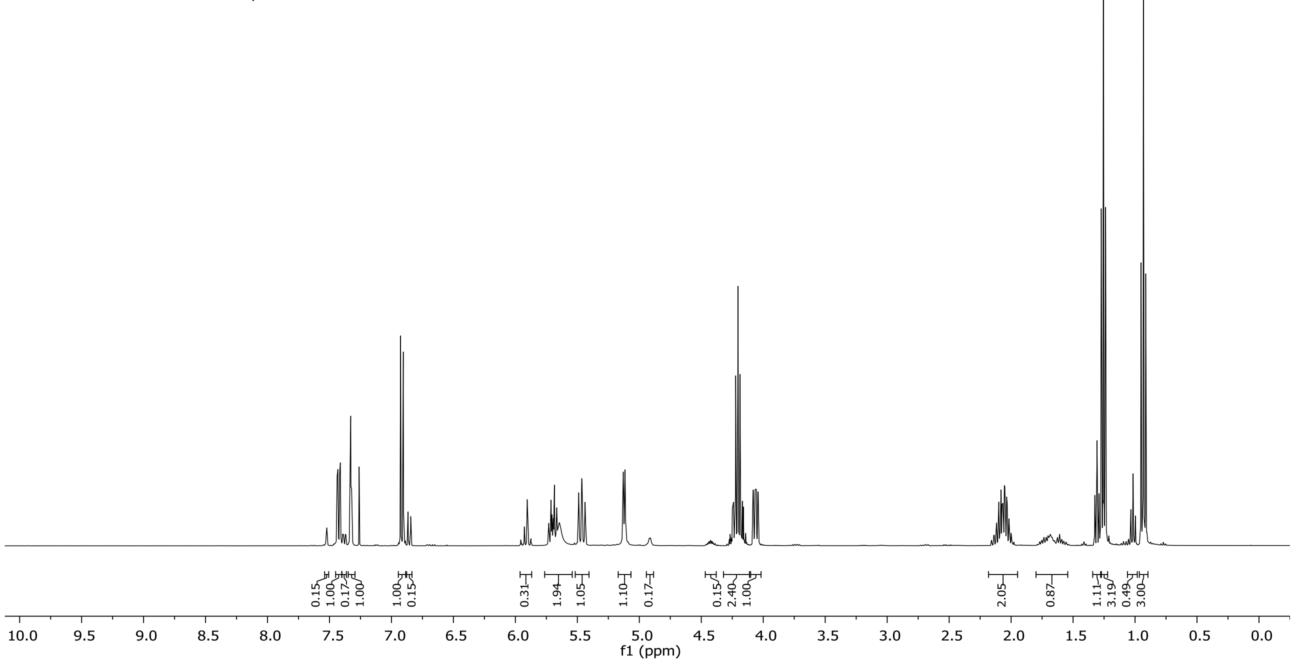

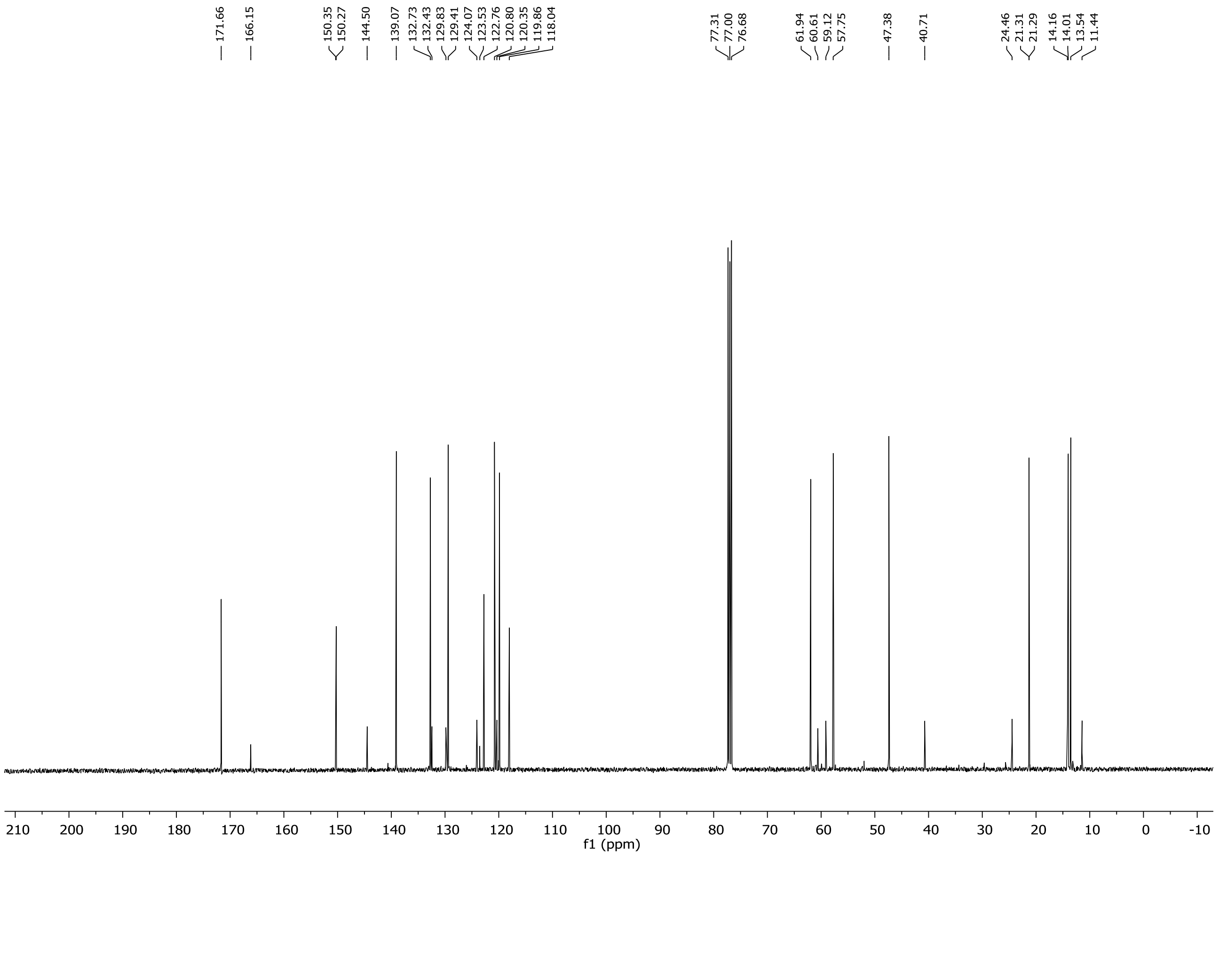

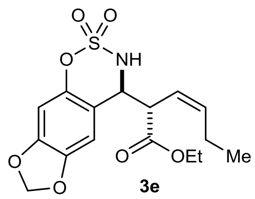
O
O
O
S
NH
O
O
O
OEt
Me

3e

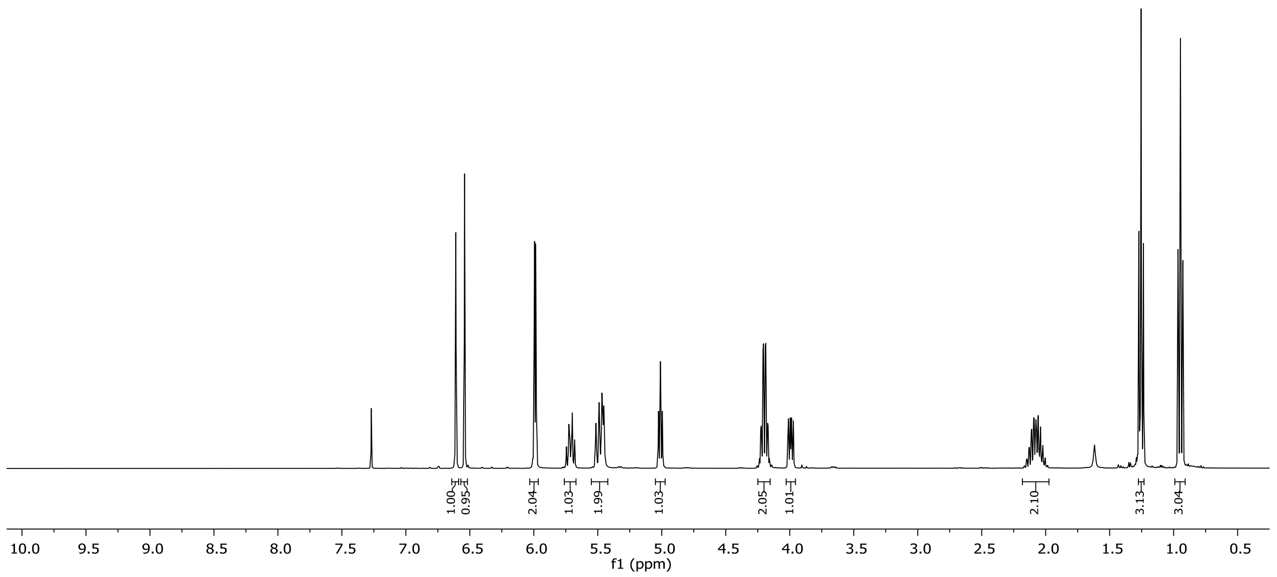
1.00
0.95
2.04
1.03
1.99
1.03
2.05
1.01
2.10
3.13
3.04
10.0 9.5 9.0 8.5 8.0 7.5 7.0 6.5 6.0 5.5 5.0 4.5 4.0 3.5 3.0 2.5 2.0 1.5 1.0 0.5
f1 (ppm)

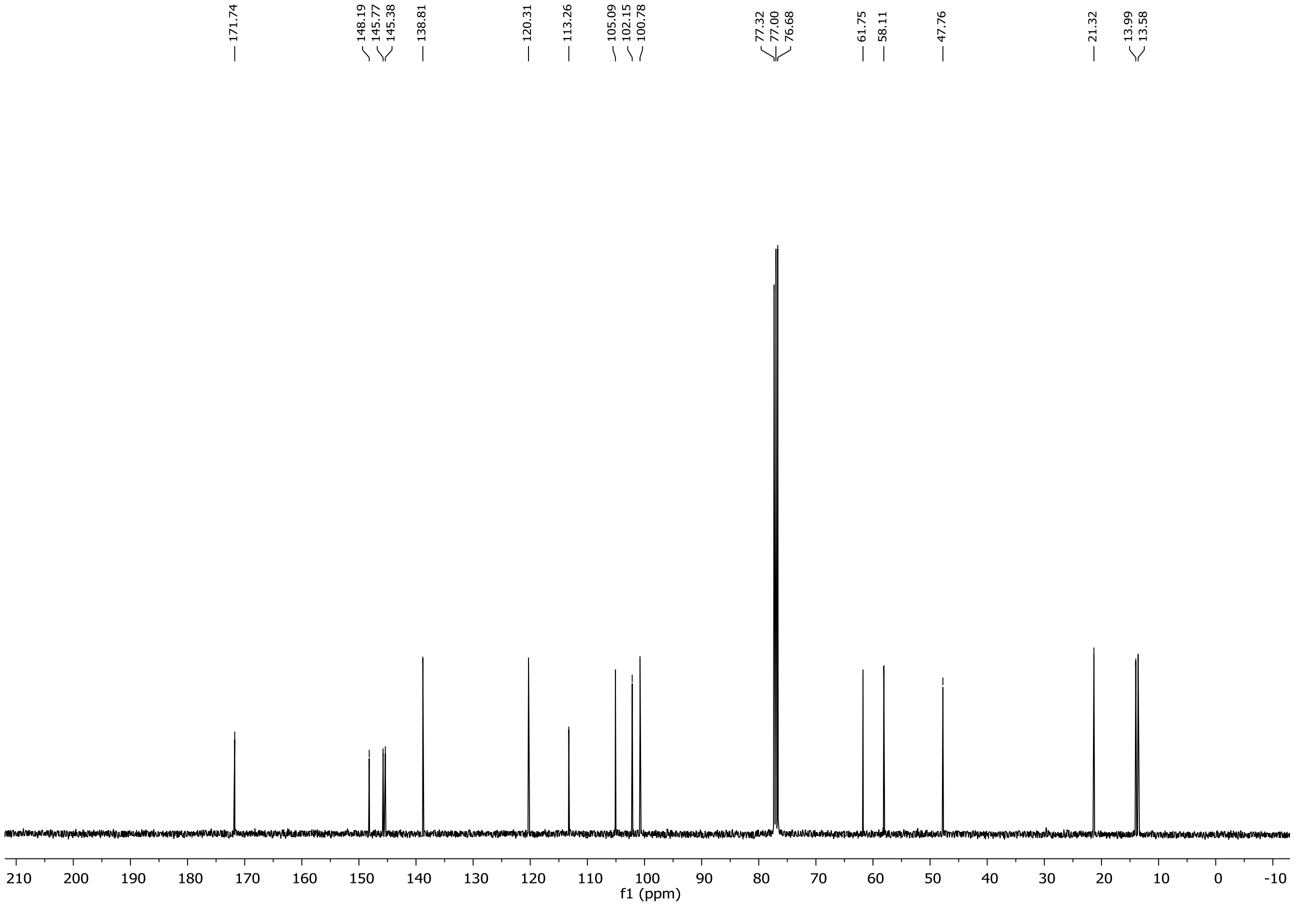
171.74
148.19
145.77
145.38
138.81
120.31
113.26
105.09
102.15
100.78
77.32
77.00
76.68
61.75
58.11
47.76
21.32
13.99
13.58
210 200 190 180 170 160 150 140 130 120 110 100 90 80 70 60 50 40 30 20 10 0 -10
f1 (ppm)

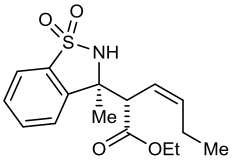
O
O
S
NH
Me
O
OEt
Me

3f

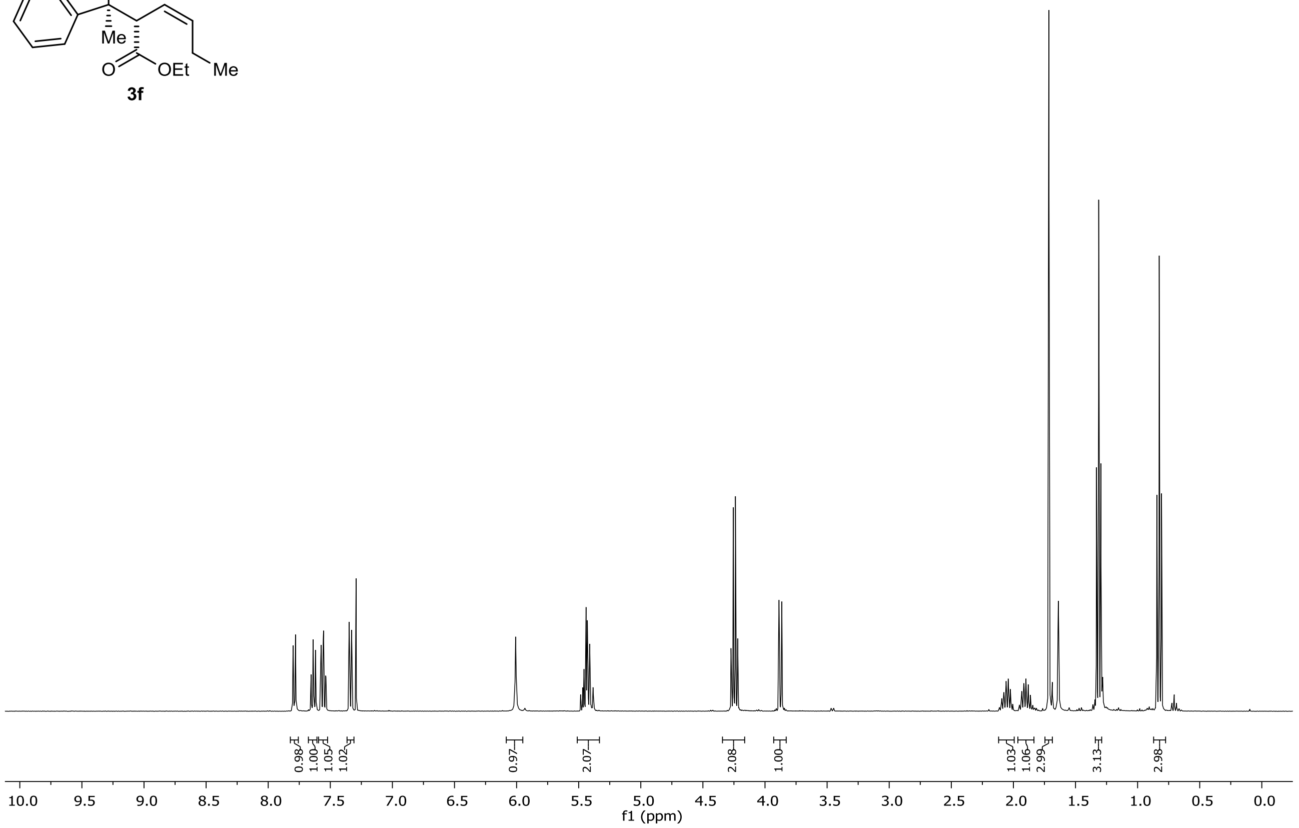
0.98
1.00
1.05
1.02
0.97
2.07
2.08
1.00
1.03
1.06
2.99
3.13
2.98
10.0 9.5 9.0 8.5 8.0 7.5 7.0 6.5 6.0 5.5 5.0 4.5 4.0 3.5 3.0 2.5 2.0 1.5 1.0 0.5 0.0
f1 (ppm)

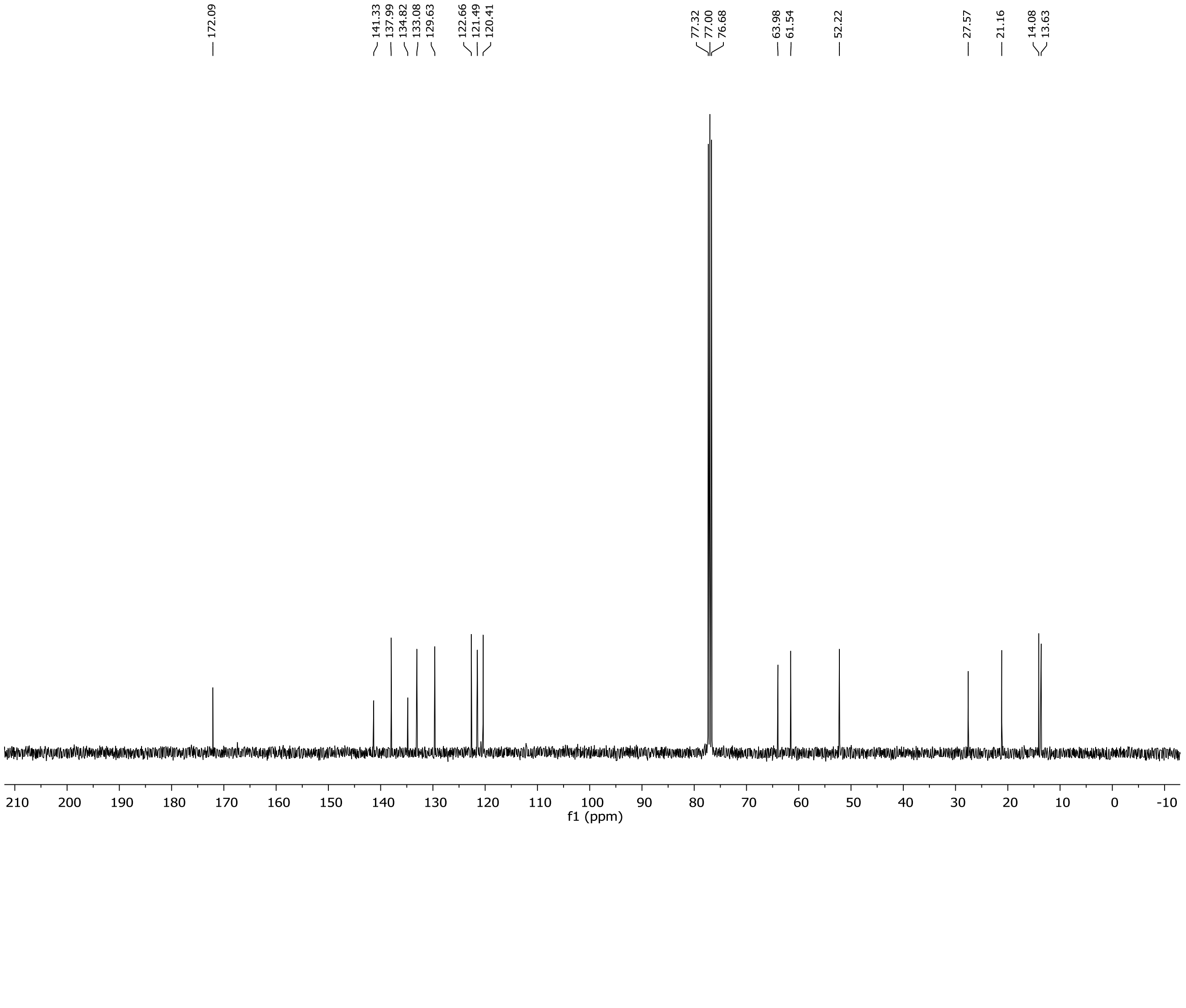
172.09
141.33
137.99
134.82
133.08
129.63
122.66
121.49
120.41
77.32
77.00
76.68
63.98
61.54
52.22
27.57
21.16
14.08
13.63
210 200 190 180 170 160 150 140 130 120 110 100 90 80 70 60 50 40 30 20 10 0 -10
f1 (ppm)

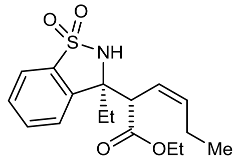
O
O
S
NH
Et
O
OEt
Me

3g

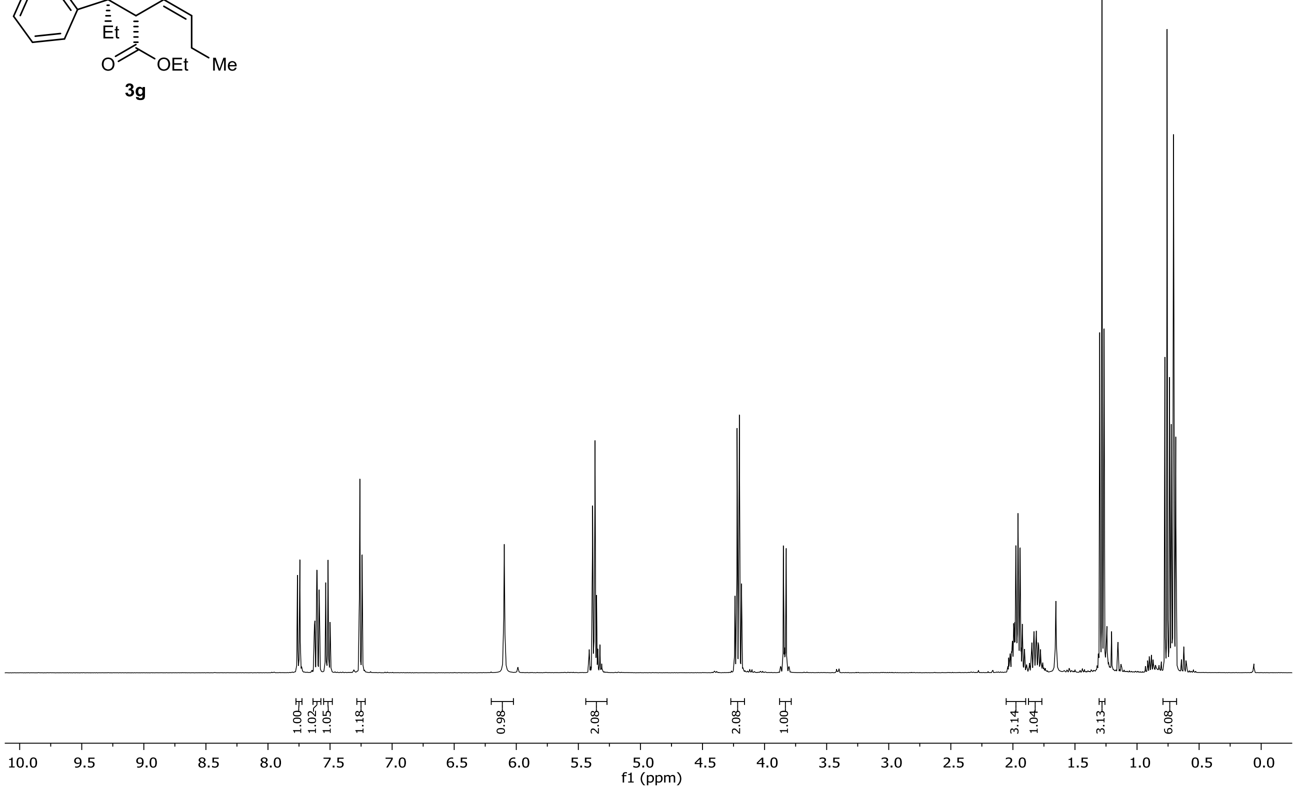
1.00
1.02
1.05
1.18
0.98
2.08
2.08
1.00
3.14
1.04
3.13
6.08
10.0 9.5 9.0 8.5 8.0 7.5 7.0 6.5 6.0 5.5 5.0 4.5 4.0 3.5 3.0 2.5 2.0 1.5 1.0 0.5 0.0
f1 (ppm)

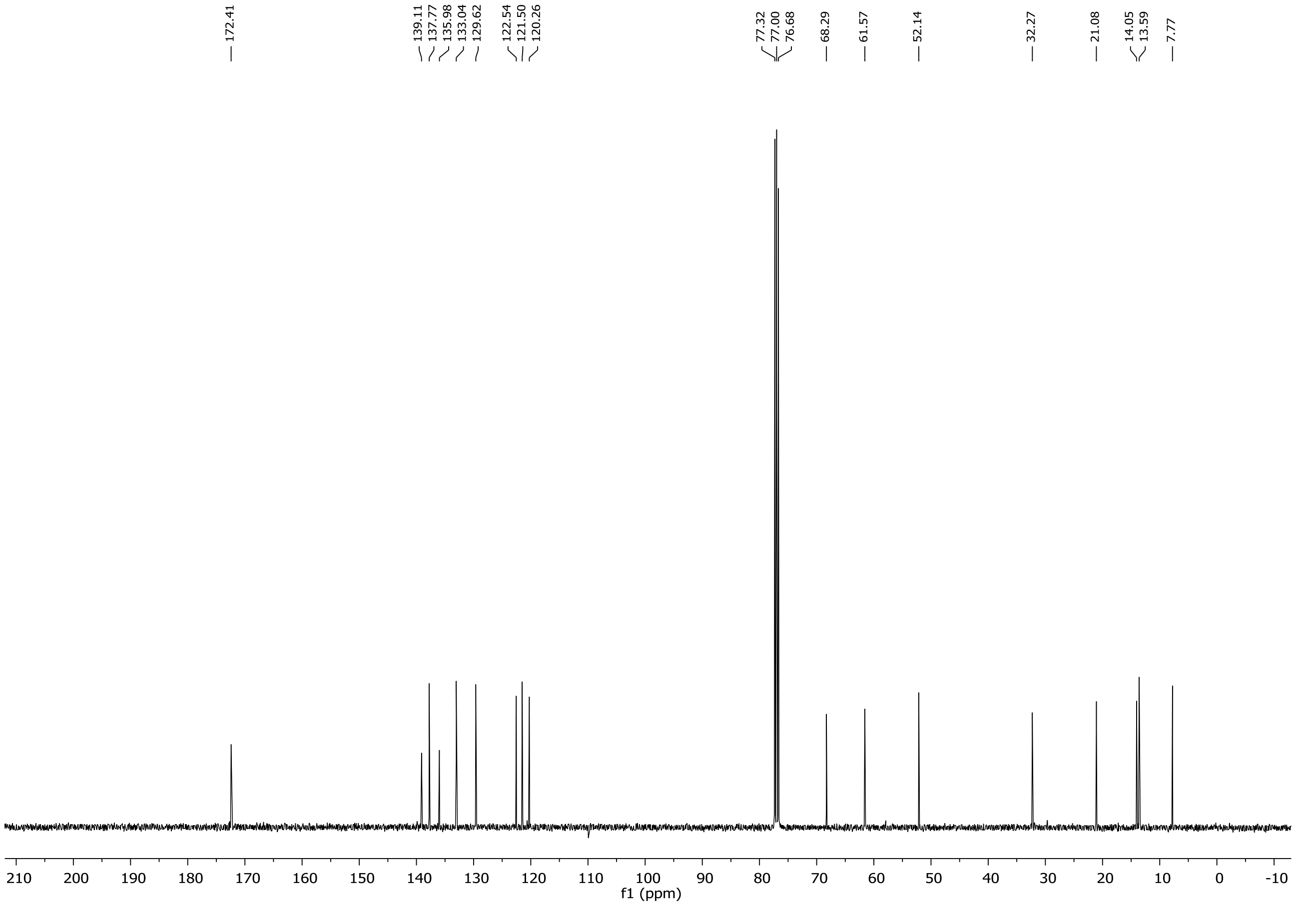
172.41
139.11
137.77
135.98
133.04
129.62
122.54
121.50
120.26
77.32
77.00
76.68
68.29
61.57
52.14
32.27
21.08
14.05
13.59
7.77
210 200 190 180 170 160 150 140 130 120 110 100 90 80 70 60 50 40 30 20 10 0 -10
f1 (ppm)

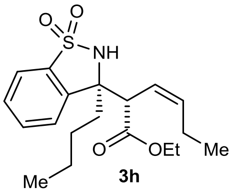
O
O
S
NH
O
OEt
Me
Me

3h

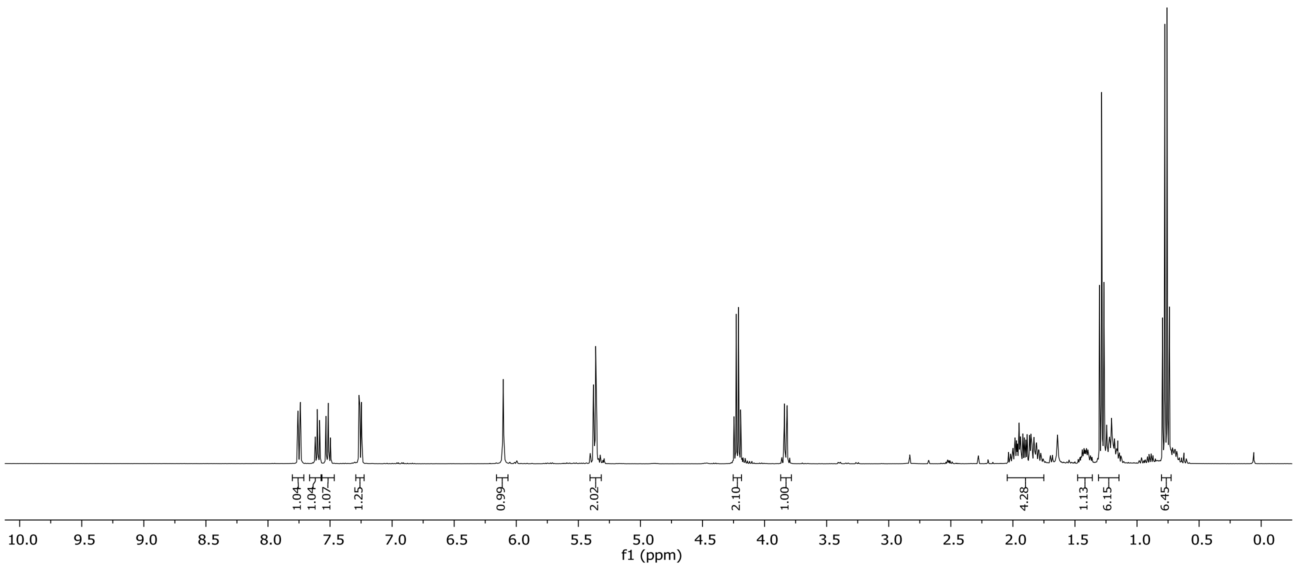
1.04
1.04
1.07
1.25
0.99
2.02
2.10
1.00
4.28
1.13
6.15
6.45
10.0 9.5 9.0 8.5 8.0 7.5 7.0 6.5 6.0 5.5 5.0 4.5 4.0 3.5 3.0 2.5 2.0 1.5 1.0 0.5 0.0
f1 (ppm)

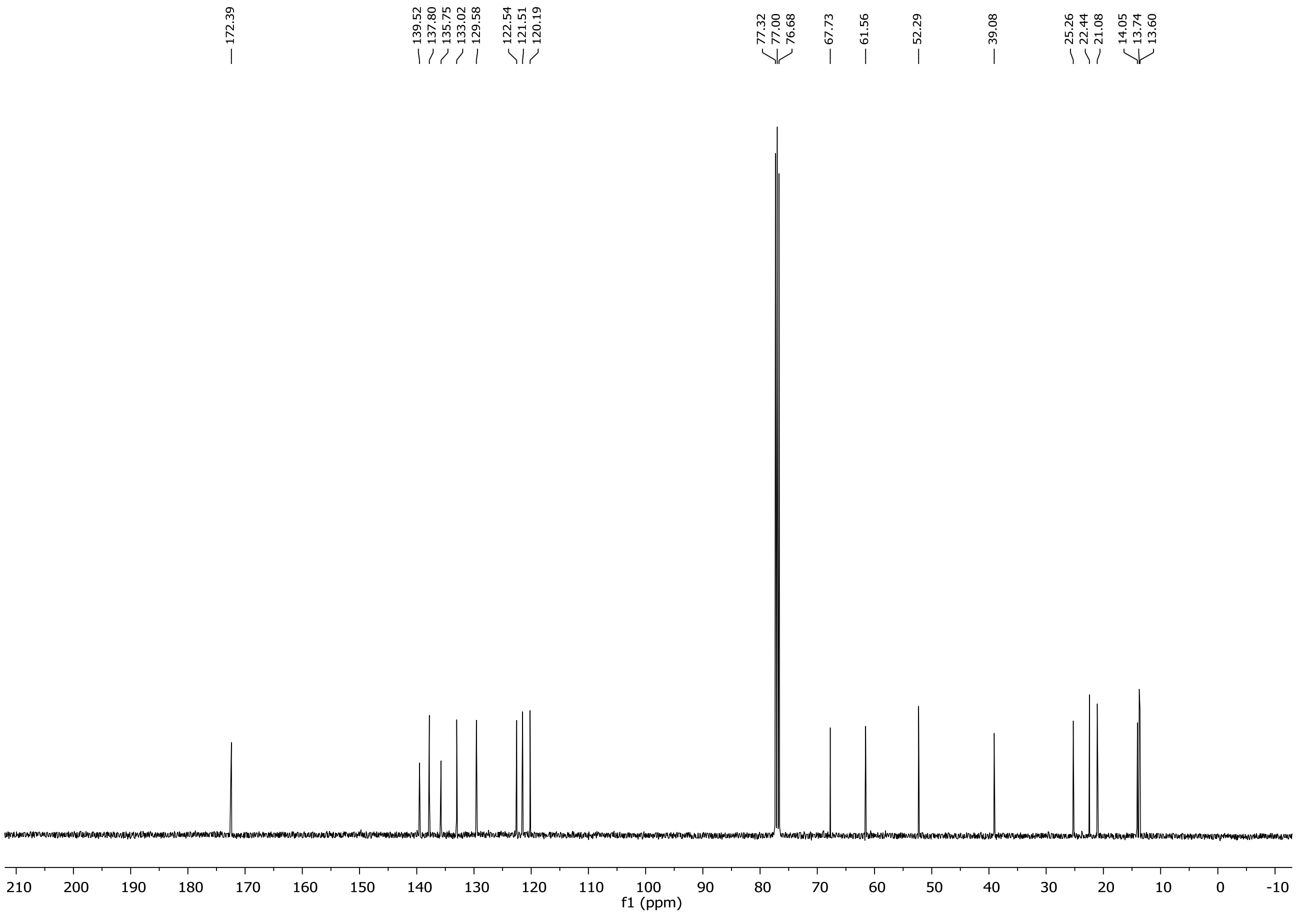
172.39
139.52
137.80
135.75
133.02
129.58
122.54
121.51
120.19
77.32
77.00
76.68
67.73
61.56
52.29
39.08
25.26
22.44
21.08
14.05
13.74
13.60
210 200 190 180 170 160 150 140 130 120 110 100 90 80 70 60 50 40 30 20 10 0 -10
f1 (ppm)

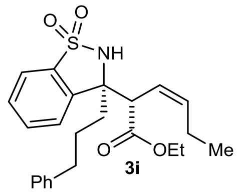
O
O
S
NH
O
OEt
Me
Ph

3i

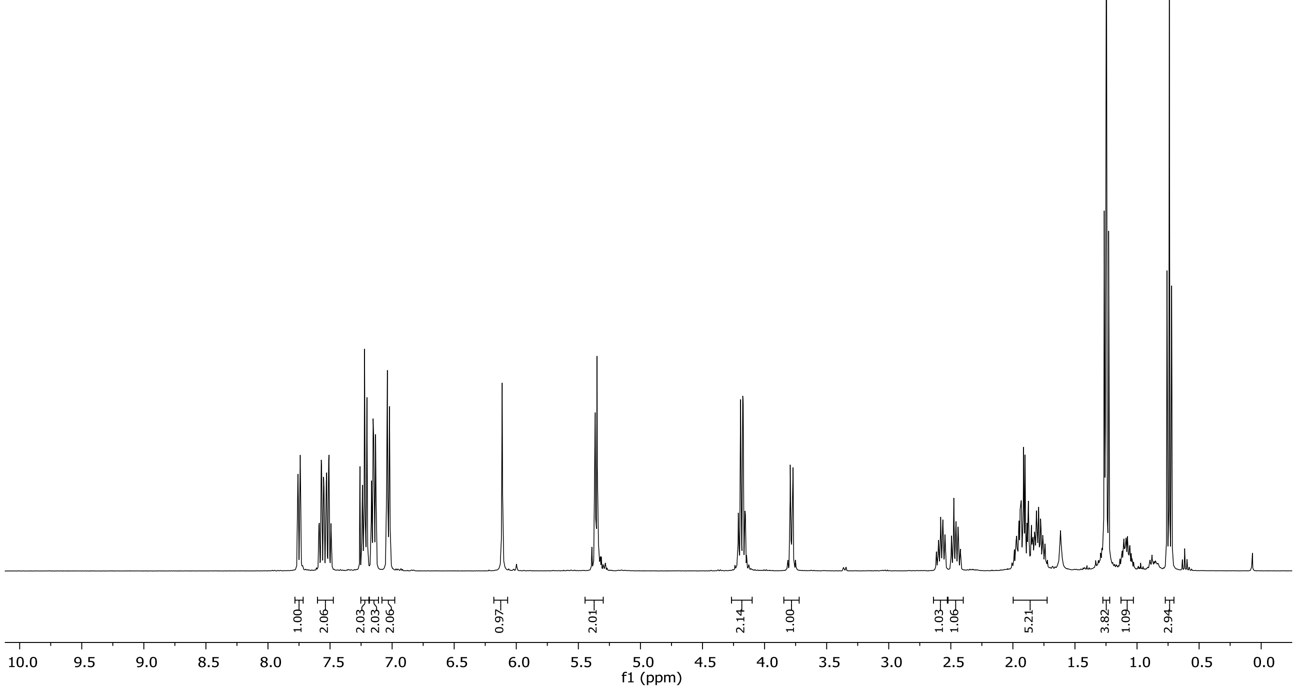
1.00
2.06
2.03
2.03
2.06
0.97
2.01
2.14
1.00
1.03
1.06
5.21
3.82
1.09
2.94
10.0 9.5 9.0 8.5 8.0 7.5 7.0 6.5 6.0 5.5 5.0 4.5 4.0 3.5 3.0 2.5 2.0 1.5 1.0 0.5 0.0
f1 (ppm)

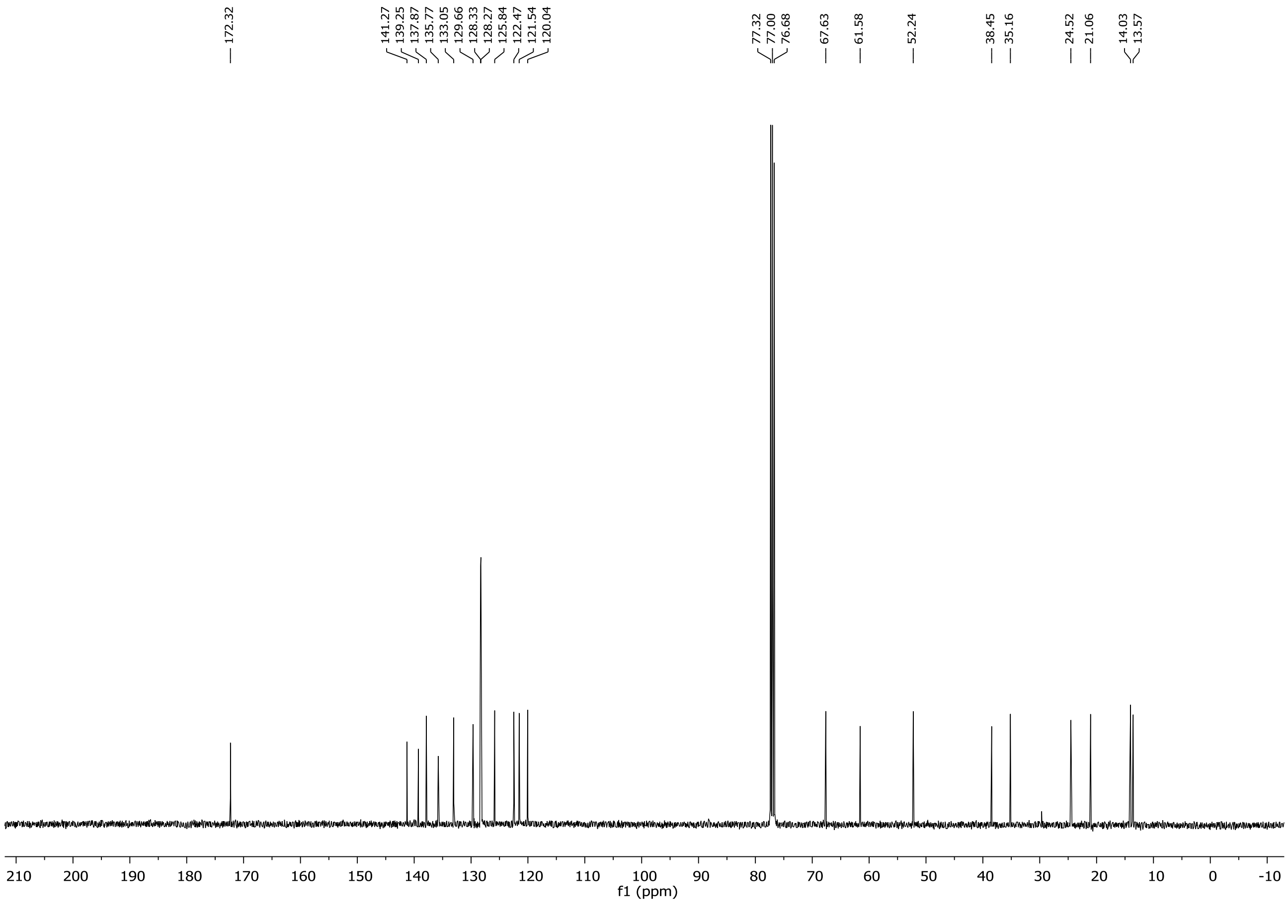
172.32
141.27
139.25
137.87
135.77
133.05
129.66
128.33
128.27
125.84
122.47
121.54
120.04
77.32
77.00
76.68
67.63
61.58
52.24
38.45
35.16
24.52
21.06
14.03
13.57
210 200 190 180 170 160 150 140 130 120 110 100 90 80 70 60 50 40 30 20 10 0 -10
f1 (ppm)

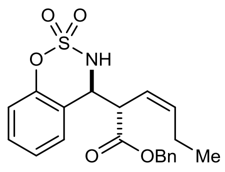
O
O
S
O
NH
O
OBn
Me

3k

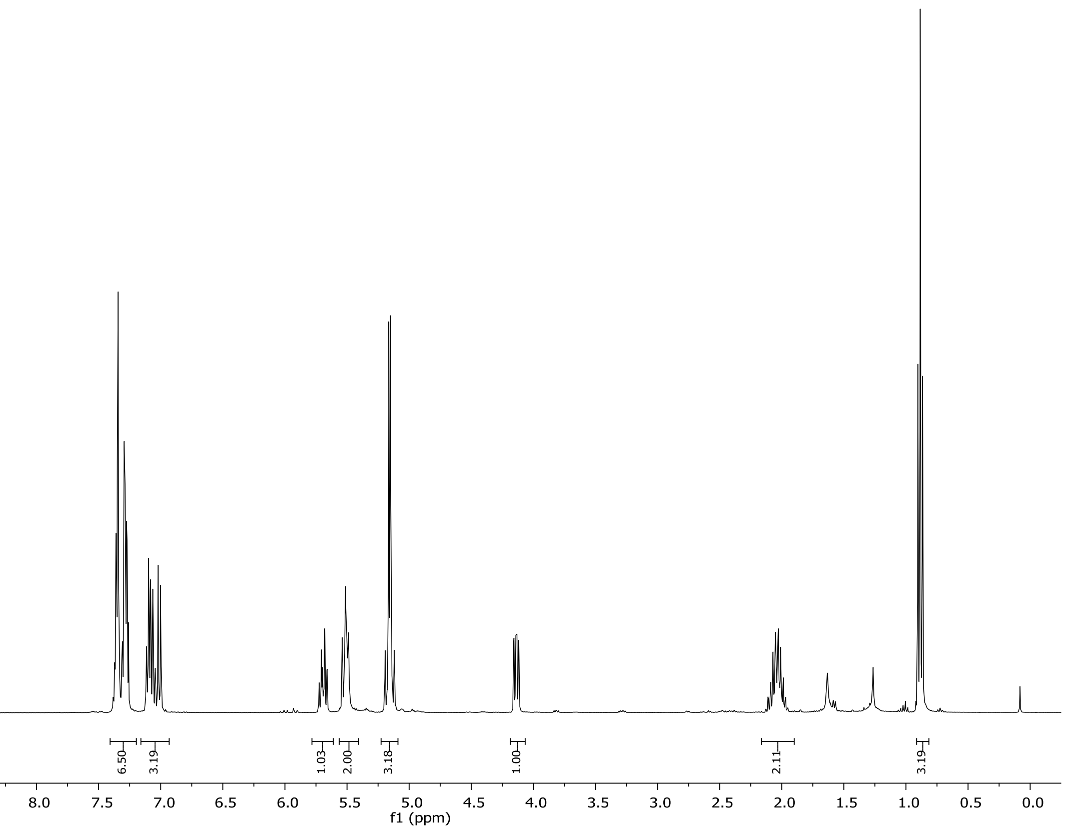
6.50
3.19
1.03
2.00
3.18
1.00
2.11
3.19
10.0 9.5 9.0 8.5 8.0 7.5 7.0 6.5 6.0 5.5 5.0 4.5 4.0 3.5 3.0 2.5 2.0 1.5 1.0 0.5 0.0
f1 (ppm)

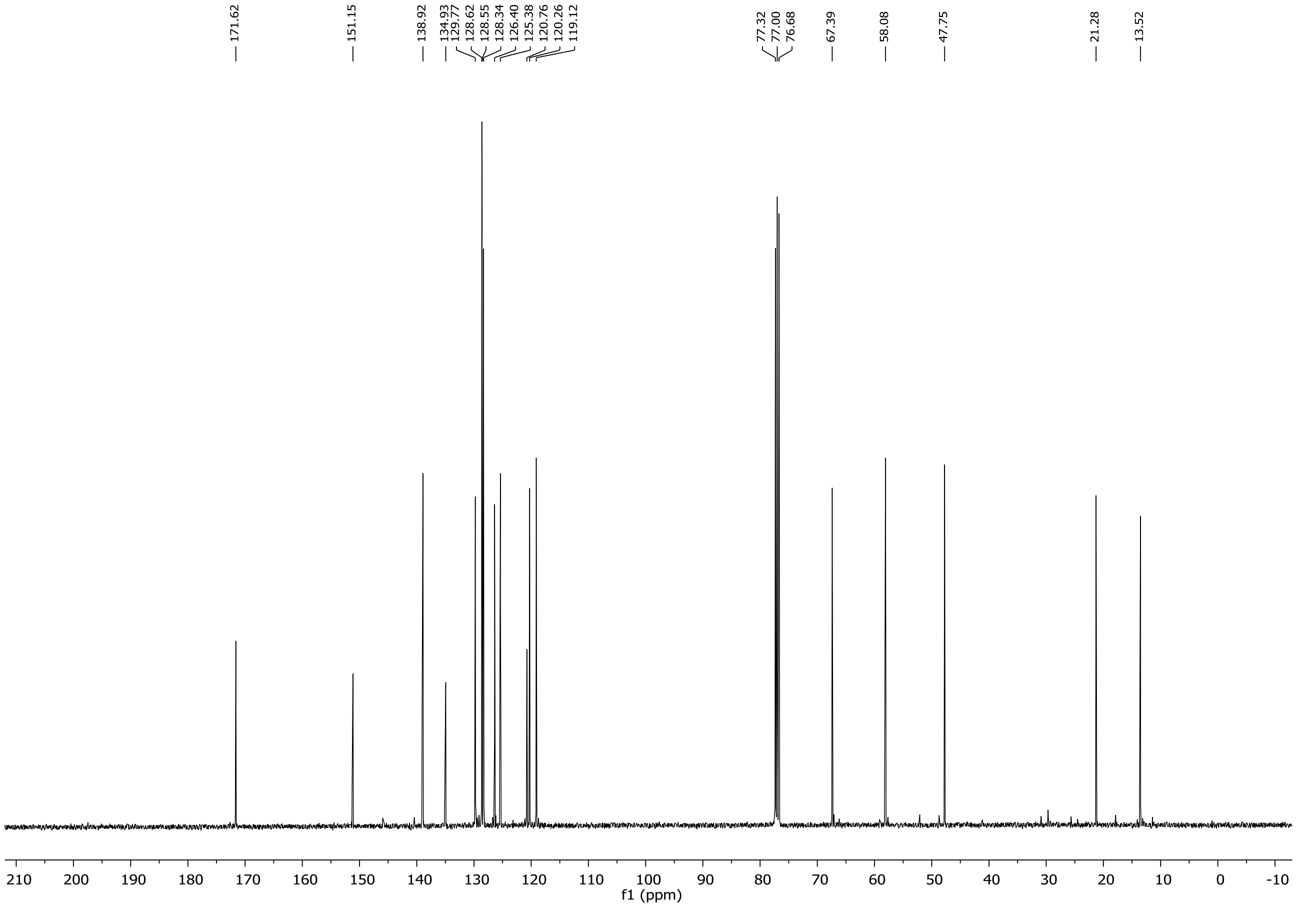
171.62
151.15
138.92
134.93
129.77
128.62
128.55
128.34
126.40
125.38
120.76
120.26
119.12
77.32
77.00
76.68
67.39
58.08
47.75
21.28
13.52
210 200 190 180 170 160 150 140 130 120 110 100 90 80 70 60 50 40 30 20 10 0 -10
f1 (ppm)

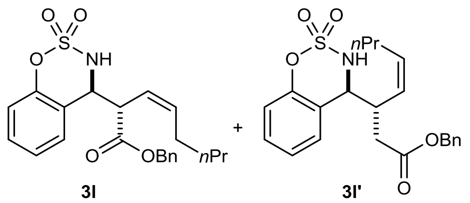

3l **3l'**
(assumed stereochemistry)

95:5 inseparable mixture

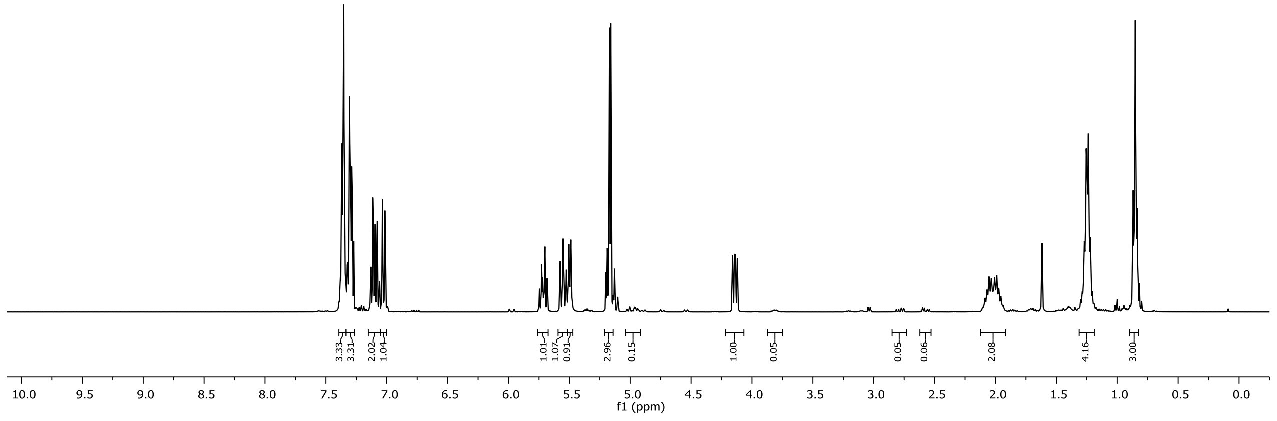

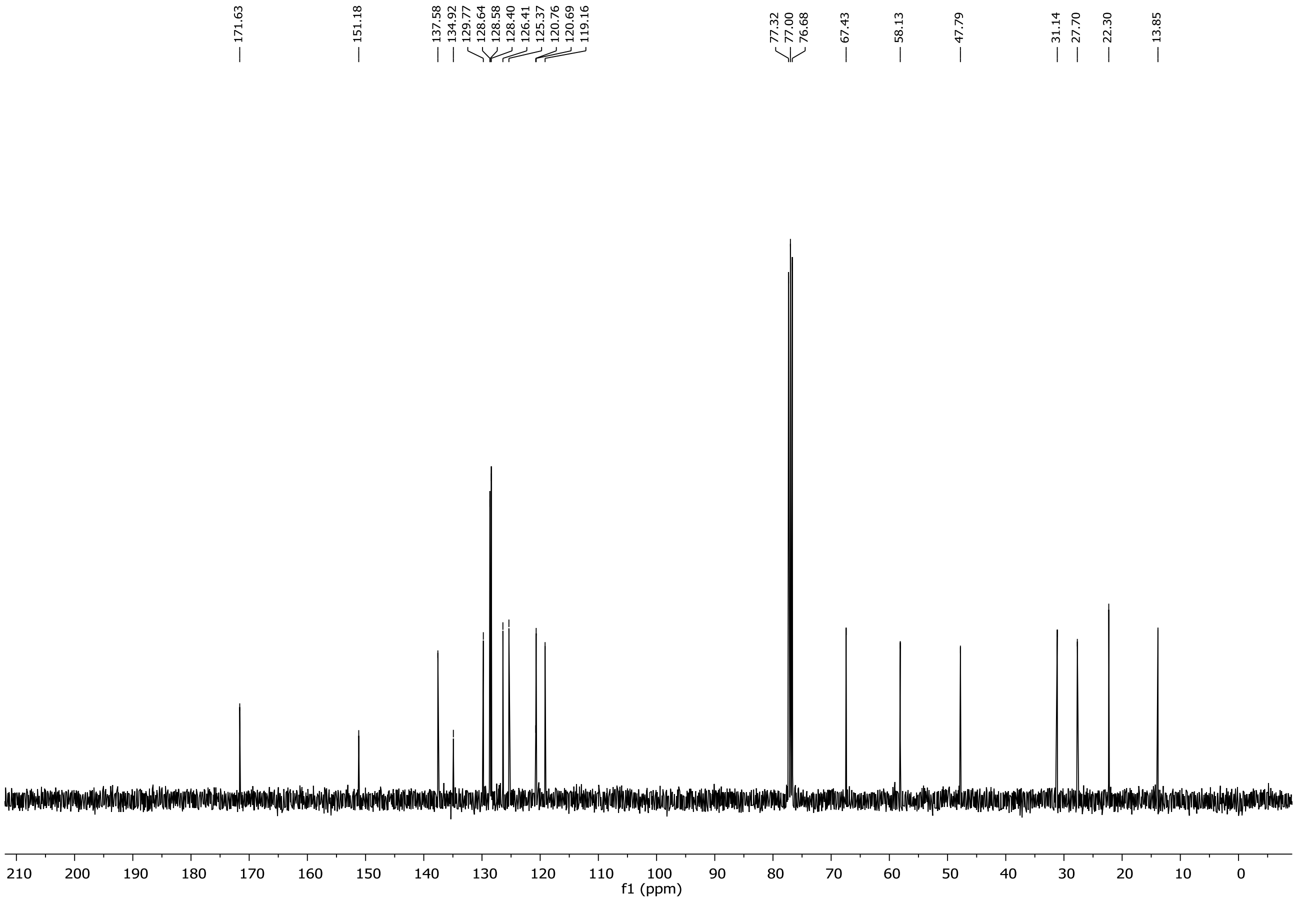

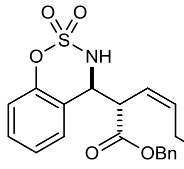
O
O
S
O
NH
Cl
O
OBn

3m

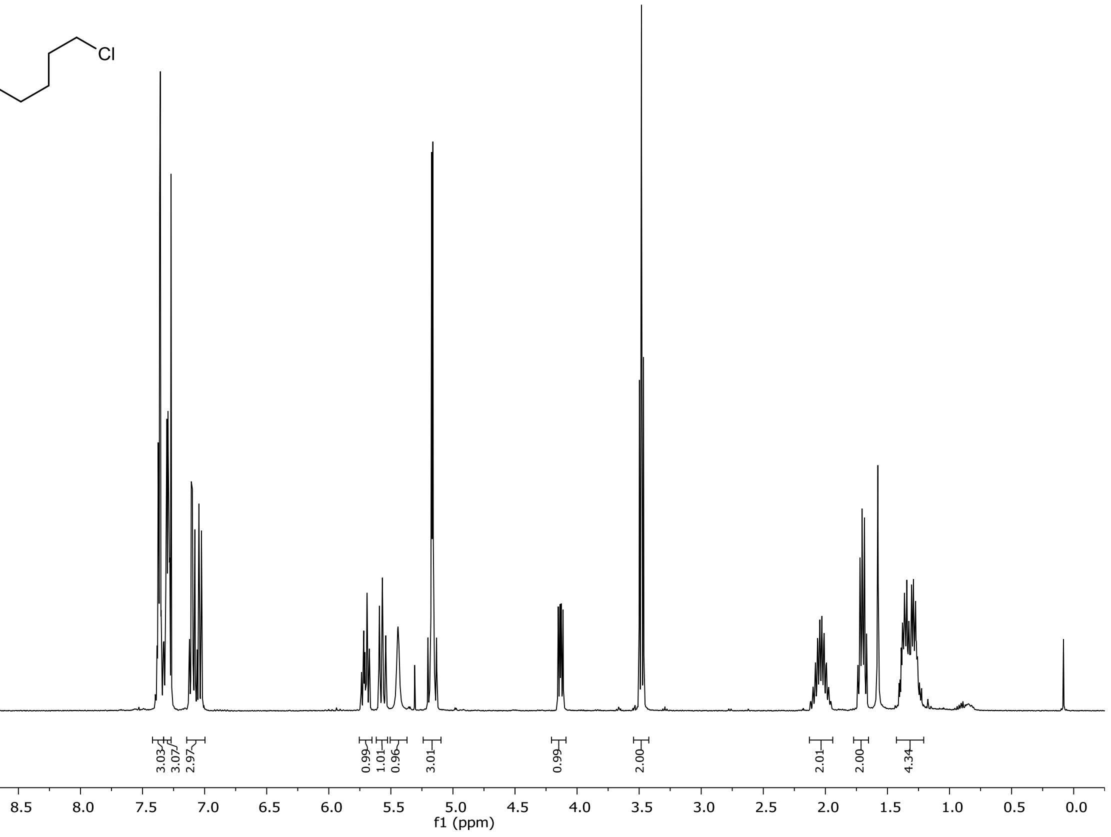
3.03
3.07
2.97
0.99
1.01
0.96
3.01
0.99
2.00
2.01
2.00
4.34
10.0
9.5
9.0
8.5
8.0
7.5
7.0
6.5
6.0
5.5
5.0
4.5
4.0
3.5
3.0
2.5
2.0
1.5
1.0
0.5
0.0
f1 (ppm)

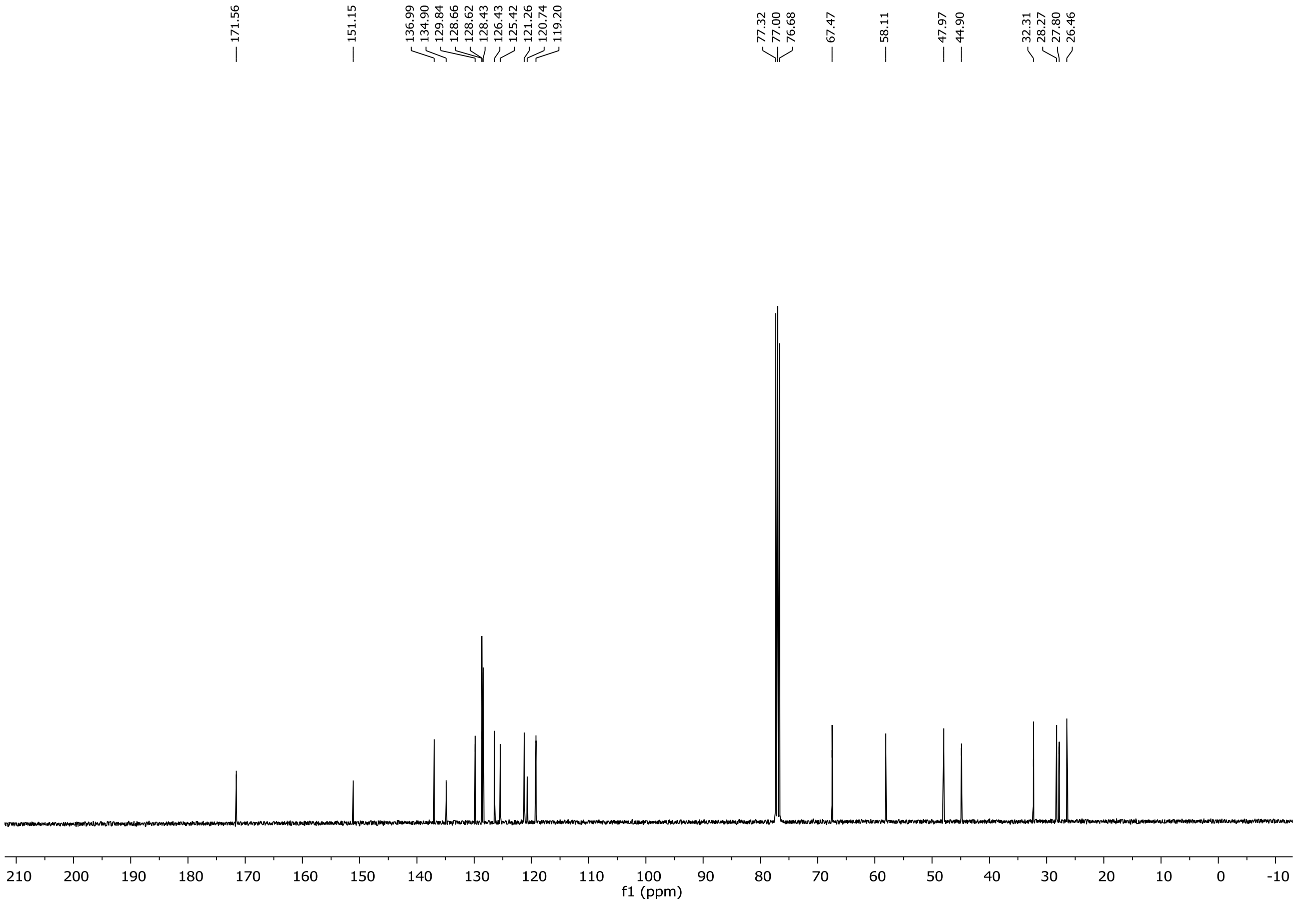
171.56
151.15
136.99
134.90
129.84
128.66
128.62
128.43
126.43
125.42
121.26
120.74
119.20
77.32
77.00
76.68
67.47
58.11
47.97
44.90
32.31
28.27
27.80
26.46
210
200
190
180
170
160
150
140
130
120
110
100
90
80
70
60
50
40
30
20
10
0
-10
f1 (ppm)

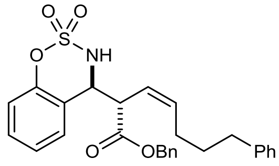
O
O
S
O
NH
O
OBn
Ph

3n

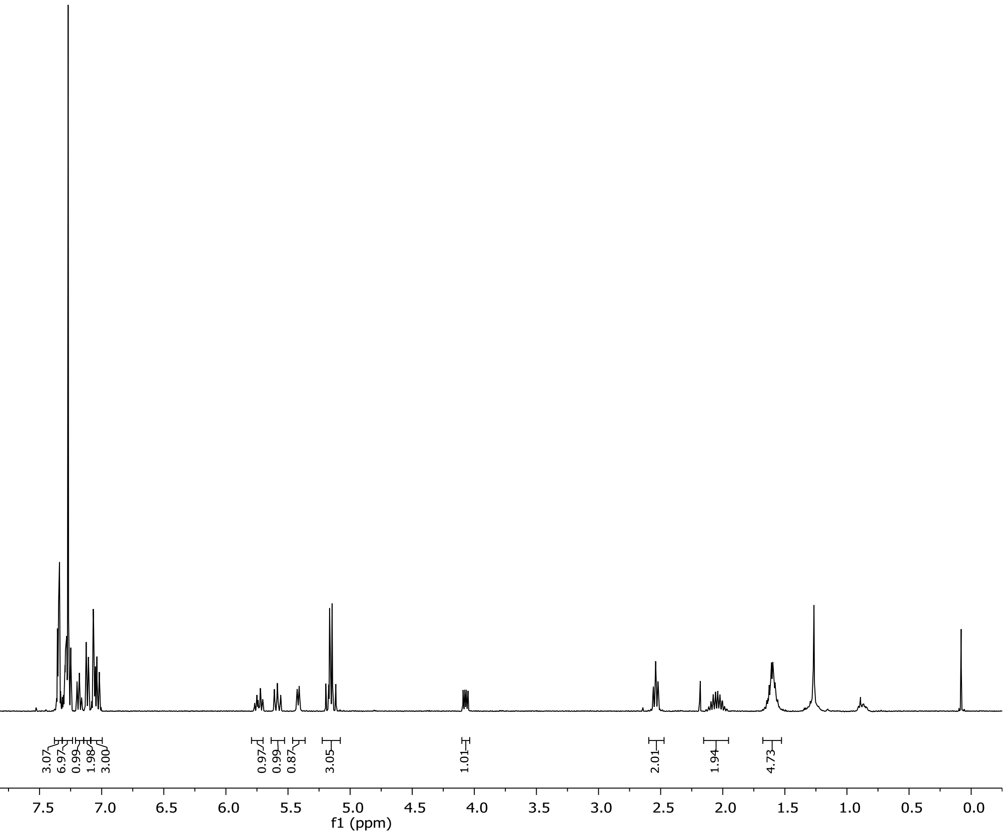
3.07
6.97
0.99
1.98
3.00
0.97
0.99
0.87
3.05
1.01
2.01
1.94
4.73
10.0 9.5 9.0 8.5 8.0 7.5 7.0 6.5 6.0 5.5 5.0 4.5 4.0 3.5 3.0 2.5 2.0 1.5 1.0 0.5 0.0
f1 (ppm)

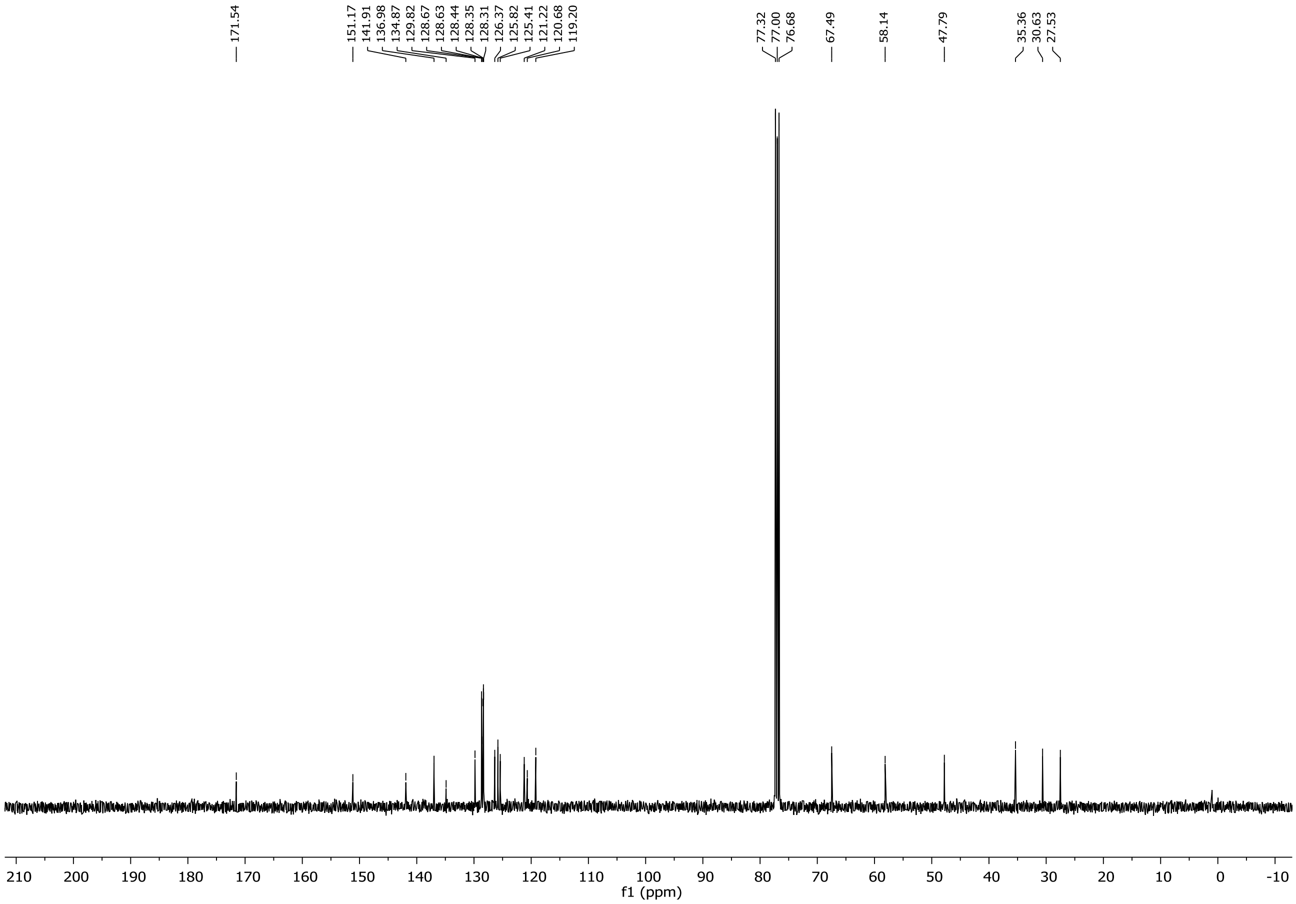
171.54
151.17
141.91
136.98
134.87
129.82
128.67
128.63
128.44
128.35
128.31
126.37
125.82
125.41
121.22
120.68
119.20
77.32
77.00
76.68
67.49
58.14
47.79
35.36
30.63
27.53
210 200 190 180 170 160 150 140 130 120 110 100 90 80 70 60 50 40 30 20 10 0 -10
f1 (ppm)

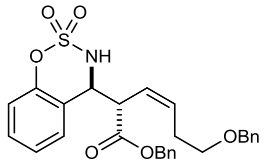
O
O
S
O
NH
O
OBn
OBn

3o

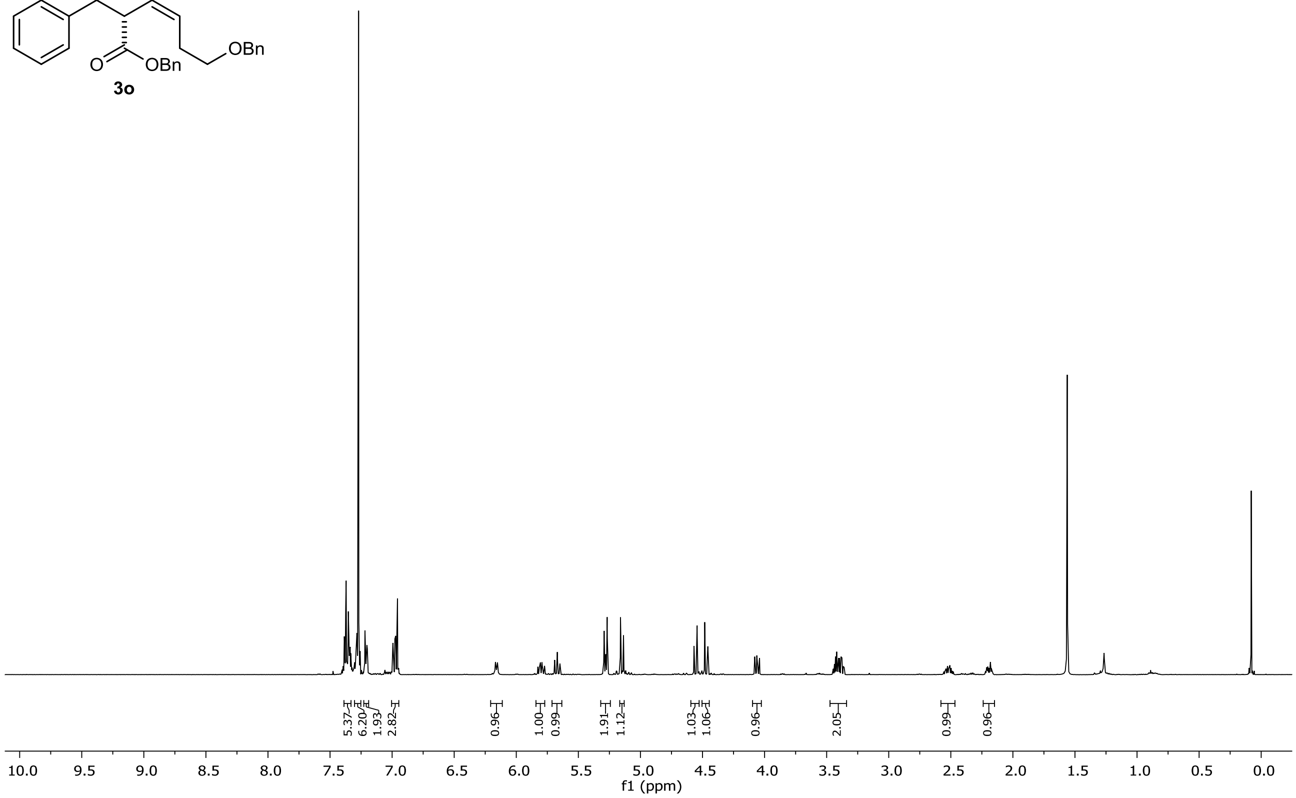
5.37
6.20
1.93
2.82
0.96
1.00
0.99
1.91
1.12
1.03
1.06
0.96
2.05
0.99
0.96
10.0 9.5 9.0 8.5 8.0 7.5 7.0 6.5 6.0 5.5 5.0 4.5 4.0 3.5 3.0 2.5 2.0 1.5 1.0 0.5 0.0
f1 (ppm)

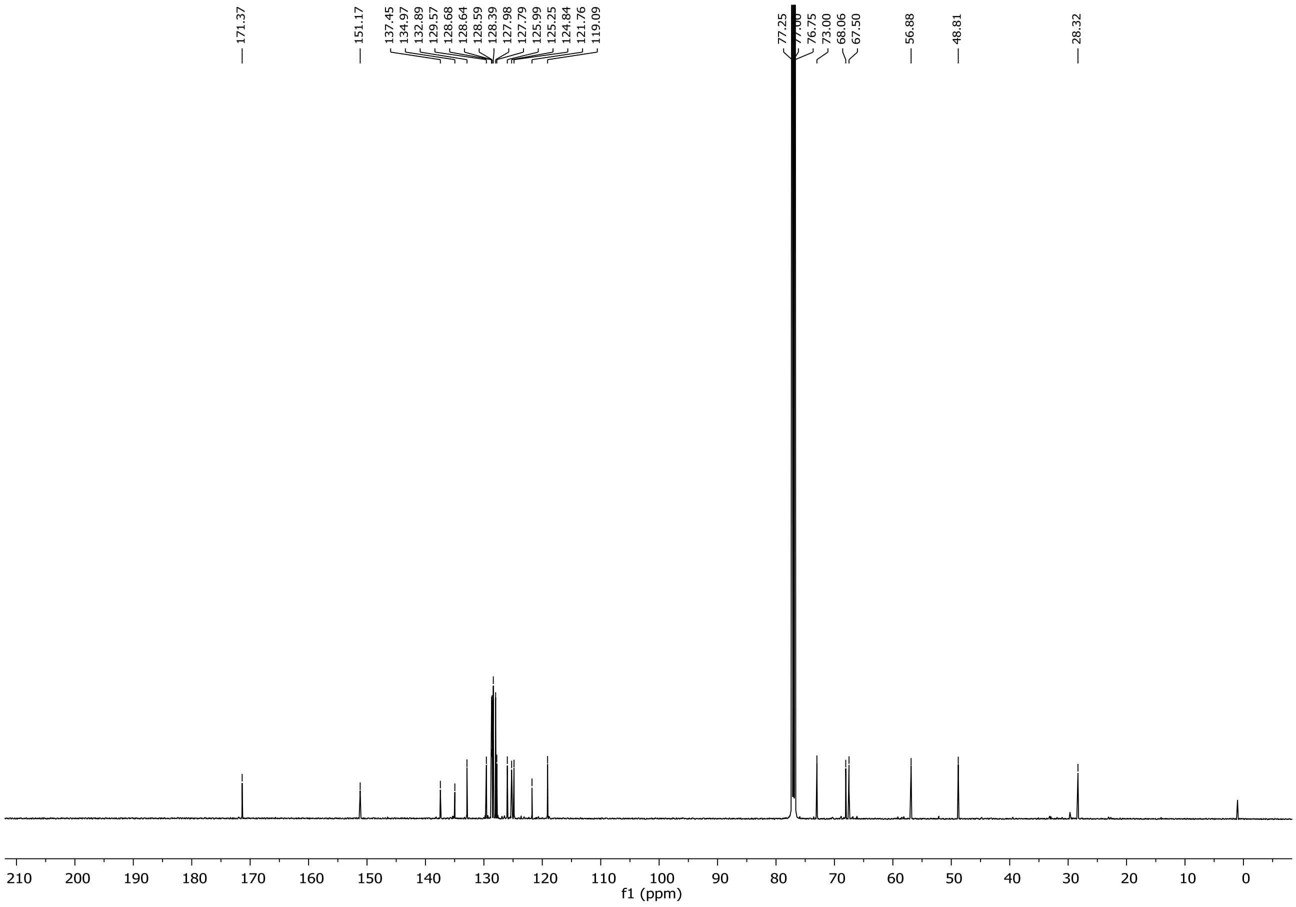
171.37
151.17
137.45
134.97
132.89
129.57
128.68
128.64
128.59
128.39
127.98
127.79
125.99
125.25
124.84
121.76
119.09
77.25
76.75
73.00
68.06
67.50
56.88
48.81
28.32
210 200 190 180 170 160 150 140 130 120 110 100 90 80 70 60 50 40 30 20 10 0
f1 (ppm)

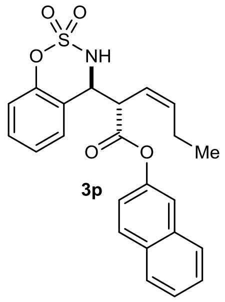
O
O
S
O
NH
O
O
Me

3p

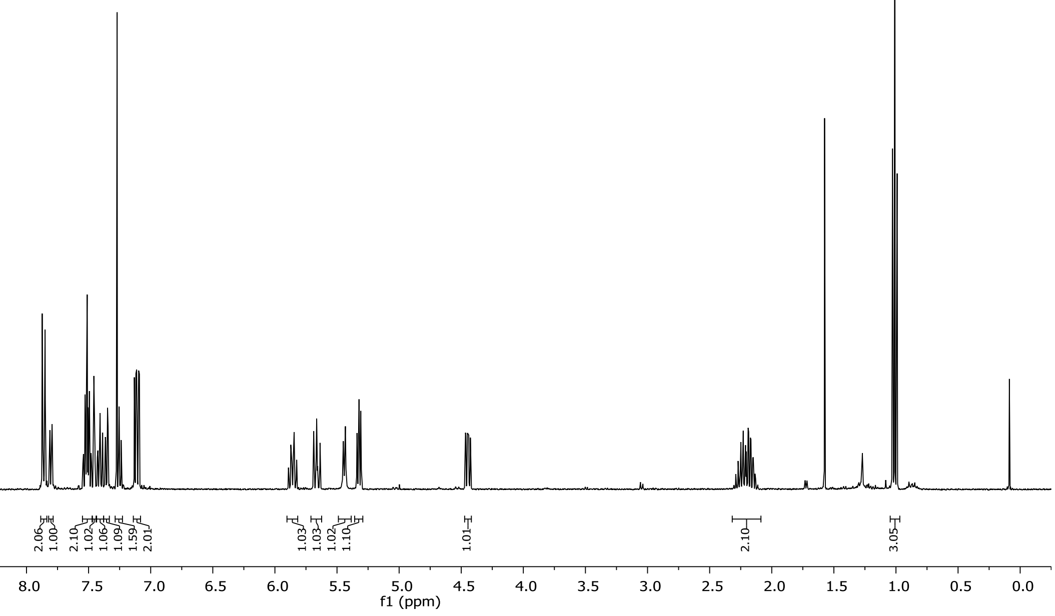
2.06
1.00
2.10
1.02
1.06
1.09
1.59
2.01
1.03
1.03
1.02
1.10
1.01
2.10
3.05
f1 (ppm)

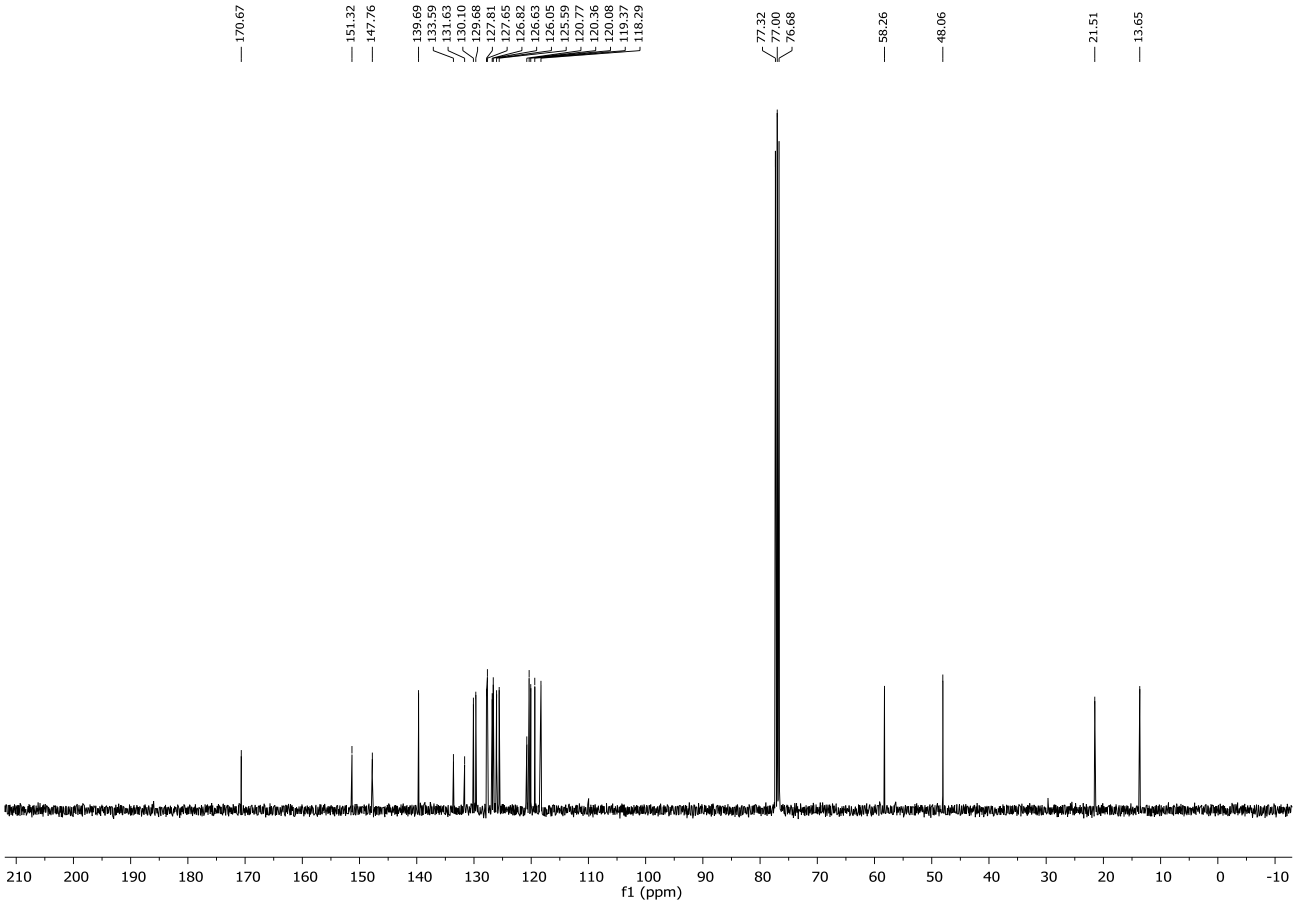
170.67
151.32
147.76
139.69
133.59
131.63
130.10
129.68
127.81
127.65
126.82
126.63
126.05
125.59
120.77
120.36
120.08
119.37
118.29
77.32
77.00
76.68
58.26
48.06
21.51
13.65
f1 (ppm)

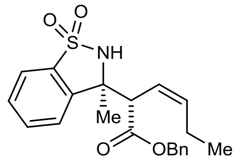
O
O
S
NH
Me
O
OBn
Me

3q

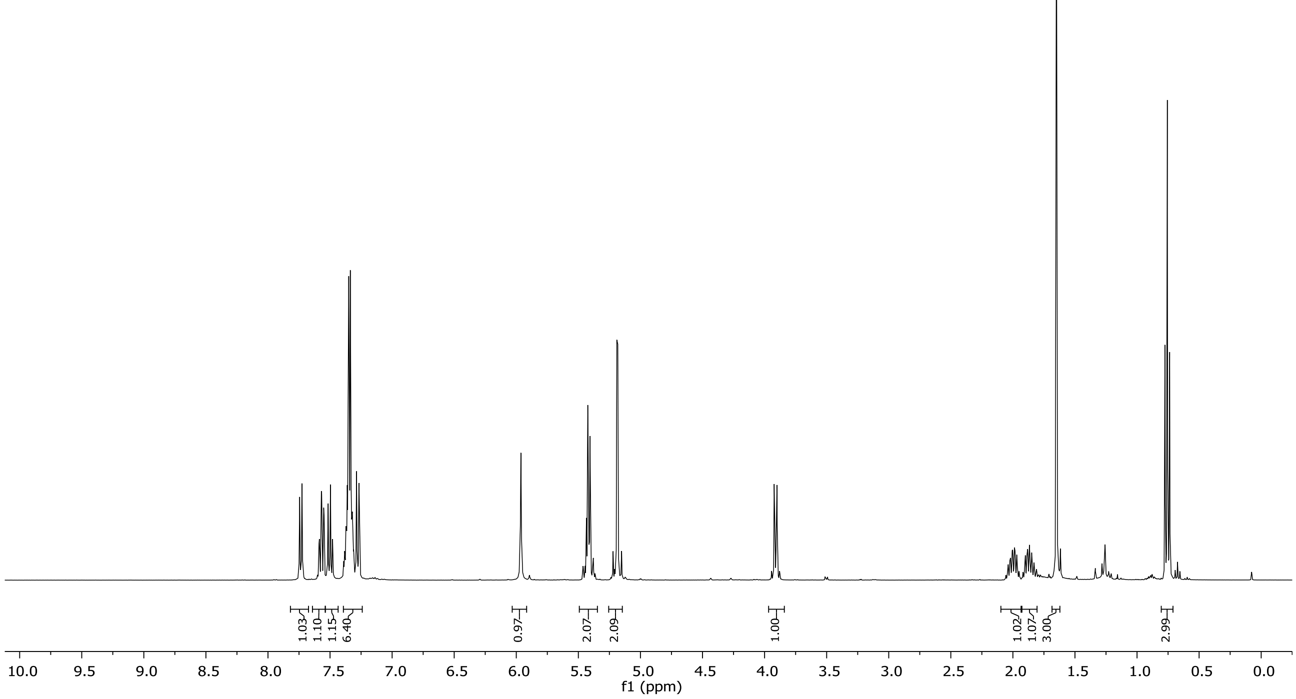
1.03
1.10
1.15
6.40
0.97
2.07
2.09
1.00
1.02
1.07
3.00
2.99
10.0 9.5 9.0 8.5 8.0 7.5 7.0 6.5 6.0 5.5 5.0 4.5 4.0 3.5 3.0 2.5 2.0 1.5 1.0 0.5 0.0
f1 (ppm)

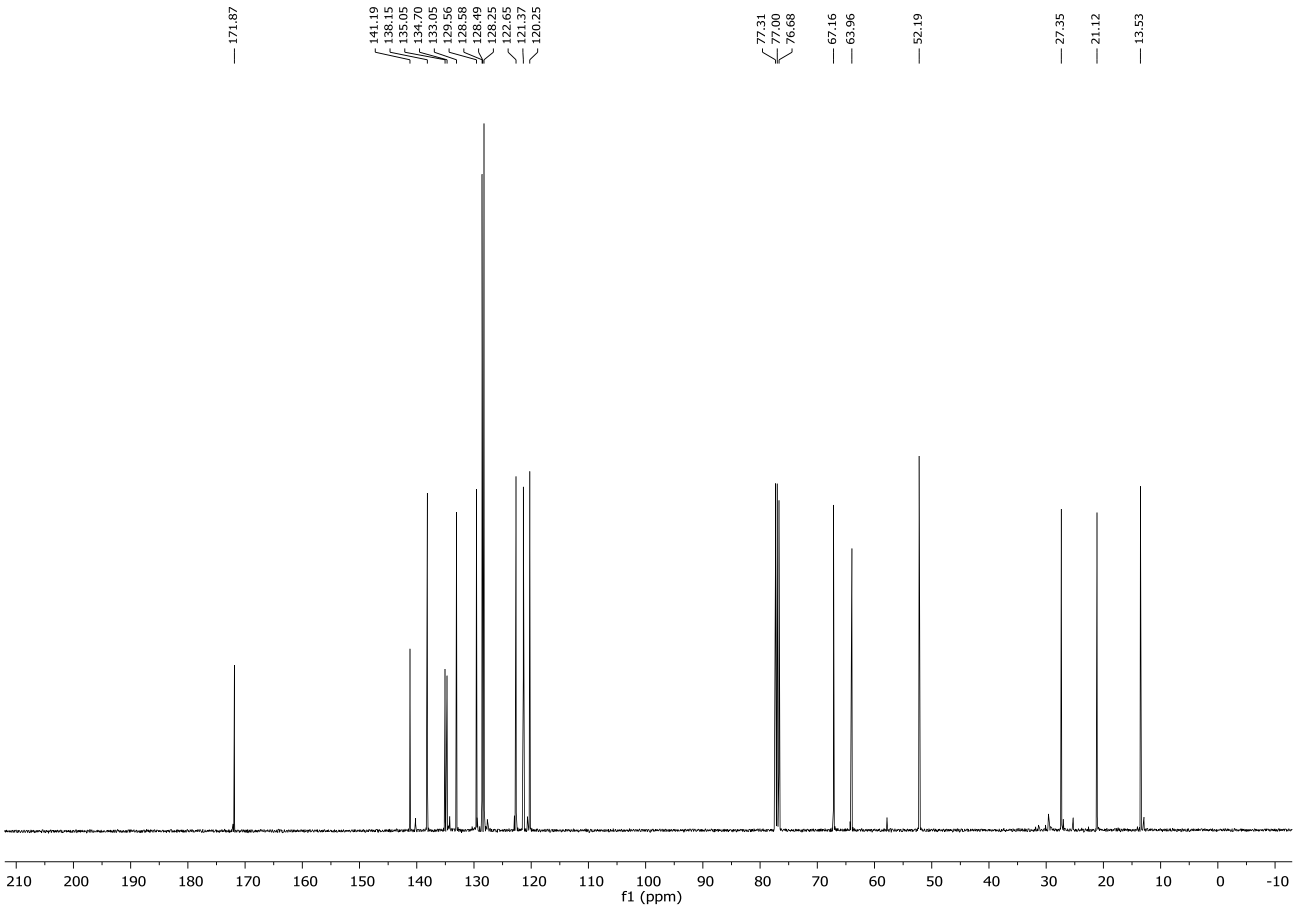
171.87
141.19
138.15
135.05
134.70
133.05
129.56
128.58
128.49
128.25
122.65
121.37
120.25
77.31
77.00
76.68
67.16
63.96
52.19
27.35
21.12
13.53
210 200 190 180 170 160 150 140 130 120 110 100 90 80 70 60 50 40 30 20 10 0 -10
f1 (ppm)

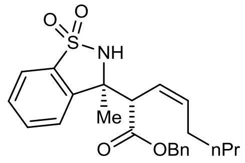
O
O
S
NH
Me
O
OBn
nPr

3r

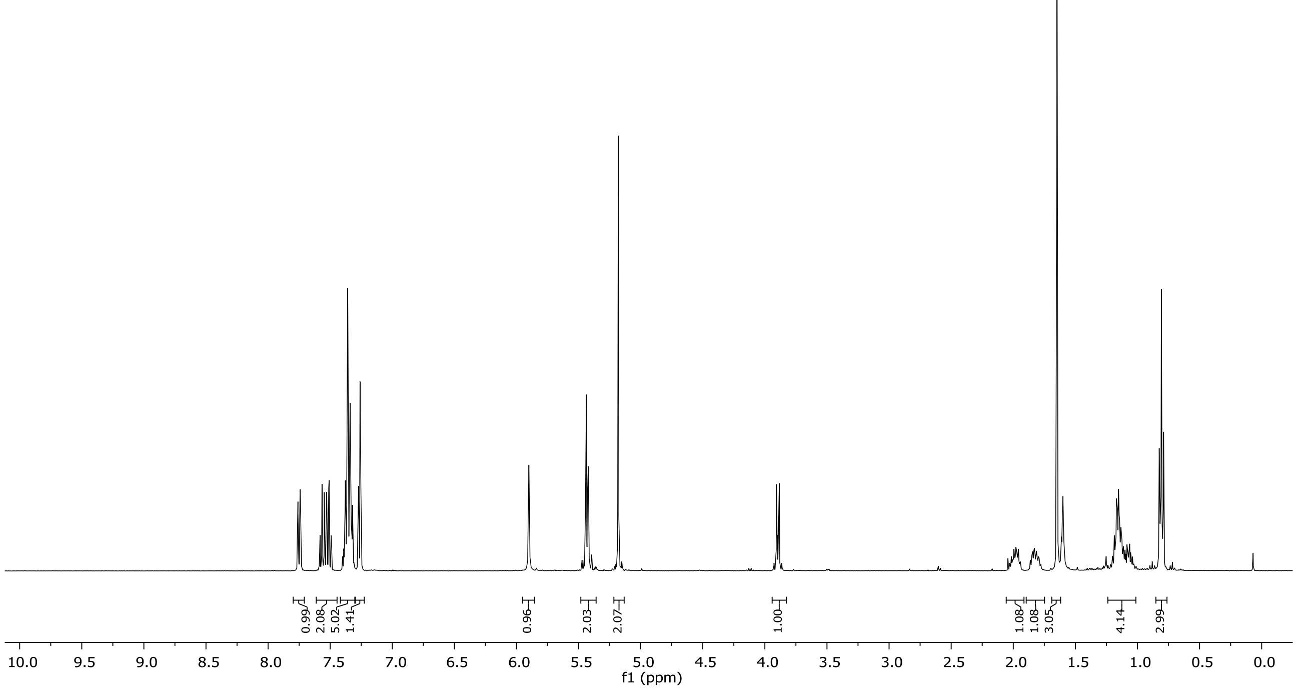
0.99
2.08
5.02
1.41
0.96
2.03
2.07
1.00
1.08
1.08
3.05
4.14
2.99
10.0 9.5 9.0 8.5 8.0 7.5 7.0 6.5 6.0 5.5 5.0 4.5 4.0 3.5 3.0 2.5 2.0 1.5 1.0 0.5 0.0
f1 (ppm)

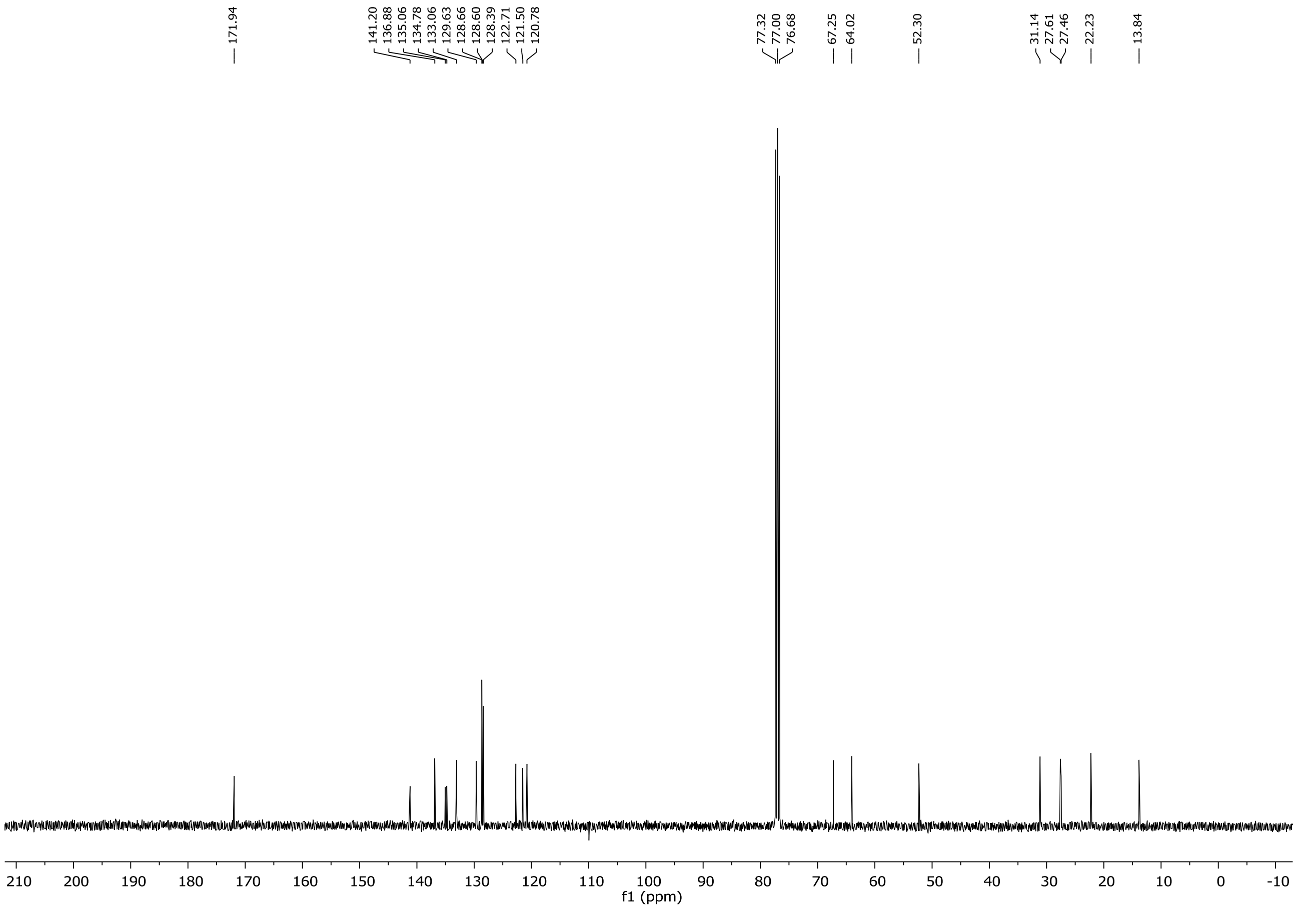
171.94
141.20
136.88
135.06
134.78
133.06
129.63
128.66
128.60
128.39
122.71
121.50
120.78
77.32
77.00
76.68
67.25
64.02
52.30
31.14
27.61
27.46
22.23
13.84
210 200 190 180 170 160 150 140 130 120 110 100 90 80 70 60 50 40 30 20 10 0 -10
f1 (ppm)

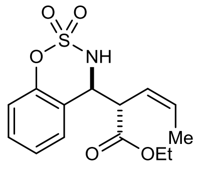

3s

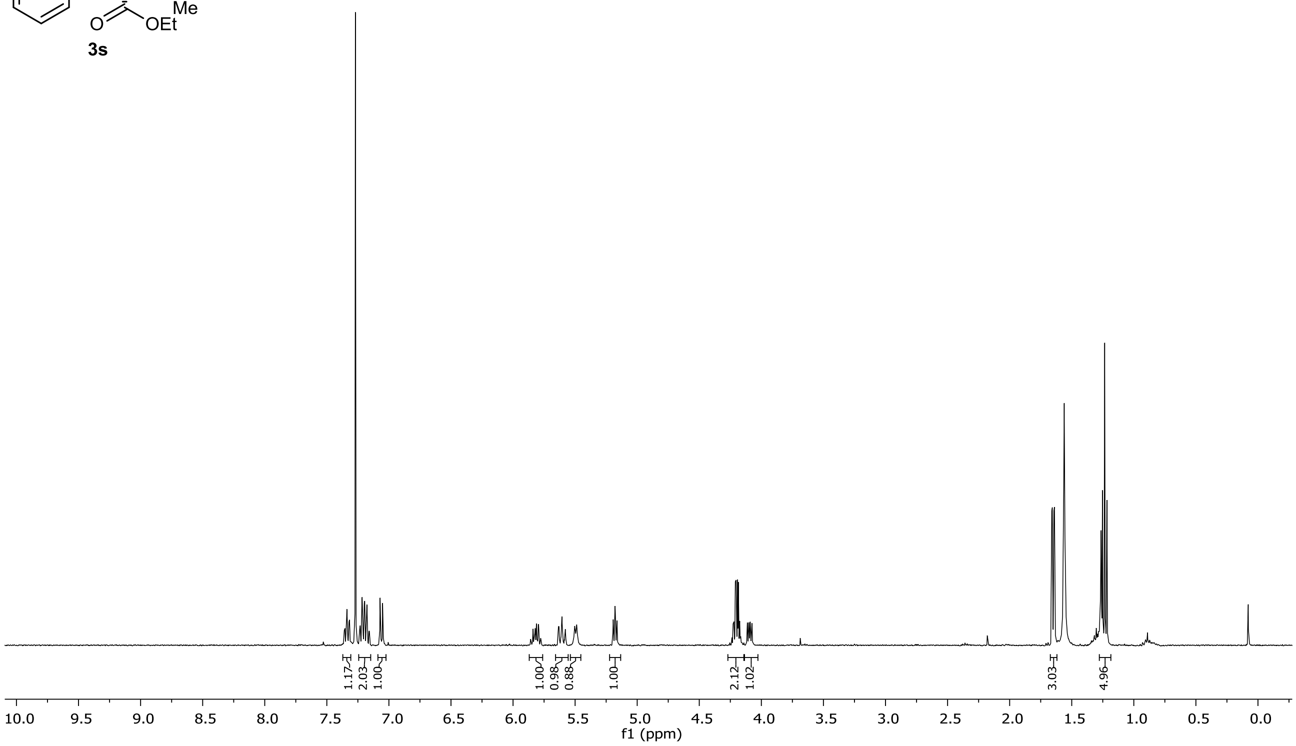
1.17
2.03
1.00
1.00
0.98
0.88
1.00
2.12
1.02
3.03
4.96
10.0
9.5
9.0
8.5
8.0
7.5
7.0
6.5
6.0
5.5
5.0
4.5
4.0
3.5
3.0
2.5
2.0
1.5
1.0
0.5
0.0
f1 (ppm)

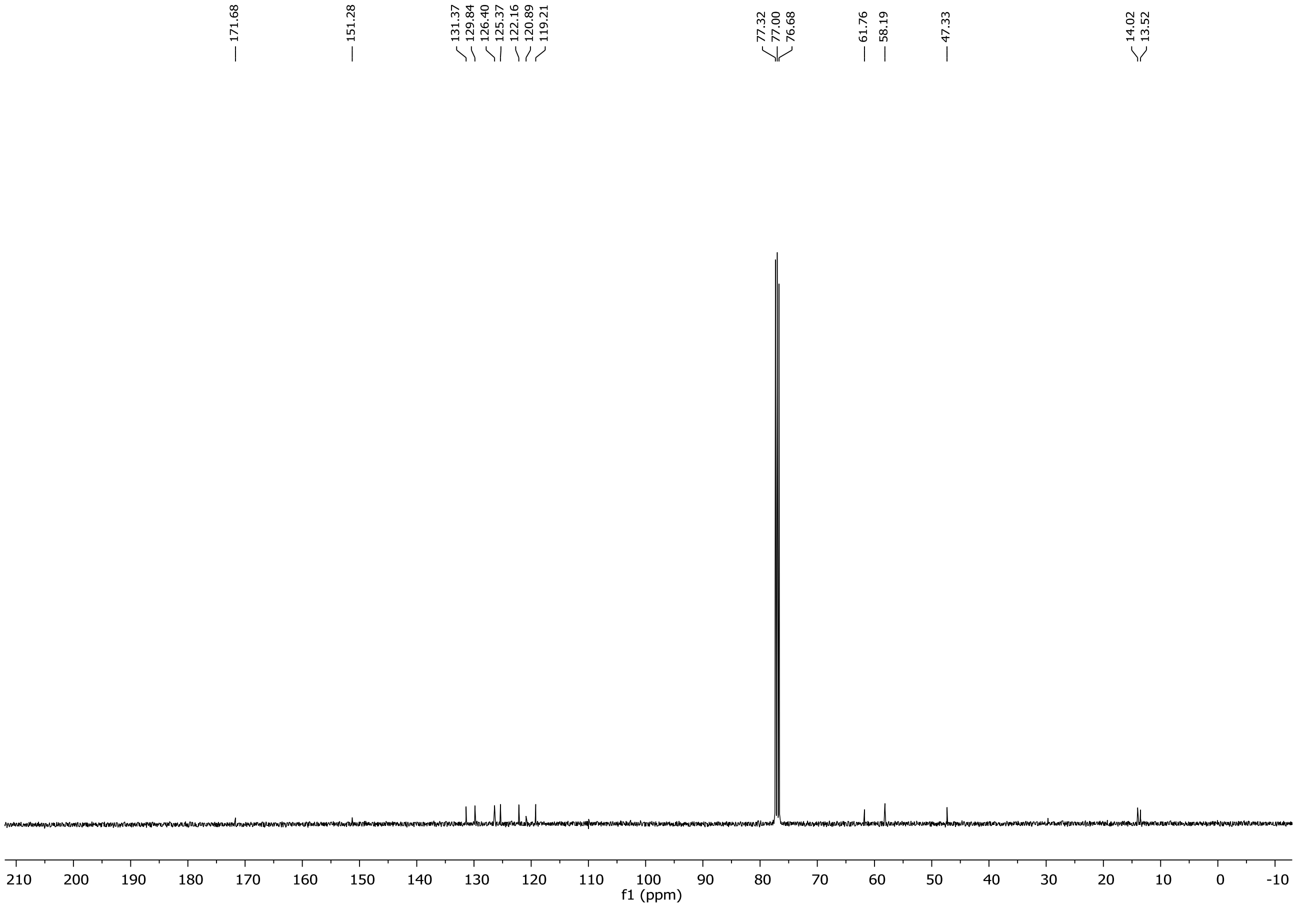
171.68
151.28
131.37
129.84
126.40
125.37
122.16
120.89
119.21
77.32
77.00
76.68
61.76
58.19
47.33
14.02
13.52
210
200
190
180
170
160
150
140
130
120
110
100
90
80
70
60
50
40
30
20
10
0
-10
f1 (ppm)

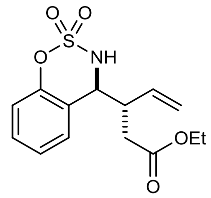

7 (10:1 mixture of diastereomers)

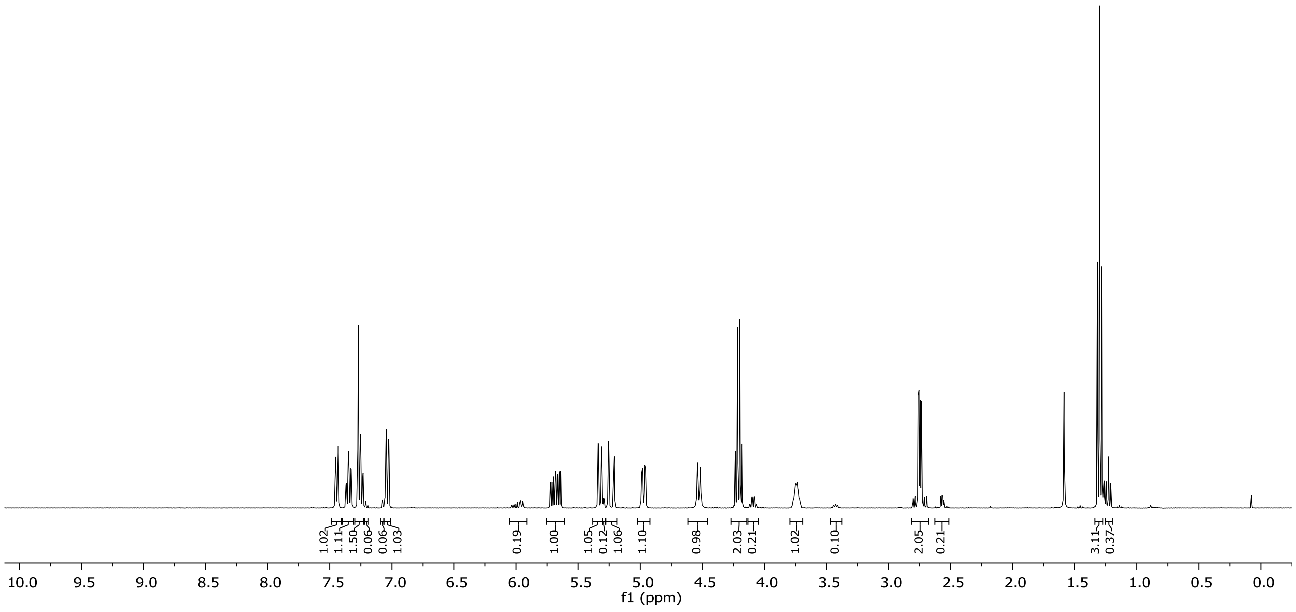

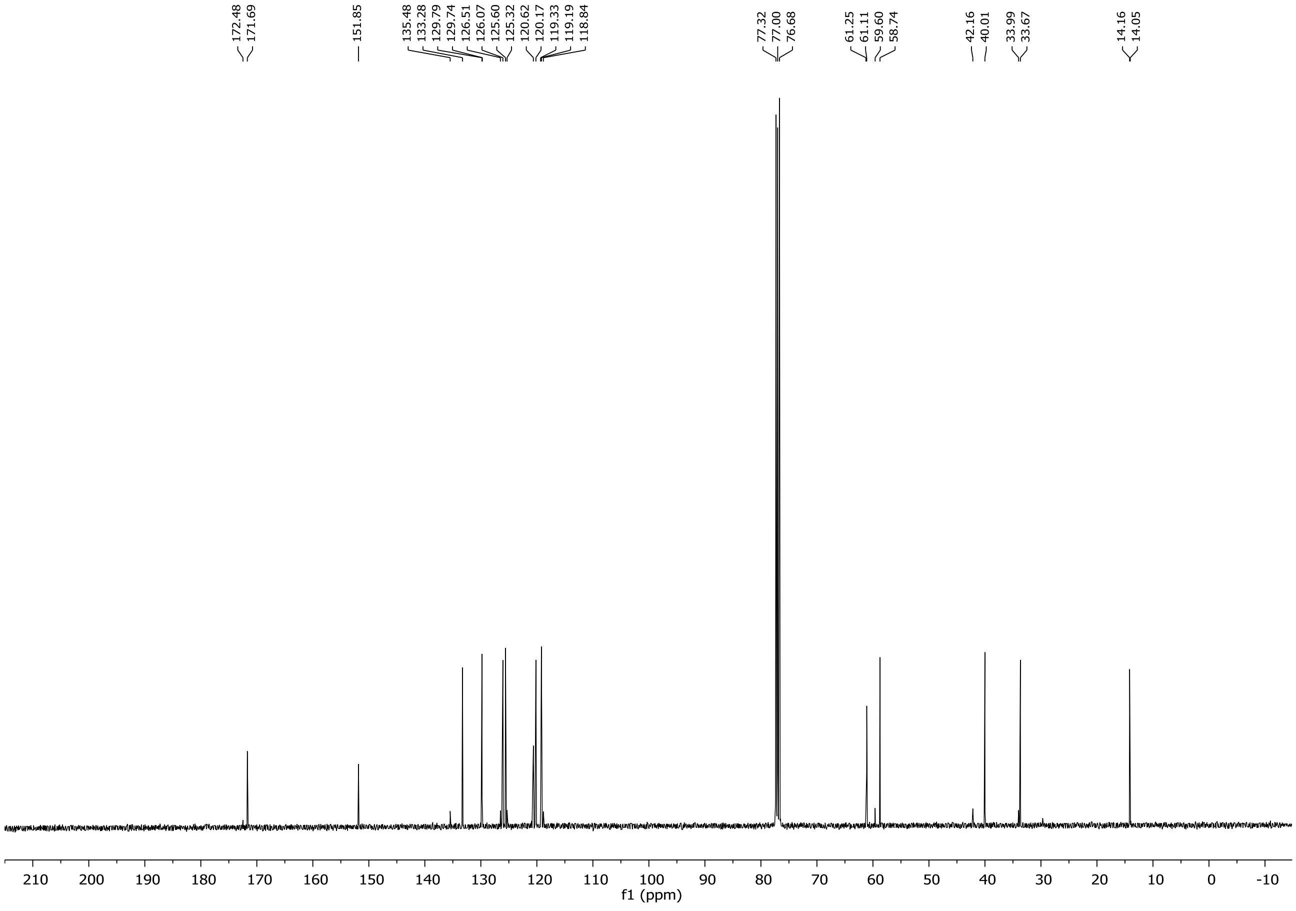

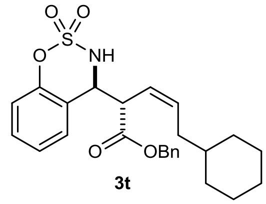

3t

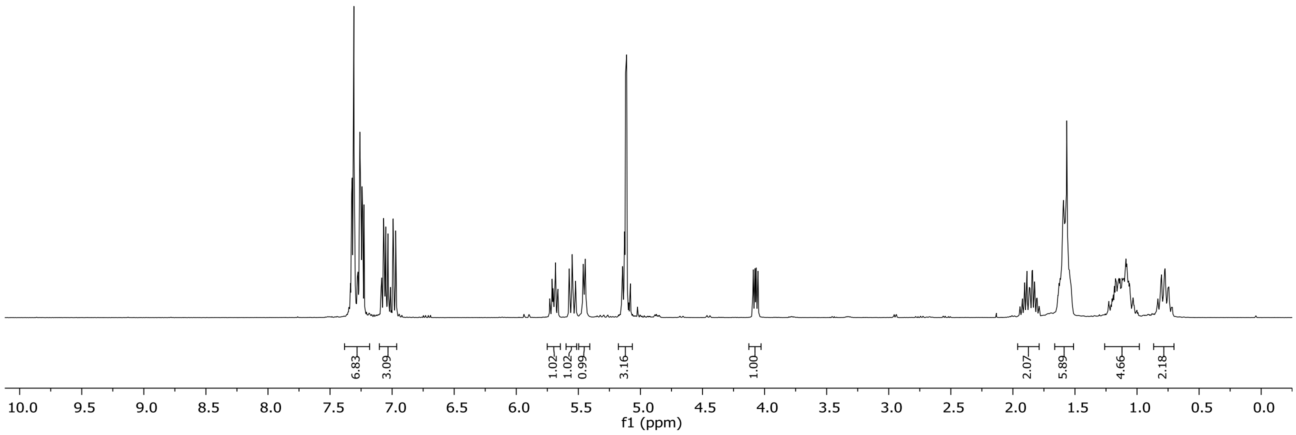

6.83
3.09
1.02
1.02
0.99
3.16
1.00
2.07
5.89
4.66
2.18
10.0 9.5 9.0 8.5 8.0 7.5 7.0 6.5 6.0 5.5 5.0 4.5 4.0 3.5 3.0 2.5 2.0 1.5 1.0 0.5 0.0
f1 (ppm)

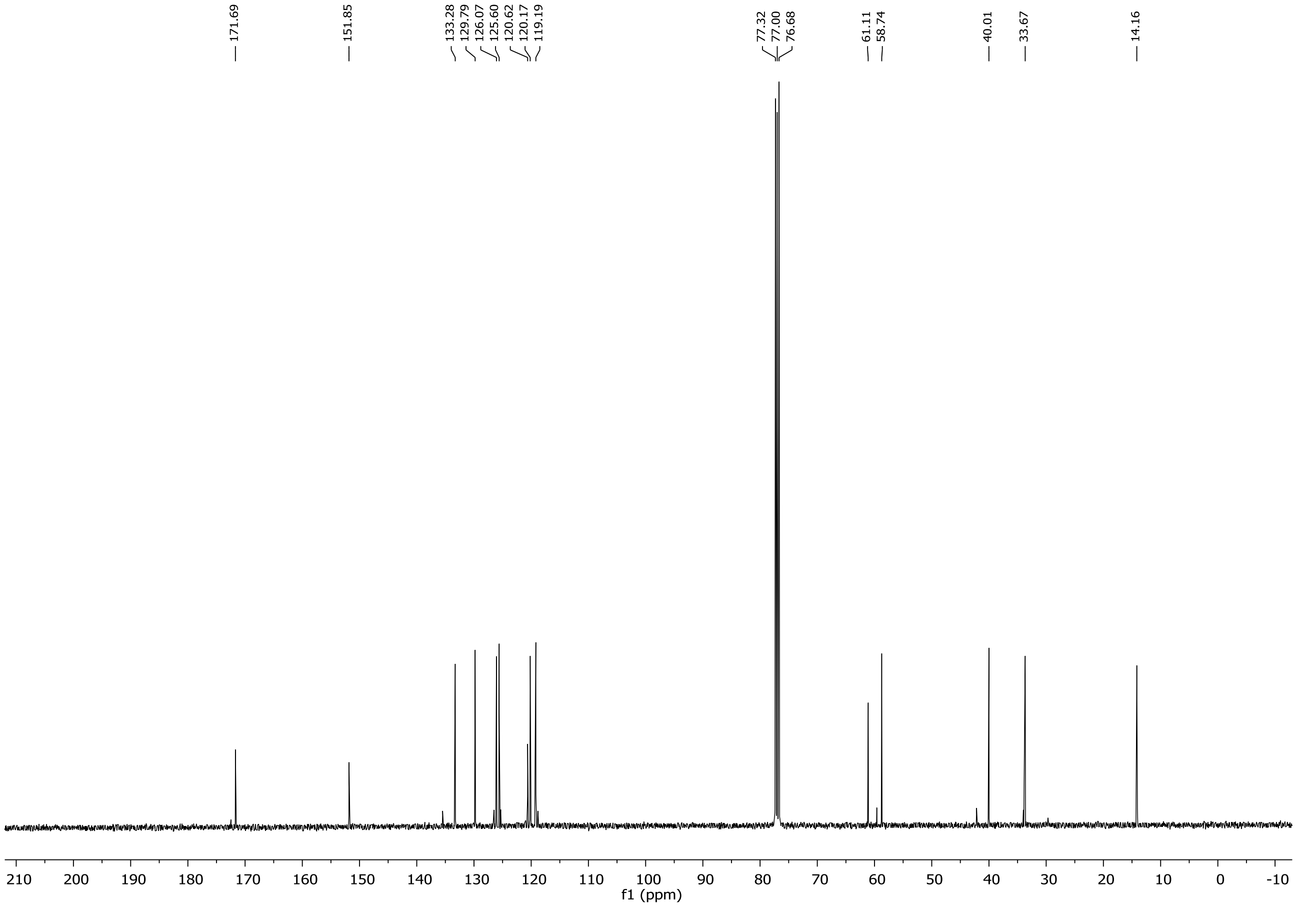

171.69
151.85
133.28
129.79
126.07
125.60
120.62
120.17
119.19
77.32
77.00
76.68
61.11
58.74
40.01
33.67
14.16
210 200 190 180 170 160 150 140 130 120 110 100 90 80 70 60 50 40 30 20 10 0 -10
f1 (ppm)
